# The tremendous potential of deep-sea mud as a source of rare-earth elements

**DOI:** 10.1038/s41598-018-23948-5

**Published:** 2018-04-10

**Authors:** Yutaro Takaya, Kazutaka Yasukawa, Takehiro Kawasaki, Koichiro Fujinaga, Junichiro Ohta, Yoichi Usui, Kentaro Nakamura, Jun-Ichi Kimura, Qing Chang, Morihisa Hamada, Gjergj Dodbiba, Tatsuo Nozaki, Koichi Iijima, Tomohiro Morisawa, Takuma Kuwahara, Yasuyuki Ishida, Takao Ichimura, Masaki Kitazume, Toyohisa Fujita, Yasuhiro Kato

**Affiliations:** 1 Department of Resources and Environmental Engineering School of Creative Science and Engineering, Waseda University, 3-4-1 Okubo, Shinjyuku, Tokyo 169-8555 Japan; 2 Research and Development Center for Submarine Resources, Japan Agency for Marine-Earth Science and Technology (JAMSTEC), 2-15 Natsushima-cho, Yokosuka, Kanagawa 237-0061 Japan; 3 Frontier Research Center for Energy and Resources, School of Engineering, The University of Tokyo, 7-3-1 Hongo, Bunkyo-ku, Tokyo 113-8656 Japan; 4 Ocean Resources Research Center for Next Generation, Chiba Institute of Technology, 2-17-1 Tsudanuma, Narashino, Chiba 275-0016 Japan; 5 Department of Systems Innovation, School of Engineering, The University of Tokyo, 7-3-1 Hongo, Bunkyo, Tokyo 113-8656 Japan; 6 Department of Solid Earth Geochemistry, Japan Agency for Marine-Earth Science and Technology (JAMSTEC), 2-15 Natsushima-cho, Yokosuka, Kanagawa 237-0061 Japan; 7 Department of Deep Earth Structure and Dynamics Research, Japan Agency for Marine-Earth Science and Technology (JAMSTEC), 2-15 Natsushima-cho, Yokosuka, Kanagawa 237-0061 Japan; 8 Department of Planetology, Graduate School of Science, Kobe University, 1-1 Rokkodai, Nada, Kobe, Hyogo 657-8501 Japan; 9 Engineering Project Department, Toa Corporation, 3-7-1 Nishi-Shinjuku, Shinjuku, Tokyo 163-1031 Japan; 10 Research and Development Center, Toa Corporation, 1-3 Anzen, Tsurumi, Yokohama, Kanagawa 230-0035 Japan; 11 Central Research Laboratory, Taiheiyo Cement Corporation, 2-4-2 Osaku, Sakura, Chiba 285-8655 Japan; 12 Department of Civil and Environmental Engineering, Tokyo Institute of Technology, 2-12-1 O-okayama, Meguro, Tokyo 152-8552 Japan

## Abstract

Potential risks of supply shortages for critical metals including rare-earth elements and yttrium (REY) have spurred great interest in commercial mining of deep-sea mineral resources. Deep-sea mud containing over 5,000 ppm total REY content was discovered in the western North Pacific Ocean near Minamitorishima Island, Japan, in 2013. This REY-rich mud has great potential as a rare-earth metal resource because of the enormous amount available and its advantageous mineralogical features. Here, we estimated the resource amount in REY-rich mud with Geographical Information System software and established a mineral processing procedure to greatly enhance its economic value. The resource amount was estimated to be 1.2 Mt of rare-earth oxide for the most promising area (105 km^2^ × 0–10 mbsf), which accounts for 62, 47, 32, and 56 years of annual global demand for Y, Eu, Tb, and Dy, respectively. Moreover, using a hydrocyclone separator enabled us to recover selectively biogenic calcium phosphate grains, which have high REY content (up to 22,000 ppm) and constitute the coarser domain in the grain-size distribution. The enormous resource amount and the effectiveness of the mineral processing are strong indicators that this new REY resource could be exploited in the near future.

## Introduction

**Figure 1:**
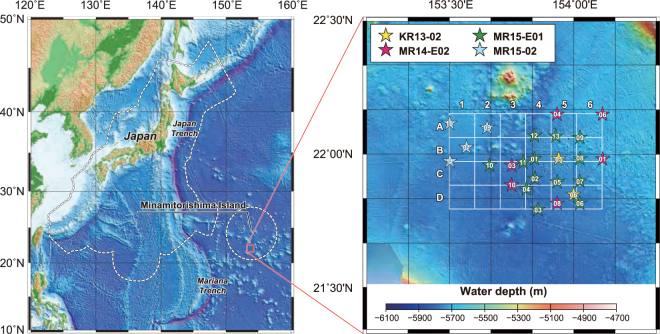
Locality and bathymetric maps of the research area. Star symbols show the piston coring sites, and the color-coding corresponds to each research cruise as noted in the legend. The white rectangle shown in the detailed map is the target area where the resource amount estimation was conducted. Bathymetric data were obtained from ETOPO1 (NOAA’s National Centers for Environmental Information; https://www.ngdc.noaa.gov/mgg/global/global.html). Those in right panel were obtained by each research cruises mentioned in the text. Both maps were created by using the Generic Mapping Tools (GMT) software (https://www/soest.hawaii.edu/gmt/), Version 4.5.8^29^.

Rare-earth elements and yttrium (REY) are critical materials to many leading-edge technologies due to their unique physical and chemical properties. Applications of REY span a wide range, including hybrid vehicles, rechargeable batteries, wind turbines, light emitting diodes, compact fluorescent lamps, screen display panels, and many medical and military technologies^1,2^. The industrial utility of REY, especially in renewable energy technologies and electronics, has driven up the demand for the essential metals in recent years^3–6^. Amid rapidly increasing global demand for REY, Kato *et al*.^7^ reported the discovery of REY-rich mud, which has a high total REY content (up to 2,230 ppm) and is widely distributed on deep-sea floor in the Pacific Ocean. Subsequently, in 2013, highly to extremely REY-rich mud (deep-sea sediments containing 2,000 ppm to more than 5,000 ppm REY) was found within the Japanese exclusive economic zone (EEZ) around Minamitorishima Island (Marcus Island) during the KR13-02 cruise of the deep-sea research vessel *KAIREI* operated by the Japan Agency for Marine-Earth Science and Technology (Fig. 1)^8,9^. REY-rich mud has several advantages, such as high rare earth element content (especially the heavy rare-earth elements [HREE], from Eu to Lu), huge amounts, a paucity of radioactive elements (U and Th), and easy extraction and recovery. Therefore, the mud is expected to be viewed as a highly promising new mineral resource^7,10^. Because of the “extremely high grade” of the REY-rich mud found within the Japanese Minamitorishima EEZ, research on a development system and economic evaluations are ongoing through collaborations among members of industry, academia, and the government of Japan. In this study, we estimated in detail the resource potential of REY-rich mud using Geographical Information System (GIS) software and proposed appropriate mineral processing techniques that can effectively enhance its economic value.

## Results and Discussion

### Resource amount estimation

**Figure 2:**
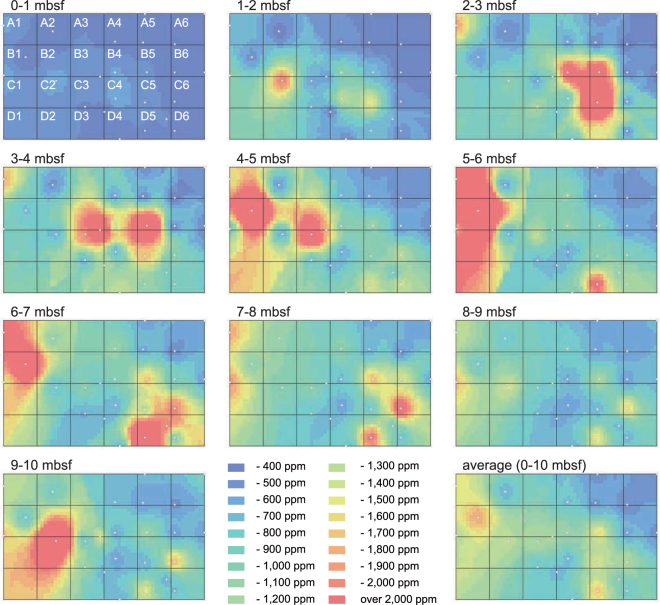
Concentration maps of average ΣREY of mud from the seafloor to 10 mbsf and of each 1-m depth interval. The target area (Fig. 1) is divided into 24 areas (A1–D6). The maps were generated by ArcGIS and are shown with 2,400 grids (60 × 40). Coring sites are shown as white circles.

After the KR13-02, MR13-E02, and KR14-02 cruises, three additional research cruises (MR14-E02, MR15-E01 Leg 2, and MR15-02) were conducted to reveal the detailed distribution of highly to extremely REY-rich mud in the southern part of the Minamitorishima EEZ (Fig. 1)^8,9^. During these cruises, REY-rich mud having a maximum of almost 8,000 ppm of total REY content (ΣREY) was confirmed. We estimated the resource amount of REY in the region bounded by 21°48′N to 22°15′N and 153°30′E to 154°07′E (about 2,500 km^2^) by using whole sediment chemical data of newly analysed 573 samples and previously reported 104 samples (KR13-02 PC05: 82 samples^8^ and KR13-02 PC06: 22 samples)^9^ from 25 sampling points (Supplementary Tables S1–S2, Fig. 1). Geographical Information System software (ArcGIS) was used to visualise the REY-rich mud distribution and evaluate its resource potential. ΣREY maps of the average concentration values from the seafloor to 10 meters below the seafloor (mbsf) and of the values for each 1 m depth interval are shown in Fig. 2. The ΣREY map was further divided into 24 grid squares (rows A to D and columns 1 to 6, A1–D6). The calculated ΣREY values and resource potential of each grid are listed in Table 1. In addition, Supplementary Table S3 shows the average ΣREY and the total resource amount from the seafloor to each target depth. There is a vast (over 400 km^2^) area high in REY in the northwest part of the research area (see the panels for 5–6 and 6–7 mbsf in Fig. 2), which continues loosely to the southeast (see the average panel map in Fig. 2). ΣREY is relatively low in the basin in the middle of the southern area and in the topographical high area in the northeast. The calculated ΣREY for the entire research area is more than 16 million tons of rare-earth oxides (Mt-REO) (average ΣREY = 964 ppm). In addition, the mud is especially enriched in Y and HREE, which accounted for 44% (Y: 4.4 Mt-REO; HREE: 2.6 Mt-REO) of the total amount of REY in this region. The research area was estimated to be able to supply Y, Eu, Tb, and Dy for 780, 620, 420, and 730 years, respectively, and has the potential to supply these metals on a semi-infinite basis to the world^11^. Of the divided areas, B1 (9.9 km × 10.6 km = 105 km^2^) shows the highest REY resource potential, with an average ΣREY of more than 1,700 ppm. This area includes MR15-02 PC01, where extremely REY-rich mud was confirmed, and the ΣREY of the 5–6 mbsf interval exceeded 5,600 ppm. The resource amount of area B1 was estimated to be 1.2 Mt-REO, which would account for 62, 47, 32, and 56 years of annual global demand for Y, Eu, Tb, and Dy, respectively (Supplementary Table S4)^11^.

**Table 1:** Average ΣREY and resource amount of each interval for each grid (A1–D6).

Area	[km^2^]	ΣREY [ppm]											Resource amount of REY [REO-t/km^2^]										
		0–1 m	1–2 m	2–3 m	3–4 m	4–5 m	5–6 m	6–7 m	7–8 m	8–9 m	9–10 m	aver-age	0–1 m	1–2 m	2–3 m	3–4 m	4–5 m	5–6 m	6–7 m	7–8 m	8–9 m	9–10 m	sum
A1	104.9	443	651	1,179	1,223	1,892	2,662	2,060	1,449	1,133	1,209	1,390	288	423	765	794	1,229	1,727	1,337	941	736	786	9,027
A2	104.9	316	361	415	482	510	594	1,060	1,004	769	826	634	205	235	270	314	332	386	689	652	500	537	4,120
A3	104.9	393	609	836	1,177	1,143	895	966	914	807	1,040	878	255	396	543	765	742	581	627	594	524	675	5,703
A4	104.9	339	427	710	812	670	862	924	863	733	677	702	221	278	461	528	435	560	600	561	477	440	4,559
A5	104.9	313	346	522	637	519	535	646	690	586	581	538	204	225	339	414	337	348	420	448	381	377	3,493
A6	104.9	287	307	359	402	444	479	525	594	562	614	457	186	200	233	261	288	311	341	386	365	399	2,972
B1	104.9	332	521	578	723	3,677	5,662	2,693	1,107	890	899	1,708	216	339	375	470	2,388	3,674	1,748	719	578	584	11,092
B2	104.9	447	1,091	719	939	1,627	1,794	1,250	1,006	1,056	2,108	1,204	291	709	467	610	1,056	1,164	812	653	686	1,368	7,815
B3	104.9	450	708	1,007	1,970	1,968	1,008	881	873	814	1,206	1,089	292	460	654	1,279	1,277	654	572	567	529	783	7,067
B4	104.9	453	754	1,722	1,472	902	927	962	817	691	715	942	295	490	1,118	956	586	602	624	531	449	464	6,114
B5	104.9	384	548	1,638	2,078	789	710	1,127	1,198	821	790	1,008	250	356	1,063	1,349	512	461	731	778	533	513	6,546
B6	104.9	295	335	487	630	532	524	752	996	646	635	583	192	218	316	409	346	341	488	647	419	413	3,788
C1	105.0	479	941	824	876	1,721	2,514	1,687	1,413	1,295	1,684	1,343	311	611	536	569	1,118	1,632	1,095	917	841	1,094	8,725
C2	105.0	520	1,593	900	862	1,090	1,014	864	939	1,203	3,058	1,204	338	1,035	585	560	708	659	561	609	781	1,985	7,819
C3	105.0	439	675	806	1,592	2,088	954	797	842	762	1,045	1,000	285	438	524	1,033	1,354	619	517	547	494	678	6,491
C4	105.0	552	1,127	1,293	1,068	717	673	730	639	573	600	797	359	732	839	694	465	437	474	415	372	390	5,178
C5	105.0	421	912	2,750	1,728	967	933	1,613	1,301	882	893	1,240	274	592	1,785	1,122	628	606	1,047	845	573	580	8,052
C6	105.0	324	451	788	771	898	766	1,387	1,506	971	1,021	888	211	293	512	501	583	498	900	978	631	664	5,770
D1	105.1	457	962	828	898	1,631	1,986	1,318	1,143	1,112	1,700	1,204	297	625	538	584	1,059	1,289	856	742	722	1,104	7,815
D2	105.1	450	961	907	1,160	1,190	757	705	783	833	1,594	934	292	624	589	754	772	491	458	508	541	1,034	6,063
D3	105.1	384	622	926	1,081	961	721	650	695	649	870	756	250	404	602	702	624	468	422	451	421	564	4,908
D4	105.1	378	672	1,201	1,147	884	951	1,155	841	709	724	866	245	436	780	745	574	617	749	546	460	470	5,623
D5	105.1	396	668	1,607	1,028	1,136	1,458	2,600	1,377	1,011	983	1,226	257	434	1,044	668	737	946	1,685	893	656	638	7,960
D6	105.1	352	519	942	869	1,147	1,108	1,545	1,124	839	862	931	229	338	612	565	745	719	1,002	730	545	560	6,046

### Determination of the host mineral of REY

**Figure 3:**
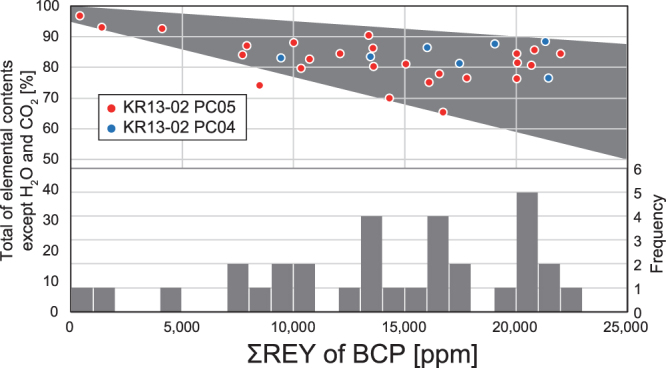
ΣREY in BCP grains determined by EPMA and LA-ICP-MS. The vertical axis shows the total value [%] of the analysis (left axis) and the frequency of the samples (right axis). A moderate negative correlation (gray shaded area) can be observed between ΣREY in BCP and the total value.

In addition to the huge resource amount, it will be possible to enhance the economic value of the REY-rich mud by selectively recovering the host mineral of REY in the mud and thereby significantly improving the ore grade. Kashiwabara *et al*.^12^ determined that the host mineral of REY in the REY-rich mud distributed in the eastern South Pacific Ocean is an apatitic mineral phase (composed of calcium phosphates) by using X-ray absorption fine structure and micro-focused X-ray fluorescence analyses. In addition, it has long been held that some trace elements, including REY, are substantially adsorbed by biogenic calcium phosphates (BCP) in marine sediment after their deposition^13,14^. Here, we conducted electron probe microanalyser (EPMA) and laser ablation-inductively coupled plasma-mass spectrometry (LA-ICP-MS) analyses of 32 BCP (KR13-02 PC04: 7 samples, PC05: 25 samples) and 9 phillipsite samples (KR13-02 PC05), which are ubiquitous in highly to extremely REY-rich mud (Supplementary Table S5). Kon *et al*.^15^ also conducted *in-situ* chemical analyses of BCP grains in REY-rich mud, although their sampling locations and stratigraphic positions were not clarified. In contrast, spatial information is well documented for our samples (Supplementary Table S5). Our measurements show that the average ΣREY in BCP exceeds 15,000 ppm (up to 22,000 ppm), and that BCP can generally account for the total REY in highly/extremely REY-rich mud (Fig. 3). A negative correlation was observed between the ΣREY of BCP and the total value of elemental contents, determined by EPMA (major elements) and LA-ICP-MS (minor elements) analyses (Fig. 3). In the EPMA analysis, H_2_O and CO_2_ were the main constituent elements of total deficit (low total from 100%). Therefore, Fig. 3 indicates that H_2_O and CO_2_ in BCP grains increase with progressing of REY uptake, owing to the successive interaction between BCP and seawater and/or pore water. The potential of BCP to uptake REY could be variable depending on the diffusive coefficient and susceptibility to diagenesis of each grain^16^. Difference in these factors may be attributable to mineralogical features of the grains, such as lattice defects or crystallinity^17^. They can differ among body regions in each organism (e.g., dentin versus enamel of fish teeth)^16,17^ or species of organisms. The broad negative correlation probably reflects such variability (Fig. 3). On the other hand, most of the phillipsite grains contained less than 100 ppm of ΣREY. In a pelagic environment, phillipsite appears to be generated by the alteration of volcanic glasses^18,19^. The requisite environmental condition for the formation of phillipsite in marine sediment (i.e., a sufficiently low sedimentation rate) is similar to that of pelagic clay enriched in BCP grains having a high REY content^20–22^. Hence, phillipsite merely co-occurs with BCP in marine sediment, resulting in a spurious correlation between the amount of phillipsite and ΣREY in the mud.

### Grain size separation with test sieves

**Figure 4:**
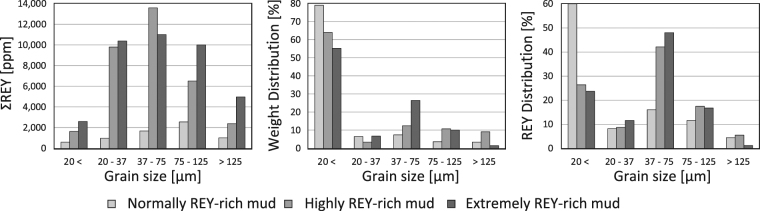
ΣREY, weight distribution, and REY amount distribution [%] of each fraction obtained from the grain-size separation experiment with test sieves.

The BCP grains in marine sediment and in highly to extremely REY-rich mud generally are larger than the grains of other constituent minerals^23^. Toyoda *et al*.^14^ conducted grain-size separation of marine sediment from the central Pacific (17°06′N, 146°12′W) and divided it into 6 grain-size categories (<1 µm, <2 µm, 2–10 µm, 10–38 µm, 38–100 µm, and >100 µm). They concluded that the larger fractions (10–38 µm and 38–100 µm) have remarkably high Ca, P, and REY contents as compared with the bulk composition and the other fractions, primarily because of an abundance of BCP grains in these larger fractions. This result indicates that it is possible to selectively collect REY-enriched BCP grains by using size separation techniques. We, therefore, conducted grain-size separation experiments to elucidate the weight, ΣREY, and REY distribution in each grain-size fraction and to demonstrate the effectiveness of grain-size separation. Three REY-rich mud samples from core KR13-02 PC05 were used in the experiments: a “normally” REY-rich mud (ΣREY = 795 ppm, 1.94–2.10 mbsf), a “highly” REY-rich mud (ΣREY = 3,950 ppm, 2.62–2.78 mbsf), and an “extremely” REY-rich mud (ΣREY = 7,226 ppm, 3.08–3.24 mbsf). The experiments were conducted with polypropylene test sieves (new Perlon Sieves, Ito-Seisakusho) with openings of 20, 37, 75, and 125 µm. The ΣREY was the lowest for the smallest grain-size fraction (<20 µm) in all of the REY-rich mud samples (normal: 589 ppm; high: 1,651 ppm; extreme: 2,586 ppm) (Supplementary Table S6). ΣREY greatly increased with increased grain-size for all samples, and the highest ΣREY was observed at 37–75 µm in normally REY-rich mud and at 75–125 µm in highly and extremely REY-rich mud (Fig. 4). The ΣREY of the mud decreased in the >125 µm size fraction (normal: 1,020 ppm; high: 2,404 ppm; extreme: 4,897 ppm), most likely because of an increase in manganese oxide components such as micro Mn-nodules. Moreover, the weight distribution was greatest in the smallest size fraction (<20 µm) for all samples (normal: 79%; high: 64%; extreme: 55%) (Fig. 4). The ΣREY of each size fraction indicates that most of BCP grains are distributed in the >20 µm size fractions. When the >20 µm fraction was selectively collected, the grade of normally, highly, and extremely REY-rich mud increased from 795 ppm to 1,496 ppm (recovery ratio of REY: 40.2%), from 3,950 ppm to 8,235 ppm (73.6%), and from 7,226 ppm to 10,360 ppm (77.4%), respectively. Our experimental data strongly suggest that the selective recovery of BCP by grain-size separation is an easily applicable method to increase REY content of marine sediment that could be used in any ocean area.

### Mineral processing by a hydrocyclone separator

**Figure 5:**
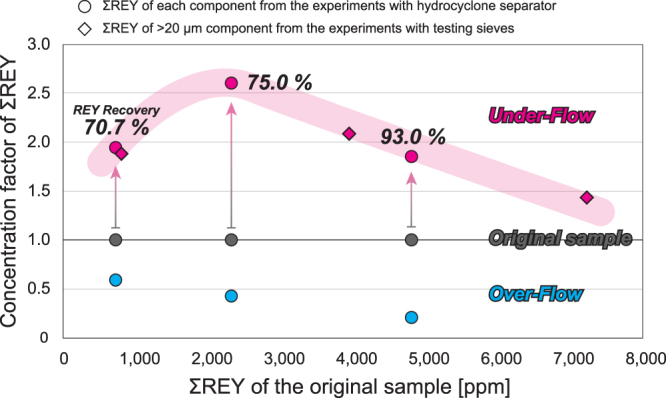
Concentration factor of ΣREY through the grain-size separation experiments with test sieves (ΣREY of the >20 µm component/ΣREY of the original sample) and the hydrocyclone separator (ΣREY of the under-flow component/ΣREY of the original slurry sample). Data of the original sample, under-flow component, and over-flow component are shown in gray, red, and blue, respectively. The pink shaded area shows the expected concentration factor of grain-size separation with respect to ΣREY of the original sample/slurry.

We conducted additional separation experiments of REY-rich mud with a hydrocyclone separator, which would be an applicable technology on an industrial scale^24^. A hydrocyclone separator is a device that can separate heavy/large and light/small components from a flowing solid-liquid mixture (slurry) by centrifugal force, and a Super-30-Cyclone (Nihon Bunri Co.) was used in our experiments. Based on the grain-size separation experiments with test sieves, the experimental conditions of the hydrocyclone separator were coordinated to separate the >15–20 µm grains from the slurry. Three REY-rich mud samples from MR15-02 PC01 cored at 22°02′N and 153°34′E were used in the experiments (Supplementary Table S7). The ΣREY of the original slurry samples were 722 ppm (normally REY-rich mud: MR15-02 PC01, 2.28–4.28 mbsf), 2,315 ppm (highly REY-rich mud: 6.27–7.05 mbsf), and 4,802 ppm (although this was less than 5,000 ppm, it was considered as an extremely REY-rich mud: 4.28–6.28 mbsf). Supplementary Fig. S1(A) shows the particle size distribution of the over-flow (OF, waste flow) and under-flow (UF, collection flow) components. The nodes of the fraction curves are roughly 15–20 μm in all cases, and separation by the hydrocyclone was generally well performed; the imperfection is calculated to be 0.74 for normally REY-rich mud, 0.38 for highly REY-rich mud, and 0.38 for extremely REY-rich mud, respectively (Supplementary Fig. S1). The ΣREY values of the OF and UF components were 429 ppm and 1,401 ppm for normally REY-rich mud, 997 ppm and 6,031 ppm for highly REY-rich mud, and 994 ppm and 8,902 ppm for extremely REY-rich mud, respectively (Supplementary Table S7). The recovery ratio of REY in UF were 70.7%, 75.0%, and 93.0% for the normally, highly, and extremely REY-rich muds, respectively (Fig. 5). The concentration factor of ΣREY through the mineral processing (the ratio of ΣREY in UF to ΣREY in the original slurry) had the highest value at a ΣREY of about 2,000–3,000 ppm in the original mud sample (Fig. 5). The improved ore grade gained via grain-size separation with test sieves and hydrocyclone treatment indicates that the separation is both an effective and predictable method for preliminary mineral processing of REY-rich mud. The ΣREY of the UF for highly REY-rich mud increased to 260% of the value of the original sample. In addition, the hydrocyclone can reduce the slurry volume in UF to less than one-fifth of the original value and mud weight to 42.5%, 33.1%, and 59.8% of the original sample weights of the normally, highly, and extremely REY-rich mud, respectively. Because the amount of the resource is enormous, improving the ore grade will greatly enhance the economic value of the mud even if the recovery yield is somewhat lower than that we observed. A decrease in mud weight and volume will directly lead to reductions in smelting costs. Moreover, if a hydrocyclone can be operated *in-situ* on the deep-sea floor, it would be possible to reduce lifting costs, which would further contribute to improving the economic efficiency of any development project.

In summary, our results demonstrate the enormous resource amount of REY-rich in the western North Pacific Ocean. In addition, a hydrocyclone separator can greatly enhance the economic value of REY-rich mud by taking the advantage of mineralogical features of the mud, which could stimulate future exploitation of this new deep-sea mineral resource. Given the huge resource amount, its high grade (notably Y and HREEs), and the effectiveness of simple grain-size separation with a hydrocyclone, we believe that the REY-rich mud has great potential as ore deposits for some of the most critically important elements in the modern society.

## Methods

### Chemical analysis of bulk sediment

The geochemical composition of REY-rich mud was measured by inductively coupled plasma-quadrupole mass spectrometry (ICP-QMS, Agilent 7500 c, Agilent Technologies, Santa Clara, USA) at the University of Tokyo in accordance with the method described in Kato *et al*.^25^ and Yasukawa *et al*.^26^. A 0.05 g powdered sample was digested in a solution of 0.8 mL HClO_4_, 2 mL HF, and 4 mL HNO_3_ at 130 °C for 24 h. After this mixed acid solution was dried by stepwise heating, 5 mL inverse aqua regia and 5 mL Milli-Q water (18.2 MΩ electrical resistivity) were added and then heated at 110 °C overnight. Finally, the sample solution was diluted with Milli-Q water and used for the ICP-QMS analysis.

### Resource amount estimation

ArcGIS (ArcMAP 10.4) software was used to visualise the REY-rich mud distribution and evaluate the resource amount of rare-earth elements. First, we calculated the average concentration of each element for each 1-m interval from 0 to 10 mbsf using ICP-QMS chemical analysis data. Coring sites (latitude and longitude) and depths are listed in Supplementary Tables S1 and S2. At MR14-E02 PC10, the maximum sampling depth was 9.347 mbsf. Therefore it was assumed that mud with the same chemical composition was distributed from 9.347 mbsf to 10 mbsf in this core. ΣREY maps were created based on the inverse distance weighted (IDW) method by using the above-mentioned average concentrations of each depth interval. Concentration maps are displayed as 2,400 small grid cells (40 × 60) in Fig. 2. The REY resource amounts were calculated for each of the 24 larger grids cells (10 × 10 small cells) (A1–D6) and are listed in Table 1. The dry bulk density of REY-rich mud was set as 0.54 g/cm^3^ (±0.05 g/cm^3^ [SD], *n* = 69) based on the measurements of REY-rich mud samples taken from the research area. To confirm the validity of the generated ΣREY maps, we also reproduced a seabed topographic map with seafloor depth data of each coring point (Supplementary Table S1) by the IDW method and compared this map to the actual submarine topographic map (Supplementary Fig. S2). As a result, except for irregular unevenness of the seafloor, such as on or near seamounts, the reproduced topography map was similar to the actual one. This confirms the validity of the IDW method for our dataset.

### EPMA and LA-ICP-MS analyses of BCP and phillipsite grains

Major element compositions including S, Cl, F, and SrO of BCP and phillipsite grains were analysed using a JXA-8800 K (JEOL, Tokyo, Japan) electron probe microanalyser (EPMA) at the Japan Agency for Marine-Earth Science and Technology (JAMSTEC). The minerals were molded in an epoxy mount with a diameter of 1 inch (2.5 cm) and polished to expose the internal surfaces of the minerals. The samples were carbon-coated and analysed under the operating conditions of 15 kV and 1.0 × 10^−8^ A or 1.2 × 10^−8^ A. A ZAF method was employed for data corrections. Analytical results are listed in Supplementary Table S5. After the EPMA analyses, 43 major and trace element compositions of the same samples were analysed by laser ablation-inductively coupled plasma-mass spectrometry (LA-ICP-MS) at JAMSTEC. An in-house developed 200-nm femtosecond LA system (OK Fs-2000 K, OK Lab, Tokyo, Japan) was coupled to a modified sector field-ICP-MS (Element XR, Thermo Fisher Scientific, Bremen, Germany). The oxide molecular yield measured by ThO^+^/Th^+^ was <0.3%, and elemental sensitivities were typically 10–360 cps ppm^−1^ for trace elements and 5,000–950,000 cps wt%^−1^ for major elements with a laser crater size of ~20 μm in diameter. Time-resolving data acquisition was used for all of the analyses, and the total oxide sum method was employed for correction of laser sampling efficiency. Instrumentation and analytical protocols, including LA and ICP-MS operating conditions, data acquisition, and data reduction were the same as detailed in Kimura and Chang^27^. The standard material used was Standard Reference Material SRM 610 synthetic glass provided by the National Institute of Standard and Technology (NIST). The reference values were from the GeoReM geostandard database (http://georem.mpch-mainz.gwdg.de/; Jochum *et al*.)^28^. Finally, all of the element abundances determined by LA-ICP-MS were normalised to the CaO content determined by EPMA to maintain internal consistency of the analytical data (Supplementary Table S5).

### Mineral processing by a hydrocyclone separator

The original slurry sample was made by mixing a marine sediment sample and “Aquamarine” synthetic seawater (Yashima Pure Chemicals Co., Ltd., Osaka, Japan; density: 1.02 g/cm^3^). After mixing, coarser grains such as rock fragments and manganese nodules (>1 mm) were removed from the slurry by sieving. The slurry density was adjusted to 12% based on the measured particle density of 2.7 g/cm^3^. The original slurry was put into a pressure container and injected into a hydrocyclone (SUPER-30-CYCLONE, Nihonbunri Co., Saitana, Japan) at 0.3 MPa. The diameter of the under-nozzle was set to 4.0 mm. The flow rate and the density of the output slurry (over-flow: OF; under-flow: UF) were measured during the experiments. A 10 ml sample of each slurry was collected for particle size distribution measurement and chemical analysis. The particle size distribution was measured with a laser diffraction grain-size distribution measuring apparatus (Microtrac MT3000, MicrotracBEL Co., Osaka, Japan) at the University of Tokyo. The particle size distribution of the OF and UF components and the partition curves are shown in Supplementary Fig. S1. The efficiency of separation *(I: imperfection)* can be expressed by taking the grain sizes at which 75%, 50% and 25% of the feed particles report to UF (d_75_, d_50_, d_25_) (Supplementary Fig. S1 (B)), and is given by the following equation^24^;

1 $${\rm{I}}=\frac{{d}_{75}-{d}_{25}}{2{d}_{50}}$$

The imperfection is calculated to be 0.74 for normally REY-rich mud, 0.38 for highly REY-rich mud, and 0.38 for extremely REY-rich mud, respectively.

All of the chemical analyses of the slurry samples were conducted by ICP-QMS (iCAP Q: Thermo Fisher Scientific, Bremen, Germany) at the University of Tokyo. A dried and powdered 0.05 g sample was digested with a solution of 0.8 mL HClO_4_, 2 mL HF, and 4 mL HNO_3_ at 130 °C for 2 h. After this mixed acid solution was dried by stepwise heating, 1.5 ml HCl and 0.5 ml HNO_3_ were added and the solution was heated at 90 °C for 3 h. The mixed acid solution was then dried by stepwise heating up to 160 °C, and a 10 ml mixed acid solution (2 wt%, HNO_3_:HCl:HF = 20:5:1) was added to this dry sample and heated at 90 °C for 3 h. Finally, the sample solution was diluted with a 2 wt% mixed acid solution and used for the ICP-QMS analysis. In the chemical analyses of the slurry samples, the slurry was dried with synthetic seawater because it is difficult to recover suspended fine-grained matter from the slurry. Hence, the chemical composition of the soil particles listed in Supplementary Table S7 includes seawater-derived elements (such as NaCl) and underestimates the other elements. In the case of the experiments with normally REY-rich mud, assuming that all of the analytical values of Na are derived from seawater, the chemical composition values of OF samples (the maximum Na content in all samples) listed in Supplementary Table S7 could be underestimated by about as much as 80% of the true value. However, the original sample also contains a considerable amount of seawater-derived components, so this underestimation is thought to be more limited.

### Data availability statement

All data generated or analysed during this study are included in this published article (and its Supplementary Information files).

## Electronic supplementary material

Supplementary information
