# Supplementary material for: The tremendous potential of deep-sea mud as a source of rare-earth elements
Source: Sci Rep. 2018 Apr 10;8:5763. doi: 10.1038/s41598-018-23948-5 (PMC5893572; doi:10.1038/s41598-018-23948-5)
Supplement: Supplementary file 1 — Supplementary information [file 41598_2018_23948_MOESM1_ESM.pdf]

The tremendous potential of deep-sea mud as a source of rare-earth elements

Authors:

Yutaro Takaya[1-4], Kazutaka Yasukawa[5,4], Takehiro Kawasaki[5], Koichiro Fujinaga[4,3], Junichiro Ohta[4,3,6], Yoichi Usui[7,2], Kentaro Nakamura[5], Jun-Ichi Kimura[6], Qing Chang[6], Morihisa Hamada[6], Gjergj Dodbiba[5], Tatsuo Nozaki[2-4,8], Koichi Iijima[2], Tomohiro Morisawa[9], Takuma Kuwahara[10], Yasuyuki Ishida[11], Takao Ichimura[11], Masaki Kitazume[12], Toyohisa Fujita[5], and Yasuhiro Kato[3,4,2*]

Affiliations:

[1] Department of Resources and Environmental Engineering School of Creative Science and Engineering, Waseda University, 3-4-1 Okubo, Shinjyuku, Tokyo 169-8555, Japan

[2] Research and Development Center for Submarine Resources, Japan Agency for Marine-Earth Science and Technology (JAMSTEC), 2-15 Natsushima, Yokosuka, Kanagawa 237-0061, Japan

[3] Frontier Research Center for Energy and Resources, School of Engineering, The University of Tokyo, 7-3-1 Hongo, Bunkyo-ku, Tokyo 113-8656, Japan

[4] Ocean Resources Research Center for Next Generation, Chiba Institute of Technology, 2-17-1 Tsudanuma, Narashino, Chiba 275-0016, Japan

[5] Department of Systems Innovation, School of Engineering, The University of Tokyo, 7-3-1 Hongo, Bunkyo, Tokyo 113-8656, Japan

[6] Department of Solid Earth Geochemistry, Japan Agency for Marine-Earth Science and Technology (JAMSTEC), 2-15 Natsushima, Yokosuka, Kanagawa 237-0061, Japan

[7] Department of Deep Earth Structure and Dynamics Research, Japan Agency for Marine-

Earth Science and Technology (JAMSTEC), 2-15 Natsushima, Yokosuka, Kanagawa 237-0061, Japan

[8] Department of Planetology, Graduate School of Science, Kobe University, 1-1 Rokkodai, Nada, Kobe, Hyogo 657-8501, Japan

[9] Engineering Project Department, Toa Corporation, 3-7-1 Nishi-Shinjuku, Shinjuku, Tokyo 163-1031, Japan

[10] Research and Development Center, Toa Corporation, 1-3 Anzen, Tsurumi, Yokohama, Kanagawa 230-0035, Japan

[11] Central Research Laboratory, Taiheiyo Cement Corporation, 2-4-2 Osaku, Sakura, Chiba 285-8655, Japan

[12] Department of Civil and Environmental Engineering, Tokyo Institute of Technology, 2-12-1 O-okayama, Meguro, Tokyo 152-8552, Japan

***corresponding author**

Yasuhiro Kato

Frontier Research Center for Energy and Resources, School of Engineering, The University of Tokyo, 7-3-1 Hongo, Bunkyo-ku, Tokyo 113-8656, Japan

Telephone: +81-3-5841-7022

E-mail: ykato@sys.t.u-tokyo.ac.jp

Figure and Table captions:

Fig. S1 (A) Particle size distribution of the over-flow (OF) and under-flow (UF) components recovered from hydrocyclone experiments. (B) Partition curves of the grain size separation experiments with hydrocyclone separator. The vertical axis shows the rate of under-flow component at each grain size fraction.

Fig. S2 Comparison of the actual submarine topographic map and a seabed topographic map reproduced by using ArcGIS with seafloor depth data of each coring point. The actual topographic map was created from the data obtained by the research cruises mentioned in the text, by using the Generic Mapping Tools software (https://www/soest.hawaii.edu/gmt/), Version 4.5.8 (ref. S1).

Table S1 Locations of sediment core samples used in the resource amount estimation.

Table S2 Whole rock chemical compositions of all sediment samples used in this study. The data of KR13-02 PC05 and PC06 are from Iijima et al. (ref. S2) and Fujinaga et al. (ref. S3), and are highlighted with yellow color.

Table S3 Average ΣREY and the total resource amount of REY-rich mud distributed from the seafloor to each target depth.

Table S4 Average concentration and resource amount of each interval for each grid (A1–D6). (A) lanthanum, (B) cerium, (C) praseodymium, (D) neodymium, (E) samarium, (F) europium, (G) gadolinium, (H) terbium, (I) dysprosium, (J) holmium, (K) erbium, (L) thulium, (M) ytterbium, (N) lutetium, and (O) yttrium.

Table S5 Chemical compositions of BCP and phillipsite grains in REY-rich mud determined by EPMA and LA-ICP-MS.

Table S6 The results of grain-size separation experiments by using test sieves for (A) “normally” REY-rich mud, (B) “highly” REY-rich mud, and (C) “extremely” REY-rich mud.

Table S7 The results of grain-size separation experiments with a hydrocyclone separator.

Supplementary References:

S1. Wessel, P. & Smith, W. H. F. New, improved version of Generic Mapping Tools released. *Eos Trans. Amer. Geophys. U.* 79, 579 (1998).

S2. Iijima, K. *et al*. Discovery of extremely REY-rich mud in the western North Pacific Ocean. *Geochem. J.*, **50**, 557–573 (2016).

S3. Fujinaga, K. *et al*. Geochemistry of REY-rich mud in the Japanese Exclusive Economic Zone around Minamitorishima Island. *Geochem. J.*, **50**, 575–590 (2016).

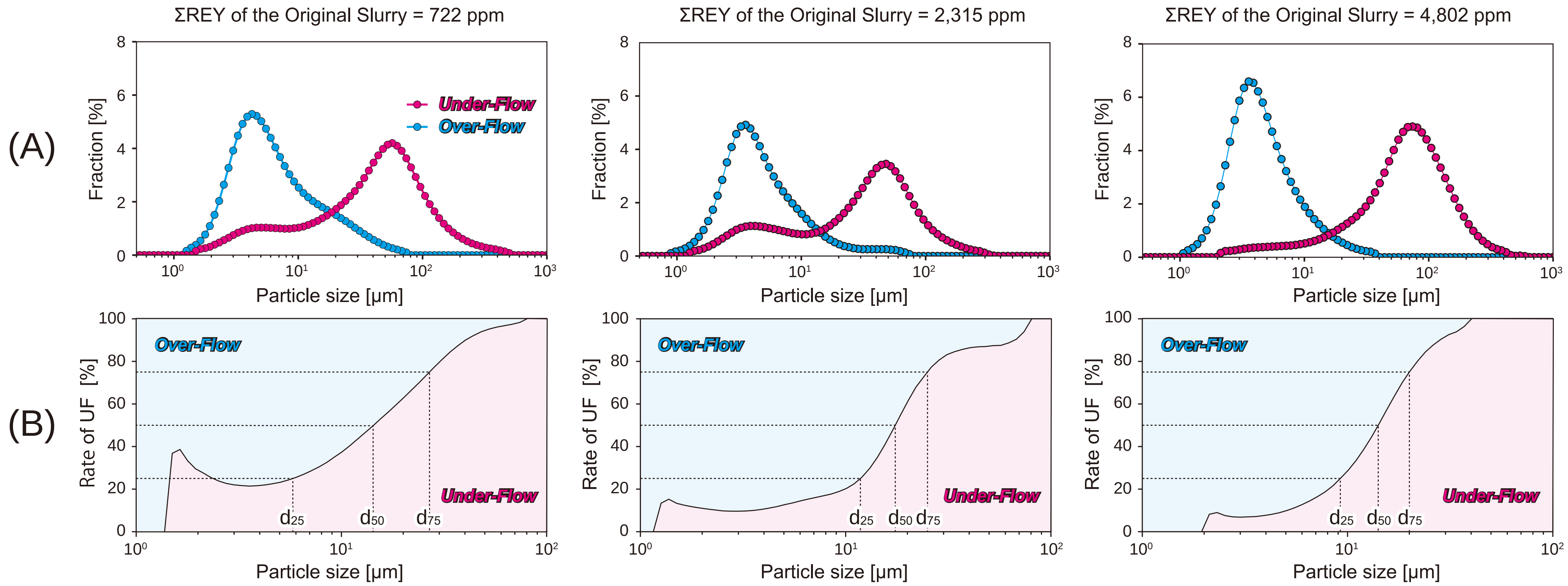
ΣREY of the Original Slurry = 722 ppm
ΣREY of the Original Slurry = 2,315 ppm
ΣREY of the Original Slurry = 4,802 ppm
(A)
(B)
Fraction [%]
Under-Flow
Over-Flow
Particle size [μm]
Rate of UF [%]
Over-Flow
Under-Flow
d25
d50
d75

Fig. S1

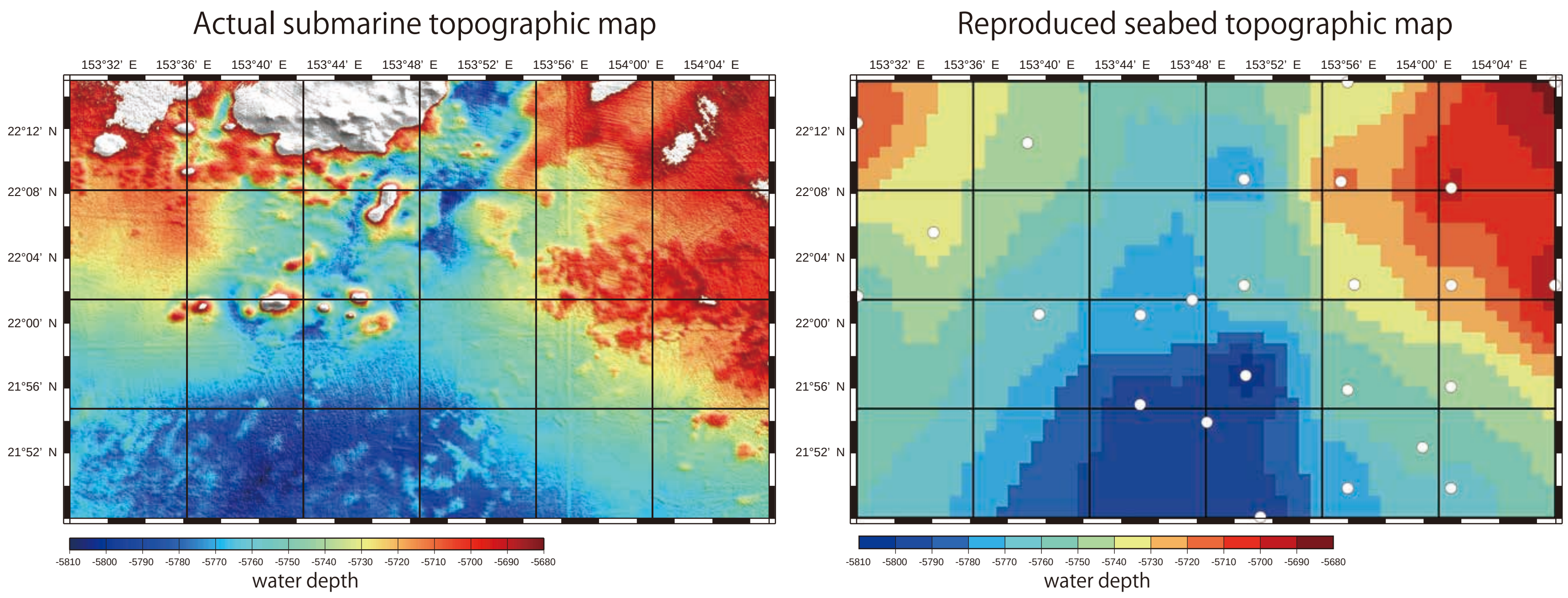

Actual submarine topographic map
153°32' E
153°36' E
153°40' E
153°44' E
153°48' E
153°52' E
153°56' E
154°00' E
154°04' E
22°12' N
22°08' N
22°04' N
22°00' N
21°56' N
21°52' N
-5810 -5800 -5790 -5780 -5770 -5760 -5750 -5740 -5730 -5720 -5710 -5700 -5690 -5680
water depth
Reproduced seabed topographic map
153°32' E
153°36' E
153°40' E
153°44' E
153°48' E
153°52' E
153°56' E
154°00' E
154°04' E
22°12' N
22°08' N
22°04' N
22°00' N
21°56' N
21°52' N
-5810 -5800 -5790 -5780 -5770 -5760 -5750 -5740 -5730 -5720 -5710 -5700 -5690 -5680
water depth

Fig. S2

Table S1

Cruise	Core No.	Latitude	Longitude	Water depth [m]
KR13-02	PC05	21°59.03'N	153°56.35E	5,735
KR13-02	PC06	21°51.01'N	153°59.98E	5,755
MR14-E02	PC01	21°59'N	154°07'E	5,694
MR14-E02	PC03	21°57.54'N	153°45'E	5,771
MR14-E02	PC04	22°09'N	153°56'E	5,740
MR14-E02	PC06	22°09'N	154°07'E	5,688
MR14-E02	PC08	21°49'N	153°56'E	5,775
MR14-E02	PC10	21°53.12'N	153°44.97'E	5,797
MR15-E01	PC01	21°59.0027'N	153°50.5056'E	5,759
MR15-E01	PC02	21°54.5504'N	153°50.5904'E	5,805
MR15-E01	PC03	21°47.5884'N	153°51.3625'E	5,795
MR15-E01	PC04	21°52.2491'N	153°48.5244'E	5,794
MR15-E01	PC05	21°53.8391'N	153°56.0060'E	5,756
MR15-E01	PC06	21°48.9981'N	154°01.4930'E	5,764
MR15-E01	PC07	21°53.9987'N	154°01.4834'E	5,748
MR15-E01	PC08	21°59.0065'N	154°01.5074'E	5,722
MR15-E01	PC09	22°03.7921'N	154°01.5021'E	5,699
MR15-E01	PC10	21°57.5755'N	153°39.6260'E	5,766
MR15-E01	PC11	21°58.2732'N	153°47.7461'E	5,770
MR15-E01	PC12	22°04.2191'N	153°50.5017'E	5,777
MR15-E01	PC13	22°04.1094'N	153°55.6448'E	5,719
MR15-02	PC01	22°01.6085'N	153°34.0013'E	5,738
MR15-02	PC09	22°06.0064'N	153°38.9968'E	5,740
MR15-02	PC10	22°07.0000'N	153°29.9631'E	5,710
MR15-02	PC11	21°58.4792'N	153°29.9763'E	5,754

Table S2 (1)

Cruise	Core	Sec.	Int. [cm]	Int. [cm]	Depth [cm]	Depth [m]	ΣREY [ppm]	Na (wt.%)	Mg	Al	P	K	Ca	Ti	V (ppm)	Cr	Mn (wt.%	Fe	Co (ppm)	Ni	Cu	Zn	Rb	Sr	Y	Zr	Nb	Mo	Cs	Ba	La	Ce	Pr	Nd	Sm	Eu	Gd	Tb	Dy	Ho	Er	Tm	Yb	Lu	Hf	Ta	Pb	Th	U
KR13-02	PC05	1	2	4	3	0.030	363	2.95	2.42	10.3	0.15	3.26	1.14	0.58	184	97.9	0.76	6.67	95.0	179	274	169	145	194	65.5	155	15.5	13.8	13.9	658	55.5	111	14.6	55.6	12.6	2.92	12.9	1.96	12.3	2.43	7.11	1.03	6.86	1.05	3.52	1.03	41.4	14.7	2.71
KR13-02	PC05	1	12	14	13	0.130	312	2.43	1.86	8.17	0.11	2.70	0.75	0.50	164	83.4	0.64	5.40	77.1	136	219	134	123	168	54.4	127	14.3	15.7	11.9	515	45.2	101	12.4	48.5	10.6	2.54	10.9	1.63	10.3	2.05	6.03	0.88	5.65	0.88	3.15	1.02	39.2	13.4	2.39
KR13-02	PC05	1	22	24	23	0.230	358	2.54	1.94	8.45	0.14	2.83	0.84	0.52	168	76.3	0.79	5.65	94.8	193	260	145	126	180	68.8	138	15.4	16.5	12.5	514	50.8	105	14.3	56.6	12.9	2.96	13.4	2.03	12.6	2.56	7.46	1.07	6.85	1.08	3.46	1.06	42.7	13.9	2.52
KR13-02	PC05	2	32	34	59.5	0.595	536	3.00	1.45	7.44	0.29	2.72	1.03	0.45	140	34.0	0.99	4.99	137	375	328	157	78.6	170	135	145	11.2	36.6	6.77	289	76.4	99.1	21.6	91.3	21.0	5.15	24.2	3.64	23.0	4.81	13.8	1.96	12.5	1.96	3.40	0.71	38.4	9.81	2.22
KR13-02	PC05	2	82	84	109.5	1.095	453	3.49	1.32	7.29	0.24	2.85	0.91	0.37	113	31.5	0.99	4.09	127	167	306	124	67.6	154	104	144	11.5	69.6	4.69	227	61.3	109	17.9	73.6	17.1	4.00	19.1	2.94	18.2	3.71	10.6	1.46	9.21	1.41	3.79	0.83	39.5	11.8	1.88
KR13-02	PC05	3	2	4	130	1.300	608	3.49	1.40	7.30	0.39	2.68	1.29	0.42	136	30.4	1.32	4.87	168	258	342	145	71.5	197	151	146	11.0	86.5	5.18	259	85.3	117	24.4	103	24.0	5.93	27.7	4.09	25.8	5.35	15.3	2.13	13.4	2.06	3.61	0.66	44.1	10.0	2.47
KR13-02	PC05	3	12	14	140	1.400	878	3.58	1.32	7.42	0.56	2.83	1.70	0.42	134	31.6	1.42	4.81	184	310	355	132	68.9	221	237	130	9.81	83.5	4.59	264	123	125	37.4	161	38.1	9.38	43.5	6.50	40.3	8.23	23.4	3.18	19.7	3.01	3.26	0.57	43.5	11.5	2.58
KR13-02	PC05	3	22	24	150	1.500	558	3.78	1.59	8.09	0.38	3.28	1.20	0.47	142	32.3	1.62	5.75	196	296	353	176	77.9	203	127	159	11.7	90.6	5.66	281	70.5	126	22.8	95.5	22.2	5.52	25.1	3.78	23.8	4.84	14.2	1.98	12.7	1.91	3.88	0.65	46.9	10.4	2.60
KR13-02	PC05	3	32	34	160	1.600	590	3.60	1.44	7.46	0.37	2.78	1.30	0.45	139	33.2	1.39	4.92	186	261	334	139	72.6	200	142	144	11.1	89.3	5.53	271	80.1	125	23.6	99.3	23.2	5.78	26.2	3.93	24.6	5.03	14.5	2.00	12.5	1.97	3.60	0.64	45.6	10.4	2.47
KR13-02	PC05	3	40	42	168	1.680	635	3.55	1.45	7.46	0.38	2.81	1.34	0.46	142	32.0	1.40	5.05	190	282	350	143	75.2	207	154	149	11.4	90.0	5.61	279	87.1	129	25.9	108	25.7	6.30	28.9	4.28	26.6	5.51	15.7	2.15	13.6	2.09	3.72	0.66	47.0	11.1	2.54
KR13-02	PC05	3	52	54	180	1.800	633	3.49	1.38	7.17	0.39	2.74	1.33	0.45	134	28.7	1.33	4.89	189	266	332	134	69.4	200	158	142	10.6	86.0	5.00	267	87.9	121	25.9	109	25.3	6.32	29.3	4.38	27.0	5.54	15.9	2.19	13.8	2.12	3.50	0.62	44.6	10.3	2.44
KR13-02	PC05	3	62	64	190	1.900	679	3.52	1.35	7.14	0.42	2.71	1.39	0.44	133	32.2	1.31	4.83	185	254	333	135	67.7	204	173	137	10.2	83.5	4.75	257	95.0	121	27.8	119	27.9	6.82	31.7	4.72	29.7	6.10	17.5	2.41	15.0	2.29	3.35	0.58	43.4	10.3	2.47
KR13-02	PC05	3	72	74	200	2.000	756	4.11	1.54	8.23	0.51	3.51	1.48	0.48	142	31.6	1.53	5.76	209	286	364	167	77.9	228	189	153	11.1	84.5	5.03	277	97.2	133	32.7	138	32.6	8.08	36.4	5.45	34.4	6.94	19.9	2.71	17.1	2.64	3.75	0.59	44.1	11.2	2.69
KR13-02	PC05	3	82	84	210	2.100	796	3.55	1.30	7.25	0.50	2.82	1.58	0.42	131	29.5	1.43	4.76	188	280	342	131	68.6	213	213	129	9.83	83.5	4.47	259	111	122	33.4	143	33.6	8.33	38.8	5.82	35.9	7.36	20.8	2.87	17.7	2.74	3.16	0.56	43.1	10.7	2.49
KR13-02	PC05	4	2	4	227	2.270	993	3.34	1.25	6.92	0.63	2.66	1.77	0.40	130	28.9	1.39	4.62	186	349	363	134	65.5	220	273	127	9.70	78.2	4.41	255	140	129	42.9	184	43.4	10.8	49.6	7.48	46.1	9.54	26.7	3.64	22.5	3.40	3.12	0.55	43.3	11.9	2.69
KR13-02	PC05	4	12	14	237	2.370	1206	3.27	1.24	7.05	0.77	2.69	2.06	0.40	130	27.9	1.41	4.66	189	276	328	129	67.0	240	343	133	10.2	78.3	4.60	257	173	137	52.4	227	53.8	13.2	61.2	9.12	56.7	11.6	33.1	4.49	27.5	4.21	3.18	0.58	45.6	13.6	3.00
KR13-02	PC05	4	22	24	247	2.470	1654	3.94	1.60	8.46	1.17	3.52	2.62	0.51	161	32.3	2.01	6.17	248	336	392	194	80.5	314	460	166	12.9	97.0	5.46	315	220	179	76.7	326	76.9	19.2	87.3	12.9	81.7	16.4	46.5	6.29	38.7	5.86	4.00	0.67	54.7	17.1	3.81
KR13-02	PC05	4	32	34	257	2.570	2331	3.14	1.28	6.66	1.48	2.41	3.48	0.45	138	29.8	1.67	4.92	226	355	366	143	58.8	317	683	150	12.0	85.8	3.81	249	337	194	105	457	109	27.1	125	18.6	115	23.6	65.9	8.89	54.2	8.27	3.60	0.66	50.3	19.6	4.39
KR13-02	PC05	4	42	44	267	2.670	2932	3.42	1.48	6.94	1.93	2.47	4.35	0.47	140	33.3	1.88	5.13	257	417	397	171	62.9	380	877	157	12.5	89.1	4.14	256	423	230	133	576	137	33.9	156	23.1	144	29.2	81.9	11.1	66.7	10.1	3.79	0.66	51.8	22.8	4.88
KR13-02	PC05	4	52	54	277	2.770	3815	3.21	1.49	6.81	2.58	2.47	5.61	0.45	134	35.9	2.07	4.85	282	496	405	180	64.7	453	1154	159	13.1	86.2	4.40	266	553	280	174	754	180	44.2	201	30.1	188	38.0	106	14.3	85.6	13.2	3.89	0.67	51.1	27.5	5.82
KR13-02	PC05	4	62	64	287	2.870	4402	2.95	1.39	6.43	2.94	2.42	6.36	0.41	116	34.3	1.63	4.38	220	406	360	165	62.3	487	1338	156	13.1	70.8	4.15	241	642	309	201	870	206	50.9	235	34.8	217	44.2	123	16.5	99.9	15.1	3.78	0.74	41.8	30.8	6.48
KR13-02	PC05	4	72	74	297	2.970	6607	2.69	1.29	5.95	4.66	2.77	8.67	0.37	90.9	29.6	1.41	3.78	172	386	309	183	58.8	651	2070	133	11.7	52.6	3.62	233	1011	491	291	1245	293	73.8	335	49.5	313	63.3	179	24.1	147	21.9	3.04	0.38	34.3	40.4	9.44
KR13-02	PC05	4	82	84	307	3.070	6677	2.56	1.21	5.30	4.82	2.25	10.1	0.33	83.7	29.8	1.12	3.40	142	327	286	162	55.0	673	2057	126	10.2	48.9	3.28	216	981	437	304	1317	313	77.0	354	52.5	328	67.1	188	25.3	153	23.0	2.79	0.21	32.0	42.4	10.3
KR13-02	PC05	4	92	94	317	3.170	5955	2.65	1.21	5.50	4.28	2.36	9.09	0.33	86.8	26.7	1.12	3.48	140	284	274	151	56.2	618	1823	143	10.3	50.1	3.19	227	878	394	272	1178	280	69.2	317	47.0	293	59.6	167	22.4	135	20.3	3.41	0.49	32.8	39.1	9.62
KR13-02	PC05	5	2	4	327	3.270	6106	2.92	1.29	5.79	4.44	2.51	9.41	0.34	94.5	28.0	1.20	3.73	144	297	278	160	59.8	642	1864	152	10.8	56.3	3.55	243	903	400	281	1213	290	71.5	325	48.1	299	61.0	170	22.8	137	20.6	3.62	0.63	36.2	40.4	10.2
KR13-02	PC05	5	12	14	337	3.370	5220	2.86	1.27	5.85	3.85	2.53	8.07	0.32	93.4	27.7	1.29	3.72	145	285	270	149	60.3	572	1580	143	10.3	61.4	3.53	249	778	345	242	1042	248	61.3	279	41.1	255	51.8	144	19.1	115	17.2	3.46	0.57	37.9	35.8	9.04
KR13-02	PC05	5	22	24	347	3.470	4269	2.86	1.35	6.23	3.18	3.00	5.97	0.32	102	30.6	1.64	3.96	172	399	283	169	68.7	488	1290	142	10.2	74.8	4.72	283	662	303	195	835	198	49.6	223	32.9	204	41.0	114	15.3	92.0	13.6	3.65	0.57	43.8	33.8	8.14
KR13-02	PC05	5	32	34	357	3.570	3055	2.89	1.70	7.67	2.12	3.29	4.82	0.38	135	38.9	1.71	4.79	196	450	311	164	106	396	908	166	12.0	89.4	8.33	359	459	271	138	588	143	34.9	157	23.1	141	28.7	79.9	10.6	63.2	9.54	4.06	0.71	54.3	28.9	6.25
KR13-02	PC05	5	42	44	367	3.670	1934	2.64	1.73	7.62	1.33	3.18	3.08	0.36	132	43.0	1.52	4.61	182	368	281	160	112	282	533	154	11.4	82.4	9.42	356	275	220	87.5	377	91.6	22.2	101	14.9	90.2	18.2	50.3	6.75	40.6	6.09	3.90	0.73	54.5	23.8	4.74
KR13-02	PC05	5	52	54	377	3.770	1533	2.60	1.81	8.01	1.02	3.29	2.44	0.37	139	42.8	1.50	4.72	185	371	275	167	124	243	409	155	11.8	80.8	10.5	378	214	210	68.2	291	70.6	17.3	77.4	11.5	69.8	14.0	39.0	5.26	31.7	4.77	4.06	0.78	56.0	22.5	4.14
KR13-02	PC05	5	62	64	387	3.870	1364	2.65	1.88	8.41	0.90	3.47	2.17	0.39	146	42.0	1.49	4.92	186	369	269	170	133	231	356	159	12.1	81.0	11.4	397	187	206	60.4	256	61.8	15.2	67.3	10.0	61.1	12.3	34.1	4.59	27.9	4.22	4.02	0.80	57.4	22.1	3.91
KR13-02	PC05	5	72	74	397	3.970	952	2.42	1.83	8.17	0.66	3.62	1.40	0.36	133	40.6	1.46	4.68	175	347	240	188	125	185	222	144	11.5	69.8	10.9	371	121	178	43.1	180	43.1	10.6	46.5	6.89	42.6	8.38	23.7	3.20	19.9	2.93	3.80	0.73	52.8	18.7	3.20
KR13-02	PC05	5	82	84	407	4.070	1024	2.59	1.78	7.87	0.63	3.20	1.57	0.36	135	39.3	1.41	4.63	175	418	252	164	123	189	256	143	11.3	74.7	10.9	356	140	180	44.4	187	44.7	11.0	48.8	7.22	44.2	8.90	24.8	3.38	20.5	3.07	3.76	0.75	52.3	18.0	3.08
KR13-02	PC05	5	92	94	417	4.170	950	2.54	1.76	7.83	0.57	3.17	1.46	0.35	130	36.7	1.37	4.52	166	348	243	161	119	182	236	142	11.3	73.7	10.5	359	130	168	41.3	175	42.0	10.1	45.3	6.71	40.7	8.19	22.6	3.04	18.5	2.78	3.67	0.73	51.7	16.5	2.82
KR13-02	PC05	6	2	4	426.5	4.265	1718	2.49	1.69	7.62	1.18	3.11	2.73	0.36	128	38.5	1.47	4.51	176	377	250	165	113	258	459	146	11.6	74.6	9.86	355	244	222	77.4	330	79.5	19.4	86.7	12.9	79.2	15.9	44.3	5.96	36.1	5.46	3.75	0.75	52.6	21.3	4.03
KR13-02	PC05	6	12	14	436.5	4.365	936	2.54	1.75	7.87	0.55	3.19	1.42	0.36	135	39.0	1.46	4.62	166	379	239	160	121	184	228	144	11.5	75.4	11.0	376	128	177	40.4	169	40.9	9.90	43.6	6.50	39.6	7.93	22.0	2.95	18.0	2.69	3.74	0.74	53.2	16.9	2.77
KR13-02	PC05	6	22	24	446.5	4.465	864	2.62	1.87	8.41	0.54	3.70	1.20	0.36	135	40.6	1.67	4.85	181	375	237	199	127	183	194	145	11.7	75.7	11.6	396	110	180	38.1	159	37.9	9.43	40.6	6.11	37.4	7.43	20.9	2.81	17.3	2.56	3.91	0.74	57.0	17.3	2.86
KR13-02	PC05	6	32	34	457	4.570	955	2.64	1.83	8.21	0.56	3.31	1.43	0.37	142	44.5	1.61	4.81	182	377	244	167	124	189	231	151	12.1	80.1	11.3	396	130	184	41.2	171	41.4	9.95	43.9	6.58	40.1	7.93	22.3	3.02	18.4	2.73	3.93	0.78	59.2	17.0	2.81
KR13-02	PC05	6	42	44	466.5	4.665	1051	2.74	1.89	8.62	0.62	3.46	1.59	0.39	145	41.3	1.63	5.00	185	417	251	173	129	207	259	155	12.3	82.6	11.6	406	144	192	45.7	192	46.3	11.2	49.0	7.34	44.8	8.89	24.8	3.34	20.4	3.05	4.06	0.79	60.1	18.2	3.04
KR13-02	PC05	6	52	54	476.5	4.765	1143	2.70	1.81	8.16	0.70	3.31	1.75	0.38	139	38.6	1.63	4.80	181	442	249	171	122	211	288	149	11.7	79.7	10.9	387	158	192	50.1	211	51.0	12.4	54.8	8.19	49.7	9.97	27.7	3.75	22.9	3.44	3.91	0.77	58.4	18.4	3.14
KR13-02	PC05	6	62	64	486.5	4.865	991	2.62	1.77	8.00	0.57	3.23	1.47	0.37	138	41.5	1.60	4.73	181	373	239	165	118	196	241	148	11.5	80.4	10.7	387	136	184	43.1	180	43.1	10.6	46.5	6.98	42.2	8.46	23.5	3.20	19.3	2.89	3.91	0.76	58.6	17.4	2.83
KR13-02	PC05	6	72	74	496.5	4.965	926	2.64	1.84	8.26	0.58	3.66	1.28	0.37	136	37.9	1.78	4.85	190	390	240	196	121	196	211	145	11.7	77.0	10.7	399	117	183	41.7	174	41.6	10.2	44.6	6.69	40.9	8.03	22.5	3.03	18.7	2.73	3.91	0.75	58.6	17.4	2.79
KR13-02	PC05	6	82	84	506.5	5.065	1019	2.71	1.81	8.11	0.58	3.29	1.50	0.37	138	39.3	1.60	4.72	178	382	241	166	122	199	250	151	11.8	77.7	10.8	394	139	185	44.5	186	45.2	10.9	48.1	7.20	43.6	8.68	24.0	3.25	19.9	2.95	3.92	0.77	58.9	17.5	2.80
KR13-02	PC05	6	92	94	516.5	5.165	1118	2.57	1.71	7.65	0.65	3.12	1.62	0.36	134	38.1	1.52	4.59	170	358	229	158	115	207	280	143	11.3	72.6	10.2	372	153	187	49.7	207	49.9	12.3	54.6	8.08	49.1	9.81	27.1	3.68	22.5	3.34	3.78	0.75	57.1	17.9	2.95
KR13-02	PC05	7	2	4	526.5	5.265	963	2.68	1.80	8.10	0.54	3.29	1.39	0.37	138	40.6	1.58	4.76	177	370	231	164	123	193	233	149	11.5	74.3	10.9	390	131	182	41.9	175	42.5	10.2	45.1	6.73	40.7	8.11	22.6	3.05	18.6	2.77	3.85	0.74	58.5	17.0	2.73
KR13-02	PC05	7	12	14	536.5	5.365	788	2.55	1.76	7.85	0.41	3.17	1.12	0.35	136	42.6	1.63	4.71	175	384	231	162	121	177	182	141	10.9	74.3	10.9	382	105	169	33.5	139	33.6	8.08	35.5	5.32	32.2	6.42	17.9	2.41	14.8	2.20	3.73	0.72	59.6	15.4	2.45
KR13-02	PC05	7	22	24	546.5	5.465	803	2.67	1.91	8.63	0.49	3.79	1.12	0.36	138	40.6	1.85	5.04	193	407	243	207	128	189	176	144	11.5	74.8	11.3	415	99.7	177	35.3	148	35.3	8.55	37.3	5.56	33.9	6.71	18.9	2.54	15.7	2.29	3.83	0.73	60.6	16.1	2.56
KR13-02	PC05	7	32	34	557	5.570	835	2.60	1.76	7.93	0.45	3.22	1.22	0.35	133	43.5	1.55	4.63	176	382	230	162	121	187	196	145	11.1	73.0	10.6	398	113	171	35.5	149	36.2	8.72	38.0	5.74	34.7	6.90	19.5	2.63	16.0	2.38	3.80	0.74	58.9	15.8	2.52
KR13-02	PC05	7	42	44	566.5	5.665	950	2.76	1.83	8.32	0.55	3.39	1.46	0.38	141	44.8	1.65	4.96	188	389	245	167	126	214	230	155	11.7	74.8	10.9	413	129	183	40.6	170	41.0	9.92	43.7	6.53	39.9	8.03	22.7	3.06	18.9	2.84	4.14	0.77	60.5	16.3	2.79
KR13-02	PC05	7	52	54	577	5.770	724	2.66	1.78	8.19	0.39	3.30	1.09	0.35	135	47.6	1.59	4.75	178	372	236	162	123	187	166	149	11.2	72.4	10.8	412	96.6	166	29.6	123	30.0	7.15	31.4	4.69	28.8	5.83	16.5	2.25	13.8	2.09	3.95	0.73	57.7	14.3	2.35
KR13-02	PC05	7	62	64	587	5.870	739	2.74	1.81	8.18	0.41	3.39	1.14	0.36	135	50.2	1.57	4.76	174	365	236	164	122	210	172	153	11.2	70.1	10.3	435	98.9	165	30.3	126	30.7	7.37	32.4	4.85	29.5	5.98	17.0	2.30	14.3	2.15	4.19	0.75	58.3	14.6	2.38
KR13-02	PC05	7	72	74	596.5	5.965	700	2.77	1.91	8.62	0.42	4.04	1.02	0.36	131	57.8	1.75	4.95	175	378	241	196	125	220	154	147	11.5	66.0	10.3	448	87.4	160	30.2	125	29.7	7.19	31.8	4.73	29.3	5.83	16.6	2.25	14.1	2.07	4.07	0.73	59.6	14.8	2.39
KR13-02	PC05	7	82	84	606.5	6.065	795	2.81	1.91	8.83	0.43	3.58	1.18	0.38	140	44.4	1.62	5.09	174	392	248	174	133	207	187	159	12.0	70.4	11.6	431	107	169	33.0	139	33.4	8.01	35.2	5.27	32.5	6.57	18.6	2.52	15.3	2.31	4.25	0.81	61.0	15.5	2.54
KR13-02	PC05	7	92	94	617	6.170	936	2.83	1.87	8.61	0.50	3.48	1.36	0.37	140	42.5	1.63	5.00	177	404	256	175	128	203	227	156	12.0	70.0	11.2	420	127	177	40.2	170	41.0	10.0	43.3	6.55	40.1	8.06	22.7	3.04	18.4	2.73	4.15	0.81	61.4	16.4	2.65
KR13-02	PC05	8	2	4	627	6.270	1082	2.78	1.87	8.54	0.58	3.46	1.48	0.38	138	42.0	1.70	4.94	179	443	268	176	127	210	267	156	12.1	70.4	11.2	414	147	187	47.8	202	49.1	12.1	52.3	7.82	47.2	9.45	26.0	3.50	21.0	3.12	4.18	0.81	60.6	17.5	2.77
KR13-02	PC05	8	12	14	637	6.370	1186	2.84	1.91	8.79	0.63	3.57	1.61	0.39	147	39.3	1.66	5.14	194	437	267	171	130	218	294	163	12.5	79.5	11.6	434	161	197	52.7	224	54.5	13.3	57.5	8.72	52.7	10.5	28.9	3.92	23.2	3.44	4.31	0.85	61.8	18.4	2.97
KR13-02	PC05	8	22	24	646.5	6.465	1200	2.60	1.83	8.20	0.73	3.61	1.56	0.35	127	36.0	1.99	4.70	211	489	276	209	117	217	285	143	11.6	69.5	9.95	402	152	192	57.0	239	58.4	14.4	62.1	9.29	56.3	11.0	30.4	4.06	24.4	3.55	3.91	0.71	58.3	17.3	2.81
KR13-02	PC05	8	32	34	657	6.570	1668	2.60	1.76	7.78	0.98	3.18	2.33	0.36	133	37.7	1.94	4.59	222	543	306	182	111	249	440	148	11.7	75.0	9.57	402	232	221	75.5	323	78.6	19.5	85.3	12.9	77.7	15.5	42.7	5.70	33.7	4.89	3.92	0.75	61.7	19.0	3.39
KR13-02	PC05	8	42	44	667	6.670	2092	2.33	1.62	7.14	1.32	2.95	3.00	0.35	121	35.5	1.83	4.25	190	539	303	172	99.5	274	564	143	11.2	67.5	8.33	371	297	232	97.1	418	101	25.3	111	16.7	101	20.0	54.2	7.16	42.3	6.15	3.64	0.74	58.6	21.1	3.82
KR13-02	PC05	8	52	54	677	6.770	2485	2.49	1.64	6.99	1.85	3.16	4.12	0.34	116	32.3	1.66	4.05	192	520	322	172	93.0	329	705	150	11.1	62.8	7.45	363	361	244	113	484	116	29.0	129	19.4	120	24.3	67.4	9.09	54.6	8.12	3.88	0.75	55.2	19.8	4.76
KR13-02	PC05	8	62	64	687	6.870	3001	2.45	1.58	6.66	2.61	3.21	5.72	0.32	105	29.6	1.49	3.63	190	492	350	169	85.5	406	902	154	10.9	60.4	6.21	337	452	254	134	571	133	33.0	151	22.8	142	29.6	84.2	11.6	71.1	10.7	3.97	0.69	44.8	16.6	5.90
KR13-02	PC05	8	72	74	696.5	6.965	2230	2.50	1.71	6.93	2.36	3.59	4.51	0.38	102	32.0	1.56	3.84	187	533	363	207	87.1	363	630	168	13.5	57.3	5.94	344	318	216	104	436	101	25.0	114	16.9	108	22.1	64.1	8.81	55.5	8.34	4.25	0.82	40.0	13.7	5.08
KR13-02	PC05	8	82	84	706.5	7.065	2156	2.39	1.66	6.82	2.08	3.26	4.63	0.36	107	31.2	1.34	3.78	172	482	359	175	86.0	340	633	167	12.5	57.7	6.41	338	332	208	96.3	405	93.4	23.0	106	15.8	99.6	20.7	59.0	8.12	49.9	7.64	4.24	0.80	40.5	13.0	4.89
KR13-02	PC05	8	92	94	717	7.170	1983	2.43	1.69	7.05	1.98	3.43	4.46	0.33	108	26.9	1.46	3.74	182	502	376	177	92.1	335	577	165	10.8	59.3	6.61	344	304	207	87.4	368	84.1	20.9	95.3	14.3	90.7	19.0	54.0	7.53	46.9	7.11	4.20	0.70	41.4	12.5	4.76
KR13-02	PC05	9	2	4	726	7.260	1766	2.31	1.65	6.81	1.80	3.29	4.06	0.32	104	27.1	1.38	3.59	173	520	375	176	89.8	311	515	159	10.4	56.0	6.26	330	267	195	76.9	323	74.9	18.4	84.0	12.6	79.9	16.8	48.1	6.68	41.5	6.32	4.01	0.68	38.8	11.9	4.25
KR13-02	PC05	9	12	14	736	7.360	1700	2.33	1.64	6.67	1.70	3.25	3.83	0.31	97.1	25.4	1.33	3.49	163	424	354	182	87.1	297	487	154	10.1	49.5	6.03	319	260	192	74.7	313	72.2	17.8	81.5	12.2	76.8	16.0	45.6	6.27	38.8	5.85	3.85	0.66	36.9	11.3	3.95
KR13-02	PC05	9	22	24	745.5	7.455	1568	2.53	1.77	7.09	1.75	3.86	3.47	0.32	97.0	25.3	1.64	3.57	188	474	370	227	90.4	301	416	149	10.0	52.6	5.90	347	222	196	73.4	303	70.2	17.3	78.6	11.7	73.7	14.9	42.9	5.88	36.9	5.47	3.87	0.64	39.3	11.2	3.82
KR13-02	PC05	9	32	34	756	7.560	1511	2.59	1.94	6.61	1.67	2.89	3.75	0.28	89.2	20.0	1.63	3.29	205	330	509	292	77.9	285	433	146	8.68	47.9	5.39	360	224	201	64.0	266	61.4	15.2	69.2	10.4	66.2	13.9	40.4	5.71	36.1	5.52	3.60	0.57	38.6	9.93	3.63
KR13-02	PC05	9	42	44	766	7.660	1560	2.35	1.70	6.65	1.63	3.13	3.72	0.29	97.3	25.4	1.50	3.47	176	397	424	210	85.4	285	441	147	9.18	53.7	6.06	350	236	200	66.0	280	64.5	15.7	72.3	10.9	69.2	14.5	41.9	5.84	36.4	5.59	3.67	0.60	41.6	10.7	3.67
KR13-02	PC05	9	52	54	776	7.760	1525	2.44	1.70	6.81	1.39	3.33	3.19	0.31	101	26.3	1.36	3.61	166	412	368	176	88.6	274	416	150	9.71	50.3	6.17	340	230	193	68.5	286	66.4	16.2	73.4	11.0	68.3	13.9	38.9	5.30	32.2	4.79	3.80	0.64	40.6	10.6	3.40
KR13-02	PC05	9	62	64	786	7.860	1493	2.49	1.63	6.90	1.42	3.46	3.29	0.30	101	24.3	1.40	3.55	168	449	375	165	86.0	285	416	148	9.20	53.0	5.92	340	224	190	65.4	274	63.4	15.7	70.6	10.6	66.6	13.8	39.1	5.35	32.8	4.90	3.76	0.61	39.8	10.1	3.32
KR13-02	PC05	9	72	74	795.5	7.955	1329	2.66	1.67	7.09	1.50	4.03	3.07	0.30	97.4	21.7	1.55	3.55	169	471	403	198	84.7	295	360	141	8.55	53.2	5.57	348	185	184	58.8	244	56.9	14.0	63.8	9.58	61.5	12.7	37.1	5.17	32.8	4.86	3.65	0.54	39.6	9.08	3.24
KR13-02	PC05	9	82	84	805.5	8.055	1484	2.63	1.62	7.03	1.45	3.60	3.38	0.30	104	23.3	1.44	3.63	156	467	377	159	86.8	306	415	145	8.71	55.2	5.91	341	221	194	64.5	269	63.1	15.5	69.8	10.4	66.1	13.6	38.8	5.34	32.9	4.94	3.77	0.58	43.5	9.67	3.37
KR13-02	PC05	9	92	94	816	8.160	1680	2.51	1.63	6.84	1.69	3.41	3.82	0.30	94.0	22.8	1.16	3.46	135	365	330	173	86.5	313	483	146	9.32	38.4	6.04	314	252	200	73.2	304	70.6	17.4	79.5	12.0	76.0	15.8	45.1	6.21	38.5	5.85	3.71	0.62	34.9	12.2	3.76
KR13-02	PC05	10	22	24	845.5	8.455	919	2.46	1.92	7.50	0.87	3.78	1.79	0.32	90.9	24.7	1.14	3.83	126	268	320	236	98.0	196	227	140	10.5	27.4	6.61	334	124	160	40.7	169	39.7	9.57	43.0	6.44	40.5	8.20	23.8	3.29	20.9	3.11	3.75	0.69	32.3	11.4	2.36
KR13-02	PC05	10	32	34	856	8.560	989	2.47	1.79	7.37	0.81	3.40	1.97	0.32	97.8	28.4	1.07	3.85	127	282	312	193	98.6	206	257	142	10.3	30.2	6.89	331	142	170	41.6	174	40.5	9.75	44.3	6.67	41.7	8.59	24.6	3.44	21.4	3.26	3.81	0.70	34.5	11.3	2.37
KR13-02	PC05	10	72	74	895.5	8.955	898	2.58	1.88	7.68	0.63	3.79	1.38	0.33	96.1	25.1	1.04	4.03	108	280	278	204	96.9	185	213	139	10.8	28.4	7.12	310	122	159	41.1	170	39.7	9.55	42.6	6.39	39.4	7.90	22.6	3.09	19.4	2.82	3.83	0.70	33.1	10.6	2.04
KR13-02	PC05	10	82	84	905.5	9.055	976	2.54	1.82	7.49	0.57	3.32	1.44	0.34	101	26.6	1.00	3.96	110	285	286	182	101	187	240	144	10.9	31.0	7.28	318	142	170	43.0	180	41.8	10.0	44.3	6.66	41.2	8.22	23.2	3.14	19.2	2.85	3.94	0.73	35.3	10.6	2.01
KR13-02	PC05	11	22	24	945	9.450	1403	2.90	2.13	8.87	0.77	3.90	1.96	0.39	146	46.0	1.19	4.64	131	442	404	194	118	224	350	151	12.7	36.2	8.01	360	218	193	66.6	279	64.4	15.4	68.4	10.1	60.7	11.8	31.9	4.19	24.7	3.53	3.85	0.75	39.3	14.5	2.21
KR13-02	PC05	11	32	34	955	9.550	1013	2.50	1.87	7.71	0.47	3.52	1.22	0.37	107	30.4	1.02	4.11	113	304	285	184	107	182	236	152	12.0	29.3	7.40	348	149	170	47.7	199	46.6	11.0	48.8	7.28	43.9	8.51	23.0	2.99	17.9	2.58	4.22	0.80	36.9	12.8	1.93
KR13-02	PC05	11	72	74	995	9.950	1052	2.57	1.90	7.58	0.63	3.97	1.41	0.34	112	35.5	1.72	4.25	178	564	345	209	94.0	207	262	139	10.8	51.4	6.28	395	147	173	46.5	194	45.4	11.0	50.3	7.61	48.0	9.73	27.8	3.77	23.2	3.36	3.74	0.69	44.3	11.5	2.01
KR13-02	PC05	11	82	84	1005	10.050	1055	2.60	1.79	7.68	0.56	3.77	1.44	0.34	104	29.2	1.43	3.85	154	375	324	194	100	220	267	148	11.5	40.1	6.28	382	155	174	45.8	193	45.2	10.7	48.9	7.36	45.4	9.22	25.9	3.54	21.4	3.19	4.03	0.76	40.5	12.3	2.02
KR13-02	PC06	1	2	4	3	0.030	276	2.81	1.93	8.07	0.10	2.72	0.89	0.48	157	83.9	0.54	5.25	83.5	135	215	129	122	172	46.3	123	14.0	8.77	11.5	619	40.2	93.0	11.4	40.1	9.85	2.22	9.56	1.44	8.92	1.77	5.24	0.74	4.89	0.75	3.09	0.98	35.0	13.3	2.33
KR13-02	PC06	1	52	54	53	0.530	449	2.77	1.90	8.47	0.21	2.87	0.89	0.52	167	69.5	0.86	5.96	127	282	326	157	119	191	106	151	15.6	22.6	12.2	424	65.0	97.3	18.9	70.3	17.6	4.37	19.3	2.97	18.7	3.83	11.2	1.57	10.2	1.58	3.66	0.98	44.7	13.1	2.52
KR13-02	PC06	2	42	44	100.5	1.005	553	3.71	1.56	8.26	0.32	3.16	1.07	0.48	145	40.1	1.04	5.40	169	329	359	153	85.0	186	143	153	12.1	63.0	6.42	275	79.4	105	22.9	85.7	22.1	5.44	25.1	3.83	24.2	4.97	14.5	2.04	13.0	2.01	3.73	0.71	41.8	10.5	2.44
KR13-02	PC06	2	92	94	150.5	1.505	546	3.95	1.53	7.86	0.37	3.01	1.23	0.49	147	34.9	1.39	5.48	204	300	367	150	79.9	208	136	159	12.1	93.7	5.79	286	76.9	117	21.9	83.0	21.4	5.28	23.8	3.63	22.9	4.68	13.7	1.90	12.2	1.88	3.92	0.67	47.1	10.1	2.66
KR13-02	PC06	3	42	44	200	2.000	790	3.99	1.47	8.14	0.51	3.25	1.60	0.48	150	34.6	1.73	5.58	267	329	378	149	78.7	237	212	151	11.5	98.6	5.01	310	112	129	34.0	129	33.8	8.40	38.0	5.73	36.0	7.30	21.0	2.87	18.1	2.77	3.71	0.59	52.2	11.4	2.81
KR13-02	PC06	3	92	94	250	2.500	2065	3.53	1.46	7.08	1.36	2.78	3.13	0.48	160	34.2	2.81	5.51	467	729	464	191	70.4	336	622	153	12.8	122	4.84	346	314	215	91.1	352	93.5	23.2	105	15.7	98.1	19.6	55.4	7.42	45.7	6.86	3.84	0.64	72.7	18.0	4.33
KR13-02	PC06	4	42	44	300	3.000	772	3.21	1.79	7.89	0.53	3.26	1.29	0.34	129	37.0	1.58	4.64	219	497	271	174	116	184	185	139	11.5	96.3	10.00	395	106	162	33.7	127	33.6	8.20	35.2	5.31	32.5	6.37	18.0	2.44	15.0	2.21	3.72	0.68	53.6	13.3	2.72
KR13-02	PC06	4	92	94	350	3.500	805	3.32	1.85	8.00	0.57	3.28	1.37	0.35	135	38.7	1.79	4.70	238	586	278	177	118	189	193	140	11.7	100	10.1	406	111	168	35.2	132	35.0	8.52	37.1	5.55	34.1	6.70	18.9	2.53	15.5	2.28	3.76	0.68	56.6	13.3	2.89
KR13-02	PC06	5	42	44	399.5	3.995	870	3.09	1.80	7.90	0.61	3.22	1.45	0.34	134	39.8	1.89	4.62	262	514	272	184	115	196	207	140	11.7	104	9.75	419	119	180	38.2	143	38.1	9.33	40.6	6.06	37.2	7.32	20.7	2.79	17.1	2.53	3.83	0.68	57.8	13.6	2.95
KR13-02	PC06	5	92	94	449.5	4.495	731	2.89	1.81	8.12	0.48	3.37	1.21	0.34	140	39.8	2.04	4.79	278	524	278	190	118	189	167	140	11.3	105	9.90	443	96.8	183	29.8	112	29.4	7.23	31.3	4.72	29.2	5.79	16.5	2.25	13.8	2.06	3.71	0.68	59.3	12.4	2.51
KR13-02	PC06	6	42	44	499	4.990	977	2.80	1.85	8.05	0.66	3.49	1.56	0.38	135	43.2	1.85	4.78	235	540	286	192	122	209	242	139	11.7	90.8	9.89	434	134	188	43.0	163	43.5	10.8	46.3	6.94	42.6	8.35	23.4	3.14	19.1	2.82	3.71	0.73	61.0	14.0	2.88
KR13-02	PC06	6	92	94	549	5.490	949	2.84	1.95	8.51	0.64	3.70	1.53	0.41	148	43.8	1.96	5.23	218	548	291	199	136	215	238	151	12.8	91.1	11.0	473	131	188	40.9	154	40.5	10.1	43.6	6.58	40.6	8.02	22.6	3.04	18.6	2.80	3.90	0.81	69.2	14.5	2.84
KR13-02	PC06	7	42	44	599	5.990	1093	2.55	1.87	8.32	0.69	3.63	1.61	0.41	137	41.4	1.55	4.87	168	447	251	183	139	207	283	142	12.0	69.8	11.4	435	151	188	48.9	186	49.8	12.3	52.6	7.87	48.4	9.50	26.7	3.56	21.7	3.25	3.57	0.78	59.0	16.4	2.92
KR13-02	PC06	7	92	94	649	6.490	2710	2.59	1.59	7.42	1.71	3.18	3.63	0.38	122	34.6	1.90	4.34	214	630	335	192	99.8	336	799	135	11.4	76.3	7.73	419	413	269	124	480	130	32.5	143	21.2	130	25.4	70.9	9.37	56.4	8.24	3.77	0.71	70.9	25.1	4.59
KR13-02	PC06	8	42	44	699	6.990	1349	2.63	1.87	7.56	1.02	3.58	2.21	0.36	109	40.4	1.21	4.33	159	470	334	189	101	229	360	144	12.0	46.2	6.57	360	215	188	60.8	231	60.1	14.7	66.2	9.77	60.4	12.0	34.2	4.63	28.7	4.20	4.03	0.75	41.3	14.8	3.10
KR13-02	PC06	8	92	94	749	7.490	721	2.46	1.76	7.26	0.57	3.52	1.29	0.30	88.4	26.0	1.11	3.37	147	290	388	213	90.7	167	173	127	10.6	33.2	5.36	334	107	132	33.0	123	31.8	7.59	34.4	5.12	31.2	6.16	17.4	2.37	14.7	2.18	3.44	0.69	31.1	10.7	2.06
KR13-02	PC06	9	42	44	799	7.990	664	2.43	1.99	7.92	0.50	3.67	1.16	0.36	92.3	34.2	0.89	3.74	110	172	302	215	110	155	164	132	12.9	18.8	6.36	326	98.2	133	28.3	105	26.7	6.34	29.2	4.38	27.8	5.58	16.5	2.30	14.7	2.18	3.51	0.88	29.2	12.1	2.17
KR13-02	PC06	9	92	94	849	8.490	600	2.45	1.92	8.41	0.37	4.09	0.89	0.40	103	34.2	0.60	4.11	81.2	281	247	164	126	149	134	138	14.3	15.9	7.48	305	89.0	130	27.2	101	25.3	5.93	26.5	3.92	24.0	4.69	13.3	1.80	11.2	1.67	3.75	0.97	25.1	13.6	2.05
KR13-02	PC06	10	42	44	899.5	8.995	666	2.48	1.89	8.43	0.43	4.30	1.03	0.42	109	36.1	0.65	4.27	85.1	295	246	162	130	164	160	145	15.3	21.0	7.61	336	102	130	29.6	111	27.6	6.49	29.3	4.38	27.2	5.40	15.5	2.13	13.4	1.99	3.85	1.00	25.6	13.8	2.16
KR13-02	PC06	10	92	94	949.5	9.495	536	2.32	1.78	7.80	0.29	3.94	0.74	0.38	96.3	31.8	0.59	3.89	78.4	300	240	148	119	143	120	131	13.5	18.2	7.25	301	80.5	118	24.1	90.3	22.0	5.19	23.2	3.44	21.2	4.13	11.5	1.57	9.66	1.41	3.51	0.89	23.6	12.4	1.77
KR13-02	PC06	11	42	44	998.5	9.985	503	2.24	1.71	7.66	0.27	3.89	0.68	0.36	91.5	32.4	0.52	3.80	66.1	182	198	146	118	135	112	125	13.2	11.7	7.13	301	75.6	114	22.1	83.8	20.3	4.73	21.2	3.16	19.6	3.85	10.8	1.49	9.14	1.36	3.35	0.88	21.6	12.2	1.71
KR13-02	PC06	11	92	94	1048.5	10.485	511	2.28	1.79	7.82	0.29	3.92	0.74	0.37	89.6	34.9	0.54	3.86	59.3	159	206	154	117	143	118	129	13.4	9.13	7.16	306	77.2	111	22.1	85.3	20.5	4.77	21.4	3.18	19.7	3.94	11.2	1.56	9.79	1.45	3.42	0.87	24.4	12.3	1.73

Table S2 (2: continued)

Cruise	Core	Sec.	Int. [cm]	Int. [cm]	Depth [cm]	Depth [m]	ΣREY [ppm]	Na (wt.%)	Mg	Al	P	K	Ca	Ti	V (ppm)	Cr	Mn (wt.%	Fe	Co (ppm)	Ni	Cu	Zn	Rb	Sr	Y	Zr	Nb	Mo	Cs	Ba	La	Ce	Pr	Nd	Sm	Eu	Gd	Tb	Dy	Ho	Er	Tm	Yb	Lu	Hf	Ta	Pb	Th	U
MR14-E02	PC01	1	4	6	5	0.05	287	3.00	2.25	9.35	0.09	2.93	0.82	0.49	144	93.2	0.57	5.88	67.2	139	207	245	137	158	41.0	105	14.6	13.6	13.3	829	39.6	103	12.0	45.6	10.0	2.27	9.65	1.41	8.94	1.71	5.21	0.71	4.92	0.75	3.06	1.04	31.1	14.5	2.58
MR14-E02	PC01	1	54	56	55	0.55	307	2.93	2.23	9.55	0.10	3.02	0.72	0.50	152	95.7	0.66	6.15	72.1	144	212	148	141	159	46.0	110	15.4	23.8	14.0	684	42.6	107	12.7	48.8	10.8	2.45	10.6	1.57	9.92	1.96	5.79	0.81	5.45	0.84	3.20	1.08	34.4	14.5	2.71
MR14-E02	PC01	2	4	6	111.7	1.117	315	2.76	2.15	9.14	0.11	2.89	0.73	0.49	145	89.9	0.68	5.88	76.8	150	236	144	133	155	50.4	109	15.4	22.4	13.3	607	43.7	104	13.1	50.8	11.3	2.59	11.3	1.67	10.6	2.11	6.29	0.88	5.81	0.91	3.14	1.06	33.4	14.1	2.65
MR14-E02	PC01	2	54	56	161.7	1.617	329	2.81	2.17	9.14	0.12	2.88	0.75	0.53	151	89.3	0.74	6.17	81.8	154	248	152	132	161	53.9	115	15.8	23.6	13.2	561	45.6	106	13.7	53.1	11.9	2.76	11.9	1.75	11.3	2.20	6.68	0.91	6.16	0.96	3.29	1.06	34.6	14.0	2.68
MR14-E02	PC01	3	4	6	211.9	2.119	358	2.79	2.21	9.35	0.13	2.96	0.77	0.57	156	93.1	0.76	6.37	87.1	160	252	155	136	163	60.5	120	16.3	24.2	13.4	524	49.3	111	15.0	58.8	13.1	3.09	13.5	1.92	12.6	2.42	7.42	0.99	6.82	1.06	3.42	1.11	36.0	14.4	2.79
MR14-E02	PC01	3	54	56	261.9	2.619	348	2.89	2.18	9.24	0.13	2.90	0.77	0.54	148	86.6	0.78	6.16	87.3	168	255	152	131	163	60.4	119	15.8	26.4	13.0	481	47.9	105	14.7	57.4	12.8	3.01	13.1	1.86	12.5	2.36	7.43	0.98	6.82	1.05	3.37	1.08	34.7	13.9	2.70
MR14-E02	PC01	4	4	6	311.5	3.115	384	2.83	2.15	9.22	0.15	2.90	0.80	0.55	152	85.4	0.82	6.22	94.2	170	254	153	133	171	70.0	121	16.2	27.6	13.3	474	52.8	110	16.3	64.2	14.4	3.42	15.1	2.09	14.3	2.67	8.54	1.09	7.78	1.20	3.48	1.09	36.8	14.2	2.79
MR14-E02	PC01	4	54	56	361.5	3.615	422	2.59	1.95	8.45	0.18	2.54	0.84	0.45	143	74.0	0.93	5.91	105	205	291	149	113	168	87.5	120	14.7	29.9	12.3	409	57.8	104	17.8	71.8	16.2	3.95	17.8	2.47	17.1	3.22	10.4	1.31	9.38	1.46	3.39	0.93	36.9	12.6	2.58
MR14-E02	PC01	5	4	6	411.1	4.111	414	2.81	2.03	8.57	0.18	2.53	0.81	0.50	145	76.4	1.01	5.98	117	239	333	156	111	160	85.1	123	15.4	34.6	11.9	398	56.4	103	17.5	70.5	15.9	3.87	17.4	2.44	16.6	3.18	9.99	1.28	9.01	1.40	3.48	0.94	36.3	11.9	2.52
MR14-E02	PC01	5	54	56	461.1	4.611	420	2.80	1.99	8.44	0.18	2.49	0.81	0.47	139	75.1	0.87	5.79	103	185	286	149	108	157	87.7	121	15.0	33.1	11.6	385	57.8	100	17.9	72.2	16.3	4.00	18.0	2.58	17.3	3.36	10.4	1.33	9.40	1.45	3.44	0.94	34.1	11.9	2.55
MR14-E02	PC01	6	4	6	511.6	5.116	457	2.98	2.08	8.84	0.20	2.64	0.87	0.55	147	75.8	0.92	6.27	106	168	299	154	113	179	97.1	133	16.4	37.5	12.6	374	63.6	107	19.4	78.3	17.4	4.30	19.4	2.80	18.8	3.73	11.3	1.47	10.4	1.61	3.75	1.02	36.9	12.4	2.75
MR14-E02	PC01	6	54	56	561.6	5.616	451	2.95	2.12	9.14	0.19	2.77	0.85	0.56	147	78.6	0.85	6.29	99.4	155	284	159	119	181	93.0	135	17.2	36.0	13.0	371	62.7	109	19.4	77.9	17.5	4.27	18.9	2.79	18.2	3.66	10.9	1.43	9.94	1.53	3.77	1.09	35.5	13.0	2.78
MR14-E02	PC01	7	4	6	611.5	6.115	494	2.99	2.04	8.73	0.21	2.61	0.92	0.56	144	73.5	0.92	6.25	107	191	288	158	109	182	106	134	17.2	38.8	12.0	364	68.4	110	21.3	86.6	19.5	4.84	21.6	3.23	20.6	4.22	12.4	1.63	11.2	1.73	3.72	1.03	35.4	12.8	2.69
MR14-E02	PC01	7	54	56	661.5	6.615	557	3.32	2.07	8.84	0.25	2.67	1.03	0.58	139	66.8	0.98	6.31	120	190	288	161	99.2	192	140	138	15.9	41.8	10.2	328	74.3	111	23.1	94.5	21.4	5.35	24.4	3.72	23.5	4.93	14.2	1.90	12.9	2.01	3.89	0.94	34.9	11.8	2.71
MR14-E02	PC01	8	4	6	711.1	7.111	557	3.59	2.08	9.47	0.25	2.93	1.12	0.57	137	66.0	0.93	6.40	118	184	290	159	102	216	141	138	14.9	41.7	10.3	350	72.8	112	22.9	93.7	21.7	5.43	24.6	3.73	23.7	4.95	14.3	1.93	12.9	2.00	3.96	0.89	34.5	12.0	2.70
MR14-E02	PC01	8	54	56	761.1	7.611	608	3.63	2.06	9.49	0.27	2.91	1.14	0.52	137	65.9	1.01	6.24	128	199	293	164	103	216	157	141	15.8	40.9	10.5	336	82.6	118	24.5	101	23.2	5.79	26.6	4.06	26.0	5.46	15.8	2.15	14.3	2.24	4.11	0.91	34.8	12.4	2.80
MR14-E02	PC01	9	4	6	811.4	8.114	618	3.56	2.01	9.40	0.28	2.94	1.10	0.49	134	62.9	1.02	6.10	128	214	305	154	103	191	160	139	15.2	40.9	10.3	326	82.2	118	25.0	103	24.0	6.03	27.5	4.18	26.8	5.58	16.2	2.22	14.8	2.28	4.02	0.90	35.2	12.2	2.78
MR14-E02	PC01	9	54	56	861.4	8.614	635	3.76	1.98	9.59	0.31	2.96	1.19	0.48	135	60.3	1.09	6.16	135	206	307	151	98.9	217	165	134	14.3	44.1	9.39	315	87.7	118	25.4	105	24.6	6.23	28.4	4.30	27.5	5.77	16.8	2.32	15.3	2.35	3.95	0.83	36.3	11.7	2.76
MR14-E02	PC01	10	4	6	911	9.11	579	3.87	1.97	9.25	0.28	2.73	1.17	0.49	138	60.2	1.13	6.33	144	196	312	153	87.6	207	151	130	13.1	47.9	7.83	296	73.5	115	22.8	95.1	22.1	5.60	25.8	3.94	25.3	5.28	15.4	2.13	13.8	2.15	3.79	0.76	36.5	10.8	2.65
MR14-E02	PC01	10	54	56	961	9.61	478	3.75	1.83	9.02	0.23	2.83	1.03	0.44	126	55.6	1.05	5.78	131	175	295	140	85.5	171	108	120	11.7	43.8	6.96	283	62.1	106	19.6	80.8	18.8	4.80	21.6	3.29	20.9	4.34	12.6	1.74	11.2	1.75	3.55	0.69	34.2	9.84	2.30
MR14-E02	PC01	11	4	6	1011	10.11	458	4.04	1.76	9.27	0.24	3.08	1.05	0.43	127	52.8	1.05	5.79	128	167	308	141	86.0	169	106	118	11.3	45.9	6.43	273	59.7	101	18.4	76.0	17.7	4.47	20.6	3.10	19.9	4.18	12.1	1.69	10.9	1.70	3.42	0.67	33.2	9.21	2.26
MR14-E02	PC03	1	4	6	5	0.05	291	2.77	2.02	8.73	0.12	2.51	1.21	0.40	134	73.9	0.58	5.57	72.9	147	226	140	110	170	54.0	101	12.3	6.29	10.2	527	40.3	85.5	11.9	46.8	10.9	2.58	11.1	1.69	10.6	2.15	6.22	0.88	5.80	0.90	2.78	0.84	32.4	12.5	2.02
MR14-E02	PC03	2	4	6	30.1	0.301	468	3.03	1.96	8.89	0.18	2.86	0.90	0.42	135	49.9	1.02	6.00	120	479	374	179	106	165	107	131	13.9	17.3	10.5	356	65.9	100.0	19.3	79.2	18.2	4.53	20.5	3.14	19.7	4.10	11.9	1.67	10.7	1.67	3.51	0.89	41.5	11.9	2.30
MR14-E02	PC03	2	54	56	80.1	0.801	504	3.84	1.68	8.64	0.24	2.97	1.04	0.41	129	42.8	1.12	5.55	145	300	319	165	81.2	172	122	126	11.6	53.9	6.27	256	71.1	99.0	20.7	85.4	19.8	4.98	22.8	3.48	22.0	4.55	13.2	1.85	11.9	1.84	3.33	0.67	37.0	8.65	2.26
MR14-E02	PC03	3	4	6	132	1.320	561	4.06	1.87	9.23	0.28	3.07	1.14	0.46	140	47.8	1.16	6.01	151	275	352	166	86.4	189	134	135	12.1	65.0	6.98	270	79.7	110	23.2	96.4	22.4	5.59	25.5	3.87	24.3	5.02	14.5	2.02	12.9	2.00	3.59	0.72	39.2	10.1	2.48
MR14-E02	PC03	3	54	56	182	1.820	609	4.34	1.91	9.55	0.29	3.22	1.16	0.44	151	39.2	1.50	6.14	186	312	367	173	91.7	201	142	145	12.9	87.5	7.32	295	85.0	132	24.6	101	23.5	5.82	26.7	4.03	25.3	5.22	15.1	2.11	13.4	2.09	3.95	0.75	47.0	11.3	2.67
MR14-E02	PC03	4	4	6	231.5	2.315	357	4.73	1.40	8.84	0.14	3.53	0.67	0.30	81.2	26.9	0.83	3.87	96.4	180	294	120	69.7	120	66.3	132	12.7	49.2	3.72	179	44.8	108	14.5	58.3	13.8	2.81	14.5	2.23	13.5	2.65	7.48	1.03	6.47	0.98	4.17	0.96	34.8	14.2	1.43
MR14-E02	PC03	4	54	56	281.5	2.815	629	4.10	1.69	8.82	0.32	3.14	1.31	0.41	138	36.5	1.48	5.74	185	252	340	151	82.3	201	149	138	11.9	86.2	6.09	284	85.3	132	25.8	107	25.3	6.25	28.3	4.26	26.4	5.42	15.5	2.16	13.7	2.13	3.81	0.68	44.9	11.1	2.59
MR14-E02	PC03	5	4	6	331.5	3.315	1043	4.13	1.51	8.68	0.56	3.23	1.86	0.37	128	33.4	1.33	5.42	165	295	350	143	77.8	224	290	121	10.4	70.6	4.94	263	152	131	45.5	191	45.5	11.3	51.8	7.77	48.2	9.80	27.9	3.81	23.7	3.61	3.36	0.55	40.0	13.0	2.91
MR14-E02	PC03	5	54	56	381.5	3.815	2471	3.73	1.63	7.72	1.38	2.60	4.39	0.41	137	34.3	1.78	5.66	217	434	384	169	65.7	350	727	135	11.9	77.6	4.68	257	368	210	119	471	113	27.8	128	19.2	120	24.4	68.8	9.30	56.9	8.59	3.75	0.63	46.6	20.7	4.62
MR14-E02	PC03	6	4	6	432.5	4.325	5740	2.79	1.43	6.04	4.13	2.40	9.78	0.30	104	28.0	2.47	4.25	311	756	428	199	57.0	616	1709	134	11.7	72.6	3.60	284	841	409	274	1176	260	64.6	297	44.2	276	56.5	159	21.5	131	19.7	4.04	0.49	49.4	37.4	8.67
MR14-E02	PC03	6	54	56	482.5	4.825	2332	2.79	1.94	8.52	1.38	3.28	4.17	0.32	131	41.6	1.75	5.16	187	524	304	181	114	320	639	136	11.9	76.1	9.75	370	339	252	114	453	110	27.3	121	18.1	110	22.0	61.1	8.18	49.6	7.37	3.98	0.71	54.8	28.1	5.41
MR14-E02	PC03	7	4	6	531.2	5.312	1252	2.74	2.17	9.55	0.70	3.55	1.90	0.34	143	48.8	1.61	5.40	187	435	267	190	137	204	314	136	12.5	70.6	12.3	406	177	208	55.6	230	56.6	13.8	60.4	9.04	54.6	10.9	30.2	4.07	25.1	3.73	3.90	0.81	55.9	22.6	3.78
MR14-E02	PC03	7	54	56	581.2	5.812	993	2.73	2.16	9.72	0.51	3.60	1.43	0.34	145	55.0	1.49	5.44	173	450	244	191	142	176	238	133	12.5	62.6	12.9	394	138	191	42.8	177	43.1	10.5	45.9	6.83	41.8	8.33	23.2	3.15	19.5	2.93	3.86	0.83	52.7	20.3	3.24
MR14-E02	PC03	8	4	6	631.7	6.317	958	2.79	2.11	9.57	0.45	3.52	1.29	0.34	139	45.7	1.57	5.45	172	396	240	190	134	175	230	128	12.4	62.3	12.3	392	134	185	41.4	171	41.4	10.0	44.2	6.61	40.2	8.03	22.4	3.04	18.7	2.78	3.77	0.80	53.5	18.4	2.85
MR14-E02	PC03	8	54	56	681.7	6.817	1029	2.78	2.06	9.34	0.48	3.43	1.34	0.33	135	47.3	1.58	5.35	166	399	234	189	131	179	246	126	12.3	58.7	12.0	388	145	191	45.2	187	45.5	11.0	48.2	7.21	43.7	8.69	24.1	3.25	19.9	2.96	3.76	0.79	53.7	18.9	2.80
MR14-E02	PC03	9	4	6	731.4	7.314	1035	2.64	1.92	8.71	0.51	3.17	1.38	0.31	126	41.7	1.54	4.93	162	373	217	177	121	178	253	120	11.6	54.1	11.0	368	147	182	45.8	190	46.2	11.2	49.1	7.31	44.3	8.79	24.5	3.30	20.1	3.00	3.50	0.73	52.1	17.9	2.70
MR14-E02	PC03	9	54	56	781.4	7.814	1031	2.59	1.93	8.64	0.51	3.12	1.38	0.31	126	41.8	1.56	5.02	162	373	224	175	120	180	250	123	11.4	53.3	10.9	372	146	183	45.7	189	46.0	11.2	49.1	7.34	44.2	8.79	24.4	3.31	20.2	3.01	3.58	0.73	53.5	17.7	2.72
MR14-E02	PC03	10	4	6	831	8.310	813	2.77	1.98	8.93	0.39	3.22	1.10	0.30	127	42.0	1.64	5.10	166	410	228	180	126	166	184	123	11.3	52.5	11.4	379	113	173	35.0	144	35.0	8.45	36.7	5.49	33.3	6.61	18.4	2.50	15.2	2.26	3.59	0.72	55.3	16.2	2.45
MR14-E02	PC03	10	54	56	881	8.810	704	2.89	2.07	9.45	0.36	3.57	1.05	0.32	130	58.1	1.67	5.30	170	396	228	181	131	203	150	132	11.8	51.5	11.3	446	92.4	170	29.9	122	29.2	7.15	30.9	4.66	28.4	5.69	16.1	2.21	13.5	2.04	3.95	0.76	56.2	15.5	2.40
MR14-E02	PC03	11	4	6	932	9.320	728	2.78	1.95	8.99	0.36	3.26	1.04	0.30	123	39.7	1.55	5.01	151	400	224	179	123	164	163	123	11.3	46.1	11.0	380	96.7	158	31.8	130	31.3	7.61	33.3	5.00	30.3	6.05	16.9	2.29	14.1	2.10	3.61	0.74	52.4	14.9	2.29
MR14-E02	PC03	11	54	56	982	9.820	835	2.67	1.90	8.61	0.40	3.11	1.13	0.29	120	41.1	1.60	4.85	162	435	230	176	118	161	195	117	11.4	47.0	10.4	364	116	163	36.6	151	36.8	8.96	38.9	5.83	35.0	6.96	19.4	2.60	16.0	2.35	3.47	0.74	51.6	15.0	2.28
MR14-E02	PC03	12	4	6	1030.4	10.304	796	2.75	2.01	9.03	0.36	3.22	1.06	0.30	128	45.7	1.79	5.13	192	465	236	183	122	163	179	123	11.8	48.7	10.9	392	106	171	34.9	143	34.7	8.37	36.5	5.45	32.9	6.52	17.9	2.41	14.6	2.17	3.71	0.72	52.7	14.6	2.28
MR14-E02	PC04	1	4	6	5	0.050	271	2.70	2.14	8.96	0.08	2.76	0.79	0.41	159	87.2	0.56	5.61	65.5	129	200	166	129	155	41.5	106	14.1	12.5	12.5	668	39.9	90.9	11.4	43.4	9.40	2.18	9.31	1.40	8.60	1.73	5.03	0.72	4.72	0.73	2.93	1.00	32.3	13.5	2.42
MR14-E02	PC04	1	54	56	55	0.550	295	2.65	2.17	9.28	0.09	2.93	0.69	0.44	171	91.1	0.60	5.93	68.1	134	212	146	136	157	46.5	113	15.1	21.5	13.1	661	43.5	96.2	12.5	47.3	10.4	2.37	10.3	1.55	9.55	1.93	5.66	0.81	5.24	0.81	3.13	1.06	36.2	13.9	2.64
MR14-E02	PC04	2	4	6	90	0.900	311	2.63	2.10	8.95	0.10	2.83	0.70	0.43	164	85.9	0.65	5.74	75.0	143	234	144	131	157	53.1	114	15.2	21.5	13.1	577	45.5	96.6	12.9	50.2	11.1	2.57	11.1	1.68	10.4	2.13	6.22	0.89	5.78	0.90	3.12	1.04	35.2	13.8	2.58
MR14-E02	PC04	2	54	56	140	1.400	330	2.66	2.16	9.14	0.11	2.83	0.73	0.45	173	86.2	0.73	6.10	83.5	152	252	152	129	167	58.2	120	15.5	23.6	12.9	546	48.3	97.1	13.9	54.3	12.0	2.84	12.4	1.88	11.5	2.38	6.91	0.98	6.40	0.98	3.25	1.06	37.7	13.7	2.65
MR14-E02	PC04	3	4	6	188.6	1.886	288	2.42	1.89	7.07	0.12	2.10	0.69	0.19	139	62.2	0.68	5.48	79.0	139	246	140	93.7	136	55.6	65.6	3.00	22.0	10.7	373	40.1	76.7	12.2	48.7	11.3	2.76	12.1	1.81	11.1	2.23	6.41	0.88	5.69	0.88	1.73	b.d.l.	34.3	9.88	1.42
MR14-E02	PC04	3	54	56	238.6	2.386	336	2.63	1.97	8.35	0.13	2.62	0.73	0.41	158	75.3	0.74	5.57	86.4	156	246	143	117	156	64.9	110	14.2	25.4	11.8	424	48.7	89.3	14.1	56.0	12.6	3.04	13.3	2.02	12.6	2.59	7.49	1.07	6.88	1.07	2.96	0.96	35.1	12.5	2.39
MR14-E02	PC04	4	4	6	290.1	2.901	358	2.57	1.99	8.58	0.14	2.72	0.74	0.43	165	76.9	0.76	5.79	89.4	161	250	147	122	160	69.3	117	15.1	26.2	12.4	425	52.0	93.8	15.2	60.2	13.6	3.22	14.4	2.16	13.5	2.76	8.05	1.13	7.29	1.13	3.15	1.02	36.8	13.0	2.53
MR14-E02	PC04	4	54	56	340.1	3.401	464	2.85	2.08	9.08	0.20	2.69	0.91	0.45	178	74.3	1.02	6.36	117	213	317	162	119	185	102	134	15.8	33.6	13.0	431	66.3	106	19.4	78.2	17.7	4.34	19.6	2.99	18.9	3.94	11.4	1.62	10.5	1.62	3.60	0.99	43.2	13.1	2.73
MR14-E02	PC04	5	4	6	389.1	3.891	400	2.76	1.95	8.26	0.17	2.42	0.77	0.40	161	70.2	0.96	5.69	113	218	300	148	105	161	86.7	123	14.5	34.4	11.3	376	57.0	93.0	16.8	67.8	15.3	3.79	16.9	2.56	16.1	3.36	9.68	1.37	8.74	1.36	3.27	0.89	37.2	11.2	2.40
MR14-E02	PC04	5	54	56	439.1	4.391	458	2.94	2.08	8.82	0.20	2.57	0.87	0.44	172	69.6	0.95	6.11	115	204	315	160	110	177	103	131	15.4	37.2	11.6	403	65.5	98.5	19.5	78.4	17.9	4.41	19.8	3.01	19.0	3.96	11.5	1.61	10.3	1.60	3.53	0.94	38.6	12.1	2.60
MR14-E02	PC04	6	4	6	489.1	4.891	462	3.05	2.10	9.02	0.20	2.69	0.87	0.46	175	72.6	0.93	6.36	110	171	309	161	114	184	102	137	16.3	39.6	12.5	382	67.3	99.2	19.8	80.1	18.2	4.45	20.0	3.03	19.1	3.97	11.5	1.63	10.4	1.61	3.67	1.00	40.0	12.5	2.68
MR14-E02	PC04	6	54	56	539.1	5.391	400	2.74	1.88	7.87	0.18	2.31	0.74	0.41	154	62.1	0.83	5.60	100	166	288	145	97.7	163	88.1	123	14.3	36.6	10.8	318	57.7	87.2	16.9	68.7	15.8	3.84	17.3	2.60	16.4	3.43	9.95	1.40	8.99	1.39	3.29	0.87	35.5	10.7	2.31
MR14-E02	PC04	7	4	6	590.1	5.901	563	3.30	1.96	8.85	0.23	2.81	1.08	0.53	133	48.3	0.96	5.98	129	228	311	159	98.6	193	145	135	14.4	41.6	9.57	322	81.5	104	23.6	90.8	22.3	5.61	25.5	3.85	24.4	5.10	14.8	2.05	13.2	2.05	3.54	0.85	36.7	11.4	2.49
MR14-E02	PC04	7	54	56	640.1	6.401	549	3.31	2.17	9.38	0.23	2.82	1.06	0.61	153	61.7	0.99	6.66	124	195	316	168	110	213	134	152	17.5	43.0	11.6	360	81.5	108	23.3	89.2	21.6	5.35	24.1	3.63	23.1	4.77	13.8	1.93	12.4	1.92	3.81	1.01	39.5	12.3	2.63
MR14-E02	PC04	8	4	6	688.7	6.887	523	3.38	1.92	8.67	0.21	2.77	1.06	0.51	129	55.8	0.85	5.94	118	191	296	154	95.2	193	131	136	13.8	37.7	9.39	319	76.1	99.4	21.7	84.2	20.6	5.17	23.5	3.56	22.8	4.72	13.7	1.91	12.3	1.91	3.62	0.84	35.3	11.3	2.46
MR14-E02	PC04	8	54	56	738.7	7.387	575	3.45	1.97	9.18	0.24	2.84	1.14	0.49	137	60.2	0.95	6.08	130	192	302	158	99.3	212	150	145	15.0	41.5	9.86	329	82.3	107	23.4	90.7	22.2	5.59	25.6	3.89	25.0	5.26	15.3	2.13	13.8	2.15	3.86	0.85	36.6	11.5	2.60
MR14-E02	PC04	9	4	6	789.7	7.897	600	3.67	1.89	9.33	0.27	2.88	1.20	0.50	133	48.7	1.00	5.97	135	214	325	154	91.8	203	163	139	13.6	43.9	8.35	297	84.5	104	24.3	95.2	23.8	6.01	27.5	4.19	26.9	5.66	16.5	2.28	14.8	2.33	3.69	0.76	36.8	10.6	2.51
MR14-E02	PC04	9	54	56	839.7	8.397	439	3.41	1.62	8.39	0.19	2.73	0.96	0.41	120	34.0	0.92	5.30	123	180	298	137	79.8	172	108	118	10.4	42.9	6.18	267	61.2	89.0	17.8	69.4	17.4	4.41	20.0	3.03	19.3	4.01	11.5	1.60	10.3	1.62	3.13	0.63	33.6	9.10	2.08
MR14-E02	PC04	10	4	6	888.9	8.889	494	3.49	1.51	8.22	0.23	2.85	1.04	0.40	117	39.9	0.94	5.19	128	178	330	132	76.3	168	125	115	10.3	43.4	5.54	238	72.0	84.7	21.1	82.4	20.6	5.13	23.5	3.55	22.4	4.63	13.3	1.86	11.9	1.84	3.03	0.60	32.6	8.48	2.04
MR14-E02	PC04	10	54	56	938.9	9.389	702	3.24	1.36	7.60	0.37	2.84	1.38	0.35	102	30.5	1.22	4.80	155	318	335	137	71.3	177	185	108	9.00	51.0	4.58	241	102	109	30.5	121	30.5	7.52	34.5	5.17	32.1	6.52	18.4	2.50	15.7	2.39	2.95	0.51	34.5	10.3	2.20
MR14-E02	PC04	11	4	6	989	9.890	575	2.48	2.24	8.74	0.32	3.76	0.93	0.35	87.9	37.6	0.61	4.40	59.8	176	225	194	122	122	121	121	14.0	14.5	7.56	316	87.4	130	26.1	98.5	23.8	5.60	25.0	3.71	22.4	4.47	12.5	1.72	10.8	1.60	3.52	0.96	24.0	13.6	2.14
MR14-E02	PC04	11	54	56	1039	10.390	458	2.14	1.96	7.74	0.23	3.26	0.70	0.30	78.1	36.3	0.44	3.86	45.1	125	170	162	107	98.5	94.4	107	11.9	9.11	6.68	253	68.6	111	20.4	76.5	18.8	4.39	19.5	2.91	17.6	3.53	9.90	1.37	8.50	1.25	3.09	0.83	18.6	12.1	1.81
MR14-E02	PC06	1	4	6	5	0.050	270	2.88	2.30	9.27	0.11	2.82	0.86	0.45	159	87.9	0.53	5.47	67.0	137	219	149	127	154	43.2	116	13.9	9.16	12.2	667	39.5	91.3	11.2	41.4	9.36	2.13	9.17	1.39	8.50	1.69	4.97	0.71	4.61	0.71	2.90	0.98	34.3	13.5	2.37
MR14-E02	PC06	1	54	56	55	0.550	288	2.91	2.28	9.66	0.12	2.88	0.76	0.48	164	87.0	0.63	5.85	74.8	142	232	142	129	159	49.9	120	14.3	16.0	12.5	604	42.6	91.1	12.0	44.7	10.2	2.35	10.1	1.53	9.58	1.90	5.60	0.80	5.21	0.80	3.00	0.99	37.4	13.2	2.45
MR14-E02	PC06	2	4	6	83	0.830	296	2.96	2.33	9.88	0.11	2.99	0.69	0.51	169	89.2	0.62	6.03	72.7	140	236	147	137	163	47.3	131	15.3	18.2	13.4	696	43.6	99.0	12.3	45.9	10.5	2.36	10.2	1.55	9.45	1.91	5.55	0.80	5.16	0.80	3.33	1.05	39.2	14.4	2.67
MR14-E02	PC06	2	54	56	133	1.330	306	2.92	2.26	9.47	0.13	2.87	0.71	0.51	162	85.8	0.65	5.71	74.1	140	255	143	129	156	54.9	128	15.0	22.0	12.7	597	45.5	93.0	12.8	47.6	10.8	2.52	11.0	1.69	10.4	2.12	6.20	0.90	5.78	0.89	3.16	1.03	37.9	13.4	2.56
MR14-E02	PC06	3	4	6	183.2	1.832	315	2.76	2.18	9.10	0.13	2.80	0.70	0.52	162	83.0	0.69	5.80	79.4	144	263	143	126	158	56.3	132	15.2	22.7	12.5	534	46.2	94.4	13.2	49.7	11.4	2.67	11.5	1.76	10.9	2.23	6.46	0.93	6.02	0.93	3.28	1.05	39.0	13.6	2.63
MR14-E02	PC06	3	54	56	233.2	2.332	365	3.07	2.27	9.48	0.16	2.91	0.79	0.55	176	81.2	0.78	6.35	91.5	157	289	156	133	174	70.0	145	16.4	25.7	13.2	527	53.1	102	15.4	58.2	13.5	3.22	14.0	2.13	13.3	2.74	7.95	1.14	7.36	1.14	3.58	1.10	42.8	14.0	2.80
MR14-E02	PC06	4	4	6	282.9	2.829	370	3.01	2.28	9.61	0.16	2.96	0.78	0.52	171	80.0	0.81	6.12	99.7	191	286	155	132	169	73.5	141	16.0	27.7	13.1	465	54.0	100.0	15.6	59.4	13.6	3.33	14.4	2.19	13.8	2.82	8.16	1.17	7.50	1.16	3.38	1.06	42.7	14.0	2.74
MR14-E02	PC06	4	54	56	332.9	3.329	463	2.99	2.19	9.34	0.22	2.75	0.89	0.48	177	69.0	1.04	6.34	122	231	327	162	122	183	106	151	15.8	34.6	13.3	460	66.5	102	19.6	76.0	17.7	4.35	19.6	3.00	19.0	3.96	11.6	1.63	10.5	1.64	3.64	1.00	47.6	13.3	2.79
MR14-E02	PC06	5	4	6	382.8	3.828	444	3.25	2.27	9.41	0.21	2.73	0.85	0.48	178	57.5	1.07	6.27	132	251	360	167	116	179	98.8	153	16.3	41.5	12.4	433	63.5	102	18.7	73.1	17.1	4.22	18.8	2.86	17.8	3.74	10.8	1.52	9.82	1.51	3.76	0.99	46.2	12.5	2.72
MR14-E02	PC06	5	54	56	432.8	4.328	447	3.09	2.18	9.16	0.21	2.67	0.83	0.46	169	62.8	0.90	6.05	111	187	325	158	113	174	102	152	15.9	36.4	12.1	405	64.4	96.2	19.1	74.4	17.4	4.26	19.3	2.91	18.4	3.83	11.2	1.57	10.2	1.58	3.73	1.00	41.9	12.4	2.69
MR14-E02	PC06	6	4	6	484	4.840	468	3.47	2.25	9.37	0.22	2.73	0.88	0.52	177	60.8	0.95	6.48	118	168	343	164	113	188	108	165	16.7	43.2	12.5	383	67.9	101	19.7	76.6	17.8	4.43	19.8	3.02	19.0	4.00	11.5	1.64	10.6	1.65	3.99	1.03	45.2	12.5	2.82
MR14-E02	PC06	6	54	56	534	5.340	524	3.50	2.29	9.72	0.25	2.87	0.97	0.55	181	60.8	0.99	6.67	121	208	341	170	118	198	124	170	18.5	45.0	12.9	386	75.8	107	22.5	88.3	20.4	5.02	22.8	3.44	21.6	4.50	13.1	1.84	11.8	1.83	4.06	1.11	44.4	13.4	2.89
MR14-E02	PC06	7	4	6	584.3	5.843	572	3.63	2.25	9.74	0.29	2.95	1.09	0.55	170	58.8	0.98	6.52	130	207	319	165	107	197	146	165	15.6	43.2	11.0	366	80.3	105	24.0	95.8	22.3	5.70	25.7	3.92	24.9	5.24	15.2	2.15	13.8	2.15	3.90	0.94	41.6	12.6	2.79
MR14-E02	PC06	7	54	56	634.3	6.343	583	3.77	1.99	9.57	0.32	2.90	1.11	0.48	153	46.6	0.99	5.92	133	229	333	158	93.0	188	157	154	13.8	43.9	8.78	306	80.1	100	23.7	96.0	22.8	5.84	26.7	4.07	25.8	5.44	15.9	2.23	14.4	2.24	3.72	0.77	39.7	11.0	2.62
MR14-E02	PC06	8	4	6	684.3	6.843	480	3.81	1.92	9.66	0.26	3.05	0.98	0.50	156	43.2	1.06	5.97	138	232	327	160	90.6	177	120	150	12.1	45.4	7.54	326	64.3	99.2	19.3	78.1	18.7	4.75	21.3	3.23	20.4	4.23	12.3	1.72	11.2	1.73	3.60	0.72	41.2	10.7	2.42
MR14-E02	PC06	8	54	56	734.3	7.343	500	4.19	1.85	9.78	0.27	3.33	1.02	0.48	151	39.1	1.06	5.85	133	192	345	145	92.9	175	128	143	12.2	49.5	7.31	280	69.0	95.3	20.5	83.2	19.5	4.93	22.3	3.40	21.5	4.46	12.8	1.80	11.5	1.79	3.47	0.72	39.7	9.66	2.37
MR14-E02	PC06	9	4	6	783.8	7.838	581	4.32	1.87	9.69	0.35	3.28	1.17	0.51	148	40.8	1.11	5.76	147	244	382	146	87.1	182	152	144	11.9	50.3	6.63	269	81.8	98.8	24.6	99.6	23.5	5.88	27.0	4.09	25.6	5.29	15.1	2.10	13.5	2.10	3.43	0.70	39.3	10.5	2.42
MR14-E02	PC06	9	54	56	833.8	8.338	624	4.01	1.71	9.17	0.36	3.15	1.15	0.44	137	44.8	1.24	5.25	157	250	361	140	86.1	176	161	139	11.4	52.4	6.47	265	87.5	109	26.4	107	25.4	6.23	28.9	4.36	27.2	5.64	16.2	2.25	14.4	2.22	3.39	0.64	41.9	11.5	2.43
MR14-E02	PC06	10	4	6	883.7	8.837	603	4.06	1.71	9.06	0.35	3.14	1.21	0.47	139	38.5	1.37	5.60	164	257	394	146	85.6	191	152	156	12.2	60.0	6.19	286	83.0	113	25.4	102	24.4	6.06	27.6	4.18	26.1	5.33	15.2	2.12	13.4	2.05	3.74	0.69	46.1	11.2	2.39
MR14-E02	PC06	10	54	56	933.7	9.337	681	4.00	1.62	8.74	0.42	3.14	1.38	0.48	131	39.0	1.44	5.39	187	275	373	144	81.1	197	174	146	11.7	58.4	5.72	286	92.4	121	29.2	119	28.6	7.06	32.0	4.83	29.9	6.11	17.4	2.41	15.2	2.35	3.65	0.64	46.5	11.6	2.49
MR14-E02	PC06	11	4	6	983.4	9.834	991	3.95	1.58	8.80	0.62	3.17	1.80	0.46	132	38.4	1.52	5.47	188	460	452	151	79.3	217	276	137	11.2	54.5	5.37	286	136	127	44.3	182	44.0	11.0	50.0	7.53	46.6	9.57	27.0	3.68	23.0	3.50	3.45	0.61	46.3	13.3	2.76
MR14-E02	PC06	11	54	56	1033.4	10.334	1799	3.74	1.61	8.06	1.17	2.72	2.76	0.52	139	32.7	1.62	5.74	200	374	404	159	70.0	268	552	150	13.0	54.8	4.92	268	261	158	80.4	333	81.0	20.1	92.6	13.9	85.9	17.6	49.5	6.62	41.2	6.18	3.70	0.69	51.0	17.1	3.70
MR14-E02	PC08	1	4	6	5	0.05	264	2.64	2.00	8.28	0.09	2.50	0.91	0.39	149	80.1	0.56	5.25	66.5	138	200	149	115	150	43.3	100	12.9	7.90	11.1	570	37.8	84.0	11.2	42.5	9.65	2.26	9.72	1.47	8.98	1.82	5.29	0.74	5.00	0.77	2.75	0.91	29.4	12.8	2.13
MR14-E02	PC08	1	54	56	55	0.55	409	2.85	1.75	8.07	0.16	2.64	0.78	0.38	138	52.2	0.90	5.30	102	386	299	158	95.6	143	94.5	124	12.5	22.2	9.31	346	57.4	81.7	17.6	70.6	16.7	4.14	18.9	2.86	18.0	3.72	10.7	1.48	9.66	1.50	3.18	0.76	34.1	10.7	2.08
MR14-E02	PC08	2	4	6	93.5	0.935	498	3.76	1.66	8.50	0.25	2.94	1.01	0.39	134	40.6	0.99	5.38	123	416	314	165	81.6	157	124	127	11.1	38.8	6.52	262	70.3	87.0	21.2	85.7	20.3	5.08	23.5	3.56	22.5	4.69	13.6	1.91	12.4	1.93	3.31	0.66	32.0	9.37	2.20
MR14-E02	PC08	2	54	56	143.5	1.435	541	4.18	1.67	8.37	0.30	2.88	1.21	0.39	141	40.7	1.35	5.54	156	353	346	164	78.5	184	126	137	11.7	76.6	5.99	276	74.7	115	22.4	90.4	21.5	5.35	24.4	3.68	23.1	4.82	13.9	1.95	12.3	1.93	3.66	0.69	38.9	9.99	2.55
MR14-E02	PC08	3	4	6	192.4	1.924	670	3.86	1.53	8.01	0.37	2.90	1.37	0.38	136	37.2	1.51	5.23	188	293	320	150	75.0	192	165	129	11.1	85.8	5.34	274	93.2	124	28.5	116	28.0	6.92	31.5	4.74	29.3	6.02	17.2	2.37	15.0	2.31	3.49	0.60	40.4	10.7	2.64
MR14-E02	PC08	3	54	56	242.4	2.424	1900	3.58	1.50	7.68	1.13	2.73	3.62	0.37	144	30.6	2.12	5.36	290	541	386	172	70.3	311	548	128	11.5	96.2	4.88	295	282	183	86.1	356	86.9	21.5	99.3	14.8	91.5	18.7	53.0	7.14	43.9	6.65	3.51	0.58	50.2	18.0	4.20
MR14-E02	PC08	4	4	6	293.4	2.934	1324	3.09	1.85	8.15	0.89	3.04	2.27	0.29	133	32.7	2.17	4.86	237	623	305	196	103	221	347	124	11.1	106	8.74	390	190	203	58.2	238	58.8	14.5	64.4	9.60	58.4	11.8	33.2	4.53	27.9	4.20	3.56	0.67	48.0	16.2	3.69
MR14-E02	PC08	4	54	56	343.4	3.434	1032	2.97	1.96	8.34	0.67	3.15	1.74	0.30	128	38.3	1.86	4.73	189	603	286	200	111	187	254	119	10.8	96.3	9.36	396	147	181	45.3	185	45.5	11.3	49.5	7.39	44.9	8.97	25.0	3.36	20.5	3.03	3.43	0.70	50.5	14.1	3.15
MR14-E02	PC08	5	4	6	393.6	3.936	935	2.88	1.98	8.27	0.62	3.17	1.59	0.30	131	39.2	1.80	4.87	160	513	254	199	115	179	233	116	11.1	89.0	9.84	408	134	165	40.6	166	40.6	10.0	44.4	6.62	40.3	8.14	22.7	3.05	18.8	2.78	3.33	0.73	54.0	13.9	2.89
MR14-E02	PC08	5	54	56	443.6	4.436	1058	2.85	2.11	8.95	0.65	3.39	1.62	0.33	133	46.3	1.62	5.00	139	528	232	199	128	177	266	118	11.4	76.9	11.0	395	151	174	46.9	192	47.4	11.8	51.6	7.71	46.6	9.36	25.9	3.49	21.4	3.16	3.35	0.78	52.0	16.3	3.11
MR14-E02	PC08	6	4	6	494.1	4.941	2236	2.98	1.92	8.50	1.35	3.30	3.63	0.31	126	40.2	1.85	4.61	177	572	273	199	110	298	602	120	11.1	82.5	9.11	392	323	237	116	436	109	27.3	120	17.8	107	21.2	58.0	7.69	46.0	6.76	3.62	0.73	54.7	22.8	4.36
MR14-E02	PC08	6	54	56	544.1	5.441	1882	2.80	1.86	8.56	1.15	3.27	3.16	0.33	129	38.8	1.93	4.82	178	550	281	203	106	266	507	123	11.4	83.1	8.62	412	272	226	86.1	357	89.1	22.2	98.6	14.6	89.2	17.8	49.2	6.55	39.6	5.83	3.66	0.75	63.5	20.9	3.72
MR14-E02	PC08	7	4	6	594.3	5.943	2388	2.97	1.89	8.47	1.55	3.24	4.09	0.35	125	40.0	1.74	4.94	160	513	265	200	107	315	661	123	11.2	71.0	8.82	383	349	230	122	463	116	29.0	128	19.0	115	23.1	63.8	8.49	51.4	7.62	3.70	0.74	56.2	24.2	4.38
MR14-E02	PC08	7	54	56	644.3	6.443	5570	2.62	1.72	6.59	4.37	2.69	8.88	0.33	109	40.4	2.02	4.35	170	654	282	214	84.4	554	1513	126	12.4	55.1	6.60	355	791	391	294	1246	278	69.3	306	45.0	274	54.6	150	20.0	121	17.8	4.03	0.75	56.7	40.8	7.73
MR14-E02	PC08	8	4	6	692.4	6.924	3907	2.38	1.90	6.81	2.59	2.75	6.37	0.30	107	41.7	1.50	4.22	133	447	212	187	96.8	410	1034	118	11.4	43.6	7.97	367	552	312	208	869	194	48.4	214	31.7	192	38.0	104	13.9	83.7	12.4	3.66	0.76	50.5	30.3	6.24
MR14-E02	PC08	8	54	56	742.4	7.424	1655	2.54	2.18	7.72	1.21	3.09	2.81	0.32	123	44.5	1.71	4.74	150	465	225	196	111	227	445	124	12.0	50.1	9.29	426	241	212	74.2	307	75.4	18.8	84.0	12.6	77.1	15.7	44.0	6.00	36.9	5.54	3.63	0.82	60.3	18.2	3.69
MR14-E02	PC08	9	4	6	792.3	7.923	1469	2.67	2.21	7.73	1.04	3.09	2.29	0.32	130	40.9	1.98	4.87	180	481	240	208	109	216	373	126	12.2	58.8	8.99	462	209	226	66.1	272	67.0	16.6	72.6	10.9	65.9	13.2	36.8	5.01	30.7	4.54	3.64	0.83	68.7	17.7	3.27
MR14-E02	PC08	9	54	56	842.3	8.423	1633	2.76	2.23	7.81	1.23	3.13	2.66	0.33	135	46.1	2.13	5.03	191	487	240	211	109	239	421	130	12.5	60.8	8.78	478	232	239	73.8	304	74.5	18.6	82.0	12.2	74.4	15.0	41.5	5.60	34.4	5.11	3.85	0.86	68.5	18.6	3.54
MR14-E02	PC08	10	4	6	892.5	8.925	1300	2.84	2.37	8.05	0.88	5.60	2.34	0.40	146	41.9	2.54	5.27	254	541	279	216	111	236	307	145	13.3	68.0	8.95	528	178	256	57.1	230	57.7	14.3	61.4	9.18	55.5	11.0	30.4	4.11	25.0	3.73	4.03	0.94	82.1	18.8	2.92
MR14-E02	PC08	10	54	56	942.5	9.425	1220	2.95	2.41	8.15	0.93	5.70	2.44	0.40	143	46.0	2.58	5.24	252	570	281	220	113	235	291	144	13.0	66.9	9.18	530	168	240	53.1	213	53.6	13.2	57.0	8.53	51.8	10.3	28.6	3.90	24.1	3.63	3.93	0.91	83.3	18.2	3.01
MR14-E02	PC08	11	4	6	992.3	9.923	1773	2.84	2.33	7.86	1.22	5.55	3.10	0.41	143	42.6	2.53	5.18	250	625	313	213	107	274	457	142	13.1	64.2	8.73	539	251	265	80.8	328	82.3	20.3	88.5	13.1	79.4	15.8	43.9	5.95	36.5	5.46	3.88	0.87	80.0	18.9	3.31
MR14-E02	PC08	11	54	56	1042.3	10.423	2378	3.18	2.37	7.94	1.59	5.63	3.92	0.41	131	45.3	2.07	4.95	218	505	267	193	106	311	646	144	12.8	50.8	8.79	466	350	282	111	449	112	27.8	122	18.1	111	22.1	61.3	8.21	49.8	7.38	3.87	0.83	67.8	20.1	3.68

Table S2 (3: continued)

Cruise	Core	Sec.	Int. [cm]	Int. [cm]	Depth [cm]	Depth [m]	ΣREY [ppm]	Na (wt.%)	Mg	Al	P	K	Ca	Ti	V (ppm)	Cr	Mn (wt.%	Fe	Co (ppm)	Ni	Cu	Zn	Rb	Sr	Y	Zr	Nb	Mo	Cs	Ba	La	Ce	Pr	Nd	Sm	Eu	Gd	Tb	Dy	Ho	Er	Tm	Yb	Lu	Hf	Ta	Pb	Th	U
MR14-E02	PC10	1	4	6	5	0.05	323	2.99	2.23	9.38	0.12	5.02	0.97	0.52	165	94.3	0.67	6.09	82.1	150	245	147	132	174	59.8	129	14.9	14.6	13.0	547	47.8	96.8	13.5	50.0	11.5	2.70	11.7	1.76	10.9	2.22	6.45	0.92	5.98	0.93	3.17	1.03	35.8	14.1	2.56
MR14-E02	PC10	1	54	56	55	0.55	359	2.91	2.20	9.35	0.14	5.10	0.90	0.53	171	90.2	0.78	6.19	93.3	185	284	155	133	173	70.7	136	16.0	21.9	13.1	516	52.3	100	15.0	56.6	13.2	3.12	13.6	2.08	12.9	2.63	7.63	1.08	7.02	1.10	3.36	1.10	39.1	14.5	2.71
MR14-E02	PC10	2	4	6	85.3	0.853	460	2.96	2.07	8.85	0.26	4.90	1.15	0.51	163	80.5	0.88	5.95	106	226	311	148	121	181	110	135	15.4	28.9	12.1	402	66.5	97.3	19.4	74.8	17.8	4.40	19.6	2.98	18.9	3.91	11.4	1.60	10.4	1.61	3.21	1.01	39.6	13.7	2.67
MR14-E02	PC10	2	54	56	135.3	1.353	592	4.19	1.72	9.26	0.28	5.96	1.14	0.47	133	44.6	1.28	5.54	152	550	439	180	87.1	166	154	168	13.4	50.1	6.52	290	84.5	109	24.4	96.3	23.2	5.50	26.7	4.07	25.7	5.35	15.3	2.12	13.4	2.07	4.00	0.84	43.9	12.1	2.15
MR14-E02	PC10	3	4	6	185.6	1.856	699	4.23	1.73	9.00	0.41	5.59	1.57	0.52	149	44.5	1.46	6.15	192	378	386	175	80.2	203	184	158	11.4	87.4	5.72	301	97.3	121	29.5	117	28.8	7.19	32.6	4.97	30.9	6.36	18.2	2.53	16.0	2.47	3.81	0.64	43.4	10.9	2.80
MR14-E02	PC10	3	54	56	235.6	2.356	1715	3.18	1.91	8.46	1.14	5.54	2.88	0.48	134	43.4	1.24	5.56	153	427	364	182	87.6	258	506	158	13.5	67.6	6.49	305	256	174	77.3	312	77.1	19.1	87.2	13.0	80.5	16.3	46.0	6.20	38.2	5.77	4.00	0.77	36.7	18.2	4.27
MR14-E02	PC10	4	4	6	285.9	2.859	711	2.68	2.11	8.55	0.48	6.50	1.24	0.37	94.0	36.9	0.72	4.28	68.0	187	241	190	118	156	176	132	12.0	22.0	8.14	349	106	131	31.5	123	29.5	7.01	31.7	4.75	29.2	5.91	16.7	2.31	14.3	2.11	3.49	0.81	25.3	13.9	2.44
MR14-E02	PC10	4	54	56	335.9	3.359	603	2.57	2.15	8.58	0.39	6.51	1.03	0.38	94.9	38.8	0.58	4.36	59.3	147	219	187	124	139	145	137	13.1	15.6	8.51	334	91.3	122	26.2	100	23.8	5.61	25.7	3.84	23.9	4.88	13.9	1.93	12.0	1.77	3.53	0.87	22.7	13.6	2.24
MR14-E02	PC10	5	4	6	386.2	3.862	640	2.61	2.24	9.05	0.44	6.67	1.15	0.36	93.7	39.3	0.76	4.40	73.0	258	287	191	122	148	159	129	11.5	21.0	8.54	360	97.5	124	27.3	105	24.8	5.96	27.2	4.10	25.8	5.30	15.3	2.13	13.4	2.01	3.46	0.78	24.9	12.6	2.25
MR14-E02	PC10	5	54	56	436.2	4.362	654	2.73	2.11	8.55	0.40	6.51	1.07	0.37	98.2	41.1	0.72	4.47	64.7	192	242	187	123	156	154	134	12.1	21.3	8.57	360	98.8	128	29.3	114	27.1	6.46	29.1	4.32	26.3	5.29	14.8	2.03	12.5	1.86	3.58	0.81	25.7	13.1	2.25
MR14-E02	PC10	6	4	6	485.1	4.851	928	2.68	2.17	9.20	0.65	6.96	1.64	0.38	104	43.0	0.75	4.67	63.2	417	273	174	127	186	245	134	13.1	31.1	8.46	339	153	150	39.5	155	36.4	8.75	40.3	6.04	37.9	7.86	22.5	3.15	19.7	2.95	3.68	0.87	21.3	14.0	2.51
MR14-E02	PC10	6	54	56	535.1	5.351	658	2.78	2.37	9.18	0.39	6.70	1.05	0.40	99.8	46.5	0.50	4.80	54.4	123	223	183	127	150	144	136	14.2	11.3	8.25	330	102	133	30.8	119	28.3	6.62	29.2	4.30	25.6	5.10	14.1	1.92	11.8	1.72	3.55	0.93	20.0	13.6	2.10
MR14-E02	PC10	7	4	6	584.5	5.845	617	2.32	2.00	7.59	0.33	5.61	0.92	0.33	88.1	38.2	0.50	4.12	49.6	106	203	166	109	135	137	118	12.6	10.3	7.39	307	98.4	116	28.9	112	26.1	6.21	28.1	4.17	25.1	5.05	14.2	1.94	11.9	1.73	3.25	0.88	21.4	12.9	2.08
MR14-E02	PC10	7	54	56	634.5	6.345	514	2.45	2.05	8.41	0.30	6.38	0.84	0.35	94.1	41.1	0.59	4.38	53.6	153	220	178	116	145	108	125	12.3	13.7	8.07	332	78.4	117	23.3	89.7	21.1	5.02	21.8	3.25	19.7	3.91	10.9	1.50	9.36	1.39	3.24	0.82	21.6	12.1	1.86
MR14-E02	PC10	8	4	6	684.7	6.847	588	2.49	2.14	8.26	0.35	6.01	0.95	0.35	93.7	41.7	0.49	4.33	43.9	152	236	174	112	145	130	120	12.7	11.4	7.57	293	94.0	107	28.4	110	25.5	6.08	26.9	3.94	23.7	4.68	12.9	1.78	10.8	1.59	3.09	0.84	19.8	12.4	1.89
MR14-E02	PC10	8	54	56	734.7	7.347	813	2.34	1.97	7.76	0.65	5.79	1.65	0.33	90.1	40.3	0.56	4.15	50.6	201	256	168	103	184	210	114	11.6	16.7	7.14	309	144	115	36.5	142	32.2	7.81	36.0	5.35	33.6	7.00	20.2	2.85	17.9	2.68	3.11	0.78	20.2	12.3	2.40
MR14-E02	PC10	9	4	6	785.4	7.854	638	2.51	2.11	7.74	0.44	5.71	1.19	0.36	95.3	45.1	0.58	4.33	48.5	186	263	170	102	157	146	117	12.6	15.7	7.07	340	107	105	30.7	119	27.3	6.55	29.3	4.30	26.0	5.23	14.6	1.98	12.2	1.77	3.16	0.84	21.0	11.6	2.00
MR14-E02	PC10	9	54	56	835.4	8.354	623	2.45	2.14	8.00	0.54	5.88	1.45	0.35	95.7	39.6	0.71	4.42	62.6	260	308	175	105	168	139	122	12.6	22.4	7.25	387	104	114	29.7	115	26.2	6.22	27.7	4.03	24.5	4.93	13.7	1.90	11.8	1.75	3.26	0.83	23.9	11.7	2.04
MR14-E02	PC10	10	4	6	884.7	8.847	702	2.69	2.29	8.24	0.58	6.05	1.52	0.39	97.2	42.8	0.70	4.75	60.7	220	340	209	107	179	160	129	13.5	15.5	7.19	370	119	121	33.6	130	29.5	7.04	31.3	4.58	27.7	5.60	15.7	2.16	13.4	1.97	3.46	0.90	25.2	12.4	2.16
MR14-E02	PC10	10	54	56	934.7	9.347	1173	2.47	2.01	7.95	0.82	6.04	2.01	0.34	89.5	34.6	0.68	4.24	57.5	188	262	182	105	216	300	122	11.8	15.5	7.02	375	203	151	55.7	218	50.9	12.2	55.7	8.17	50.1	10.0	27.9	3.77	23.0	3.38	3.35	0.79	23.8	15.3	2.65
MR15-E-01	PC01	01	5	7	6	0.06	418	2.64	1.75	7.99	0.18	2.74	0.76	0.47	150	68.5	0.89	5.73	119	363	327	150	106	169	97.6	138	14.1	15.7	10.2	384	60.6	94.3	16.7	67.8	15.6	3.83	17.2	2.60	16.6	3.39	9.94	1.40	9.02	1.40	3.41	0.92	42.1	11.9	2.17
MR15-E-01	PC01	01	24	26	25	0.25	513	3.46	1.62	7.85	0.26	2.81	0.91	0.48	140	54.4	0.99	5.54	144	438	332	170	83.7	175	131	144	12.0	32.8	7.23	296	75.3	96.0	20.5	84.7	19.6	4.92	22.5	3.41	21.8	4.49	13.1	1.84	12.0	1.84	3.53	0.76	38.8	9.91	2.20
MR15-E-01	PC01	02	24	26	80.4	0.804	551	3.71	1.61	8.07	0.32	2.89	0.99	0.46	143	49.7	1.23	5.52	177	339	343	164	80.0	189	135	152	12.2	74.4	6.17	272	78.7	117	21.5	89.4	20.8	5.12	23.5	3.56	22.8	4.67	13.5	1.88	12.1	1.88	3.93	0.76	45.5	10.7	2.45
MR15-E-01	PC01	02	74	76	130.4	1.304	621	3.70	1.46	7.77	0.39	2.96	1.18	0.45	135	40.4	1.28	5.35	178	311	345	138	74.9	201	158	146	11.3	85.5	5.31	268	89.2	115	25.1	105	24.6	6.04	27.8	4.20	26.5	5.40	15.6	2.17	13.9	2.12	3.74	0.67	44.5	10.3	2.60
MR15-E-01	PC01	03	24	26	189.4	1.894	1192	3.65	1.35	7.45	0.75	2.84	1.83	0.41	135	36.0	1.28	5.21	182	267	312	129	72.6	241	344	131	10.6	78.6	4.99	275	174	132	51.8	219	52.6	13.0	59.6	8.89	56.5	11.4	32.6	4.42	27.6	4.15	3.41	0.61	45.0	14.4	3.13
MR15-E-01	PC01	03	74	76	239.4	2.394	4378	2.75	1.22	5.60	2.84	2.24	5.79	0.34	107	31.0	1.57	4.18	216	489	324	148	55.6	466	1358	14.3	8.67	71.2	3.76	246	667	306	200	828	199	49.1	227	33.6	213	43.0	122	16.4	101	15.0	0.63	0.19	43.6	29.9	7.08
MR15-E-01	PC01	04	24	26	289	2.89	1271	2.64	1.66	7.40	0.90	2.95	1.90	0.32	124	43.4	1.62	4.59	210	543	301	168	102	217	338	131	10.9	98.5	8.68	375	181	185	55.6	235	57.2	14.0	62.2	9.19	57.1	11.3	31.9	4.30	26.5	3.93	3.61	0.70	52.2	15.9	3.52
MR15-E-01	PC01	04	74	76	339	3.39	1468	2.63	1.68	7.59	1.02	3.05	2.10	0.33	125	43.3	1.54	4.69	197	507	298	169	106	233	400	138	11.3	95.8	9.07	382	214	197	64.2	272	66.1	16.1	71.7	10.7	66.1	13.1	36.7	4.96	30.4	4.53	3.77	0.73	53.1	17.9	3.88
MR15-E-01	PC01	05	24	26	389.5	3.895	1059	2.34	1.76	7.06	1.18	3.34	2.37	0.31	93.4	33.9	0.97	3.88	123	303	292	201	93.7	208	287	132	10.1	42.9	6.41	335	157	160	44.8	187	43.8	10.7	48.4	7.29	46.0	9.35	27.0	3.75	23.6	3.57	3.60	0.68	33.1	11.2	3.18
MR15-E-01	PC01	05	74	76	439.5	4.395	886	2.58	1.80	7.29	0.82	3.37	1.70	0.32	94.6	34.0	0.85	4.03	104	290	272	182	96.5	176	222	131	10.4	39.3	6.68	305	132	146	39.0	162	38.0	9.19	41.0	6.10	37.9	7.54	21.3	2.88	18.0	2.66	3.68	0.72	31.2	10.9	2.58
MR15-E-01	PC01	06	24	26	489.7	4.897	851	2.49	1.82	7.41	0.65	3.35	1.37	0.34	99.6	35.6	0.86	4.14	107	265	261	184	101	161	208	134	11.0	40.9	7.04	316	126	151	37.7	156	36.6	8.79	38.8	5.73	35.3	6.96	19.6	2.67	16.5	2.45	3.79	0.74	33.7	10.5	2.27
MR15-E-01	PC01	6	74	76	539.7	5.397	1079	2.52	1.82	7.52	0.66	3.44	1.40	0.35	105	38.0	0.96	4.22	118	368	289	179	101	175	264	140	11.5	48.3	6.99	329	164	164	50.3	209	49.3	11.8	52.2	7.74	47.0	9.10	25.2	3.32	19.9	2.86	3.93	0.77	36.3	12.3	2.41
MR15-E-01	PC01	7	24	26	589.7	5.897	1109	2.59	1.84	7.41	0.70	3.29	1.50	0.34	107	46.4	1.07	4.36	136	412	294	173	93.9	190	280	137	11.2	54.8	6.28	340	168	165	50.4	210	49.6	11.9	53.2	7.90	48.4	9.56	26.7	3.59	21.8	3.14	3.90	0.74	36.9	12.3	2.47
MR15-E-01	PC01	7	74	76	639.7	6.397	985	2.53	1.78	7.51	0.65	3.60	1.39	0.34	105	35.8	1.18	4.00	151	348	292	193	97.5	190	256	141	11.8	53.1	6.02	364	150	151	43.0	178	41.8	9.99	46.0	6.84	42.4	8.51	24.3	3.32	20.8	3.08	3.89	0.81	36.7	12.0	2.44
MR15-E-01	PC01	8	24	26	689.5	6.895	870	2.41	1.71	7.37	0.56	3.58	1.22	0.32	98.0	31.5	1.16	3.72	143	315	294	198	93.0	173	223	130	11.4	47.8	5.63	348	133	142	37.5	154	35.8	8.60	39.9	6.01	37.7	7.58	21.6	2.94	18.2	2.69	3.71	0.79	33.7	11.1	2.23
MR15-E-01	PC01	8	74	76	739.5	7.395	630	2.21	1.67	7.53	0.39	3.43	0.88	0.33	90.5	33.9	0.79	3.52	97.7	169	201	176	102	142	154	136	12.6	24.8	5.73	307	95.0	125	26.0	106	24.4	5.78	26.8	4.05	25.4	5.18	15.1	2.10	13.4	1.99	3.66	0.91	25.6	11.6	2.02
MR15-E-01	PC01	9	24	26	791	7.91	622	2.21	1.80	7.53	0.36	3.46	0.81	0.36	95.0	37.0	0.75	3.91	89.9	183	189	175	107	136	141	134	13.3	23.5	6.29	294	90.8	136	26.4	107	25.1	5.93	26.9	4.03	24.8	4.88	13.8	1.89	11.9	1.76	3.65	0.97	27.6	12.3	1.98
MR15-E-01	PC01	9	74	76	841	8.41	545	2.11	1.70	7.44	0.31	3.50	0.73	0.35	94.2	36.9	0.67	3.76	85.8	163	189	167	110	134	124	124	13.1	20.1	6.39	304	80.0	123	22.4	91.1	21.2	4.98	22.8	3.41	21.4	4.29	12.3	1.71	10.9	1.62	3.45	0.97	25.7	11.9	1.89
MR15-E-01	PC01	10	24	26	890.7	8.907	580	2.28	1.82	7.63	0.33	3.62	0.76	0.38	102	38.2	0.64	4.10	79.0	263	217	154	116	129	129	134	13.8	23.7	7.00	301	87.0	128	24.8	100	23.3	5.43	24.5	3.65	22.5	4.44	12.6	1.72	10.8	1.60	3.58	1.02	25.4	13.1	1.99
MR15-E-01	PC01	10	74	76	940.7	9.407	576	2.06	1.71	7.41	0.33	3.60	0.75	0.38	100	36.9	0.55	4.02	70.0	176	187	153	114	124	132	134	14.1	18.4	6.71	293	87.4	123	24.5	98.3	22.6	5.37	24.2	3.64	22.7	4.50	12.8	1.77	11.2	1.65	3.67	1.05	24.5	12.9	2.01
MR15-E-01	PC01	11	24	26	991.1	9.911	543	2.24	1.80	7.63	0.29	3.71	0.71	0.38	102	35.7	0.68	4.01	68.9	299	236	134	114	127	119	131	13.9	31.5	6.70	342	81.6	123	23.3	93.9	21.8	5.08	22.8	3.41	21.0	4.12	11.6	1.59	9.93	1.46	3.61	1.04	26.8	13.0	1.93
MR15-E-01	PC01	11	74	76	1041.1	10.411	643	2.16	1.76	7.49	0.41	3.57	0.92	0.36	89.4	36.0	0.52	3.97	63.2	150	217	147	107	140	158	125	13.1	12.3	6.59	298	102	119	27.3	111	25.3	5.95	27.3	4.10	25.8	5.20	15.0	2.10	13.2	1.93	3.52	0.97	23.5	12.6	1.95
MR15-E-01	PC02	01	24	26	25	0.25	515	3.54	1.50	7.39	0.27	2.88	0.91	0.45	126	41.8	1.04	5.35	152	350	316	161	79.2	173	126	156	12.3	34.0	6.06	291	73.2	107	20.4	84.7	19.7	4.83	22.4	3.36	21.4	4.36	12.7	1.77	11.3	1.74	3.92	0.80	42.9	10.6	2.03
MR15-E-01	PC02	01	74	76	75	0.75	612	3.62	1.51	7.65	0.34	2.96	1.10	0.47	137	42.4	1.25	5.54	184	308	312	153	80.0	195	153	155	11.9	73.8	5.99	306	85.6	119	24.6	103	24.4	6.03	27.3	4.09	25.9	5.27	15.2	2.10	13.5	2.08	3.90	0.72	45.0	10.8	2.34
MR15-E-01	PC02	02	24	26	116.7	1.167	943	3.82	1.37	7.43	0.56	3.01	1.54	0.44	132	36.3	1.36	5.35	203	284	315	133	70.8	225	260	140	10.2	91.6	4.41	271	134	125	40.5	172	41.3	10.3	46.8	6.99	43.9	8.85	25.3	3.45	21.7	3.30	3.53	0.59	43.4	12.0	2.65
MR15-E-01	PC02	02	74	76	166.7	1.667	1803	3.36	1.37	6.97	1.09	2.70	2.46	0.45	146	37.0	1.49	5.52	214	326	352	142	70.0	286	542	143	11.2	94.8	4.85	292	271	161	78.9	336	81.2	20.1	91.9	13.7	86.0	17.3	49.3	6.65	41.3	6.20	3.70	0.66	48.3	17.3	3.81
MR15-E-01	PC02	03	24	26	216.7	2.167	943	2.32	1.69	7.29	0.61	3.64	1.30	0.36	102	36.7	0.96	3.96	117	245	229	187	109	167	250	147	13.5	34.3	6.34	394	139	149	40.1	166	38.9	9.43	43.6	6.51	40.9	8.33	23.7	3.28	20.5	3.05	3.89	0.96	32.0	13.9	2.71
MR15-E-01	PC02	03	74	76	266.7	2.667	545	2.28	1.81	7.41	0.34	3.49	0.78	0.38	95.9	45.5	0.50	4.03	64.7	140	169	165	112	121	121	136	14.0	16.1	6.58	255	79.2	124	23.1	93.7	21.8	5.07	22.8	3.42	21.2	4.20	12.0	1.66	10.6	1.58	3.63	1.05	21.1	12.9	2.16
MR15-E-01	PC02	04	24	26	316.1	3.161	538	2.28	1.73	7.47	0.32	3.62	0.75	0.36	98.3	36.9	0.69	3.92	87.0	201	193	177	114	129	117	133	13.5	23.1	6.73	321	78.0	126	22.7	92.0	21.4	5.03	22.6	3.41	20.9	4.13	11.6	1.59	10.1	1.47	3.68	1.00	26.3	12.3	2.11
MR15-E-01	PC02	04	74	76	366.1	3.661	613	2.36	1.77	7.61	0.38	3.71	0.86	0.37	102	36.6	0.65	4.05	81.7	213	195	174	118	131	144	135	14.1	22.0	7.09	295	91.8	128	25.8	104	24.0	5.70	25.9	3.91	24.3	4.90	14.1	1.96	12.4	1.84	3.74	1.04	26.4	13.0	2.23
MR15-E-01	PC02	05	24	26	416.4	4.164	564	2.32	1.76	7.52	0.32	3.65	0.76	0.38	99.1	37.5	0.53	4.10	70.1	221	188	155	115	120	125	133	13.9	17.7	7.02	288	83.9	125	24.3	98.5	22.7	5.33	23.9	3.58	21.9	4.32	12.2	1.67	10.5	1.54	3.68	1.04	25.1	13.2	2.10
MR15-E-01	PC02	05	74	76	466.4	4.664	547	2.30	1.74	7.53	0.32	3.78	0.75	0.38	102	37.3	0.65	4.14	78.9	279	207	153	115	122	123	132	13.9	24.2	6.95	321	82.1	123	23.0	93.4	21.5	5.05	22.7	3.40	21.1	4.18	11.9	1.64	10.4	1.53	3.61	1.04	25.9	12.4	2.01
MR15-E-01	PC02	06	24	26	516.1	5.161	595	2.23	1.73	7.36	0.35	3.65	0.80	0.39	97.6	37.4	0.58	4.16	71.5	215	213	150	112	126	134	131	14.3	20.7	6.84	318	91.6	126	25.9	105	24.0	5.65	25.3	3.75	23.0	4.55	12.8	1.75	11.0	1.60	3.56	1.04	26.1	12.6	1.98
MR15-E-01	PC02	6	74	76	566.1	5.661	551	2.31	1.82	7.95	0.29	3.84	0.70	0.39	99.6	38.8	0.62	4.31	72.6	205	210	157	118	127	120	134	14.2	18.5	7.29	325	83.6	126	23.6	95.3	21.9	5.12	23.0	3.45	21.0	4.15	11.5	1.58	9.82	1.44	3.71	1.06	26.5	13.0	1.94
MR15-E-01	PC02	7	24	26	616.1	6.161	598	2.30	1.75	7.87	0.34	3.78	0.78	0.38	93.8	36.9	0.37	4.19	52.2	149	183	140	115	129	142	132	13.9	12.6	7.14	282	93.6	121	25.3	102	23.2	5.47	24.9	3.74	23.4	4.75	13.5	1.89	11.8	1.74	3.67	1.03	20.6	12.9	1.97
MR15-E-01	PC02	7	74	76	666.1	6.661	614	2.24	1.77	7.72	0.34	3.75	0.80	0.38	98.1	37.0	0.58	4.28	66.3	245	214	149	116	133	144	134	13.9	20.5	7.35	318	94.7	124	26.3	107	24.3	5.78	25.9	3.88	24.1	4.81	13.8	1.90	11.9	1.76	3.71	1.01	26.1	13.0	2.00
MR15-E-01	PC02	8	24	26	716.6	7.166	573	2.16	1.75	7.75	0.30	3.74	0.71	0.38	96.4	37.5	0.45	4.27	58.5	215	197	142	118	126	130	133	13.9	18.3	7.37	293	86.9	124	24.6	99.1	23.0	5.39	24.1	3.62	22.2	4.43	12.5	1.70	10.7	1.58	3.68	1.03	22.2	13.2	1.93
MR15-E-01	PC02	8	74	76	766.6	7.666	541	2.14	1.70	7.51	0.28	3.65	0.67	0.36	92.0	36.4	0.53	4.12	57.5	142	185	147	112	126	123	127	13.1	13.5	7.12	322	81.7	120	22.8	91.8	20.9	4.97	22.4	3.36	20.7	4.16	11.8	1.63	10.1	1.49	3.54	0.98	24.6	12.8	1.83
MR15-E-01	PC02	9	24	26	817.8	8.178	498	2.14	1.79	7.52	0.24	3.67	0.57	0.37	95.4	38.4	0.63	4.24	70.9	155	191	157	118	119	103	127	13.0	14.6	7.43	365	72.6	128	20.9	83.7	19.3	4.51	20.0	2.99	18.2	3.59	10.1	1.39	8.56	1.25	3.52	0.97	27.4	12.6	1.80
MR15-E-01	PC02	9	74	76	867.8	8.678	523	2.21	1.74	7.36	0.29	3.58	0.68	0.36	88.2	36.6	0.50	4.03	57.1	136	178	149	111	129	119	126	13.0	11.7	6.84	319	79.0	119	21.7	87.5	20.2	4.71	21.1	3.19	19.8	3.98	11.4	1.59	9.97	1.47	3.46	0.95	22.2	12.3	1.80
MR15-E-01	PC02	10	24	26	918.5	9.185	530	2.27	1.83	7.69	0.28	3.68	0.67	0.37	94.6	39.3	0.54	4.22	62.0	169	180	150	119	125	117	134	13.4	13.6	7.41	334	80.3	122	22.3	89.4	20.6	4.82	21.6	3.25	19.9	4.00	11.3	1.57	9.76	1.43	3.66	0.99	24.6	12.9	1.89
MR15-E-01	PC02	10	74	76	968.5	9.685	490	2.19	1.75	7.42	0.27	3.48	0.65	0.34	87.1	35.0	0.55	3.99	65.8	162	178	149	110	127	107	121	11.6	12.9	7.15	316	72.6	118	20.3	81.3	18.8	4.39	19.7	2.98	18.3	3.69	10.5	1.45	9.17	1.35	3.36	0.86	25.9	11.7	1.73
MR15-E-01	PC02	11	24	26	1018.6	10.186	423	2.07	1.88	7.54	0.21	3.55	0.54	0.39	90.6	38.6	0.30	4.28	21.7	71.0	137	144	120	102	89.5	141	14.3	4.31	7.22	263	64.4	104	17.8	70.8	16.1	3.73	16.7	2.49	15.4	3.09	8.78	1.22	7.74	1.14	3.70	1.04	16.7	13.1	1.84
MR15-E-01	PC03	01	24	26	25	0.25	290	2.62	1.95	8.52	0.10	2.73	0.69	0.49	161	86.2	0.56	5.86	79.6	131	212	131	124	170	49.8	122	14.3	15.3	11.9	578	43.1	92.8	11.7	45.5	10.0	2.36	9.99	1.50	9.44	1.87	5.46	0.79	5.11	0.79	3.18	1.06	36.0	12.9	2.47
MR15-E-01	PC03	02	24	26	68.3	0.683	312	2.58	1.96	8.63	0.11	2.84	0.63	0.50	167	86.8	0.58	5.96	79.5	135	217	133	130	168	54.1	126	15.1	20.8	12.6	583	45.9	98.8	12.5	48.8	10.7	2.53	10.8	1.65	10.6	2.05	6.00	0.86	5.56	0.86	3.30	1.13	39.3	13.7	2.53
MR15-E-01	PC03	02	74	76	118.3	1.183	329	2.64	1.88	8.06	0.13	2.68	0.65	0.50	163	76.9	0.65	5.85	88.6	138	238	134	120	169	62.1	131	14.9	22.8	11.7	489	48.1	95.5	13.2	52.2	11.7	2.82	12.0	1.85	11.8	2.33	6.78	0.97	6.30	0.97	3.28	1.10	39.3	13.1	2.51
MR15-E-01	PC03	03	24	26	168.5	1.685	487	2.76	1.87	8.19	0.22	2.62	0.83	0.49	169	62.5	0.94	6.20	131	257	330	149	111	190	118	148	15.1	36.1	11.7	378	70.9	100	19.8	80.8	18.4	4.60	20.6	3.14	20.1	4.12	12.2	1.71	11.0	1.71	3.68	1.00	46.0	12.4	2.60
MR15-E-01	PC03	03	74	76	218.5	2.185	552	3.09	1.58	7.71	0.26	2.71	0.86	0.45	142	40.9	0.97	5.48	141	441	370	153	86.2	170	143	146	12.7	40.2	7.79	310	80.3	95.7	22.5	93.8	21.8	5.40	24.9	3.77	24.2	4.94	14.3	1.99	12.8	1.97	3.63	0.79	41.4	10.3	2.24
MR15-E-01	PC03	04	24	26	268	2.68	679	3.59	1.45	7.85	0.41	2.97	1.16	0.45	140	31.7	1.41	5.48	199	482	380	152	75.3	198	178	151	11.7	86.1	5.30	272	96.4	119	27.7	116	27.4	6.83	31.0	4.68	29.5	6.00	17.5	2.41	15.3	2.36	3.79	0.73	47.7	10.6	2.64
MR15-E-01	PC03	04	74	76	318	3.18	1494	3.42	1.39	7.58	0.91	2.91	2.09	0.44	151	28.8	1.84	5.67	281	487	356	155	72.9	270	440	147	11.5	102	4.94	318	221	158	64.4	275	65.8	16.2	74.4	11.1	69.8	14.1	40.3	5.47	34.1	5.13	3.73	0.67	54.6	15.4	3.56
MR15-E-01	PC03	05	24	26	368.2	3.682	1409	2.40	1.70	7.48	0.93	3.51	1.87	0.34	101	26.3	1.16	4.00	145	378	295	195	93.5	222	407	137	11.8	52.9	5.83	384	223	164	60.8	253	59.1	14.4	66.2	9.92	62.4	12.7	36.3	4.92	30.6	4.53	3.80	0.82	35.5	13.8	3.20
MR15-E-01	PC03	05	74	76	418.2	4.182	1023	2.92	1.64	7.62	0.76	3.73	1.57	0.30	90.0	24.5	1.08	3.57	134	292	273	186	89.5	211	296	122	10.4	50.0	5.32	345	163	129	42.5	174	40.2	9.73	46.0	6.95	45.0	9.41	27.9	3.93	25.1	3.81	3.56	0.73	31.9	10.4	2.65
MR15-E-01	PC03	06	24	26	467.7	4.677	915	2.70	1.79	7.79	0.56	3.57	1.19	0.34	109	26.7	1.35	4.09	170	368	299	206	96.8	190	221	137	12.1	68.9	6.03	408	137	161	40.3	166	39.0	9.33	42.5	6.37	39.4	7.81	21.8	2.99	18.3	2.69	3.88	0.89	40.1	12.2	2.58
MR15-E-01	PC03	6	74	76	517.7	5.177	969	2.59	1.74	7.86	0.61	3.59	1.28	0.34	116	29.3	0.95	4.21	149	438	287	153	98.6	195	254	144	12.0	70.2	6.08	357	147	156	41.1	169	39.6	9.43	43.9	6.62	41.8	8.49	24.6	3.40	21.4	3.17	3.87	0.84	39.4	11.8	2.68
MR15-E-01	PC03	7	24	26	567.9	5.679	856	2.50	1.86	7.97	0.50	3.57	1.06	0.38	111	30.1	1.17	4.33	141	311	256	183	106	169	204	140	14.0	46.1	6.43	432	129	156	37.9	155	36.4	8.59	38.5	5.76	35.6	7.02	19.8	2.70	16.6	2.42	3.82	0.97	36.2	12.8	2.34
MR15-E-01	PC03	7	74	76	617.9	6.179	907	2.24	1.84	8.24	0.50	3.56	1.06	0.39	116	30.1	1.21	4.37	146	391	250	194	114	158	224	148	14.4	50.4	6.81	401	140	158	39.6	161	37.5	8.90	40.7	6.14	38.3	7.64	21.6	2.95	18.3	2.67	4.03	1.02	38.5	13.5	2.50
MR15-E-01	PC03	8	24	26	668.4	6.684	379	2.00	1.86	8.26	0.19	3.56	0.47	0.41	94.9	44.4	0.23	4.24	23.4	60.8	127	141	122	89.7	74.4	146	15.3	4.36	6.92	222	54.6	108	15.5	61.4	14.0	3.22	14.2	2.14	13.2	2.62	7.49	1.05	6.78	1.02	3.86	1.16	14.4	13.0	1.92
MR15-E-01	PC03	8	74	76	718.4	7.184	704	2.18	1.79	7.85	0.44	3.41	0.93	0.37	102	32.2	0.85	4.11	115	198	175	169	110	130	170	143	13.8	31.0	6.59	326	106	143	29.1	119	27.4	6.46	29.6	4.43	27.8	5.69	16.5	2.33	15.0	2.27	3.72	1.04	32.5	12.8	2.30
MR15-E-01	PC03	9	24	26	767.7	7.677	785	2.21	1.77	7.95	0.54	3.34	1.11	0.33	89.3	29.1	0.85	3.73	114	183	214	161	101	149	194	130	12.7	20.6	5.85	332	119	147	32.8	134	31.2	7.38	34.0	5.16	32.4	6.56	18.9	2.64	16.7	2.49	3.62	0.95	31.8	11.7	2.23
MR15-E-01	PC03	9	74	76	817.7	8.177	618	2.21	1.77	8.07	0.36	3.61	0.80	0.36	102	34.2	0.77	4.11	103	228	206	161	116	129	139	132	13.4	21.1	6.87	349	90.5	136	26.0	106	24.7	5.82	26.4	3.97	24.6	4.89	14.0	1.95	12.3	1.82	3.75	1.01	29.4	12.9	2.17
MR15-E-01	PC03	10	24	26	868	8.68	630	2.03	1.73	7.84	0.39	3.71	0.82	0.38	104	35.4	0.63	4.22	79.3	234	198	149	118	128	145	134	14.2	21.9	7.03	329	95.7	129	27.2	111	25.7	6.02	27.0	4.06	25.1	4.98	14.2	1.96	12.4	1.83	3.81	1.07	27.0	13.4	2.21
MR15-E-01	PC03	10	74	76	918	9.18	577	2.04	1.78	7.98	0.35	3.70	0.75	0.37	98.2	35.2	0.44	4.16	57.3	207	202	143	116	123	131	132	13.9	15.5	7.05	277	88.5	118	25.0	101	23.3	5.51	24.7	3.69	22.8	4.53	12.8	1.77	11.3	1.66	3.67	1.06	22.0	13.4	2.12
MR15-E-01	PC03	11	24	26	967.5	9.675	636	2.14	1.79	8.15	0.39	3.89	0.84	0.38	100	31.9	0.70	4.23	80.1	273	242	147	117	139	146	132	13.7	26.2	7.18	360	97.1	128	27.7	112	25.9	6.16	27.3	4.11	25.5	5.03	14.3	1.98	12.5	1.85	3.69	1.03	28.1	13.2	2.14
MR15-E-01	PC03	11	74	76	1017.5	10.175	540	2.16	1.79	7.86	0.31	3.70	0.70	0.36	92.2	31.6	0.60	4.06	72.8	192	217	147	112	126	120	127	13.1	16.3	6.75	326	81.6	118	23.3	94.3	21.5	5.10	22.7	3.40	20.8	4.11	11.5	1.60	10.1	1.47	3.45	0.99	25.6	12.4	1.93
MR15-E-01	PC04	01	24	26	25	0.25	287	2.40	1.83	7.95	0.10	2.55	0.65	0.46	153	77.5	0.54	5.58	76.6	130	207	126	118	163	50.4	115	13.7	13.9	11.3	520	43.0	88.7	11.6	45.4	10.1	2.36	10.1	1.52	9.47	1.92	5.56	0.80	5.21	0.80	2.96	1.02	35.7	12.6	2.28
MR15-E-01	PC04	01	74	76	75	0.75	312	2.45	1.83	7.98	0.11	2.62	0.63	0.47	157	76.9	0.60	5.69	80.7	132	222	128	122	165	57.8	119	14.3	20.5	11.8	533	46.4	91.4	12.7	49.9	11.2	2.66	11.4	1.75	10.9	2.20	6.36	0.92	5.93	0.91	3.07	1.06	38.5	12.9	2.37
MR15-E-01	PC04	02	24	26	108.4	1.084	366	2.58	1.85	7.94	0.15	2.61	0.71	0.51	163	73.3	0.71	5.93	99.1	147	248	132	117	176	74.1	130	15.1	25.1	11.2	442	53.5	96.7	14.9	59.7	13.6	3.28	14.1	2.17	13.5	2.77	7.98	1.14	7.41	1.14	3.25	1.10	41.1	13.0	2.51
MR15-E-01	PC04	02	74	76	158.4	1.584	467	2.74	1.85	8.03	0.20	2.53	0.79	0.49	169	63.4	0.93	6.13	130	232	319	147	110	187	110	147	15.2	38.1	11.5	377	68.3	98.5	18.8	77.5	17.7	4.34	19.6	2.99	19.2	3.95	11.5	1.62	10.6	1.62	3.68	1.01	44.3	12.1	2.60
MR15-E-01	PC04	03-1	24	26	209.8	2.098	454	2.82	1.84	7.96	0.20	2.55	0.74	0.52	164	62.1	0.80	6.09	115	215	302	149	106	185	108	155	16.1	42.5	11.2	347	67.1	95.2	18.5	75.6	17.3	4.22	19.0	2.90	18.4	3.80	11.1	1.56	10.1	1.54	3.79	1.07	40.6	11.6	2.56
MR15-E-01	PC04	03-2	24	26	263.8	2.638	614	3.47	1.54	7.67	0.30	2.79	0.99	0.45	140	43.2	1.20	5.50	172	388	347	153	81.6	185	159	144	12.5	69.1	6.63	287	88.2	109	25.0	104	24.4	6.02	27.5	4.16	26.6	5.47	15.7	2.18	13.9	2.11	3.55	0.73	45.0	10.4	2.33
MR15-E-01	PC04	04	24	26	346.8	3.468	1840	3.27	1.48	7.43	1.14	2.67	2.47	0.45	143	30.9	1.37	5.75	208	401	352	155	71.5	283	561	148	11.5	92.4	5.11	281	279	172	79.3	336	80.8	19.8	91.0	13.7	86.1	17.5	49.6	6.73	41.6	6.24	3.78	0.70	45.2	17.3	4.23
MR15-E-01	PC04	04	74	76	396.8	3.968	554	2.43	1.95	7.72	0.32	3.55	0.71	0.36	93.3	33.9	0.53	4.22	60.9	181	193	161	115	123	127	133	12.9	18.2	7.32	289	82.6	121	23.4	94.4	21.7	5.14	22.9	3.49	21.4	4.30	12.4	1.70	10.7	1.57	3.57	0.95	23.0	12.8	2.00
MR15-E-01	PC04	05	24	26	446.4	4.464	592	2.22	1.83	7.35	0.38	3.28	0.80	0.37	90.0	35.9	0.33	4.19	37.5	113	161	145	109	119	138	130	13.7	9.65	6.69	243	91.6	113	26.2	106	24.4	5.73	25.8	3.89	23.9	4.80	13.6	1.87	11.8	1.71	3.53	1.03	16.6	13.9	2.24
MR15-E-01	PC04	05	74	76	496.4	4.964	579	2.20	1.87	7.58	0.36	3.35	0.76	0.38	90.4	37.0	0.35	4.24	35.4	90.5	166	156	112	127	131	133	13.9	9.20	6.80	255	89.4	115	25.9	105	24.1	5.64	25.2	3.76	22.9	4.50	12.7	1.74	10.8	1.58	3.51	1.03	18.8	14.1	2.14
MR15-E-01	PC04	06	24	26	546.1	5.461	586	2.07	1.87	7.65	0.38	3.55	0.81	0.34	89.4	31.7	0.61	4.24	69.2	175	209	171	114	135	136	125	11.5	17.0	7.63	327	88.7	125	24.2	98.4	22.5	5.38	24.5	3.67	23.3	4.73	13.6	1.89	11.9	1.74	3.47	0.89	25.6	12.6	2.12
MR15-E-01	PC04	6	74	76	596.1	5.961	623	2.17	1.81	7.55	0.44	3.51	0.93	0.33	93.0	29.9	0.71	4.18	83.9	323	250	155	109	153	149	124	11.0	33.7	7.50	347	93.8	127	25.7	105	24.4	5.83	26.2	3.94	24.9	5.05	14.6	2.05	13.1	1.94	3.41	0.84	26.2	12.3	2.22
MR15-E-01	PC04	7	24	26	647.6	6.476	601	2.24	1.74	7.39	0.36	3.44	0.78	0.34	88.9	32.7	0.52	4.20	56.7	128	203	157	109	157	136	127	11.5	16.8	7.28	327	91.2	122	26.3	108	24.6	5.87	26.2	3.90	24.2	4.79	13.5	1.84	11.6	1.70	3.61	0.87	26.0	12.4	2.07
MR15-E-01	PC04	7	74	76	697.6	6.976	835	2.24	1.77	7.28	0.52	3.37	1.07	0.35	90.8	33.0	0.63	4.25	65.8	243	246	161	107	176	203	129	12.1	22.7	7.05	325	132	138	37.4	154	35.1	8.46	37.9	5.64	35.1	7.02	19.9	2.73	17.0	2.48	3.73	0.92	29.3	13.8	2.40
MR15-E-01	PC04	8	24	26	749.3	7.493	553	2.22	1.92	7.34	0.29	3.27	0.65	0.36	89.7	34.8	0.49	4.33	56.4	117	212	170	112	135	115	129	13.1	11.8	7.16	318	83.7	123	24.9	101	23.0	5.39	23.5	3.44	21.1	4.12	11.5	1.57	9.82	1.42	3.54	1.01	24.6	12.7	2.01
MR15-E-01	PC04	8	74	76	799.3	7.993	548	2.10	1.80	6.95	0.27	3.18	0.62	0.34	86.3	34.6	0.51	4.12	59.7	122	204	154	107	138	115	122	12.4	13.2	7.01	333	83.4	120	24.7	100	22.9	5.41	23.8	3.48	21.3	4.12	11.5	1.56	9.68	1.40	3.46	0.96	25.7	12.2	1.91
MR15-E-01	PC04	9	24	26	847.7	8.477	537	2.04	1.81	7.06	0.27	3.20	0.61	0.34	85.7	33.9	0.41	4.11	42.9	99.9	186	150	109	135	117	122	12.7	9.84	7.19	296	84.6	110	24.2	98.0	22.4	5.25	23.1	3.43	21.1	4.15	11.7	1.62	9.94	1.45	3.50	0.96	21.7	12.6	1.99
MR15-E-01	PC04	9	74	76	897.7	8.977	572	2.04	1.66	6.85	0.31	3.26	0.70	0.32	84.0	31.2	0.55	4.00	55.3	146	211	155	104	150	127	121	11.4	13.3	7.08	317	89.6	115	25.3	103	23.5	5.62	24.7	3.66	22.7	4.52	12.8	1.76	11.1	1.62	3.40	0.87	25.2	11.9	1.97
MR15-E-01	PC04	10	24	26	947.8	9.478	636	2.08	1.70	6.85	0.34	3.09	0.77	0.32	84.5	31.9	0.54	3.97	52.2	189	233	148	100	155	140	117	11.4	14.4	6.92	304	101	117	29.6	121	27.6	6.58	29.0	4.29	26.1	5.10	14.4	1.93	12.0	1.75	3.34	0.89	25.2	12.9	2.02
MR15-E-01	PC04	10	74	76	997.8	9.978	644	2.34	1.87	7.37	0.42	3.38	0.93	0.34	94.0	33.6	0.70	4.33	63.3	222	273	159	104	175	150	123	11.4	20.2	7.21	349	104	115	29.1	119	26.6	6.47	28.6	4.19	26.0	5.17	14.6	2.01	12.6	1.84	3.39	0.87	27.1	12.1	2.09
MR15-E-01	PC04	11-1	24	26	1048	10.48	659	2.36	1.95	7.35	0.45	3.34	0.99	0.35	90.9	33.6	0.61	4.37	62.1	174	277	165	103	172	151	125	12.1	14.5	7.10	363	107	120	30.2	123	27.4	6.54	28.9	4.23	26.2	5.16	14.6	1.98	12.4	1.83	3.45	0.92	26.9	12.7	2.11

Table S2 (4: continued)

Cruise	Core	Sec.	Int. [cm]	Int. [cm]	Depth [cm]	Depth [m]	ΣREY [ppm]	Na (wt.%)	Mg	Al	P	K	Ca	Ti	V (ppm)	Cr	Mn (wt.%	Fe	Co (ppm)	Ni	Cu	Zn	Rb	Sr	Y	Zr	Nb	Mo	Cs	Ba	La	Ce	Pr	Nd	Sm	Eu	Gd	Tb	Dy	Ho	Er	Tm	Yb	Lu	Hf	Ta	Pb	Th	U
MR15-E-01	PC05	01	24	26	25	0.25	582	3.46	1.64	7.88	0.31	2.91	0.96	0.48	139	52.7	0.98	5.66	143	458	335	170	87.3	179	151	147	12.3	31.0	7.41	315	84.0	100	23.9	99.1	23.2	5.74	26.3	3.99	25.7	5.27	15.4	2.15	14.0	2.15	3.62	0.79	40.4	11.0	2.36
MR15-E-01	PC05	02	24	26	71.6	0.716	317	4.62	1.13	8.14	0.13	3.80	0.51	0.31	71.5	22.6	0.58	3.55	80.0	155	235	103	72.5	106	58.6	137	12.4	35.8	3.31	189	39.4	101	12.4	49.7	11.9	2.40	12.4	1.86	11.5	2.25	6.30	0.87	5.43	0.82	4.45	1.19	33.1	16.3	1.27
MR15-E-01	PC05	02	74	76	121.6	1.216	634	3.79	1.50	7.64	0.39	2.96	1.17	0.46	137	39.9	1.34	5.50	200	286	326	144	78.7	208	156	147	11.6	93.3	5.67	285	88.4	122	26.1	109	26.0	6.38	28.8	4.34	27.2	5.50	15.8	2.21	14.0	2.14	3.82	0.70	47.5	11.1	2.65
MR15-E-01	PC05	03	24	26	172.2	1.722	1113	3.30	1.19	6.57	0.68	2.65	1.62	0.37	117	28.4	1.31	4.67	191	305	300	119	65.2	221	325	117	9.64	74.9	4.16	253	163	119	48.8	206	49.6	12.3	55.4	8.30	52.2	10.5	29.8	4.06	25.3	3.84	3.05	0.56	42.2	12.6	2.74
MR15-E-01	PC05	04	24	26	227.5	2.275	5619	2.71	1.17	5.40	3.48	2.28	6.95	0.32	107	29.5	1.81	4.09	276	604	373	151	53.7	556	1718	40.2	5.19	102	3.33	259	836	441	263	1057	255	63.3	291	43.2	272	55.0	155	20.8	128	19.0	1.52	0.18	42.9	36.2	8.34
MR15-E-01	PC05	04	74	76	277.5	2.775	6323	2.55	1.07	5.10	3.99	2.34	8.24	0.28	80.7	28.8	1.00	3.57	146	297	231	131	54.2	606	1876	17.8	4.01	52.6	3.20	228	937	479	296	1291	285	70.9	324	48.1	303	60.7	171	22.9	139	20.6	1.04	0.10	34.2	41.1	10.8
MR15-E-01	PC05	05	24	26	325.4	3.254	1013	2.65	1.90	8.75	0.65	3.64	1.44	0.39	144	56.5	1.30	5.47	203	381	250	168	135	193	246	147	12.1	76.8	11.8	386	134	208	42.2	176	43.1	10.5	45.7	6.85	42.3	8.32	23.6	3.19	19.6	2.94	3.87	0.74	52.3	19.1	3.24
MR15-E-01	PC05	05	74	76	375.4	3.754	985	2.70	1.85	8.59	0.59	3.52	1.35	0.38	139	51.7	1.45	5.46	200	366	243	168	127	194	233	144	12.2	81.5	11.3	397	131	208	41.6	173	41.9	10.2	44.4	6.65	40.8	7.98	22.4	3.01	18.5	2.73	3.79	0.74	54.6	17.4	2.95
MR15-E-01	PC05	06	24	26	425.4	4.254	1042	2.83	1.77	8.19	0.66	3.38	1.46	0.37	134	47.7	1.50	5.23	202	383	237	166	119	203	251	140	11.7	81.9	10.5	395	139	208	44.3	185	45.0	10.9	47.6	7.15	44.2	8.61	24.4	3.28	20.2	2.99	3.74	0.74	56.5	17.6	3.07
MR15-E-01	PC05	6	74	76	475.4	4.754	865	2.82	1.79	8.18	0.53	3.40	1.25	0.36	134	49.1	1.55	5.26	204	449	236	166	122	195	198	144	11.4	79.9	10.8	407	114	195	36.0	150	36.4	8.90	38.6	5.76	35.4	6.94	19.5	2.64	16.3	2.42	3.79	0.71	58.7	16.0	2.81
MR15-E-01	PC05	7	24	26	525.2	5.252	661	2.80	1.78	8.23	0.39	3.49	1.00	0.35	128	55.4	1.47	5.20	190	365	224	159	118	199	143	144	11.1	70.4	10.1	423	84.9	175	26.2	108	25.8	6.25	27.3	4.09	25.2	5.02	14.3	1.94	12.1	1.79	3.93	0.70	56.9	13.7	2.38
MR15-E-01	PC05	7	74	76	575.2	5.752	1038	2.63	1.71	8.14	0.62	3.38	1.40	0.35	128	45.8	1.53	5.07	198	433	238	169	119	202	248	144	11.5	71.3	10.2	404	138	199	45.2	189	46.5	11.3	49.4	7.35	45.1	8.80	24.5	3.27	19.9	2.91	3.78	0.72	56.1	16.3	2.86
MR15-E-01	PC05	8	24	26	624.4	6.244	1258	2.69	1.68	7.65	0.76	3.25	1.73	0.33	128	40.5	1.97	4.90	277	558	279	179	107	227	313	142	11.2	85.3	8.89	418	167	232	53.4	226	55.0	13.5	59.8	8.87	55.2	10.8	30.4	4.05	24.7	3.63	3.79	0.68	58.9	15.6	2.92
MR15-E-01	PC05	8	74	76	674.4	6.744	1483	2.45	1.71	7.68	0.94	3.26	2.04	0.38	123	45.7	1.52	4.92	180	466	249	169	115	230	387	138	11.4	64.8	9.38	391	202	221	65.4	278	68.1	16.9	74.5	11.0	68.0	13.4	37.3	4.97	30.1	4.39	3.54	0.70	56.5	18.1	3.38
MR15-E-01	PC05	9	24	26	725.8	7.258	641	2.29	1.79	7.96	0.42	3.88	0.94	0.39	106	41.2	0.60	4.44	89.6	277	219	162	121	124	141	142	14.1	23.5	7.31	292	90.4	156	26.3	107	24.8	5.83	26.3	3.94	24.5	4.86	14.0	1.93	12.2	1.82	3.78	0.96	25.7	13.7	2.17
MR15-E-01	PC05	9	74	76	775.8	7.758	656	2.32	1.81	8.25	0.42	4.05	0.97	0.41	107	40.7	0.65	4.51	85.2	284	244	163	122	132	144	146	14.6	26.1	7.38	318	95.1	155	27.2	111	25.7	6.04	27.3	4.06	25.3	5.00	14.1	1.94	12.2	1.81	3.83	0.98	27.0	13.7	2.19
MR15-E-01	PC05	10	24	26	825.3	8.253	533	2.32	1.86	8.27	0.30	3.98	0.74	0.40	97.9	39.3	0.53	4.46	69.2	164	222	165	122	122	106	141	14.4	12.3	7.31	297	75.3	145	22.1	89.1	20.6	4.75	21.2	3.13	19.1	3.72	10.6	1.44	9.00	1.33	3.66	0.98	24.5	13.4	1.93
MR15-E-01	PC05	10	74	76	875.3	8.753	642	2.35	1.85	8.10	0.38	3.85	0.89	0.39	98.3	40.6	0.59	4.51	76.3	257	242	155	118	134	141	138	14.0	18.4	7.26	311	93.8	151	27.0	109	25.2	5.91	26.6	3.96	24.4	4.85	13.7	1.87	11.7	1.71	3.63	0.93	27.0	13.5	2.02
MR15-E-01	PC05	11	24	26	925.3	9.253	609	2.25	1.84	8.01	0.35	3.85	0.82	0.39	95.1	40.3	0.57	4.42	74.0	170	213	164	116	135	133	132	13.4	13.1	7.31	311	88.5	149	25.1	101	23.2	5.44	24.7	3.70	23.0	4.58	13.0	1.77	11.0	1.62	3.65	0.91	25.5	13.1	1.94
MR15-E-01	PC05	11	74	76	975.3	9.753	573	2.41	1.95	8.16	0.31	3.97	0.76	0.40	98.3	40.6	0.61	4.58	75.5	185	214	169	122	133	122	140	13.8	13.8	7.58	331	81.5	152	22.9	92.6	21.1	5.00	22.4	3.37	21.0	4.22	12.1	1.66	10.4	1.54	3.73	0.93	24.9	13.2	1.92
MR15-E-01	PC05	12	24	26	1025.4	10.254	587	2.44	1.92	7.88	0.31	3.79	0.76	0.38	95.0	38.8	0.59	4.43	81.4	172	193	164	118	134	125	135	12.8	13.2	7.56	329	84.2	152	24.0	96.7	22.4	5.22	23.5	3.50	21.5	4.30	12.2	1.68	10.5	1.54	3.60	0.86	26.0	12.8	1.87
MR15-E-01	PC06	01	24	26	25	0.25	303	2.54	1.92	8.36	0.09	2.87	0.74	0.51	163	87.5	0.54	5.92	84.9	131	210	131	131	173	45.2	128	14.7	13.0	12.1	683	41.4	116	11.5	44.1	9.73	2.24	9.30	1.41	8.66	1.73	5.06	0.73	4.76	0.72	3.22	1.01	35.1	13.8	2.40
MR15-E-01	PC06	01	74	76	75	0.75	335	2.59	1.82	8.00	0.11	2.76	0.70	0.51	164	83.1	0.63	6.06	95.8	133	214	130	125	175	55.1	134	14.9	19.6	12.3	570	45.9	118	12.8	50.1	11.2	2.59	11.2	1.71	10.6	2.13	6.21	0.89	5.85	0.89	3.30	1.00	40.7	13.7	2.51
MR15-E-01	PC06	02	24	26	122.7	1.227	372	2.52	1.82	7.87	0.13	2.79	0.72	0.53	168	84.4	0.67	6.17	105	158	243	138	125	177	67.2	149	15.8	20.3	12.1	500	51.0	120	14.3	56.9	12.8	3.05	13.3	2.01	12.6	2.57	7.46	1.07	7.02	1.06	3.54	1.07	41.4	14.3	2.70
MR15-E-01	PC06	02	74	76	172.7	1.727	483	3.08	1.62	7.59	0.20	2.74	0.82	0.49	154	57.6	0.90	6.02	144	390	341	160	91.7	172	114	165	13.3	34.7	8.55	322	65.4	112	18.5	76.9	18.0	4.46	20.3	3.09	19.7	4.09	11.9	1.69	10.9	1.65	3.73	0.78	40.7	10.6	2.40
MR15-E-01	PC06	03	24	26	222.1	2.221	556	3.72	1.51	8.19	0.29	3.17	1.00	0.49	148	47.8	1.00	5.79	166	308	324	149	83.7	186	139	150	12.0	58.1	6.42	283	75.8	116	21.7	90.1	21.1	5.27	24.2	3.66	23.4	4.84	14.0	1.99	12.8	1.99	3.61	0.66	38.8	9.50	2.41
MR15-E-01	PC06	03	74	76	272.1	2.721	379	4.01	1.31	7.86	0.16	3.38	0.66	0.38	102	31.6	0.86	4.48	127	259	356	131	73.5	133	72.5	161	12.8	54.4	4.39	222	46.4	120	14.4	58.6	14.1	3.02	14.8	2.26	13.8	2.70	7.65	1.05	6.74	1.00	4.39	0.96	36.5	14.1	1.63
MR15-E-01	PC06	04	24	26	322	3.22	602	3.76	1.52	7.90	0.32	3.06	1.16	0.51	155	41.9	1.52	6.06	233	386	361	154	82.8	213	137	169	12.8	104	6.11	303	78.9	152	23.0	95.7	22.5	5.56	25.0	3.77	23.8	4.78	13.9	1.94	12.4	1.88	4.09	0.68	49.2	10.9	2.67
MR15-E-01	PC06	04	74	76	372	3.72	698	3.91	1.45	7.93	0.38	3.13	1.36	0.50	154	39.0	1.52	6.10	246	305	337	141	77.2	225	173	160	11.4	105	5.08	310	93.0	147	27.7	116	27.5	6.82	30.8	4.65	29.3	5.94	17.0	2.37	14.9	2.30	3.68	0.59	48.1	10.5	2.68
MR15-E-01	PC06	05	24	26	422.1	4.221	1944	3.35	1.37	6.71	1.16	2.57	2.77	0.52	174	34.7	2.71	6.11	443	703	452	178	63.6	322	569	157	13.4	138	4.25	336	286	224	82.7	351	84.3	20.9	95.8	14.4	89.9	18.2	51.5	6.98	43.1	6.47	3.68	0.62	74.3	16.4	4.29
MR15-E-01	PC06	05	74	76	472.1	4.721	893	2.64	1.77	7.55	0.72	3.62	1.61	0.33	94.3	32.6	0.87	4.15	117	289	283	193	99.8	188	227	139	10.5	39.5	6.86	321	127	170	37.1	153	35.7	8.60	38.5	5.78	36.4	7.38	21.5	3.00	19.2	2.92	3.71	0.66	30.4	10.4	2.53
MR15-E-01	PC06	06	24	26	522.5	5.225	1373	2.50	1.68	7.00	0.81	3.39	1.77	0.33	96.8	36.8	0.90	4.12	122	329	280	173	95.7	210	334	139	11.0	44.3	6.53	337	203	204	64.9	270	63.8	15.3	68.2	10.1	61.7	11.9	32.5	4.31	25.8	3.64	3.79	0.68	34.5	14.5	2.86
MR15-E-01	PC06	6	74	76	572.5	5.725	1139	2.67	1.74	7.13	0.64	3.40	1.48	0.34	104	40.0	1.09	4.35	151	451	303	177	93.4	205	270	136	11.1	57.0	6.26	341	165	196	52.3	217	51.3	12.3	54.6	8.09	49.4	9.60	26.5	3.53	21.2	3.05	3.78	0.67	36.0	12.6	2.47
MR15-E-01	PC06	7	24	26	621.8	6.218	1115	2.58	1.70	7.42	0.70	3.78	1.59	0.33	97.0	29.7	1.14	3.90	158	342	301	189	96.0	212	285	132	11.3	50.8	5.72	350	165	179	49.0	203	47.3	11.4	52.0	7.71	48.1	9.61	27.5	3.79	23.7	3.52	3.68	0.71	33.5	12.3	2.63
MR15-E-01	PC06	7	74	76	671.8	6.718	903	2.71	1.78	7.91	0.50	4.00	1.22	0.34	97.5	32.0	1.25	3.99	174	307	358	210	100	190	209	130	11.4	43.9	5.80	400	131	178	39.8	165	38.3	9.09	40.8	6.06	37.3	7.27	20.3	2.74	17.0	2.48	3.78	0.72	36.1	11.3	2.19
MR15-E-01	PC06	8	24	26	720.9	7.209	736	2.43	1.74	7.75	0.40	3.78	0.99	0.36	97.2	33.6	1.00	3.93	131	224	257	189	110	172	167	137	13.1	31.4	6.24	383	105	166	30.9	126	29.3	6.97	31.4	4.71	29.2	5.73	16.2	2.23	13.9	2.07	3.81	0.84	30.4	12.2	2.14
MR15-E-01	PC06	8	74	76	770.9	7.709	695	2.49	1.99	8.19	0.37	3.76	0.91	0.40	99.7	39.3	0.71	4.37	95.8	151	232	186	118	158	148	134	14.0	17.5	6.69	325	96.6	170	29.5	120	27.8	6.52	29.3	4.31	26.4	5.18	14.6	2.01	12.7	1.89	3.72	0.95	28.1	13.6	2.15
MR15-E-01	PC06	9	24	26	821.2	8.212	533	2.34	1.90	8.35	0.25	3.88	0.66	0.38	99.1	39.6	0.70	4.14	97.4	174	198	180	123	145	107	135	13.7	18.4	7.04	319	72.9	146	22.0	89.1	20.5	4.81	21.5	3.20	19.8	3.84	10.7	1.48	9.25	1.35	3.77	0.92	24.6	12.6	1.90
MR15-E-01	PC06	9	74	76	871.2	8.712	760	2.37	1.85	8.18	0.44	4.02	1.04	0.42	109	41.5	0.70	4.62	93.5	234	225	172	126	171	172	138	15.2	21.9	7.43	361	111	164	32.7	134	30.8	7.26	32.2	4.82	29.8	5.90	16.7	2.30	14.5	2.15	3.88	0.97	28.9	14.2	2.27
MR15-E-01	PC06	10	24	26	920.6	9.206	696	2.35	1.87	8.26	0.40	4.02	0.97	0.41	103	40.9	0.56	4.45	85.3	258	232	159	122	164	156	135	14.5	18.5	7.20	310	102	155	29.7	120	27.7	6.56	29.4	4.36	27.0	5.33	15.1	2.07	13.0	1.92	3.69	0.95	25.3	13.6	2.13
MR15-E-01	PC06	10	74	76	970.6	9.706	713	2.18	1.72	7.51	0.43	3.85	1.02	0.40	98.4	37.2	0.68	4.32	86.2	233	238	158	115	160	163	140	14.3	21.3	7.00	370	107	150	30.8	125	28.7	6.79	30.5	4.54	28.0	5.56	15.8	2.17	13.6	2.00	3.65	0.93	27.9	12.9	2.11
MR15-E-01	PC06	11	24	26	1021.6	10.216	550	2.29	1.79	7.71	0.28	3.87	0.72	0.36	89.2	35.2	0.67	4.03	82.2	177	266	162	111	145	113	125	12.5	13.6	6.70	349	78.4	142	23.2	94.0	21.5	4.99	22.5	3.31	20.4	3.98	11.1	1.51	9.42	1.38	3.36	0.85	25.1	12.0	1.79
MR15-E-01	PC07	01	24	26	25	0.25	303	2.63	1.89	8.15	0.09	2.82	0.77	0.50	164	85.4	0.55	5.99	85.1	128	208	131	129	176	46.3	126	14.5	13.4	12.4	689	41.5	114	11.6	44.6	9.88	2.28	9.71	1.45	8.99	1.79	5.23	0.76	4.95	0.76	3.19	0.97	35.3	13.8	2.39
MR15-E-01	PC07	02	24	26	96.3	0.963	318	2.54	1.90	8.49	0.10	2.98	0.68	0.51	169	86.7	0.55	6.18	82.8	123	201	132	135	173	50.3	125	14.9	22.4	12.6	587	43.7	116	12.1	47.3	10.3	2.38	10.2	1.56	9.65	1.94	5.65	0.81	5.32	0.81	3.26	0.99	37.6	13.9	2.57
MR15-E-01	PC07	02	74	76	146.3	1.463	360	2.62	1.80	7.69	0.14	2.69	0.71	0.51	164	77.0	0.65	6.05	101	139	239	132	117	173	66.3	141	14.9	24.1	11.4	451	48.9	114	13.9	55.3	12.6	2.99	13.1	1.99	12.4	2.51	7.30	1.05	6.78	1.04	3.29	1.00	39.1	13.2	2.53
MR15-E-01	PC07	03	24	26	173.1	1.731	457	2.90	1.91	8.47	0.19	2.92	0.88	0.55	176	78.3	0.84	6.60	129	193	279	148	124	199	98.2	151	15.9	29.8	12.6	431	62.5	122	17.9	72.1	16.5	4.02	17.7	2.70	17.3	3.55	10.3	1.46	9.54	1.48	3.73	1.01	43.8	13.7	2.71
MR15-E-01	PC07	03	74	76	223.1	2.231	521	3.30	1.56	8.34	0.24	3.14	0.88	0.48	149	51.0	0.93	5.92	150	370	320	158	91.1	171	131	153	12.2	36.9	7.81	325	71.1	111	19.9	82.9	19.3	4.77	22.2	3.39	21.8	4.57	13.4	1.88	12.2	1.88	3.57	0.71	38.1	9.91	2.25
MR15-E-01	PC07	04	24	26	272.4	2.724	583	3.92	1.53	7.93	0.35	3.05	1.21	0.49	151	43.0	1.38	6.10	210	330	365	155	81.8	212	139	161	12.4	89.4	6.02	289	78.2	140	22.0	91.1	21.3	5.31	24.2	3.64	23.1	4.77	13.8	1.93	12.4	1.92	3.94	0.68	46.3	10.3	2.60
MR15-E-01	PC07	04	74	76	322.4	3.224	633	3.87	1.50	7.88	0.38	3.10	1.29	0.49	147	43.9	1.40	5.96	231	282	329	146	81.8	218	149	154	11.8	91.8	5.83	294	82.9	150	24.8	102	24.5	6.00	26.8	4.07	25.5	5.15	14.9	2.07	13.2	2.02	3.92	0.64	46.0	10.9	2.57
MR15-E-01	PC07	05	24	26	372.7	3.727	1009	3.72	1.38	7.56	0.64	3.02	1.76	0.45	145	36.5	1.77	5.73	281	438	374	149	74.3	246	273	147	11.4	91.3	4.82	300	138	157	42.3	179	43.1	10.6	48.2	7.22	45.5	9.17	26.2	3.59	22.4	3.38	3.48	0.57	52.2	12.2	2.78
MR15-E-01	PC07	05	74	76	422.7	4.227	1871	2.82	1.58	7.27	1.18	3.03	2.60	0.36	125	41.2	1.82	4.94	270	547	304	174	94.7	270	527	146	12.0	87.4	7.82	347	275	227	81.4	346	83.9	20.5	93.1	13.9	86.5	17.2	48.4	6.50	39.6	5.84	3.71	0.66	56.6	18.0	3.73
MR15-E-01	PC07	06	24	26	472.5	4.725	861	2.67	1.69	7.47	0.58	3.03	1.39	0.33	141	42.7	1.76	4.84	254	505	268	162	109	187	201	145	11.1	132	9.58	419	114	187	35.6	149	36.0	8.72	38.7	5.76	35.6	7.06	19.9	2.70	16.7	2.46	3.66	0.66	56.1	13.1	2.89
MR15-E-01	PC07	6	74	76	522.5	5.225	717	3.02	1.84	8.22	0.48	3.27	1.20	0.33	139	45.4	1.76	5.12	273	536	262	182	110	205	161	165	11.3	88.2	9.52	382	92.4	159	30.6	129	30.8	7.47	32.6	4.87	29.8	5.82	16.3	2.21	13.6	2.02	3.63	0.68	53.1	12.1	2.43
MR15-E-01	PC07	7	24	26	572.5	5.725	921	2.80	1.86	8.09	0.64	3.33	1.50	0.35	141	48.0	1.89	5.13	279	558	297	196	111	230	222	165	11.0	96.2	9.15	416	122	169	40.6	171	40.7	10.1	44.3	6.62	40.6	7.96	22.4	3.00	18.5	2.73	3.64	0.69	59.8	13.4	2.74
MR15-E-01	PC07	7	74	76	622.5	6.225	1096	2.67	1.94	8.41	0.76	3.50	1.72	0.39	149	51.5	1.82	5.52	233	510	280	199	122	248	275	174	12.2	88.2	10.2	442	148	175	49.2	208	49.7	12.4	54.5	8.12	50.2	9.91	27.6	3.72	23.0	3.39	3.79	0.83	68.8	16.1	3.11
MR15-E-01	PC07	8	24	26	672.3	6.723	2667	2.87	1.72	7.79	1.71	3.26	3.56	0.36	135	41.2	2.13	4.92	275	708	353	220	102	388	769	165	11.2	85.7	7.93	412	396	244	121	523	127	31.8	140	20.9	127	24.9	69.1	9.15	55.2	8.06	3.85	0.70	76.3	24.7	4.53
MR15-E-01	PC07	8	74	76	722.3	7.223	3190	2.43	1.60	6.34	2.51	2.91	5.09	0.36	116	38.0	1.57	4.36	233	527	299	184	82.2	453	927	170	11.1	59.8	6.23	372	489	275	147	631	150	37.2	163	24.1	149	29.4	81.8	10.9	66.5	9.78	3.86	0.61	59.3	18.4	5.93
MR15-E-01	PC07	9	24	26	774.1	7.741	1740	2.62	1.76	7.32	1.73	3.27	3.63	0.34	118	36.0	1.44	4.53	238	477	333	190	91.0	330	496	174	10.7	60.7	6.81	373	243	203	79.1	338	78.8	19.2	84.9	12.6	78.4	15.7	44.0	5.91	36.3	5.36	4.06	0.60	53.1	11.5	4.13
MR15-E-01	PC07	9	74	76	824.1	8.241	1344	2.57	1.82	7.72	1.22	3.42	2.62	0.37	119	35.9	1.42	4.71	237	468	341	187	94.1	275	349	183	11.5	55.7	6.88	370	188	189	62.0	261	61.3	14.9	65.6	9.80	60.5	12.1	33.9	4.53	27.7	4.12	4.13	0.71	50.2	10.3	3.05
MR15-E-01	PC07	10	24	26	873.8	8.738	1573	2.55	1.85	7.87	1.36	3.61	2.89	0.37	115	38.4	1.30	4.67	213	438	333	187	97.7	298	454	184	12.1	44.9	7.03	359	220	190	70.5	298	69.4	17.0	75.2	11.3	69.9	14.1	40.2	5.44	33.8	5.00	4.20	0.74	47.5	10.8	3.22
MR15-E-01	PC07	10	74	76	923.8	9.238	1629	2.28	1.75	7.26	1.30	3.48	2.68	0.34	98.5	35.9	1.39	3.86	168	406	323	209	93.0	264	440	147	11.0	39.8	6.64	346	225	197	75.5	318	73.7	18.2	82.2	12.2	77.3	15.7	44.6	6.08	37.3	5.51	4.00	0.66	44.2	10.9	2.98
MR15-E-01	PC07	11	24	26	973.8	9.738	1742	2.52	1.84	7.80	1.33	3.69	2.81	0.37	114	36.1	1.29	4.53	210	412	326	177	97.1	315	537	182	11.6	42.2	6.93	373	244	181	76.3	325	75.3	18.5	82.5	12.4	78.4	16.0	45.5	6.16	38.2	5.70	4.06	0.71	48.2	10.8	2.79
MR15-E-01	PC07	11	74	76	1023.8	10.238	1751	2.52	1.78	7.34	1.32	3.49	2.83	0.33	109	33.6	1.31	4.24	222	413	314	180	91.4	318	527	174	10.4	44.3	6.43	351	249	184	78.0	331	76.7	18.8	83.7	12.5	78.2	15.9	45.4	6.28	39.1	5.84	3.80	0.60	44.1	11.2	2.84
MR15-E-01	PC08	01	24	26	25	0.25	268	2.55	1.92	8.13	0.09	2.80	0.74	0.47	167	81.7	0.52	5.86	88.3	122	207	128	126	192	43.1	146	14.0	12.4	11.8	642	38.4	87.4	11.2	43.8	9.51	2.17	9.25	1.41	8.66	1.75	5.04	0.73	4.77	0.73	3.05	0.99	35.6	13.3	2.35
MR15-E-01	PC08	02	24	26	92.5	0.925	289	2.54	1.92	8.40	0.10	2.93	0.65	0.50	178	84.9	0.57	6.26	94.3	121	213	135	132	196	46.0	161	15.1	19.9	12.7	677	41.6	94.9	12.0	46.8	10.3	2.34	10.0	1.51	9.35	1.89	5.50	0.79	5.18	0.80	3.33	1.07	40.2	14.2	2.63
MR15-E-01	PC08	02	74	76	142.5	1.425	306	2.53	1.92	8.22	0.11	2.88	0.66	0.50	175	84.7	0.58	6.10	97.1	129	225	134	130	192	52.8	164	15.3	22.8	12.5	603	44.4	94.0	12.7	50.0	10.9	2.53	10.9	1.66	10.4	2.14	6.20	0.88	5.84	0.89	3.42	1.08	40.1	14.2	2.72
MR15-E-01	PC08	03	24	26	190.7	1.907	359	2.66	1.92	8.12	0.14	2.85	0.72	0.52	183	80.2	0.70	6.42	121	147	254	143	126	207	67.6	178	16.0	26.5	12.2	500	51.5	99.3	15.0	59.7	13.4	3.18	13.9	2.11	13.3	2.69	7.76	1.13	7.30	1.12	3.70	1.11	43.6	14.4	2.84
MR15-E-01	PC08	03	74	76	240.7	2.407	355	2.74	1.93	8.12	0.14	2.84	0.73	0.53	181	81.7	0.73	6.43	122	157	264	143	125	206	68.0	181	15.9	29.1	12.4	478	50.3	97.7	14.7	59.0	12.9	3.16	13.7	2.09	13.2	2.71	7.86	1.13	7.32	1.10	3.71	1.09	43.2	14.0	2.82
MR15-E-01	PC08	04	24	26	290.6	2.906	434	2.73	1.92	8.24	0.20	2.81	0.84	0.53	187	73.9	0.91	6.55	155	187	295	148	120	217	95.3	184	16.0	36.3	12.3	416	61.9	99.4	18.2	74.1	16.6	4.10	18.1	2.76	17.5	3.58	10.4	1.49	9.66	1.45	3.69	1.02	46.3	13.3	2.76
MR15-E-01	PC08	04	74	76	340.6	3.406	456	2.89	1.96	8.22	0.20	2.72	0.83	0.51	193	78.9	1.00	6.81	175	248	350	160	116	221	102	191	16.3	44.8	12.2	440	64.5	100	19.0	78.2	17.8	4.39	19.5	2.97	19.0	3.91	11.3	1.60	10.4	1.58	3.86	1.01	47.6	12.7	2.79
MR15-E-01	PC08	05	24	26	390.3	3.903	463	2.56	1.85	7.98	0.20	2.65	0.81	0.52	187	74.4	0.92	6.63	157	222	339	156	113	218	105	193	16.4	45.1	12.2	401	66.3	97.8	19.5	80.1	17.9	4.45	19.8	3.02	19.6	4.04	11.7	1.66	10.8	1.63	3.87	1.02	46.3	12.8	2.80
MR15-E-01	PC08	05	74	76	440.3	4.403	476	2.89	1.94	8.62	0.23	2.93	0.88	0.56	188	76.4	0.87	6.79	148	191	301	158	120	234	108	205	17.9	49.0	12.5	381	68.6	102	20.2	82.8	18.3	4.53	20.0	3.04	19.3	3.97	11.5	1.62	10.4	1.62	4.05	1.09	43.9	12.9	2.77
MR15-E-01	PC08	06	24	26	490.9	4.909	572	3.11	1.81	8.38	0.28	2.91	1.01	0.55	177	62.4	0.98	6.73	179	224	309	159	103	235	143	208	15.7	53.7	9.86	351	80.6	101	24.0	100	22.9	5.72	25.8	3.93	25.6	5.26	15.3	2.18	14.0	2.16	4.15	0.93	44.3	12.1	2.77
MR15-E-01	PC08	6	74	76	540.9	5.409	462	3.31	1.53	8.18	0.23	2.99	0.89	0.47	158	46.8	0.93	6.03	167	248	291	145	85.7	202	116	182	11.4	52.2	6.87	285	62.7	89.2	18.4	77.7	18.1	4.56	20.7	3.15	20.4	4.24	12.3	1.76	11.3	1.75	3.64	0.67	39.2	9.34	2.29
MR15-E-01	PC08	7	24	26	591.1	5.911	565	3.83	1.57	8.54	0.33	3.25	1.11	0.49	162	47.0	1.09	6.26	199	264	365	146	86.5	226	140	190	12.3	70.9	6.21	285	78.8	101	23.9	100.0	23.4	5.79	26.5	3.97	25.3	5.16	14.7	2.09	13.4	2.08	3.85	0.72	42.5	10.5	2.62
MR15-E-01	PC08	7	74	76	641.1	6.411	626	3.90	1.65	8.96	0.40	3.45	1.32	0.52	170	46.8	1.52	6.60	261	371	401	166	92.4	254	153	207	13.1	95.5	6.61	316	85.2	125	25.8	107	25.3	6.19	28.1	4.19	26.6	5.40	15.6	2.21	14.0	2.17	4.12	0.71	50.6	11.7	2.86
MR15-E-01	PC08	8	24	26	690.4	6.904	1008	3.84	1.50	8.65	0.65	3.50	1.80	0.48	164	41.6	1.63	6.36	290	388	393	156	86.9	286	271	190	12.6	94.1	5.65	326	139	134	44.8	190	45.6	11.1	50.3	7.59	47.4	9.61	27.1	3.72	23.4	3.57	3.90	0.64	52.9	14.0	3.29
MR15-E-01	PC08	8	74	76	740.4	7.404	2188	2.64	1.76	7.73	1.48	3.65	3.10	0.45	133	40.2	1.49	5.38	264	435	346	185	97.4	326	661	187	14.8	67.5	5.93	305	332	187	97.6	416	99.5	24.3	111	16.4	103	20.5	57.5	7.77	47.6	7.06	3.86	0.84	43.6	20.6	4.42
MR15-E-01	PC08	9	24	26	790	7.9	513	2.30	2.02	8.62	0.35	4.22	0.84	0.37	97.8	36.2	0.65	4.51	87.3	180	268	210	122	139	112	167	13.0	18.3	7.39	316	72.1	123	21.8	88.0	20.3	4.76	21.1	3.16	19.5	3.89	11.1	1.56	9.85	1.46	3.63	0.88	25.7	12.9	1.98
MR15-E-01	PC08	9	74	76	840	8.4	506	2.41	2.11	8.31	0.33	3.87	0.80	0.39	106	39.1	0.59	4.72	84.1	207	215	182	123	141	109	173	13.5	18.7	7.85	308	72.8	120	21.9	87.5	19.9	4.68	20.8	3.11	19.4	3.88	11.0	1.55	9.69	1.43	3.70	0.89	25.8	13.1	2.02
MR15-E-01	PC08	10	24	26	890.2	8.902	542	2.30	1.98	8.12	0.34	3.76	0.83	0.36	100	37.7	0.61	4.56	85.7	143	225	179	115	160	115	157	12.2	16.9	7.67	328	78.0	124	24.1	97.3	22.4	5.27	23.1	3.46	21.2	4.20	11.7	1.60	9.84	1.42	3.49	0.80	26.6	12.5	1.96
MR15-E-01	PC08	10	74	76	940.2	9.402	505	2.22	2.01	7.94	0.33	3.69	0.81	0.39	106	39.4	0.62	4.70	88.0	172	210	172	119	148	105	166	13.8	17.1	7.58	335	71.2	120	22.4	90.8	21.0	4.93	21.4	3.21	19.5	3.82	10.7	1.47	9.27	1.35	3.60	0.93	25.2	13.9	2.24
MR15-E-01	PC08	11	24	26	990.8	9.908	587	2.39	2.11	8.24	0.42	3.89	0.98	0.37	100	37.6	0.67	4.69	85.2	235	279	182	121	167	133	167	12.6	16.1	8.15	354	86.7	126	25.2	102	23.3	5.51	24.3	3.69	23.2	4.66	13.4	1.89	12.1	1.79	3.60	0.83	24.7	13.1	2.19
MR15-E-01	PC08	11	74	76	1040.8	10.408	599	2.44	1.83	8.08	0.43	4.10	1.02	0.33	95.5	35.2	0.67	4.41	85.9	322	258	160	114	194	144	155	11.0	21.7	7.77	314	91.6	116	25.5	103	23.2	5.52	25.0	3.80	24.0	4.94	14.3	2.02	12.9	1.92	3.38	0.73	23.4	11.9	2.08
MR15-E-01	PC09	01	24	26	25	0.25	267	2.77	2.01	8.47	0.10	2.96	0.80	0.49	173	84.1	0.52	6.10	89.9	127	212	135	127	200	42.5	144	14.2	12.5	12.0	808	38.7	88.7	11.2	43.1	9.54	2.14	9.02	1.39	8.40	1.68	4.86	0.70	4.60	0.71	2.93	0.97	35.2	13.2	2.31
MR15-E-01	PC09	02	24	26	58.7	0.587	281	2.78	2.05	8.90	0.10	3.06	0.78	0.52	183	87.1	0.58	6.44	96.1	129	216	137	133	206	45.0	157	15.0	15.1	12.9	685	40.8	92.6	11.7	45.3	10.0	2.30	9.51	1.44	8.98	1.80	5.16	0.75	4.92	0.75	3.20	1.02	37.4	13.7	2.45
MR15-E-01	PC09	02	74	76	108.7	1.087	290	2.70	1.97	8.78	0.11	3.06	0.74	0.52	181	83.2	0.63	6.41	100	123	226	135	131	211	49.3	162	14.9	21.1	12.5	654	42.0	92.5	12.0	46.8	10.2	2.38	9.95	1.52	9.42	1.88	5.51	0.78	5.17	0.80	3.26	0.97	38.7	12.8	2.38
MR15-E-01	PC09	03	24	26	157.7	1.577	276	2.47	1.91	8.38	0.10	2.99	0.63	0.50	178	84.8	0.57	6.19	91.8	127	209	133	130	191	45.8	159	14.9	22.8	12.4	598	40.5	88.9	11.5	44.1	9.71	2.20	9.40	1.43	8.86	1.78	5.13	0.75	4.94	0.76	3.17	0.99	36.4	12.5	2.34
MR15-E-01	PC09	03	74	76	207.7	2.077	313	2.58	1.95	8.51	0.12	3.02	0.70	0.52	183	84.4	0.64	6.43	105	142	251	139	133	203	55.8	169	16.0	24.9	13.0	647	45.4	94.6	13.0	50.9	11.2	2.61	11.2	1.69	10.6	2.17	6.28	0.91	5.99	0.90	3.45	1.08	39.7	13.8	2.64
MR15-E-01	PC09	04	24	26	257.6	2.576	343	2.71	2.02	8.72	0.14	3.07	0.75	0.56	193	87.7	0.71	6.85	119	153	275	148	135	216	61.7	183	16.8	26.1	13.3	569	49.6	99.8	14.4	56.5	12.6	2.95	12.8	1.94	12.1	2.44	7.11	1.02	6.69	1.03	3.71	1.14	44.0	14.5	2.82
MR15-E-01	PC09	04	74	76	307.6	3.076	340	2.52	1.97	8.71	0.15	3.12	0.73	0.56	192	83.6	0.69	6.73	117	152	264	149	135	211	63.1	176	16.5	24.5	13.0	542	48.9	97.4	14.2	56.4	12.7	2.98	12.7	1.91	11.9	2.41	6.91	0.99	6.45	1.00	3.50	1.07	40.6	13.6	2.63
MR15-E-01	PC09	05	24	26	358	3.58	349	2.69	1.95	8.54	0.15	3.08	0.75	0.55	186	80.6	0.73	6.72	123	160	276	148	134	216	66.2	177	16.4	27.8	12.8	497	50.0	96.9	14.6	58.3	12.8	3.10	13.2	2.02	12.5	2.54	7.36	1.06	6.86	1.04	3.54	1.10	41.6	13.9	2.66
MR15-E-01	PC09	05	74	76	408	4.08	350	2.57	1.90	8.47	0.15	3.01	0.74	0.53	184	76.2	0.73	6.49	125	165	266	146	129	207	69.6	170	15.7	28.2	12.4	453	50.0	92.9	14.7	58.9	13.4	3.12	13.5	2.03	12.9	2.62	7.54	1.08	6.97	1.06	3.34	1.01	40.2	13.0	2.55
MR15-E-01	PC09	06	24	26	456	4.56	444	2.73	1.91	8.67	0.22	2.92	0.88	0.53	192	78.0	0.95	6.83	155	203	323	152	123	229	102	188	16.0	35.4	12.6	431	63.5	97.7	18.5	75.2	17.0	4.13	18.2	2.81	17.9	3.63	10.7	1.53	9.84	1.52	3.64	0.97	45.1	12.8	2.58
MR15-E-01	PC09	6	74	76	506	5.06	419	2.75	1.87	8.14	0.21	2.72	0.80	0.50	186	75.6	0.98	6.57	167	222	341	148	111	216	95.6	177	15.2	38.6	11.5	425	59.3	92.2	17.4	71.5	16.1	3.91	17.6	2.66	16.8	3.46	10.0	1.42	9.18	1.41	3.39	0.88	43.1	11.3	2.41
MR15-E-01	PC09	7	24	26	557.8	5.578	432	2.90	1.91	8.32	0.21	2.74	0.84	0.51	188	75.1	0.96	6.68	169	220	338	154	111	222	99.4	183	15.3	39.8	11.1	420	60.9	92.7	18.1	74.3	16.8	4.13	18.2	2.78	17.9	3.63	10.5	1.49	9.60	1.49	3.58	0.90	43.5	11.6	2.51
MR15-E-01	PC09	7	74	76	607.8	6.078	434	2.86	1.89	8.51	0.21	2.79	0.85	0.52	183	74.0	0.86	6.66	146	158	312	146	112	229	99.3	188	15.7	39.9	11.8	375	62.6	92.4	18.3	75.1	16.9	4.12	18.3	2.78	17.7	3.65	10.6	1.51	9.78	1.51	3.71	0.94	42.3	12.0	2.60
MR15-E-01	PC09	8	24	26	657.7	6.577	445	2.94	1.91	8.38	0.21	2.76	0.85	0.55	185	72.0	0.87	6.82	150	153	328	152	110	230	102	198	16.3	41.8	11.8	364	63.8	95.7	18.5	75.8	17.3	4.23	18.7	2.84	18.3	3.77	10.8	1.56	10.1	1.55	3.96	0.98	43.7	12.0	2.61
MR15-E-01	PC09	8	74	76	707.7	7.077	443	2.68	1.81	8.09	0.22	2.76	0.82	0.52	176	73.1	0.83	6.41	140	166	291	145	110	222	99.6	186	16.7	39.2	11.8	354	64.0	94.6	18.9	77.3	17.4	4.28	18.6	2.85	18.1	3.67	10.6	1.52	9.82	1.51	3.72	1.03	41.5	12.2	2.54
MR15-E-01	PC09	9	24	26	758	7.58	500	2.83	1.80	8.05	0.24	2.68	0.90	0.54	175	66.9	0.87	6.56	148	178	303	145	102	232	118	193	16.7	41.6	10.7	342	72.7	95.6	21.4	88.4	20.2	4.97	21.9	3.33	21.5	4.37	12.6	1.80	11.6	1.78	3.92	0.99	42.4	12.2	2.59
MR15-E-01	PC09	9	74	76	808	8.08	512	3.12	1.72	7.62	0.25	2.64	0.92	0.51	154	57.3	0.82	6.10	130	192	330	137	89.0	186	129	156	14.4	40.8	8.48	305	72.4	94.2	21.8	83.6	20.8	5.20	23.7	3.60	23.2	4.76	14.0	1.96	12.5	1.92	3.76	0.86	38.5	11.1	2.46
MR15-E-01	PC09	10	24	26	857.6	8.576	534	3.34	1.84	8.88	0.28	2.97	1.08	0.53	165	56.3	0.89	6.51	138	197	342	145	98.4	218	141	164	14.9	41.3	9.27	330	74.5	98.9	22.3	82.8	21.4	5.35	23.9	3.71	23.7	4.92	14.4	2.01	13.0	2.03	3.98	0.82	38.3	11.0	2.48
MR15-E-01	PC09	10	74	76	907.6	9.076	552	3.28	1.74	8.37	0.29	2.89	0.98	0.50	157	54.3	0.90	6.19	139	211	345	136	93.5	197	145	156	14.0	41.3	8.83	300	76.0	99.5	23.0	87.0	22.6	5.60	25.6	3.93	25.0	5.23	15.3	2.16	13.8	2.13	3.91	0.82	39.0	11.0	2.51
MR15-E-01	PC09	11	24	26	957.4	9.574	463	3.23	1.56	7.28	0.25	2.40	0.91	0.47	148	47.3	0.89	5.84	142	191	331	126	71.6	179	120	142	11.5	44.3	6.10	249	63.3	88.2	18.6	72.7	18.2	4.60	21.2	3.19	20.6	4.30	12.4	1.78	11.3	1.72	3.36	0.64	36.1	8.53	2.16
MR15-E-01	PC09	11	74	76	1007.4	10.074	421	3.43	1.56	7.92	0.22	2.80	0.86	0.47	144	49.9	0.90	5.81	140	189	341	126	77.1	178	103	137	11.0	44.6	5.94	265	56.3	87.5	17.1	68.8	16.7	4.20	18.9	2.87	18.2	3.76	10.9	1.53	9.82	1.50	3.38	0.63	37.0	8.68	2.03

Table S2 (5: continued)

Cruise	Core	Sec.	Int. [cm]	Int. [cm]	Depth [cm]	Depth [m]	ΣREY [ppm]	Na (wt.%)	Mg	Al	P	K	Ca	Ti	V (ppm)	Cr	Mn (wt.%	Fe	Co (ppm)	Ni	Cu	Zn	Rb	Sr	Y	Zr	Nb	Mo	Cs	Ba	La	Ce	Pr	Nd	Sm	Eu	Gd	Tb	Dy	Ho	Er	Tm	Yb	Lu	Hf	Ta	Pb	Th	U
MR15-E-01	PC10	01	24	26	25	0.25	485	3.56	1.65	8.19	0.27	3.02	0.96	0.50	150	52.5	0.96	6.11	146	404	370	159	81.9	179	121	147	11.7	40.9	6.69	278	67.1	89.2	20.1	83.9	19.5	4.85	22.1	3.34	21.3	4.40	12.8	1.80	11.6	1.79	3.44	0.68	37.7	9.08	2.12
MR15-E-01	PC10	02	24	26	64.4	0.644	503	3.87	1.53	8.05	0.33	3.07	1.08	0.46	148	40.1	1.25	5.92	174	214	378	136	74.5	197	124	153	11.5	87.7	5.27	263	68.6	102	20.3	85.0	19.7	4.88	22.2	3.32	21.1	4.36	12.6	1.75	11.4	1.72	3.64	0.63	41.2	8.98	2.17
MR15-E-01	PC10	02	74	76	114.4	1.144	850	3.96	1.44	7.96	0.59	3.16	1.58	0.44	145	37.0	1.25	5.77	175	310	384	130	73.3	224	234	134	10.3	74.8	4.86	270	117	110	37.6	159	37.7	9.30	42.2	6.29	39.6	8.12	23.1	3.15	19.9	3.00	3.26	0.52	41.3	11.3	2.58
MR15-E-01	PC10	03	24	26	165.6	1.656	3511	3.01	1.46	6.60	2.32	2.55	4.62	0.40	128	35.6	1.74	5.13	252	570	414	160	61.1	419	1093	16.3	11.8	74.6	4.33	271	531	289	152	651	156	38.1	176	26.2	166	33.6	95.8	12.8	79.5	11.8	0.60	0.24	45.6	26.0	5.49
MR15-E-01	PC10	03	74	76	215.6	2.156	807	2.82	1.79	8.08	0.54	3.26	1.22	0.34	129	44.5	1.48	5.17	182	533	308	166	112	175	198	136	11.1	81.9	9.83	369	106	145	35.6	148	36.0	8.68	38.4	5.74	35.6	7.05	20.0	2.70	16.7	2.44	3.63	0.70	54.0	13.8	2.57
MR15-E-01	PC10	04	24	26	264.4	2.644	679	2.89	1.84	8.07	0.44	3.25	1.04	0.34	133	46.0	1.62	5.31	201	627	298	170	114	166	152	137	11.4	81.1	9.81	380	89.0	144	29.9	123	30.1	7.21	31.7	4.71	28.8	5.62	15.8	2.14	13.2	1.93	3.68	0.71	53.4	13.2	2.48
MR15-E-01	PC10	05	24	26	315.8	3.158	651	3.32	1.89	8.11	0.41	3.24	1.01	0.34	134	44.4	1.63	5.30	210	506	273	169	114	166	146	136	11.4	80.9	9.79	382	86.6	141	28.7	116	28.3	6.82	29.9	4.42	27.1	5.32	14.9	2.01	12.4	1.77	3.64	0.68	52.1	12.4	2.33
MR15-E-01	PC10	05	74	76	365.8	3.658	645	3.04	1.86	7.95	0.40	3.17	0.98	0.33	134	47.0	1.58	5.28	207	471	264	164	114	161	143	133	11.2	78.3	9.90	375	85.2	140	28.3	114	28.5	6.81	30.0	4.44	27.3	5.37	15.0	2.02	12.4	1.80	3.61	0.68	54.0	12.6	2.36
MR15-E-01	PC10	06	24	26	409.1	4.091	645	2.87	1.85	7.99	0.41	3.19	1.00	0.33	138	46.5	1.74	5.22	226	531	273	169	116	164	145	135	11.0	81.0	9.96	387	84.4	144	27.9	111	28.1	6.70	29.6	4.35	27.1	5.30	15.0	2.03	12.6	1.86	3.56	0.67	55.2	12.4	2.35
MR15-E-01	PC10	6	74	76	459.1	4.591	624	2.75	1.79	7.91	0.36	3.11	0.90	0.32	135	45.3	1.75	5.19	234	539	277	169	111	158	136	135	11.1	77.8	9.54	388	81.2	146	26.8	107	26.7	6.45	28.6	4.21	25.8	5.10	14.3	1.96	12.0	1.75	3.63	0.68	55.1	12.2	2.18
MR15-E-01	PC10	7	24	26	509	5.09	597	2.78	1.79	7.93	0.32	3.08	0.82	0.32	133	42.3	1.82	5.19	241	558	285	170	109	159	125	137	11.1	75.6	9.23	390	75.6	150	25.3	102	25.3	6.05	26.7	3.96	24.2	4.74	13.2	1.80	11.1	1.60	3.67	0.68	55.0	11.8	2.02
MR15-E-01	PC10	7	74	76	559	5.59	673	2.81	1.83	8.08	0.39	3.21	0.97	0.33	135	42.4	1.92	5.27	249	588	303	174	109	173	147	139	11.2	74.8	9.12	402	85.4	163	28.3	114	28.3	6.84	29.9	4.47	27.4	5.42	15.2	2.06	12.7	1.86	3.71	0.71	58.5	12.7	2.08
MR15-E-01	PC10	8	24	26	609.1	6.091	652	2.54	1.83	8.12	0.39	3.29	0.94	0.35	136	44.1	1.85	5.28	230	603	312	181	115	164	143	134	10.7	70.7	9.38	409	83.4	156	27.5	112	27.4	6.64	29.0	4.33	26.5	5.29	14.8	2.01	12.4	1.83	3.54	0.69	58.0	11.8	2.08
MR15-E-01	PC10	8	74	76	659.1	6.591	747	2.72	1.91	8.16	0.48	3.29	1.12	0.40	134	56.9	1.72	5.51	208	581	308	182	114	175	173	138	12.1	62.4	9.28	400	98.3	152	32.6	135	32.5	7.99	34.8	5.21	32.0	6.34	17.6	2.39	14.8	2.16	3.69	0.78	58.1	12.6	2.23
MR15-E-01	PC10	9	24	26	710.6	7.106	776	2.62	1.87	8.19	0.48	3.34	1.13	0.37	138	49.3	1.72	5.47	181	540	287	175	119	179	185	135	11.7	58.7	9.78	405	103	150	33.7	141	33.9	8.29	36.4	5.41	33.5	6.67	18.8	2.54	15.7	2.31	3.51	0.77	63.1	13.5	2.22
MR15-E-01	PC10	9	74	76	760.6	7.606	1026	2.28	1.68	7.05	0.61	2.92	1.33	0.35	121	50.6	1.30	4.83	148	450	248	159	105	172	255	120	10.7	45.7	8.61	344	138	151	47.2	198	48.4	12.0	53.9	7.95	48.8	9.64	27.0	3.64	22.2	3.16	3.22	0.74	52.6	15.7	2.45
MR15-E-01	PC10	10	24	26	810	8.1	936	2.36	1.76	7.49	0.55	3.09	1.22	0.35	130	49.7	1.46	4.95	168	512	271	167	110	169	232	125	10.4	49.9	9.13	363	124	155	42.3	174	43.3	10.7	47.3	7.00	43.1	8.53	23.8	3.21	19.6	2.82	3.23	0.71	57.5	15.2	2.40
MR15-E-01	PC10	10	74	76	860	8.6	1052	2.52	1.71	7.41	0.65	3.05	1.43	0.34	129	40.7	1.82	4.86	227	632	328	177	97.9	199	266	127	10.3	58.4	7.95	384	141	166	48.3	195	49.1	12.2	54.0	7.97	48.8	9.66	26.9	3.57	21.8	3.12	3.35	0.66	63.6	14.5	2.34
MR15-E-01	PC10	11	24	26	910.7	9.107	2598	2.79	1.86	9.26	1.65	3.71	3.25	0.42	137	49.0	1.88	5.59	201	633	329	201	118	337	762	146	11.9	51.3	9.27	414	384	280	118	472	123	30.5	131	19.8	121	23.6	65.2	8.56	51.7	7.68	3.83	0.74	68.9	26.4	3.53
MR15-E-01	PC10	11	74	76	960.7	9.607	4242	2.53	1.70	7.61	2.89	3.14	5.41	0.42	123	46.9	2.05	5.12	206	754	355	205	94.0	458	1296	145	12.2	47.4	7.32	382	640	340	223	764	202	50.4	219	33.0	203	40.4	112	14.9	90.6	13.5	3.82	0.75	66.9	33.2	5.31
MR15-E-01	PC10	12	24	26	1009.2	10.092	5416	2.10	1.62	6.00	3.88	2.64	7.07	0.35	103	47.0	1.74	4.23	162	666	298	191	82.5	541	1554	29.9	10.8	32.4	6.30	354	797	381	290	1157	261	64.7	282	41.8	256	50.0	138	18.1	109	16.0	0.94	0.35	60.9	37.1	6.87
MR15-E-01	PC11	01	24	26	25	0.25	401	2.79	1.75	7.99	0.21	2.80	0.80	0.46	154	67.8	0.84	6.17	116	365	368	163	104	172	92.7	143	13.9	18.3	9.98	376	55.8	86.6	17.2	64.4	16.4	4.04	17.8	2.74	17.4	3.56	10.4	1.46	9.50	1.46	3.59	0.87	42.5	11.9	2.21
MR15-E-01	PC11	02	24	26	94.8	0.948	492	3.48	1.55	7.86	0.30	2.91	0.96	0.47	148	46.8	0.99	5.86	150	288	370	150	78.3	182	122	142	11.3	60.4	6.00	255	68.8	90.9	21.1	81.4	20.5	5.14	23.1	3.52	22.3	4.57	13.2	1.84	11.8	1.84	3.50	0.66	38.1	9.32	2.24
MR15-E-01	PC11	02	74	76	144.8	1.448	504	3.54	1.48	7.72	0.31	2.86	0.93	0.43	145	43.0	1.19	5.62	173	291	364	144	75.8	184	122	144	11.2	77.1	5.59	259	69.2	102	21.0	82.5	20.3	4.99	22.9	3.46	22.0	4.49	12.9	1.82	11.6	1.78	3.61	0.63	42.0	9.36	2.19
MR15-E-01	PC11	03	24	26	195.3	1.953	499	3.74	1.41	7.85	0.32	3.09	0.99	0.43	136	36.6	1.10	5.46	153	198	355	127	75.2	186	122	148	11.2	76.9	4.91	255	68.9	97.8	21.1	83.3	20.2	4.97	22.9	3.46	22.0	4.49	12.9	1.81	11.5	1.76	3.66	0.69	40.1	9.90	2.07
MR15-E-01	PC11	03	74	76	245.3	2.453	680	3.64	1.39	7.78	0.45	3.11	1.28	0.44	136	39.0	1.25	5.67	186	257	362	130	75.2	210	175	139	10.5	77.9	4.81	266	91.5	108	29.9	123	30.1	7.36	33.1	5.01	31.5	6.33	18.1	2.49	15.8	2.43	3.43	0.55	41.4	10.4	2.33
MR15-E-01	PC11	04	24	26	294.7	2.947	1532	3.49	1.39	7.23	1.05	2.71	2.29	0.49	156	35.5	1.34	6.05	187	291	372	140	67.3	275	443	112	11.2	79.2	4.52	262	238	140	68.4	290	69.3	17.2	78.1	11.7	73.6	14.9	42.2	5.71	35.3	5.34	2.68	0.46	46.1	14.7	3.41
MR15-E-01	PC11	04	74	76	344.7	3.447	4173	3.07	1.40	6.21	2.87	2.47	5.47	0.39	123	32.1	1.58	4.95	227	505	379	155	60.2	478	1320	27.5	11.4	69.6	3.96	259	632	317	181	782	184	45.5	208	31.1	197	39.9	113	15.3	93.8	14.1	0.63	0.27	41.6	28.8	6.34
MR15-E-01	PC11	05	24	26	395.1	3.951	4209	2.42	1.37	6.18	2.88	2.70	5.45	0.34	117	36.5	1.87	4.59	253	655	379	168	78.0	465	1297	26.6	11.0	70.6	5.84	302	638	339	185	797	189	46.6	212	31.5	199	40.0	113	15.3	93.4	14.0	0.68	0.28	49.5	30.9	6.59
MR15-E-01	PC11	05	74	76	445.1	4.451	997	2.46	1.72	7.93	0.68	3.27	1.45	0.35	133	45.5	1.35	5.14	168	436	267	155	121	193	244	137	11.4	74.0	10.5	374	132	163	45.4	193	45.8	11.2	48.9	7.32	45.3	8.97	25.2	3.41	21.0	3.13	3.69	0.72	53.4	17.7	3.09
MR15-E-01	PC11	06	24	26	495.1	4.951	932	2.55	1.73	7.97	0.65	3.24	1.40	0.35	135	47.6	1.48	5.29	178	411	264	160	118	196	221	137	11.2	76.8	10.5	391	123	163	42.3	179	43.1	10.5	45.4	6.77	41.9	8.21	23.0	3.14	19.2	2.87	3.72	0.73	57.0	17.4	3.03
MR15-E-01	PC11	6	74	76	545.1	5.451	899	2.64	1.77	8.17	0.59	3.40	1.29	0.36	136	49.6	1.51	5.38	177	437	257	162	126	195	210	139	11.3	73.0	11.1	405	118	163	40.8	173	41.6	10.1	43.1	6.47	39.8	7.79	21.7	2.95	18.0	2.68	3.68	0.72	57.8	17.2	2.86
MR15-E-01	PC11	7	24	26	594.3	5.943	700	2.83	1.87	8.74	0.46	3.63	1.08	0.36	142	56.6	1.58	5.69	185	422	254	172	134	197	157	146	11.4	74.4	11.6	437	89.8	153	30.3	127	30.3	7.34	31.2	4.71	29.0	5.78	16.3	2.22	13.9	2.09	3.90	0.72	60.3	14.8	2.56
MR15-E-01	PC11	7	74	76	644.3	6.443	727	2.82	1.84	8.83	0.46	3.68	1.08	0.36	138	53.5	1.53	5.59	171	416	258	172	133	199	167	146	11.6	67.0	11.4	437	94.3	147	32.3	135	32.2	7.79	33.1	5.02	30.8	6.10	17.1	2.33	14.2	2.15	3.86	0.72	58.1	14.8	2.45
MR15-E-01	PC11	8	24	26	694.6	6.946	929	2.82	1.87	8.88	0.58	3.62	1.28	0.37	140	49.9	1.63	5.61	186	474	274	177	130	206	222	147	12.2	68.5	11.3	426	121	162	42.4	180	43.1	10.5	44.5	6.69	41.2	8.01	22.5	3.02	18.4	2.76	3.91	0.76	58.0	16.0	2.63
MR15-E-01	PC11	8	74	76	744.6	7.446	719	2.51	1.69	7.64	0.42	2.86	0.97	0.30	124	41.1	1.47	4.81	193	468	256	151	98.5	160	161	122	10.5	61.0	8.34	342	93.5	135	32.8	139	33.5	8.10	35.8	5.35	32.3	6.34	17.7	2.36	14.4	2.06	3.41	0.65	50.4	13.0	2.22
MR15-E-01	PC11	9	24	26	795.9	7.959	844	2.76	1.89	8.67	0.51	3.57	1.20	0.36	149	48.3	1.99	5.61	245	573	297	185	129	203	196	154	12.3	75.4	11.2	450	109	168	37.4	158	37.5	9.05	38.7	5.84	35.9	7.04	19.8	2.66	16.4	2.48	3.99	0.72	60.3	14.1	2.47
MR15-E-01	PC11	9	74	76	845.9	8.459	786	2.88	1.91	8.76	0.48	3.58	1.16	0.35	149	47.6	2.06	5.61	253	597	311	186	125	202	179	150	11.8	75.6	10.5	451	100	173	33.7	141	33.5	8.11	35.0	5.29	32.7	6.46	18.2	2.47	15.2	2.28	3.88	0.70	59.2	13.4	2.29
MR15-E-01	PC11	10	24	26	895.7	8.957	801	2.65	1.90	8.44	0.51	3.34	1.17	0.37	139	49.0	1.82	5.36	225	598	321	177	112	187	183	136	11.5	68.0	9.28	412	102	161	35.0	149	35.4	8.77	38.0	5.70	35.1	6.91	19.4	2.62	16.2	2.36	3.60	0.74	58.9	13.1	2.29
MR15-E-01	PC11	10	74	76	945.7	9.457	1190	2.53	1.90	8.44	0.77	3.53	1.66	0.40	143	52.4	1.79	5.61	185	580	318	184	125	227	303	145	12.6	62.6	10.4	436	158	174	54.6	233	56.0	13.8	59.7	8.95	55.1	10.9	30.2	4.06	24.6	3.63	3.68	0.80	62.6	16.4	2.67
MR15-E-01	PC11	11	24	26	996	9.96	2332	2.34	1.74	7.38	1.95	3.33	3.76	0.36	116	38.8	1.75	4.68	207	718	419	188	90.8	328	666	145	11.6	49.7	6.85	384	346	253	104	448	107	26.4	116	17.2	106	21.0	58.8	7.90	48.0	7.12	3.88	0.69	56.5	16.2	4.11
MR15-E-01	PC11	11	74	76	1046	10.46	1452	2.22	1.74	7.49	1.32	3.52	2.62	0.35	110	35.5	1.24	4.54	169	487	373	172	96.3	248	396	146	11.5	38.9	6.98	362	203	177	66.9	282	65.5	16.2	71.6	10.8	67.7	13.7	39.2	5.33	32.9	4.87	3.93	0.70	46.3	10.5	3.09
MR15-E01	PC12	01	24	26	25	0.25	304	2.50	2.23	9.38	0.10	2.71	0.94	0.53	172	84.1	0.64	5.45	80.9	142	216	107	132	179	50.5	121	15.7	13.8	12.8	589	46.9	100	11.8	48.4	10.2	2.35	9.78	1.48	9.19	1.77	5.17	0.76	4.86	0.76	3.32	0.94	36.9	13.0	2.39
MR15-E01	PC12	01	74	76	75	0.75	323	2.59	2.21	9.40	0.11	2.79	0.75	0.54	170	86.2	0.64	5.27	78.3	142	222	106	136	178	57.5	123	16.4	23.4	12.8	600	49.1	102	12.7	51.5	10.9	2.50	10.8	1.62	9.90	1.93	5.73	0.81	5.34	0.82	3.15	0.97	38.0	13.1	2.42
MR15-E01	PC12	02	24	26	119.7	1.197	354	2.56	2.26	9.37	0.14	2.82	0.79	0.60	173	82.8	0.76	5.40	91.4	160	250	111	132	185	67.4	133	17.3	26.0	12.8	586	53.7	105	13.8	57.0	12.0	2.89	12.1	1.85	11.5	2.28	6.64	0.95	6.18	0.96	3.37	1.00	39.8	13.3	2.47
MR15-E01	PC12	02	74	76	169.7	1.697	393	2.63	2.22	9.13	0.17	2.75	0.88	0.61	170	81.6	0.81	5.28	102	181	266	112	128	186	81.5	138	17.2	29.6	12.3	455	59.4	104	15.4	64.1	13.8	3.31	14.5	2.17	13.7	2.69	8.06	1.15	7.46	1.12	3.53	1.00	40.3	13.3	2.53
MR15-E01	PC12	03	24	26	219.9	2.199	516	2.76	2.20	9.18	0.23	2.64	1.14	0.61	182	75.5	1.07	5.62	132	242	319	119	123	208	124	152	17.3	40.5	12.7	441	77.9	110	20.1	87.7	18.6	4.58	20.3	3.06	19.6	3.90	11.7	1.68	10.7	1.63	3.81	0.92	45.7	12.5	2.62
MR15-E01	PC12	03	74	76	269.9	2.699	520	2.83	2.21	8.97	0.24	2.57	1.04	0.66	184	70.0	1.12	5.70	146	272	360	125	114	205	126	150	17.6	49.7	11.7	422	77.8	109	20.2	89.0	18.6	4.64	20.9	3.18	20.1	4.04	12.0	1.69	10.7	1.66	3.81	0.89	44.9	11.8	2.55
MR15-E01	PC12	04	24	26	319.2	3.192	522	3.07	2.21	8.91	0.25	2.61	1.13	0.70	182	76.5	1.01	5.87	130	199	338	127	114	207	126	163	18.5	53.0	12.1	383	80.8	109	20.0	88.3	18.5	4.63	20.9	3.16	20.0	4.02	12.0	1.69	11.0	1.69	4.11	0.98	44.7	12.1	2.68
MR15-E01	PC12	04	74	76	369.2	3.692	494	2.77	2.14	8.72	0.24	2.64	1.01	0.72	176	73.6	0.95	5.65	121	214	307	122	117	198	116	157	19.2	50.4	12.2	375	76.3	107	19.2	84.3	17.8	4.32	19.5	2.97	18.3	3.71	11.0	1.53	9.99	1.51	4.03	1.03	41.9	12.0	2.61
MR15-E01	PC12	05	24	26	420	4.2	590	2.89	1.92	8.86	0.29	2.79	1.23	0.65	157	58.4	0.93	5.19	126	206	289	112	98.5	196	159	152	15.4	47.7	9.17	325	88.1	99.2	22.7	102	21.8	5.52	25.4	3.89	24.4	4.99	15.0	2.09	13.6	2.09	3.81	0.77	36.2	10.6	2.35
MR15-E01	PC12	05	74	76	470	4.7	498	3.42	1.72	8.77	0.27	2.85	1.07	0.60	149	48.5	1.03	4.95	133	315	325	112	85.4	176	131	137	12.7	56.5	6.47	262	73.6	95.0	18.7	82.8	18.1	4.52	20.6	3.19	20.0	4.10	12.1	1.71	10.9	1.67	3.48	0.63	36.6	8.36	2.10
MR15-E01	PC12	06	24	26	519.1	5.191	1142	2.91	1.62	8.02	0.82	2.98	1.95	0.54	129	28.1	1.38	4.45	172	561	393	118	80.4	230	321	145	12.2	62.7	5.95	304	172	143	47.2	216	47.9	11.6	54.3	8.02	50.6	9.96	28.7	3.87	24.1	3.64	3.67	0.58	39.9	12.9	2.92
MR15-E01	PC12	06	74	76	569.1	5.691	1041	2.45	1.96	7.86	0.97	3.20	1.86	0.44	97.4	25.4	1.00	3.44	109	299	293	142	98.6	204	268	131	11.7	41.7	6.95	333	161	159	44.0	198	43.8	10.4	47.3	7.04	43.7	8.35	24.1	3.26	20.1	2.93	3.63	0.63	31.2	10.5	2.58
MR15-E01	PC12	07	24	26	619.1	6.191	1011	2.51	2.01	7.93	0.69	3.23	1.42	0.41	106	29.0	1.06	3.65	118	317	290	147	107	181	251	135	12.3	50.1	7.42	351	157	161	43.6	195	43.7	10.0	46.1	6.83	42.6	8.07	22.6	3.01	18.3	2.62	3.79	0.66	34.8	10.9	2.27
MR15-E01	PC12	07	74	76	669.1	6.691	1099	2.54	1.90	7.98	0.76	3.54	1.59	0.41	99.5	22.3	1.50	3.36	158	384	343	165	94.1	206	286	129	11.9	60.5	5.73	381	171	167	45.7	206	45.6	10.9	50.3	7.40	47.0	8.97	25.5	3.40	20.9	3.04	3.58	0.62	36.8	10.7	2.18
MR15-E01	PC12	08	24	26	718.1	7.181	1007	2.50	1.88	7.93	0.70	3.47	1.57	0.38	99.7	26.7	1.37	3.40	162	373	325	155	102	198	258	137	12.8	57.2	5.91	381	154	163	42.3	192	41.7	9.78	44.7	6.54	40.1	7.82	21.9	3.01	18.8	2.77	3.71	0.69	35.4	11.0	2.22
MR15-E01	PC12	08	74	76	768.1	7.681	907	2.42	1.94	8.23	0.63	3.38	1.33	0.41	118	25.1	1.49	3.64	152	419	304	164	117	191	224	147	15.7	61.9	6.64	451	137	171	36.9	165	35.8	8.48	38.3	5.78	35.5	7.04	19.8	2.73	16.9	2.51	3.91	0.83	38.1	12.1	2.24
MR15-E01	PC12	09	24	26	819.7	8.197	919	2.38	1.91	8.45	0.68	3.52	1.51	0.37	95.9	26.5	1.08	3.52	129	245	255	149	109	199	236	130	13.9	32.9	6.07	374	141	162	36.4	166	35.5	8.43	38.8	5.91	36.7	7.36	21.0	2.87	18.1	2.70	3.71	0.80	32.6	11.8	2.26
MR15-E01	PC12	09	74	76	869.7	8.697	712	2.17	1.97	8.67	0.55	3.51	1.16	0.40	104	32.6	0.80	3.79	94.2	246	226	136	124	149	172	137	16.1	23.9	7.13	357	110	143	28.5	128	27.0	6.25	28.4	4.31	26.7	5.30	15.1	2.09	13.2	1.99	3.91	0.94	29.3	12.9	2.15
MR15-E01	PC12	10	24	26	920.5	9.205	730	2.13	1.99	8.43	0.56	3.59	1.16	0.42	105	36.7	0.69	4.00	77.8	257	224	129	126	149	175	138	17.3	24.1	7.16	339	116	138	30.1	136	27.9	6.55	29.9	4.47	27.8	5.40	15.3	2.08	13.0	1.92	3.94	0.99	26.6	13.1	2.16
MR15-E01	PC12	10	74	76	970.5	9.705	614	2.14	1.98	8.71	0.36	3.71	0.85	0.40	97.8	35.1	0.69	3.93	80.8	273	227	121	124	131	136	134	16.0	20.7	7.35	346	93.4	138	25.4	112	23.4	5.47	24.7	3.69	22.0	4.29	11.9	1.62	10.0	1.47	3.94	0.96	26.2	13.0	1.97
MR15-E01	PC12	11	24	26	1019.7	10.197	602	2.14	1.92	8.69	0.42	3.58	0.82	0.38	96.7	37.5	0.64	3.95	64.4	220	213	122	123	126	139	132	15.7	17.3	7.22	345	93.7	128	24.5	110	22.8	5.29	24.0	3.58	21.7	4.25	12.1	1.63	10.3	1.52	3.77	0.94	26.5	13.0	1.98
MR15-E01	PC13	01	24	26	25	0.25	340	2.72	2.19	10.5	0.14	2.98	0.90	0.58	176	91.2	0.65	5.80	84.1	147	240	112	143	187	57.5	131	17.6	16.2	13.8	735	52.3	109	13.4	55.7	11.1	2.57	10.9	1.66	10.2	2.01	5.89	0.84	5.55	0.83	3.50	1.07	40.1	14.3	2.55
MR15-E01	PC13	01	53	55	54	0.54	317	2.55	2.07	9.97	0.12	2.81	0.82	0.54	169	83.5	0.64	5.51	80.6	140	221	108	136	177	53.9	123	16.0	17.5	12.9	656	49.1	102	12.4	51.1	10.4	2.43	10.4	1.59	9.68	1.90	5.60	0.79	5.17	0.79	3.19	0.96	37.4	13.0	2.38
MR15-E01	PC13	02	24	26	81.7	0.817	335	2.54	2.03	9.53	0.14	2.78	0.77	0.56	169	82.3	0.71	5.41	86.9	140	241	107	133	178	58.8	126	16.2	23.4	12.8	628	51.0	105	13.0	54.9	11.1	2.65	11.0	1.71	10.4	2.08	6.07	0.85	5.58	0.86	3.36	0.97	41.6	13.1	2.48
MR15-E01	PC13	02	74	76	131.7	1.317	335	2.43	2.11	9.63	0.11	2.99	0.82	0.59	172	88.6	0.65	5.33	76.8	177	248	112	145	172	57.8	131	17.6	26.4	13.1	707	50.8	107	12.9	54.8	11.1	2.56	10.8	1.66	10.2	2.00	5.91	0.83	5.44	0.85	3.46	1.05	39.2	13.8	2.62
MR15-E01	PC13	03	24	26	182	1.82	365	2.49	2.20	9.79	0.14	3.07	0.87	0.67	176	87.9	0.75	5.47	91.7	163	269	118	144	179	65.1	140	18.6	24.9	13.4	664	56.0	112	14.2	60.3	12.3	2.88	12.2	1.88	11.3	2.30	6.77	0.95	6.26	0.97	3.73	1.10	41.3	14.3	2.70
MR15-E01	PC13	03	74	76	232	2.32	413	2.55	2.18	9.61	0.15	2.95	1.03	0.67	182	85.0	0.84	5.42	106	175	290	122	142	193	81.3	147	18.8	29.5	13.3	542	62.0	116	15.7	69.1	14.3	3.39	14.4	2.18	13.5	2.76	8.03	1.14	7.53	1.11	3.69	1.08	43.9	14.1	2.78
MR15-E01	PC13	04	24	26	282.5	2.825	386	2.55	2.11	9.07	0.16	2.81	0.94	0.65	170	78.6	0.78	5.11	98.1	172	279	117	134	186	77.2	144	17.6	29.3	12.4	513	58.4	106	14.9	64.3	13.3	3.19	13.9	2.11	13.0	2.63	7.64	1.10	7.07	1.10	3.62	1.02	39.9	13.2	2.60
MR15-E01	PC13	04	74	76	332.5	3.325	421	2.53	2.11	9.26	0.21	2.82	1.00	0.63	176	75.0	0.83	5.26	108	176	280	117	132	189	87.7	140	17.9	31.9	12.8	509	63.5	110	16.3	71.1	14.6	3.55	15.4	2.38	14.6	2.99	8.65	1.23	7.82	1.20	3.61	1.04	42.8	13.5	2.66
MR15-E01	PC13	05	24	26	382.3	3.823	501	2.61	2.07	9.27	0.24	2.64	1.08	0.62	182	77.7	1.10	5.44	138	249	345	124	121	201	117	151	17.8	40.7	12.7	481	75.2	113	19.4	85.3	17.8	4.27	19.5	2.97	18.3	3.81	11.2	1.58	9.95	1.56	3.92	0.94	46.2	12.3	2.70
MR15-E01	PC13	05	74	76	432.3	4.323	566	3.10	2.37	10.4	0.27	2.83	1.12	0.69	205	84.1	1.24	5.98	162	286	403	141	127	227	132	170	20.5	50.9	13.2	520	85.7	127	22.0	95.3	20.4	4.91	22.4	3.43	21.3	4.39	12.7	1.79	11.3	1.78	4.53	1.08	51.9	13.4	3.05
MR15-E01	PC13	06	24	26	481.8	4.818	515	2.72	2.11	9.25	0.26	2.64	1.02	0.64	184	74.8	0.94	5.36	129	204	331	124	116	208	121	160	18.8	43.0	12.2	466	79.0	112	20.3	87.4	18.6	4.51	20.8	3.14	19.3	4.04	11.7	1.64	10.5	1.64	4.07	1.00	43.8	12.4	2.74
MR15-E01	PC13	06	74	76	531.8	5.318	511	2.68	2.08	8.94	0.26	2.59	1.02	0.70	180	69.6	0.94	5.46	128	169	327	126	115	204	121	161	18.7	45.3	12.4	418	78.7	111	19.8	86.7	18.1	4.38	19.7	3.03	19.1	3.92	11.6	1.62	10.3	1.60	4.12	1.00	44.0	11.9	2.73
MR15-E01	PC13	07	24	26	581.8	5.818	494	2.61	2.05	8.91	0.25	2.65	0.91	0.71	178	73.9	0.91	5.35	120	196	331	123	118	203	114	155	18.8	44.0	12.2	393	76.4	109	19.4	85.2	17.8	4.32	19.3	2.95	18.3	3.76	10.8	1.55	9.83	1.51	4.04	1.03	43.2	11.9	2.65
MR15-E01	PC13	07	74	76	631.8	6.318	573	2.73	2.04	8.93	0.29	2.67	1.04	0.74	177	67.6	0.93	5.39	129	203	333	127	111	208	141	155	19.7	46.6	11.4	368	87.9	112	22.7	101	21.2	5.03	23.4	3.53	22.1	4.49	13.3	1.85	11.8	1.84	3.96	0.97	42.1	11.8	2.64
MR15-E01	PC13	08	24	26	681.5	6.815	627	2.91	2.01	9.09	0.33	2.82	1.17	0.81	164	56.6	1.01	5.41	146	217	328	126	104	211	166	158	17.1	48.4	9.21	343	95.1	110	24.4	108	23.2	5.72	26.5	3.99	25.4	5.18	15.4	2.14	13.7	2.12	3.92	0.86	41.2	10.9	2.58
MR15-E01	PC13	08	74	76	731.5	7.315	580	3.02	1.88	8.86	0.29	2.77	1.09	0.76	155	56.1	0.91	5.32	133	197	318	119	101	217	151	154	15.8	45.1	9.44	331	87.3	106	22.4	99.6	21.8	5.36	24.4	3.71	23.5	4.74	14.2	1.98	12.7	1.94	4.01	0.80	37.6	10.9	2.48
MR15-E01	PC13	09	24	26	781.3	7.813	596	3.04	1.80	8.68	0.30	2.69	1.16	0.69	152	55.2	0.92	5.13	140	199	326	117	95.5	196	157	155	16.1	45.1	8.84	315	87.7	109	22.8	102	22.1	5.45	25.1	3.86	24.3	4.99	14.5	2.02	13.1	2.03	4.10	0.80	38.6	10.4	2.56
MR15-E01	PC13	09	74	76	831.3	8.313	612	3.10	1.65	8.81	0.33	2.72	1.24	0.61	148	51.9	0.98	5.04	144	198	332	108	92.1	200	166	149	14.8	46.3	7.98	301	89.6	105	23.3	105	23.1	5.68	26.4	4.04	25.3	5.23	15.4	2.18	13.9	2.14	3.93	0.73	38.7	10.1	2.50
MR15-E01	PC13	10	24	26	881.1	8.811	535	3.28	1.69	8.69	0.31	2.56	1.17	0.60	148	54.9	1.04	5.28	153	187	342	106	76.3	195	138	145	12.9	51.7	6.39	280	77.5	103	20.0	90.7	19.6	4.93	22.4	3.47	21.9	4.43	13.0	1.84	11.8	1.79	3.79	0.64	38.9	8.80	2.34
MR15-E01	PC13	10	74	76	931.1	9.311	461	3.43	1.46	8.38	0.26	2.66	1.08	0.56	143	48.8	1.00	4.90	144	171	330	101	76.2	178	118	134	11.8	52.6	5.83	267	67.2	93.3	17.0	77.0	16.6	4.15	18.5	2.91	18.5	3.81	11.0	1.57	9.91	1.52	3.54	0.62	37.8	8.42	2.21
MR15-E01	PC13	11	24	26	981.4	9.814	512	3.43	1.43	8.53	0.28	2.86	1.14	0.52	140	48.3	1.11	4.87	156	222	341	98.8	79.0	178	129	136	12.9	50.6	6.20	268	75.5	99.2	19.5	88.7	18.8	4.70	21.3	3.29	20.8	4.17	12.3	1.73	11.0	1.72	3.53	0.63	38.5	8.43	2.22
MR15-E01	PC13	11	74	76	1031.4	10.314	582	3.34	1.48	8.44	0.34	2.73	1.33	0.55	139	50.2	1.12	4.76	161	249	362	102	78.8	183	147	138	12.7	50.4	6.22	267	87.3	106	22.8	104	22.2	5.44	24.9	3.82	23.5	4.77	13.8	1.91	12.1	1.90	3.61	0.64	39.1	9.39	2.24

Table S2 (6: continued)

Cruise	Core	Sec.	Int. [cm]	Int. [cm]	Depth [cm]	Depth [m]	ΣREY [ppm]	Na (wt.%)	Mg	Al	P	K	Ca	Ti	V (ppm)	Cr	Mn (wt.%	Fe	Co (ppm)	Ni	Cu	Zn	Rb	Sr	Y	Zr	Nb	Mo	Cs	Ba	La	Ce	Pr	Nd	Sm	Eu	Gd	Tb	Dy	Ho	Er	Tm	Yb	Lu	Hf	Ta	Pb	Th	U
MR15-02	PC01	1	4	6	5	0.05	281	2.84	1.94	7.99	0.11	2.46	0.85	0.47	156	80.1	0.57	5.70	87.6	140	228	138	118	172	46.5	121	14.0	9.01	10.9	540	39.9	92.7	11.3	44.1	9.77	2.29	9.80	1.48	9.14	1.81	5.34	0.77	5.04	0.76	3.14	0.95	39.3	13.4	2.29
MR15-02	PC01	1	14	16	15	0.15	277	2.69	2.00	8.57	0.11	2.81	0.87	0.49	173	84.3	0.57	6.07	93.7	130	216	154	124	206	48.6	151	14.0	11.4	11.7	573	40.8	88.4	11.6	40.3	10.1	2.34	9.89	1.50	9.37	1.86	5.47	0.78	5.10	0.80	3.09	0.95	35.5	13.2	2.30
MR15-02	PC01	1	24	26	25	0.25	280	2.53	1.91	8.36	0.11	2.57	0.74	0.48	156	80.8	0.54	5.79	77.2	121	207	127	122	168	47.4	124	13.9	12.7	11.6	570	40.7	89.7	11.4	44.2	9.83	2.24	9.66	1.48	9.10	1.86	5.38	0.78	5.14	0.79	3.23	0.99	35.5	13.3	2.40
MR15-02	PC01	2	4	6	31.9	0.319	283	2.57	1.97	8.44	0.11	2.81	0.75	0.49	174	83.3	0.58	6.13	95.8	126	206	130	125	202	49.6	153	14.4	15.1	12.0	552	41.8	89.5	11.9	40.9	10.3	2.37	10.3	1.56	9.77	1.96	5.74	0.82	5.54	0.84	3.20	1.01	38.0	13.6	2.46
MR15-02	PC01	2	14	16	41.9	0.419	288	2.73	2.00	8.62	0.10	2.94	0.70	0.51	176	84.6	0.58	6.24	93.8	126	207	132	130	204	48.8	158	14.8	15.8	12.5	608	42.1	94.7	12.0	41.5	10.4	2.40	10.2	1.55	9.52	1.91	5.64	0.80	5.33	0.81	3.31	1.04	39.1	14.2	2.54
MR15-02	PC01	2	24	26	51.9	0.519	298	2.56	1.91	8.35	0.11	2.66	0.64	0.49	157	81.1	0.56	5.88	78.3	123	209	128	127	165	51.4	125	14.6	18.1	12.2	552	43.4	93.1	12.2	47.3	10.5	2.43	10.6	1.60	9.91	2.01	5.86	0.84	5.61	0.85	3.32	1.03	38.3	13.7	2.49
MR15-02	PC01	2	34	36	61.9	0.619	310	2.68	2.01	8.68	0.12	2.98	0.73	0.51	177	86.7	0.57	6.22	92.4	128	216	133	133	204	56.8	158	15.2	18.7	12.5	606	45.8	93.4	13.2	45.9	11.5	2.70	11.6	1.77	11.0	2.20	6.49	0.92	6.01	0.93	3.25	1.06	38.4	14.3	2.55
MR15-02	PC01	2	44	46	71.9	0.719	314	2.60	1.93	8.28	0.13	2.83	0.73	0.50	173	80.3	0.62	6.12	97.8	128	221	131	125	202	60.7	151	14.6	21.3	12.1	554	46.4	89.7	13.3	46.8	11.8	2.77	12.0	1.82	11.5	2.31	6.80	0.97	6.31	0.97	3.12	0.99	39.3	13.4	2.42
MR15-02	PC01	2	54	56	81.9	0.819	312	2.51	1.93	8.28	0.13	2.86	0.71	0.51	172	79.4	0.63	6.10	103	138	222	134	127	202	59.3	158	15.1	19.4	12.1	546	45.5	91.5	13.2	46.5	11.7	2.75	11.8	1.82	11.2	2.26	6.59	0.94	6.11	0.94	3.23	1.02	38.1	13.8	2.45
MR15-02	PC01	2	64	66	91.9	0.919	332	2.57	1.94	8.29	0.14	2.84	0.76	0.52	178	74.2	0.68	6.29	109	147	233	138	124	209	65.7	161	15.1	20.5	12.0	482	48.6	92.1	14.1	49.7	12.6	3.03	13.1	2.01	12.5	2.51	7.33	1.04	6.85	1.05	3.29	1.02	40.0	13.6	2.49
MR15-02	PC01	2	74	76	101.9	1.019	378	2.65	1.94	8.48	0.17	2.68	0.78	0.53	167	78.7	0.70	6.29	103	155	262	141	123	183	77.9	141	15.8	22.6	11.9	458	54.3	98.1	15.6	61.6	14.2	3.42	15.0	2.27	14.4	2.91	8.41	1.20	7.85	1.21	3.55	1.08	42.1	13.8	2.63
MR15-02	PC01	2	84	86	111.9	1.119	380	2.80	1.95	8.28	0.19	2.84	0.86	0.53	181	79.1	0.74	6.41	121	156	259	139	122	221	81.7	172	15.7	25.5	11.9	455	55.3	95.1	16.2	57.3	14.8	3.55	15.5	2.40	15.0	3.03	8.93	1.25	8.20	1.27	3.46	1.03	41.1	13.5	2.56
MR15-02	PC01	2	94	96	121.9	1.219	413	2.73	1.90	8.21	0.23	2.81	0.93	0.51	173	64.3	0.73	6.19	124	157	260	137	118	220	94.9	173	15.6	26.9	11.4	415	60.5	93.0	17.6	63.5	16.4	3.96	17.7	2.70	17.2	3.50	10.3	1.47	9.42	1.45	3.45	1.03	40.2	13.6	2.58
MR15-02	PC01	3	4	6	132.4	1.324	417	2.75	1.91	8.32	0.20	2.81	0.88	0.52	181	61.5	0.82	6.45	136	173	281	142	119	226	96.1	182	15.5	31.0	11.9	418	60.7	94.9	17.6	63.1	16.2	3.94	17.8	2.71	17.2	3.51	10.4	1.49	9.58	1.47	3.66	1.01	43.0	13.5	2.60
MR15-02	PC01	3	14	16	142.4	1.424	454	2.79	1.89	8.19	0.22	2.69	0.90	0.50	178	57.1	0.90	6.44	149	228	313	149	112	226	111	184	15.3	33.7	11.4	382	66.1	93.3	19.2	69.9	18.0	4.46	19.9	3.06	19.6	4.01	11.8	1.66	10.8	1.67	3.71	0.95	43.6	12.6	2.53
MR15-02	PC01	3	24	26	152.4	1.524	475	2.94	1.96	8.57	0.21	2.60	0.85	0.52	171	61.2	0.86	6.56	132	233	340	155	113	196	112	159	16.3	36.9	11.9	392	68.6	98.6	19.7	79.4	18.5	4.58	20.3	3.12	20.0	4.09	12.0	1.69	11.0	1.69	4.00	1.01	45.2	12.8	2.64
MR15-02	PC01	3	34	36	162.4	1.624	449	2.83	1.90	8.34	0.21	2.77	0.85	0.52	180	54.8	0.84	6.58	142	237	313	155	111	226	107	197	16.3	35.0	11.7	385	65.6	93.8	19.3	70.1	18.0	4.44	19.8	3.02	19.2	3.93	11.5	1.62	10.4	1.62	3.93	1.00	42.4	12.3	2.58
MR15-02	PC01	3	44	46	172.4	1.724	451	2.74	1.85	8.18	0.21	2.76	0.85	0.51	176	55.0	0.85	6.50	145	232	304	156	109	223	107	197	16.1	36.4	11.3	377	65.6	93.6	19.4	70.0	18.2	4.51	20.2	3.06	19.5	3.98	11.6	1.63	10.6	1.63	3.93	0.99	42.8	12.2	2.55
MR15-02	PC01	3	54	56	182.4	1.824	504	3.16	1.95	9.03	0.24	3.05	0.95	0.54	183	49.6	0.89	6.95	153	259	324	162	112	238	124	208	16.7	39.4	11.3	374	72.5	97.1	21.8	79.4	20.7	5.12	23.2	3.52	22.6	4.63	13.4	1.91	12.4	1.91	4.21	1.02	44.1	12.7	2.67
MR15-02	PC01	3	64	66	192.4	1.924	511	3.19	1.81	8.59	0.26	2.96	0.95	0.51	170	44.9	0.89	6.55	156	280	330	157	100	226	131	200	14.9	41.9	9.60	332	73.0	91.9	21.8	80.2	21.2	5.22	23.9	3.62	23.4	4.79	14.0	1.98	12.9	1.99	3.99	0.88	41.8	11.4	2.48
MR15-02	PC01	3	74	76	202.4	2.024	504	3.20	1.77	8.53	0.24	2.75	0.89	0.50	155	47.0	0.88	6.25	137	319	349	161	95.0	182	126	159	13.9	40.6	8.92	324	70.7	93.8	20.6	85.2	20.1	5.03	22.9	3.48	22.3	4.62	13.4	1.90	12.3	1.89	3.95	0.85	41.0	11.0	2.43
MR15-02	PC01	3	84	86	212.4	2.124	466	3.25	1.71	8.39	0.23	2.97	0.89	0.50	168	44.2	0.89	6.40	158	294	322	160	92.7	208	117	198	13.1	41.8	8.55	311	66.1	90.6	19.4	71.5	18.8	4.69	21.4	3.26	20.9	4.31	12.6	1.77	11.5	1.77	3.85	0.79	39.6	10.4	2.38
MR15-02	PC01	3	94	96	222.4	2.224	478	3.50	1.69	8.64	0.25	3.17	0.91	0.50	170	42.8	0.97	6.43	170	326	335	161	91.7	211	123	197	12.7	44.8	8.02	304	68.0	92.4	19.7	72.2	19.2	4.81	21.9	3.34	21.4	4.41	12.9	1.83	11.7	1.82	3.91	0.75	40.1	9.96	2.38
MR15-02	PC01	4	4	6	232.6	2.326	480	3.62	1.52	8.06	0.26	3.01	0.93	0.46	156	36.4	0.98	5.89	176	312	320	146	81.0	206	126	178	11.5	55.0	6.21	269	68.5	89.7	19.6	71.9	19.0	4.78	22.0	3.35	21.5	4.49	13.2	1.87	12.0	1.86	3.54	0.67	38.7	8.76	2.24
MR15-02	PC01	4	14	16	242.6	2.426	470	3.50	1.43	7.56	0.26	2.90	0.91	0.44	151	34.9	0.96	5.66	177	290	312	141	77.5	198	122	172	11.2	57.0	5.87	253	67.3	86.4	19.4	71.5	19.0	4.74	21.9	3.31	21.2	4.42	12.9	1.82	11.8	1.81	3.42	0.65	37.6	8.59	2.24
MR15-02	PC01	4	24	26	252.6	2.526	498	3.60	1.57	8.20	0.28	2.83	0.95	0.49	145	40.9	0.93	5.87	155	261	344	143	79.3	178	127	144	11.6	56.2	6.05	257	70.2	89.4	20.5	84.6	19.9	5.01	22.8	3.44	21.9	4.49	13.0	1.82	11.7	1.81	3.46	0.67	37.6	8.89	2.32
MR15-02	PC01	4	34	36	262.6	2.626	503	3.62	1.55	7.97	0.29	2.92	1.00	0.48	156	37.0	1.03	5.91	188	323	353	148	79.7	212	128	178	11.6	62.2	6.21	258	72.2	92.6	21.4	78.3	20.7	5.16	23.7	3.60	22.7	4.68	13.5	1.91	12.2	1.89	3.57	0.68	39.0	9.48	2.37
MR15-02	PC01	4	44	46	272.6	2.726	540	3.68	1.60	8.24	0.32	3.07	1.02	0.49	163	39.6	1.16	6.05	208	326	363	154	85.7	223	137	187	12.2	71.1	6.69	271	77.3	104	22.8	83.3	22.1	5.46	24.8	3.78	23.8	4.86	14.1	1.96	12.7	1.97	3.71	0.68	41.8	10.2	2.40
MR15-02	PC01	4	54	56	282.6	2.826	584	3.89	1.65	8.60	0.35	3.23	1.08	0.49	168	39.5	1.23	6.19	220	277	358	155	88.8	236	150	198	12.7	80.4	6.77	285	83.6	113	24.5	89.2	23.5	5.84	26.6	4.04	25.7	5.24	15.2	2.13	13.6	2.11	3.91	0.71	44.9	10.9	2.52
MR15-02	PC01	4	64	66	292.6	2.926	578	3.85	1.60	8.55	0.35	3.22	1.06	0.49	165	37.9	1.28	6.14	223	299	367	154	87.7	234	147	204	12.8	82.5	6.51	281	82.6	116	24.0	87.0	23.1	5.62	26.0	3.97	25.1	5.13	14.9	2.08	13.3	2.06	4.04	0.73	45.8	11.4	2.50
MR15-02	PC01	4	74	76	302.6	3.026	495	4.11	1.50	8.42	0.28	3.14	0.89	0.43	129	34.3	1.03	5.23	157	241	336	133	79.2	172	118	155	12.5	71.0	5.33	247	68.4	108	19.9	82.2	19.3	4.49	21.4	3.25	20.5	4.18	12.0	1.68	10.7	1.63	4.03	0.82	41.7	12.0	2.16
MR15-02	PC01	4	84	86	312.6	3.126	495	4.22	1.42	8.34	0.30	3.44	0.97	0.43	135	32.2	1.04	5.33	180	224	335	131	80.9	205	122	190	12.3	71.9	5.07	251	69.0	103	20.9	76.0	20.4	4.85	22.5	3.42	21.7	4.35	12.4	1.73	11.1	1.71	3.98	0.78	41.3	11.5	2.12
MR15-02	PC01	4	94	96	322.6	3.226	581	3.89	1.46	8.16	0.37	3.19	1.18	0.46	152	31.9	1.25	5.83	209	261	352	141	80.8	236	151	195	11.9	84.5	5.56	271	83.4	107	24.6	90.1	23.9	5.93	27.0	4.10	25.9	5.30	15.2	2.14	13.5	2.08	3.89	0.66	43.9	10.2	2.47
MR15-02	PC01	5	4	6	332.2	3.322	585	4.04	1.46	8.05	0.38	3.17	1.19	0.45	149	30.3	1.25	5.78	213	261	355	138	79.9	235	151	190	11.6	86.4	5.46	270	83.3	109	24.7	91.0	24.2	5.97	27.2	4.12	26.0	5.30	15.3	2.13	13.7	2.11	3.85	0.65	43.3	10.5	2.50
MR15-02	PC01	5	14	16	342.2	3.422	557	3.51	1.32	7.24	0.35	2.75	1.08	0.40	130	28.3	1.19	5.06	208	298	323	125	67.8	207	139	163	10.3	78.6	4.68	241	77.8	103	24.0	88.8	23.9	5.92	27.2	4.07	25.7	5.16	14.9	2.08	13.3	2.01	3.46	0.60	41.8	10.3	2.43
MR15-02	PC01	5	24	26	352.2	3.522	653	4.00	1.50	8.27	0.41	3.10	1.23	0.47	138	32.9	1.33	5.72	210	280	354	140	78.3	207	166	152	11.6	88.1	5.33	276	90.4	119	27.3	112	27.4	6.69	30.0	4.52	28.5	5.74	16.5	2.28	14.4	2.22	3.80	0.62	44.5	10.7	2.54
MR15-02	PC01	5	34	36	362.2	3.622	596	3.64	1.32	7.19	0.38	2.77	1.14	0.42	133	28.7	1.18	5.29	223	232	307	123	67.0	216	152	162	10.2	82.7	4.44	244	83.2	106	25.7	95.0	25.6	6.38	29.4	4.39	27.7	5.61	16.2	2.24	14.2	2.13	3.46	0.58	41.9	10.1	2.45
MR15-02	PC01	5	44	46	372.2	3.722	699	3.60	1.31	7.16	0.46	2.73	1.29	0.41	133	28.5	1.18	5.31	218	242	306	123	64.2	225	184	156	9.73	80.6	4.09	245	98.7	109	30.8	114	30.9	7.71	35.5	5.33	33.5	6.82	19.5	2.69	17.0	2.51	3.29	0.56	41.2	10.6	2.52
MR15-02	PC01	5	54	56	382.2	3.822	830	3.53	1.24	7.32	0.55	2.87	1.47	0.39	129	24.1	1.24	5.15	228	270	320	120	65.2	235	228	152	9.34	76.7	3.92	244	118	108	37.5	141	38.4	9.54	43.7	6.57	41.2	8.31	23.7	3.27	20.5	3.04	3.24	0.53	39.8	11.5	2.63
MR15-02	PC01	5	64	66	392.2	3.922	938	3.46	1.21	7.12	0.62	2.79	1.56	0.37	127	22.3	1.19	4.95	211	253	306	117	65.1	241	263	148	9.28	72.5	4.00	239	135	109	43.0	161	43.9	10.9	50.2	7.52	47.1	9.50	27.1	3.70	23.1	3.44	3.15	0.52	39.8	12.4	2.74
MR15-02	PC01	5	74	76	402.2	4.022	1464	3.83	1.38	7.80	0.98	2.95	2.22	0.44	141	27.9	1.54	5.54	227	326	373	141	71.2	268	453	139	10.6	85.2	4.43	275	224	139	62.9	259	64.0	15.7	71.9	10.8	68.1	13.7	39.4	5.33	33.2	5.07	3.37	0.55	45.5	14.8	3.34
MR15-02	PC01	5	84	86	412.2	4.122	1999	3.31	1.33	6.73	1.33	2.52	2.81	0.44	147	26.2	1.53	5.46	269	354	347	143	60.9	341	612	172	11.2	86.3	4.02	255	311	184	88.6	334	90.8	22.5	104	15.6	97.9	19.8	56.4	7.62	47.3	6.98	3.55	0.57	48.2	18.0	4.18
MR15-02	PC01	5	94	96	422.2	4.222	2184	3.45	1.36	6.75	1.46	2.49	3.05	0.46	156	27.7	1.77	5.73	309	420	377	153	59.6	366	670	177	11.3	95.9	3.89	265	339	197	97.5	367	100	24.8	114	17.2	107	21.6	61.6	8.31	51.1	7.63	3.67	0.60	51.9	19.0	4.50
MR15-02	PC01	6	4	6	432.5	4.325	3604	3.06	1.43	6.71	2.53	2.52	5.39	0.41	127	30.9	1.58	5.03	267	414	371	158	62.1	428	1145	149	11.8	87.6	4.27	288	554	289	157	637	160	39.4	182	27.1	171	34.7	99.3	13.4	82.3	12.3	3.85	0.65	43.2	26.7	6.11
MR15-02	PC01	6	14	16	442.5	4.425	3354	2.92	1.48	6.71	2.39	2.66	5.03	0.40	124	30.5	1.50	4.89	221	448	364	156	66.0	411	1062	150	11.9	93.8	4.46	307	518	284	146	582	148	36.7	168	25.1	159	32.2	92.7	12.5	77.1	11.6	3.97	0.61	41.2	25.3	5.98
MR15-02	PC01	6	24	26	452.5	4.525	3621	2.78	1.46	6.32	2.58	2.66	5.33	0.37	112	28.1	1.46	4.64	222	483	357	161	64.6	431	1141	148	11.8	78.8	4.24	299	564	308	159	620	159	39.6	182	27.3	172	35.3	102	13.7	84.3	12.6	4.04	0.67	42.5	26.5	6.38
MR15-02	PC01	6	34	36	462.5	4.625	4939	2.41	1.21	5.12	3.48	2.24	7.06	0.31	95.7	24.2	1.89	3.86	294	604	394	163	52.0	558	1531	132	9.06	76.4	3.31	274	746	375	234	936	217	53.9	247	36.8	233	47.3	135	18.2	112	16.8	3.50	0.32	43.4	31.9	7.69
MR15-02	PC01	6	44	46	472.5	4.725	6203	2.51	1.26	5.26	4.40	2.35	8.91	0.31	98.0	25.8	2.24	3.94	337	702	432	181	55.5	693	1941	22.2	7.41	80.2	3.41	291	937	448	296	1187	271	67.1	308	46.0	289	59.2	169	22.7	140	20.8	1.29	0.20	49.0	38.1	9.07
MR15-02	PC01	6	54	56	482.5	4.825	6366	2.44	1.21	5.06	4.41	2.32	8.96	0.30	93.0	23.6	2.13	3.81	320	675	433	177	55.2	691	1976	17.0	9.62	75.2	3.35	285	961	455	303	1236	279	69.5	318	47.4	299	60.7	173	23.4	144	21.5	1.03	0.17	46.8	38.7	9.19
MR15-02	PC01	6	64	66	492.5	4.925	6051	2.44	1.23	5.05	4.19	2.30	8.59	0.31	93.9	24.7	2.05	3.87	315	648	394	174	54.8	648	1869	17.1	9.82	74.0	3.39	280	912	440	289	1182	265	65.5	301	44.7	283	57.4	164	22.0	136	20.2	0.94	0.19	47.6	37.0	8.71
MR15-02	PC01	6	74	76	502.5	5.025	7224	2.34	1.14	4.69	4.98	2.15	10.2	0.28	82.4	23.0	1.78	3.51	245	579	357	167	51.8	760	2219	52.9	9.46	62.6	3.23	258	1087	502	347	1430	318	79.2	363	53.9	341	69.2	198	26.7	165	24.5	2.07	0.29	44.6	43.9	10.7
MR15-02	PC01	6	84	86	512.5	5.125	7835	2.31	1.13	4.71	5.35	2.18	11.0	0.28	79.6	26.0	1.57	3.42	218	511	331	161	53.0	798	2387	94.9	9.95	56.2	3.32	254	1177	537	376	1578	345	86.4	394	58.4	371	75.3	215	29.0	179	26.7	3.00	0.46	42.5	47.6	11.6
MR15-02	PC01	6	94	96	522.5	5.225	7087	2.25	1.09	4.52	4.87	2.08	10.1	0.27	75.2	23.4	1.44	3.27	197	455	307	151	50.5	732	2172	95.7	9.27	51.5	3.12	236	1054	482	339	1440	311	77.3	354	52.6	334	67.7	193	26.0	161	23.8	2.97	0.45	38.2	42.7	10.3
MR15-02	PC01	7	4	6	532.8	5.328	6872	2.39	1.14	4.95	4.58	2.26	9.45	0.29	80.7	27.7	1.20	3.56	165	351	278	146	55.8	641	2086	76.8	9.08	50.5	3.70	247	1017	469	331	1405	305	76.1	347	51.4	327	65.8	187	25.2	155	23.1	2.64	0.42	38.2	43.4	10.2
MR15-02	PC01	7	14	16	542.8	5.428	6930	2.39	1.12	4.96	4.53	2.25	9.37	0.29	81.5	26.4	1.21	3.62	165	347	281	146	55.5	645	2083	57.3	7.97	50.1	3.55	244	1034	471	337	1425	310	77.2	351	52.2	330	66.3	189	25.3	156	23.2	2.05	0.31	38.0	44.0	10.2
MR15-02	PC01	7	24	26	552.8	5.528	6265	2.44	1.13	5.11	4.05	2.27	8.41	0.29	83.7	27.3	1.13	3.77	153	313	264	138	56.5	596	1891	17.4	8.98	51.9	3.63	244	939	424	302	1287	280	69.6	317	47.0	296	59.7	168	22.6	139	20.7	0.91	0.21	37.9	40.6	9.38
MR15-02	PC01	7	34	36	562.8	5.628	6169	2.55	1.14	5.22	3.97	2.34	8.31	0.30	86.3	26.7	1.16	3.87	154	313	264	138	57.6	595	1865	21.0	9.12	54.2	3.71	248	927	416	299	1265	277	68.6	311	46.3	291	58.6	166	22.2	136	20.1	0.96	0.21	38.9	40.4	9.24
MR15-02	PC01	7	44	46	572.8	5.728	6245	2.64	1.18	5.46	4.02	2.44	8.43	0.31	88.2	27.3	1.19	4.01	158	308	266	140	60.7	604	1881	38.0	9.61	57.1	3.92	258	937	424	305	1283	280	69.5	317	46.9	296	59.3	167	22.4	137	20.4	1.37	0.32	40.7	41.8	9.32
MR15-02	PC01	7	54	56	582.8	5.828	5824	2.64	1.20	5.65	3.82	2.54	7.85	0.31	90.1	27.9	1.29	4.09	163	348	280	144	64.4	581	1753	101	9.80	60.3	4.07	265	872	398	284	1204	262	65.0	294	43.7	274	54.8	154	20.7	126	18.7	2.68	0.42	41.3	39.9	8.89
MR15-02	PC01	7	64	66	592.8	5.928	4673	2.82	1.28	6.00	3.17	2.72	5.98	0.31	97.9	27.6	1.41	4.39	172	385	291	142	68.7	507	1438	140	10.00	68.5	4.42	282	716	343	230	887	212	52.5	237	35.2	221	44.1	124	16.6	101	14.9	3.67	0.55	42.7	33.9	7.53
MR15-02	PC01	7	74	76	602.8	6.028	3351	2.22	1.05	4.81	2.28	1.90	4.20	0.22	75.2	21.8	1.04	3.28	138	340	242	108	46.6	338	979	101	7.86	53.1	3.11	201	521	257	151	633	157	39.4	186	27.1	169	33.9	95.6	12.9	79.2	10.8	2.81	0.48	35.1	28.9	6.40
MR15-02	PC01	7	84	86	612.8	6.128	3713	2.84	1.37	6.28	2.51	2.84	4.81	0.33	108	30.9	1.59	4.73	197	387	305	146	74.8	438	1133	144	10.7	75.9	5.22	313	573	302	167	694	171	42.3	190	28.1	175	34.9	97.8	13.1	79.6	11.7	3.74	0.60	47.5	30.1	6.40
MR15-02	PC01	7	94	96	622.8	6.228	2813	2.36	1.20	5.37	1.89	2.07	3.53	0.25	87.7	24.2	1.29	3.80	172	389	272	118	51.8	308	808	110	9.03	63.3	3.78	238	433	236	126	535	132	32.9	155	22.7	140	28.2	79.5	10.7	65.1	8.90	3.07	0.55	41.7	26.3	5.59
MR15-02	PC01	8	4	6	632.3	6.323	3648	2.81	1.38	6.44	2.45	2.85	4.69	0.34	108	30.4	1.55	4.76	202	364	301	147	77.6	432	1110	145	11.0	75.7	5.46	323	560	299	164	687	168	41.6	187	27.7	172	34.1	95.4	12.8	77.7	11.4	3.78	0.60	47.2	30.7	6.29
MR15-02	PC01	8	14	16	642.3	6.423	3205	2.82	1.43	6.52	2.10	2.85	4.08	0.34	114	30.7	1.68	4.92	213	386	309	150	78.4	398	967	148	11.3	80.2	5.80	332	490	277	143	604	147	36.4	164	24.2	150	29.9	83.6	11.2	68.0	9.94	3.83	0.62	50.0	28.1	5.63
MR15-02	PC01	8	24	26	652.3	6.523	3076	2.90	1.48	6.70	1.99	2.91	3.90	0.35	119	33.8	1.72	5.15	220	359	312	152	83.3	387	921	154	11.8	82.2	6.21	348	469	274	137	583	140	34.9	156	23.1	143	28.7	79.8	10.7	64.9	9.52	4.00	0.65	51.9	28.4	5.51
MR15-02	PC01	8	34	36	662.3	6.623	2415	2.32	1.24	5.42	1.56	2.04	2.96	0.26	90.5	25.6	1.21	3.93	176	274	254	116	55.2	272	682	114	9.41	62.6	4.20	238	368	217	108	466	112	28.2	133	19.3	119	23.9	66.9	9.01	55.2	7.47	3.20	0.57	42.8	25.4	4.82
MR15-02	PC01	8	44	46	672.3	6.723	3016	2.94	1.56	7.12	1.94	3.08	3.80	0.38	124	37.6	1.76	5.49	223	394	317	156	90.8	387	895	163	12.4	79.6	6.99	353	458	278	135	577	139	34.3	152	22.5	139	27.5	76.8	10.2	62.2	9.23	4.15	0.69	54.2	29.5	5.39
MR15-02	PC01	8	54	56	682.3	6.823	2372	3.20	1.80	8.02	1.47	3.46	2.99	0.42	141	40.1	2.00	6.21	259	419	345	170	108	347	689	178	13.7	89.3	8.62	402	356	258	105	447	108	26.5	117	17.3	107	21.1	58.6	7.83	47.6	7.12	4.53	0.75	61.2	25.6	4.55
MR15-02	PC01	8	64	66	692.3	6.923	1768	3.28	1.90	8.32	1.06	3.57	2.26	0.43	150	43.6	2.14	6.49	275	430	354	179	118	303	493	180	14.0	92.3	9.65	440	263	234	77.5	327	78.9	19.3	84.2	12.5	76.9	15.2	42.2	5.62	34.3	5.11	4.55	0.76	65.6	21.5	3.79
MR15-02	PC01	8	73	75	701.3	7.013	1680	3.42	1.87	8.22	1.03	3.56	2.21	0.43	149	42.6	2.10	6.44	267	393	343	177	118	305	456	179	13.6	88.8	9.52	438	252	228	74.3	313	75.1	18.4	80.0	11.9	73.3	14.4	40.3	5.37	32.8	4.94	4.40	0.73	63.9	20.5	3.62
MR15-02	PC01	9	4	6	709.5	7.095	1204	2.83	1.72	7.46	0.76	3.07	1.69	0.37	142	35.7	1.82	5.64	274	382	296	152	102	271	311	188	12.1	78.6	8.40	370	170	183	56.4	209	57.7	14.2	62.3	9.32	56.5	11.1	31.2	4.19	25.4	3.71	4.06	0.70	60.2	18.2	3.19
MR15-02	PC01	9	14	16	719.5	7.195	1126	2.56	1.54	6.40	0.68	2.72	1.52	0.34	135	35.1	1.64	5.26	259	357	282	145	94.7	244	288	175	11.4	73.6	7.93	345	161	165	53.1	198	54.4	13.4	59.6	8.81	53.6	10.6	29.9	4.01	24.5	3.54	3.79	0.69	59.1	17.7	3.12
MR15-02	PC01	9	24	26	729.5	7.295	1213	3.00	1.96	8.62	0.74	3.54	1.62	0.42	153	46.7	2.09	6.54	267	443	333	177	127	250	301	175	13.5	85.6	10.6	443	163	211	53.9	226	54.7	13.3	57.6	8.55	52.4	10.3	28.8	3.87	23.7	3.54	4.37	0.78	68.4	18.6	3.19
MR15-02	PC01	9	34	36	739.5	7.395	965	2.59	1.69	7.13	0.55	3.15	1.31	0.37	149	44.7	1.78	5.85	267	388	286	161	118	239	236	190	11.9	76.9	10.2	404	135	173	44.4	164	44.9	10.9	47.5	7.06	43.1	8.43	23.8	3.19	19.5	2.89	3.98	0.72	65.1	17.6	2.95
MR15-02	PC01	9	44	46	749.5	7.495	1058	2.70	1.73	7.47	0.63	3.29	1.47	0.38	155	44.0	1.84	5.97	274	397	289	165	123	256	263	197	12.2	77.5	10.6	410	147	188	48.6	181	49.1	12.0	51.7	7.72	47.0	9.24	26.1	3.53	21.5	3.23	4.03	0.74	66.5	18.4	3.10
MR15-02	PC01	9	54	56	759.5	7.595	845	2.53	1.72	7.47	0.48	3.20	1.18	0.36	148	43.6	1.69	5.64	253	375	267	160	122	224	203	185	11.6	70.0	10.6	399	117	163	38.5	143	38.6	9.32	40.1	6.01	36.7	7.19	20.2	2.72	16.7	2.50	3.85	0.71	62.4	16.7	2.73
MR15-02	PC01	9	64	66	769.5	7.695	851	2.52	1.77	7.77	0.49	3.33	1.18	0.37	152	44.6	1.72	5.81	258	395	270	163	126	226	203	189	12.0	69.9	10.9	407	117	167	38.6	145	38.9	9.38	40.4	6.04	36.6	7.15	20.1	2.71	16.5	2.49	3.93	0.73	63.4	17.1	2.73
MR15-02	PC01	9	74	76	779.5	7.795	942	2.49	1.83	7.99	0.55	3.05	1.21	0.36	134	42.7	1.63	5.51	224	396	280	153	111	186	224	145	11.7	65.9	9.49	366	126	172	41.8	176	42.7	10.4	45.7	6.77	41.2	8.13	22.6	3.05	18.9	2.72	3.76	0.73	59.8	17.0	2.71
MR15-02	PC01	9	84	86	789.5	7.895	802	2.36	1.71	7.55	0.45	3.19	1.10	0.36	144	41.8	1.57	5.40	244	370	250	155	121	208	191	179	11.4	63.4	10.6	388	110	157	36.5	138	37.0	8.83	38.3	5.68	34.6	6.72	19.0	2.54	15.5	2.33	3.72	0.71	60.5	16.7	2.56
MR15-02	PC01	9	94	96	799.5	7.995	902	2.44	1.73	7.73	0.52	3.22	1.27	0.37	142	41.9	1.48	5.36	238	385	246	157	124	214	221	177	11.6	60.0	10.8	375	124	162	41.3	157	41.6	10.1	43.8	6.54	39.9	7.82	22.0	2.94	18.1	2.69	3.74	0.73	58.8	17.8	2.70
MR15-02	PC01	10	4	6	808.4	8.084	836	2.34	1.79	8.05	0.47	3.34	1.15	0.37	144	47.8	1.46	5.42	231	378	244	159	127	203	198	177	11.9	57.9	11.3	388	114	162	38.1	144	38.5	9.27	40.1	5.99	36.3	7.10	19.9	2.67	16.5	2.46	3.80	0.75	59.2	18.2	2.65
MR15-02	PC01	10	14	16	818.4	8.184	750	2.38	1.73	7.78	0.42	3.18	1.06	0.36	139	44.6	1.37	5.18	220	360	232	153	123	189	174	165	11.4	53.6	10.6	366	102	151	33.9	130	34.3	8.24	35.5	5.30	32.3	6.31	17.6	2.37	14.6	2.16	3.57	0.73	55.7	16.8	2.44
MR15-02	PC01	10	24	26	828.4	8.284	1063	2.33	1.86	8.25	0.64	3.20	1.32	0.37	132	45.0	1.44	5.39	208	395	255	156	120	187	252	145	12.2	56.2	10.5	372	142	194	47.3	199	48.3	11.7	51.4	7.65	46.9	9.14	25.8	3.49	21.6	3.11	3.80	0.78	58.0	18.7	2.88
MR15-02	PC01	10	34	36	838.4	8.384	752	2.47	1.85	8.37	0.42	3.37	1.08	0.37	144	48.0	1.45	5.42	234	400	239	159	129	193	174	174	11.8	53.8	11.2	387	101	156	33.6	130	33.8	8.12	35.3	5.26	32.1	6.26	17.7	2.41	14.8	2.20	3.80	0.77	58.3	17.3	2.56
MR15-02	PC01	10	44	46	848.4	8.484	748	2.47	1.85	8.36	0.43	3.39	1.08	0.37	143	45.7	1.42	5.38	232	377	235	160	130	193	174	175	11.8	53.2	11.3	383	101	156	33.2	129	33.3	8.04	34.5	5.18	31.7	6.16	17.5	2.36	14.6	2.20	3.77	0.75	56.2	17.1	2.54
MR15-02	PC01	10	54	56	858.4	8.584	763	2.49	1.88	8.52	0.43	3.46	1.11	0.38	147	46.4	1.42	5.44	233	381	235	161	133	195	179	178	11.9	52.7	11.7	388	102	158	33.7	132	34.0	8.18	35.2	5.27	32.1	6.28	17.8	2.40	14.8	2.23	3.82	0.76	55.6	17.3	2.52
MR15-02	PC01	10	64	66	868.4	8.684	773	2.55	1.91	8.70	0.45	3.51	1.13	0.38	147	46.6	1.42	5.54	233	378	236	163	136	199	183	181	12.0	52.0	11.8	393	104	157	34.3	134	34.4	8.25	35.6	5.30	32.5	6.37	17.9	2.43	15.0	2.25	3.82	0.75	54.4	17.3	2.53
MR15-02	PC01	10	74	76	878.4	8.784	719	2.37	1.87	8.48	0.39	3.32	0.88	0.36	133	51.5	1.36	5.38	196	374	238	155	129	157	160	140	11.9	51.0	11.3	374	94.2	158	31.3	130	31.3	7.48	32.7	4.87	29.7	5.82	16.3	2.21	13.7	2.00	3.71	0.77	54.2	16.9	2.46
MR15-02	PC01	10	84	86	888.4	8.884	727	2.42	1.87	8.44	0.39	3.19	1.00	0.36	140	43.3	1.30	5.28	216	350	219	153	129	183	166	172	11.5	47.1	11.3	371	97.6	166	32.0	115	31.8	7.64	33.4	5.01	30.4	5.98	17.0	2.29	14.1	2.12	3.77	0.75	53.3	17.0	2.51
MR15-02	PC01	10	94	96	898.4	8.984	935	2.45	1.89	8.64	0.55	3.20	1.31	0.38	143	43.4	1.33	5.44	219	351	221	156	131	208	225	176	11.9	47.1	11.6	378	128	185	42.0	153	42.2	10.2	44.9	6.76	41.2	8.13	23.0	3.13	19.2	2.89	3.92	0.79	54.8	18.6	2.84
MR15-02	PC01	11	4	6	909.9	9.099	706	2.48	1.91	8.83	0.37	3.28	0.96	0.37	145	47.6	1.29	5.48	214	332	212	157	134	184	158	173	11.9	45.4	11.9	382	93.8	169	30.6	111	30.3	7.32	31.7	4.81	29.3	5.74	16.3	2.22	13.9	2.08	3.83	0.78	53.8	17.3	2.53
MR15-02	PC01	11	14	16	919.9	9.199	772	2.33	1.90	8.90	0.42	3.34	1.05	0.37	146	48.4	1.32	5.54	219	344	219	161	135	191	179	176	12.0	46.6	12.0	387	103	176	33.5	121	33.3	8.08	35.1	5.32	32.5	6.45	18.3	2.48	15.4	2.32	3.90	0.79	54.8	17.7	2.62
MR15-02	PC01	11	24	26	929.9	9.299	670	2.51	1.91	8.80	0.34	3.42	0.82	0.37	138	53.2	1.34	5.59	197	353	228	160	135	157	149	144	12.1	46.6	11.9	381	87.8	155	28.4	117	28.1	6.74	29.3	4.35	26.7	5.32	15.0	2.06	12.8	1.88	3.78	0.77	53.1	16.0	2.35
MR15-02	PC01	11	34	36	939.9	9.399	697	2.43	1.85	8.65	0.36	3.27	0.95	0.36	140	46.5	1.32	5.42	215	348	216	157	126	183	159	170	11.5	45.4	11.4	364	92.4	167	29.8	108	29.7	7.12	31.0	4.66	28.8	5.68	16.2	2.20	13.8	2.04	3.77	0.75	53.3	16.2	2.36
MR15-02	PC01	11	44	46	949.9	9.499	736	2.35	1.81	8.34	0.40	3.20	1.02	0.36	137	43.7	1.40	5.31	217	391	233	155	121	188	174	167	11.5	49.7	10.8	365	98.8	165	32.0	115	31.7	7.67	33.5	5.04	31.0	6.11	17.4	2.37	14.7	2.19	3.69	0.73	54.3	15.8	2.33
MR15-02	PC01	11	54	56	959.9	9.599	743	2.45	1.82	8.38	0.41	3.25	1.03	0.36	134	42.5	1.33	5.34	210	340	213	155	122	188	175	169	11.5	44.6	10.9	359	100	166	32.2	116	32.0	7.73	33.9	5.08	31.4	6.21	17.6	2.39	14.9	2.22	3.72	0.74	52.4	15.7	2.33
MR15-02	PC01	11	64	66	969.9	9.699	716	2.50	1.80	8.19	0.39	3.20	1.00	0.35	130	39.7	1.31	5.19	203	343	210	154	118	184	168	166	11.4	43.3	10.7	350	96.2	161	31.1	112	31.0	7.52	32.7	4.93	30.3	5.96	16.9	2.29	14.2	2.10	3.68	0.73	50.3	15.1	2.23
MR15-02	PC01	11	74	76	979.9	9.799	816	2.51	1.78	8.01	0.44	3.21	0.99	0.36	125	48.9	1.37	5.27	186	367	226	153	123	168	194	138	11.8	45.1	10.6	359	109	157	35.8	150	35.8	8.63	38.0	5.64	34.6	6.88	19.3	2.62	16.2	2.37	3.69	0.74	52.4	15.7	2.30
MR15-02	PC01	11	84	86	989.9	9.899	805	2.35	1.81	8.31	0.45	3.27	1.11	0.36	133	43.2	1.36	5.28	209	350	216	154	118	192	196	169	11.7	43.9	10.6	354	109	169	35.3	127	35.4	8.54	37.4	5.61	34.5	6.78	19.3	2.60	16.1	2.39	3.67	0.72	51.3	15.7	2.26
MR15-02	PC01	11	94	96	999.9	9.999	859	2.51	1.79	8.19	0.48	3.21	1.20	0.36	131	39.1	1.36	5.21	204	350	214	153	117	201	212	166	11.6	43.5	10.5	352	117	171	38.1	138	38.5	9.28	40.7	6.10	37.5	7.38	20.9	2.81	17.4	2.57	3.62	0.72	51.7	16.2	2.31
MR15-02	PC01	12	4	6	1010.4	10.104	847	2.41	1.77	8.25	0.47	3.21	1.17	0.36	132	41.1	1.32	5.29	201	336	209	153	120	199	205	164	11.7	42.8	10.8	357	115	172	37.7	136	38.0	9.22	40.0	6.03	36.9	7.25	20.3	2.74	17.0	2.51	3.64	0.74	52.4	16.3	2.33

Table S2 (7: continued)

Cruise	Core	Sec.	Int. [cm]	Int. [cm]	Depth [cm]	Depth [m]	ΣREY [ppm]	Na (wt.%)	Mg	Al	P	K	Ca	Ti	V (ppm)	Cr	Mn (wt.%	Fe	Co (ppm)	Ni	Cu	Zn	Rb	Sr	Y	Zr	Nb	Mo	Cs	Ba	La	Ce	Pr	Nd	Sm	Eu	Gd	Tb	Dy	Ho	Er	Tm	Yb	Lu	Hf	Ta	Pb	Th	U
MR15-02	PC09	01	4	6	5	0.05	289	2.67	2.17	8.40	0.11	2.45	1.01	0.48	179	97.6	0.57	5.80	81.1	150	239	137	134	172	47.8	125	14.9	14.3	12.0	734	45.6	98.4	11.1	44.3	9.08	2.20	8.95	1.34	8.24	1.63	4.64	0.66	4.42	0.68	3.02	0.96	33.2	12.3	2.18
MR15-02	PC09	01	24	26	25	0.25	289	2.48	2.03	8.53	0.11	2.43	0.93	0.48	182	95.9	0.57	5.94	82.2	143	232	133	132	173	48.1	129	15.0	17.0	12.2	624	45.3	98.2	11.0	44.6	8.95	2.16	9.00	1.34	8.13	1.63	4.80	0.68	4.43	0.69	3.15	0.96	33.5	12.1	2.27
MR15-02	PC09	01	42	44	43	0.43	329	2.84	2.05	8.27	0.14	2.43	1.01	0.47	183	93.5	0.68	5.92	97.0	152	264	132	127	184	61.5	134	15.4	23.8	12.0	557	50.8	102	12.5	51.0	10.5	2.62	11.0	1.66	10.1	2.01	5.92	0.84	5.47	0.86	3.21	0.95	37.2	12.0	2.28
MR15-02	PC09	02	24	26	72	0.72	317	2.61	2.12	8.69	0.13	2.54	0.86	0.48	186	95.4	0.63	5.80	87.7	150	238	133	137	174	55.3	133	15.6	26.5	13.0	627	49.2	103	12.2	49.7	9.80	2.44	10.5	1.54	9.18	1.89	5.53	0.77	5.10	0.80	3.17	0.97	36.7	12.6	2.34
MR15-02	PC09	02	74	76	122	1.22	346	2.53	2.08	8.37	0.15	2.62	0.92	0.49	184	94.6	0.66	5.79	95.5	160	273	129	135	176	64.9	134	16.3	27.2	12.9	573	53.3	108	13.3	53.5	11.0	2.75	11.6	1.72	10.6	2.18	6.40	0.87	5.82	0.93	3.32	1.04	37.1	12.8	2.35
MR15-02	PC09	03	24	26	172.2	1.722	371	2.67	2.15	8.42	0.17	2.57	0.93	0.52	195	96.9	0.76	6.30	109	180	297	145	136	187	72.7	143	16.5	28.8	12.6	520	55.8	111	14.0	58.1	12.1	3.08	13.1	1.93	11.7	2.39	6.99	0.99	6.30	1.00	3.27	1.01	40.0	12.5	2.38
MR15-02	PC09	03	74	76	222.2	2.222	374	2.70	2.10	8.14	0.17	2.46	0.94	0.53	188	90.2	0.76	6.06	110	180	299	136	128	185	78.1	142	16.4	33.4	12.1	450	57.6	102	14.6	59.2	12.3	3.16	13.5	2.02	12.5	2.52	7.47	1.05	6.79	1.07	3.42	1.01	40.0	12.3	2.39
MR15-02	PC09	04	24	26	272	2.72	435	2.75	2.02	8.05	0.20	2.39	1.00	0.54	190	84.7	0.88	6.06	126	195	309	140	125	192	96.4	144	16.3	37.1	12.2	418	66.5	110	16.8	69.3	14.7	3.84	16.2	2.45	15.3	3.11	9.24	1.29	8.20	1.28	3.45	0.98	40.8	12.2	2.40
MR15-02	PC09	04	74	76	322	3.22	474	2.65	2.03	7.89	0.22	2.35	1.08	0.53	193	84.3	0.99	6.10	143	239	349	142	116	191	112	153	16.2	44.7	12.2	426	72.2	110	18.5	76.3	16.3	4.21	18.4	2.75	17.5	3.59	10.7	1.49	9.62	1.49	3.67	0.93	42.9	11.2	2.39
MR15-02	PC09	05	24	26	372.1	3.721	486	2.92	2.13	8.28	0.24	2.44	1.11	0.54	200	79.7	0.97	6.44	147	237	366	150	114	202	118	153	16.1	47.9	11.5	419	74.2	109	18.5	78.9	16.4	4.30	19.1	2.84	17.9	3.76	11.0	1.51	9.88	1.53	3.68	0.90	40.2	11.1	2.35
MR15-02	PC09	05	74	76	422.1	4.221	491	3.03	2.14	8.30	0.23	2.45	1.18	0.58	200	78.6	0.92	6.33	135	185	348	147	122	204	119	162	17.4	51.3	12.4	388	75.3	109	19.0	80.0	16.9	4.27	19.1	2.89	17.9	3.74	11.0	1.53	9.84	1.51	3.79	0.97	40.7	11.4	2.44
MR15-02	PC09	06	24	26	472.1	4.721	480	3.02	2.14	8.51	0.23	2.51	1.11	0.58	206	65.1	0.89	6.32	135	205	371	153	120	208	112	183	17.7	51.3	12.1	367	73.2	114	18.3	77.7	16.0	4.19	17.9	2.74	17.5	3.63	10.5	1.46	9.48	1.48	3.94	0.98	41.1	11.1	2.36
MR15-02	PC09	06	74	76	522.1	5.221	532	2.94	2.13	8.77	0.26	2.58	1.18	0.60	202	67.8	0.95	6.54	140	208	346	150	116	209	131	184	18.4	53.5	11.5	361	80.5	113	20.8	89.9	18.4	4.76	20.8	3.13	19.9	4.09	11.9	1.64	10.7	1.65	3.89	1.00	38.9	11.3	2.34
MR15-02	PC09	07	24	26	572.1	5.721	581	3.09	1.86	8.74	0.32	2.58	1.30	0.51	170	49.5	0.88	5.86	144	232	351	140	96.7	201	159	160	14.3	47.5	8.73	309	86.7	101	21.9	97.2	20.8	5.46	24.5	3.74	24.2	4.97	14.4	2.01	13.0	2.02	3.79	0.75	35.5	10.1	2.13
MR15-02	PC09	07	74	76	622.1	6.221	712	3.74	1.48	8.55	0.48	2.85	1.40	0.47	144	32.5	1.26	5.22	182	409	414	155	82.5	184	189	151	11.7	70.5	5.41	246	109	123	27.7	122	26.7	6.58	30.3	4.66	29.5	6.03	17.6	2.44	15.8	2.39	3.67	0.63	39.5	10.0	2.10
MR15-02	PC09	08	24	26	672.1	6.721	1495	3.23	1.57	8.42	1.16	2.81	2.40	0.46	145	34.0	1.34	4.98	194	425	359	161	90.6	238	420	144	11.5	80.7	6.34	276	232	171	62.6	285	66.4	15.5	70.4	10.8	67.3	13.4	37.5	5.20	33.0	4.82	3.76	0.61	46.7	15.5	3.24
MR15-02	PC09	08	74	76	722.1	7.221	1072	2.72	1.91	8.74	0.91	2.98	1.81	0.41	153	45.8	1.56	5.00	196	412	282	181	124	193	262	141	12.1	98.6	10.6	368	155	192	45.1	202	47.5	10.7	47.6	7.29	43.7	8.60	23.8	3.33	20.6	2.98	3.80	0.70	54.7	15.5	2.99
MR15-02	PC09	09	24	26	772.6	7.726	860	2.77	1.96	8.87	0.65	2.96	1.45	0.40	158	50.3	1.61	5.11	204	446	274	211	132	179	200	142	11.9	99.9	11.2	378	123	184	35.0	155	35.0	8.39	36.1	5.48	33.1	6.52	18.3	2.53	15.8	2.24	3.83	0.71	58.9	14.6	2.59
MR15-02	PC09	09	74	76	822.6	8.226	829	2.75	1.88	8.77	0.58	3.02	1.40	0.40	153	53.6	1.62	5.15	205	492	267	198	131	183	193	146	12.1	96.7	11.0	386	118	177	33.2	150	34.0	7.98	34.4	5.34	32.0	6.30	17.6	2.43	15.2	2.22	3.87	0.70	55.7	13.8	2.54
MR15-02	PC09	10	24	26	873.1	8.731	678	2.92	1.91	8.95	0.44	3.14	1.20	0.37	148	58.6	1.62	5.06	201	448	251	197	127	190	153	144	11.5	90.3	10.4	381	95.5	165	25.8	115	26.3	6.37	26.9	4.21	25.2	4.95	14.0	1.94	12.0	1.76	3.87	0.67	55.0	12.8	2.18
MR15-02	PC09	10	74	76	923.1	9.231	771	2.87	1.88	8.81	0.52	3.23	1.31	0.38	145	50.4	1.48	4.98	176	399	242	191	130	197	180	148	12.0	78.9	10.6	387	112	165	30.6	138	30.9	7.50	31.9	4.93	29.5	5.82	16.6	2.27	14.2	2.08	3.98	0.70	53.7	13.5	2.30
MR15-02	PC09	11	24	26	971.4	9.714	867	2.74	1.88	8.73	0.55	3.19	1.40	0.39	150	46.2	1.57	5.28	192	511	269	197	130	179	204	148	12.3	82.4	10.9	380	126	175	35.3	158	36.1	8.58	37.3	5.71	33.8	6.74	18.8	2.61	16.2	2.31	4.10	0.74	58.1	14.4	2.37
MR15-02	PC09	11	74	76	1021.4	10.214	931	2.73	1.85	8.63	0.60	3.02	1.42	0.40	150	49.7	1.66	5.13	200	494	268	200	128	179	224	145	12.7	82.6	10.7	372	134	181	38.1	171	39.0	9.54	40.1	6.22	37.7	7.41	20.5	2.81	17.4	2.52	3.80	0.74	55.1	14.0	2.27
MR15-02	PC10	01	4	6	5	0.05	366	2.73	2.05	8.65	0.17	2.72	1.03	0.57	189	105	0.70	6.04	95.8	205	278	160	138	173	69.0	149	15.6	20.3	12.3	492	56.0	109	13.5	59.2	12.5	2.87	12.3	1.95	11.9	2.41	7.07	1.03	6.72	1.02	3.42	0.97	39.7	12.7	2.32
MR15-02	PC10	01	24	26	25	0.25	505	3.40	1.72	8.14	0.30	2.69	1.11	0.53	171	49.2	0.98	5.90	146	486	345	205	92.9	169	135	151	12.1	37.7	7.52	297	75.3	100	17.9	79.0	18.0	4.37	20.0	3.17	20.3	4.22	12.4	1.80	11.8	1.81	3.64	0.67	37.6	8.59	2.04
MR15-02	PC10	01	74	76	75	0.75	557	3.70	1.64	7.87	0.37	2.78	1.30	0.54	172	49.7	1.13	5.66	178	335	391	177	89.4	186	141	151	12.2	81.1	6.72	264	83.0	112	20.9	92.7	20.6	4.95	22.9	3.58	22.3	4.53	13.3	1.87	12.0	1.83	3.80	0.66	39.1	8.76	2.23
MR15-02	PC10	02	24	26	103.7	1.037	574	3.90	1.61	8.15	0.40	2.87	1.27	0.50	167	41.6	1.25	5.46	186	288	378	170	86.8	186	144	159	12.5	98.4	6.27	267	84.3	123	21.4	93.9	21.2	4.89	22.8	3.64	22.4	4.54	13.2	1.88	11.9	1.78	3.92	0.69	42.1	9.95	2.12
MR15-02	PC10	02	74	76	153.7	1.537	642	3.83	1.48	7.69	0.49	2.80	1.53	0.50	161	38.3	1.29	5.44	195	258	381	160	83.7	200	163	150	11.6	111	5.51	263	95.7	122	24.7	109	25.2	5.88	26.7	4.19	26.4	5.28	15.5	2.21	14.0	2.09	3.69	0.58	41.0	9.05	2.21
MR15-02	PC10	03	24	26	203.7	2.037	860	3.86	1.47	7.81	0.66	2.80	1.79	0.48	156	33.1	1.32	5.14	196	301	367	153	79.7	211	236	137	10.5	99.0	4.92	260	129	124	34.2	154	36.1	8.59	39.4	6.05	38.1	7.60	21.6	2.97	19.0	2.84	3.35	0.52	40.2	10.5	2.38
MR15-02	PC10	03	74	76	253.7	2.537	1779	3.67	1.42	7.22	1.59	2.47	3.26	0.55	179	34.2	1.65	5.71	247	392	400	175	73.1	293	514	148	11.9	114	4.64	269	269	171	76.0	344	82.2	18.9	86.6	13.4	85.3	16.9	47.3	6.62	41.9	5.84	3.65	0.57	49.0	15.0	3.56
MR15-02	PC10	04	24	26	303.3	3.033	2800	2.80	1.60	6.83	2.43	2.48	4.43	0.42	148	42.8	1.89	4.62	264	624	381	184	86.5	339	811	138	11.7	110	6.56	300	434	255	124	531	132	29.8	138	21.4	135	26.6	75.6	10.6	65.5	9.20	3.58	0.59	51.4	21.2	4.67
MR15-02	PC10	04	74	76	353.3	3.533	1296	2.39	1.85	8.28	1.03	3.06	2.03	0.45	171	55.0	1.64	5.25	222	558	339	196	133	204	343	159	12.4	111	10.6	371	189	197	55.1	240	58.8	13.5	60.4	9.06	55.9	11.0	30.0	4.17	26.0	3.62	3.82	0.71	57.9	17.7	3.17
MR15-02	PC10	05	24	26	403	4.03	1334	2.47	1.83	7.39	1.36	3.10	2.52	0.39	124	36.0	1.27	4.36	189	510	368	202	104	205	369	145	11.3	77.1	7.52	343	204	184	56.0	245	58.1	13.0	59.8	9.03	56.6	11.2	32.0	4.52	28.4	3.97	3.83	0.63	45.7	8.79	2.73
MR15-02	PC10	05	74	76	453	4.53	1432	2.62	1.96	7.59	1.34	3.29	2.76	0.43	129	36.4	1.33	4.39	195	500	374	211	107	226	393	154	12.0	76.2	7.47	352	219	190	60.7	269	61.8	14.2	65.9	9.94	61.9	12.5	35.4	4.89	30.1	4.26	4.01	0.65	45.8	8.94	2.78
MR15-02	PC10	06	24	26	503.1	5.031	1833	2.73	1.89	7.29	1.77	3.07	3.57	0.41	130	34.6	1.55	4.37	233	561	374	224	98.2	268	491	151	11.2	85.4	7.02	377	294	218	79.8	359	81.8	18.5	85.7	12.8	79.7	15.9	45.4	6.32	39.9	5.66	3.94	0.60	47.3	9.55	3.32
MR15-02	PC10	06	74	76	553.1	5.531	1912	2.60	1.86	7.20	1.75	3.07	3.50	0.41	128	34.9	1.64	4.35	240	552	407	230	96.6	275	521	154	11.6	84.6	6.75	398	302	217	82.5	379	85.3	19.2	90.1	13.4	83.7	16.5	47.9	6.69	41.0	5.81	3.95	0.61	48.1	9.31	3.20
MR15-02	PC10	07	24	26	603.3	6.033	2511	2.65	1.78	6.89	2.34	2.99	4.72	0.36	122	32.4	1.62	3.97	234	534	410	219	93.2	331	704	154	10.9	83.2	6.47	399	402	241	112	502	113	26.3	120	18.1	113	22.6	63.6	8.90	56.4	8.09	3.92	0.56	44.1	11.3	3.89
MR15-02	PC10	07	74	76	653.3	6.533	2139	2.83	1.74	7.04	2.03	3.14	4.11	0.38	121	31.4	1.59	3.99	227	546	418	213	93.9	311	628	157	10.6	81.4	6.30	360	340	212	89.1	404	89.9	20.8	99.9	15.0	96.2	19.8	57.3	8.11	51.5	7.44	3.98	0.55	37.6	10.1	3.34
MR15-02	PC10	08	24	26	704.2	7.042	2010	2.73	1.80	7.03	1.81	3.01	3.78	0.39	121	33.6	1.43	4.10	207	523	416	201	98.0	300	566	163	11.6	70.2	6.93	385	330	198	87.2	395	87.6	20.4	95.6	14.4	89.5	18.1	51.8	7.13	44.2	6.32	4.05	0.62	39.8	10.0	3.14
MR15-02	PC10	08	74	76	754.2	7.542	1776	3.28	1.92	6.47	1.59	3.32	4.90	0.22	94.1	28.5	1.55	3.56	204	453	517	239	82.1	327	544	132	9.10	53.1	5.34	427	289	193	72.1	304	72.0	16.8	79.8	11.7	75.5	15.5	45.9	6.58	43.4	6.28	3.04	0.45	36.6	8.52	3.21
MR15-02	PC10	09	24	26	803.5	8.035	1363	3.17	1.69	6.52	1.10	3.52	3.50	0.24	105	30.2	1.42	3.76	170	470	383	170	86.5	294	391	124	8.71	56.6	5.56	395	217	180	55.5	238	56.7	13.2	61.9	9.02	57.2	11.6	33.2	4.63	29.0	4.27	2.97	0.42	38.5	7.20	2.26
MR15-02	PC10	09	74	76	853.5	8.535	1104	2.95	1.64	6.21	0.94	3.55	3.04	0.33	104	47.4	0.99	4.12	129	305	318	162	79.9	253	320	119	14.0	39.1	5.00	335	175	150	44.2	192	44.5	10.3	48.9	7.03	45.2	9.19	26.7	3.69	23.5	3.50	2.84	0.69	27.1	7.42	2.08
MR15-02	PC10	10	24	26	903.5	9.035	1587	2.87	1.81	6.80	1.17	3.74	3.77	0.24	96.2	37.6	1.18	3.81	154	299	338	193	95.2	277	472	127	9.44	33.5	6.25	401	250	193	64.9	278	65.8	15.2	72.4	10.5	66.2	13.5	39.5	5.44	34.8	4.97	2.99	0.46	32.2	9.70	2.38
MR15-02	PC10	10	74	76	953.5	9.535	1211	2.83	1.76	6.65	0.83	3.85	2.73	0.25	96.6	40.0	1.02	3.93	135	283	302	185	100	221	347	127	10.7	28.7	6.54	387	191	167	49.6	212	49.8	11.5	53.6	7.81	49.1	9.97	28.9	3.99	25.6	3.80	3.12	0.54	31.0	9.79	2.08
MR15-02	PC10	11	24	26	1003.7	10.037	987	2.82	1.75	6.94	0.52	3.70	2.02	0.26	99.1	36.6	0.94	4.00	118	298	284	174	102	193	256	123	11.2	25.2	6.68	371	154	158	41.9	180	41.6	9.77	45.5	6.45	39.7	7.86	22.2	3.02	18.9	2.70	3.17	0.56	29.3	9.12	1.64
MR15-02	PC11	01	4	6	5	0.05	328	2.83	2.07	8.21	0.10	3.18	1.03	0.41	175	101	0.64	5.59	87.7	164	246	160	129	170	63.5	112	14.9	15.5	11.9	580	50.2	96.4	12.7	51.4	11.7	2.62	11.7	1.74	10.6	2.13	6.13	0.89	5.79	0.86	2.64	0.83	35.9	11.5	2.02
MR15-02	PC11	01	24	26	25	0.25	484	3.09	1.86	7.78	0.20	3.34	1.17	0.44	173	71.2	0.97	5.87	141	420	353	192	101	175	124	148	14.8	30.0	9.83	416	72.5	95.1	18.8	78.8	18.3	4.47	20.4	3.04	19.4	3.98	11.6	1.62	10.5	1.57	3.23	0.67	38.4	9.26	1.97
MR15-02	PC11	02	24	26	56.9	0.569	610	4.03	1.81	8.03	0.31	3.54	1.44	0.45	172	40.9	1.35	5.57	201	370	405	186	88.1	201	158	156	13.8	84.0	6.66	314	91.8	120	23.2	99.1	23.2	5.52	25.6	3.80	24.1	4.91	14.2	1.99	13.0	1.94	3.31	0.58	40.0	8.59	2.06
MR15-02	PC11	02	74	76	106.9	1.069	691	4.23	1.68	8.29	0.38	3.63	1.74	0.43	159	36.5	1.54	5.54	230	348	396	190	83.6	221	178	154	13.3	100	5.75	326	101	134	27.4	114	26.9	6.56	29.5	4.40	27.5	5.54	15.8	2.23	14.3	2.13	3.41	0.57	44.6	9.60	2.32
MR15-02	PC11	03	24	26	157.7	1.577	1162	4.14	1.56	7.82	0.71	3.49	2.51	0.39	158	38.1	1.73	5.44	252	365	369	168	77.5	250	343	137	10.8	95.3	4.96	325	175	140	47.8	205	50.2	12.0	56.8	8.20	51.3	10.3	29.5	4.10	26.2	3.72	2.76	0.41	43.1	10.4	2.58
MR15-02	PC11	03	74	76	207.7	2.077	904	2.72	2.02	8.26	0.64	3.60	1.90	0.35	149	52.2	1.76	5.29	192	522	302	213	124	192	233	139	13.4	106	9.92	451	129	172	36.5	155	38.0	8.92	40.1	5.77	35.5	7.03	20.2	2.78	17.4	2.49	3.19	0.70	54.0	12.3	2.53
MR15-02	PC11	04	24	26	258.2	2.582	878	2.95	2.02	8.59	0.58	3.54	1.72	0.32	141	43.6	1.59	4.91	173	504	262	200	126	176	223	126	12.1	93.7	10.2	439	129	162	35.9	153	37.8	9.03	40.4	5.75	35.0	6.94	19.4	2.63	16.2	2.29	2.89	0.66	51.8	12.1	2.33
MR15-02	PC11	04	74	76	308.2	3.082	887	2.98	2.18	9.14	0.57	3.67	1.76	0.34	152	41.9	1.77	5.19	196	578	281	210	135	186	221	136	12.6	99.3	10.8	460	128	179	35.7	151	37.2	8.71	39.5	5.60	34.2	6.84	19.3	2.63	16.0	2.32	2.97	0.63	53.4	12.1	2.29
MR15-02	PC11	05	24	26	358.6	3.586	817	3.34	2.03	8.73	0.49	3.49	1.53	0.32	149	49.1	2.05	4.96	249	650	321	212	121	189	197	134	12.1	113	9.64	481	114	177	32.5	139	33.8	8.03	35.9	5.21	31.2	6.14	17.6	2.35	14.5	2.08	3.07	0.60	58.4	10.8	2.07
MR15-02	PC11	05	74	76	408.6	4.086	983	3.23	2.01	8.81	0.58	3.46	1.86	0.33	149	47.6	2.12	4.87	257	649	323	214	115	203	249	134	12.3	106	9.21	469	140	187	40.0	174	42.0	9.86	44.3	6.36	38.9	7.65	21.4	2.90	17.5	2.48	2.99	0.54	56.1	10.9	2.03
MR15-02	PC11	06	24	26	460	4.6	889	3.05	1.96	8.62	0.51	3.29	1.66	0.32	142	45.7	2.12	4.81	256	642	321	204	109	194	219	125	11.5	101	8.89	459	125	187	35.3	152	36.8	8.57	38.6	5.63	34.3	6.72	18.8	2.58	15.8	2.28	3.08	0.58	63.6	11.0	1.99
MR15-02	PC11	06	74	76	510	5.1	973	3.12	2.00	8.99	0.54	3.42	1.75	0.33	146	46.7	2.04	5.09	248	601	319	208	114	201	246	129	11.7	89.8	9.25	460	136	193	39.3	168	40.4	9.62	43.4	6.35	38.7	7.53	21.2	2.88	17.4	2.50	3.14	0.60	64.2	11.6	1.99
MR15-02	PC11	07	24	26	558.8	5.588	2627	2.88	1.89	8.39	1.65	3.30	4.26	0.33	135	46.6	1.66	4.55	192	521	289	197	109	317	756	128	12.0	76.0	8.14	437	396	252	118	509	127	29.9	138	20.1	122	23.5	65.6	8.86	54.3	7.50	3.04	0.59	56.9	21.6	3.60
MR15-02	PC11	07	74	76	608.8	6.088	1441	2.82	2.14	7.86	1.25	3.39	3.26	0.34	149	46.7	2.21	4.97	235	596	320	230	110	256	382	138	13.9	78.3	8.36	524	205	237	58.7	262	62.5	14.6	66.5	9.69	59.7	11.7	33.7	4.65	29.3	4.17	3.09	0.72	63.5	13.9	2.87
MR15-02	PC11	08	24	26	659.1	6.591	1441	2.84	2.26	7.82	1.07	3.32	2.89	0.32	157	56.9	2.39	4.99	277	752	373	230	108	243	367	140	12.4	83.9	8.50	580	207	250	60.4	265	63.4	14.7	67.2	9.59	58.9	11.4	32.1	4.31	26.4	3.80	2.98	0.54	68.4	13.2	2.78
MR15-02	PC11	08	74	76	709.1	7.091	1752	2.77	2.15	7.37	1.31	3.48	3.40	0.31	143	42.2	1.85	4.98	237	512	287	208	108	254	470	138	11.7	65.2	8.14	501	257	248	75.5	330	79.5	18.4	84.6	12.3	75.2	14.4	41.0	5.58	34.9	4.84	2.96	0.52	57.4	12.7	2.77
MR15-02	PC11	09	24	26	755.9	7.559	1901	2.75	2.08	7.02	1.34	3.48	3.35	0.32	136	40.4	1.85	4.93	240	547	306	207	101	269	522	140	12.6	60.7	7.37	476	284	249	82.0	362	85.4	20.0	93.0	13.2	81.7	15.9	44.8	6.10	37.6	5.26	3.16	0.60	56.7	11.9	2.74
MR15-02	PC11	09	74	76	805.9	8.059	1861	2.75	1.90	8.47	1.39	3.11	3.61	0.38	133	37.4	1.77	4.70	236	544	322	198	99.5	263	515	143	11.3	56.9	7.23	418	281	234	81.4	351	82.1	20.1	90.9	13.3	80.6	16.4	46.4	6.11	36.4	5.32	3.79	0.63	59.2	11.6	3.04
MR15-02	PC11	10	24	26	856.7	8.567	1566	2.40	2.30	8.17	1.08	3.30	3.09	0.38	122	34.0	1.68	4.49	222	545	347	194	98.3	243	421	139	12.2	52.0	7.24	396	239	228	67.3	291	65.4	16.1	73.1	10.7	65.8	13.0	36.4	4.87	30.1	4.31	3.62	0.61	51.5	10.0	2.59
MR15-02	PC11	10	74	76	906.7	9.067	1471	2.64	2.03	8.16	1.14	3.40	2.96	0.39	116	32.8	1.46	4.63	204	494	346	187	100	240	400	143	13.2	40.9	7.04	371	225	214	62.1	273	61.6	14.7	67.1	9.88	61.0	12.0	34.0	4.53	27.9	4.00	3.76	0.65	44.8	9.11	2.33
MR15-02	PC11	11	24	26	957.2	9.572	1610	2.64	2.01	8.52	1.20	3.62	2.95	0.35	115	30.7	1.39	4.62	196	478	347	194	104	254	455	144	12.1	36.2	7.26	376	253	206	67.8	299	67.2	16.1	74.8	10.8	67.5	13.5	38.6	5.10	31.3	4.45	3.69	0.56	44.2	9.50	2.31
MR15-02	PC11	11	74	76	1007.2	10.072	1808	2.66	2.14	8.49	1.53	3.47	3.67	0.37	119	32.1	1.69	4.39	234	550	390	209	98.6	277	529	153	13.1	46.7	6.68	409	288	212	77.4	330	75.4	17.8	83.0	12.1	75.8	15.0	43.8	5.91	36.9	5.29	3.72	0.62	44.5	9.04	2.48

Table S3

Area	ΣREY [ppm]										Resource amount of REY [REO-t/km^2]									
	0-1 m	0-2 m	0-3 m	0-4 m	0-5 m	0-6 m	0-7 m	0-8 m	0-9 m	0-10 m	0-1 m	0-2 m	0-3 m	0-4 m	0-5 m	0-6 m	0-7 m	0-8 m	0-9 m	0-10 m
A1	443	547	758	874	1,078	1,342	1,444	1,445	1,410	1,390	288	711	1,477	2,271	3,500	5,226	6,564	7,505	8,241	9,027
A2	316	338	364	394	417	446	534	593	612	634	205	440	711	1,024	1,357	1,743	2,431	3,084	3,583	4,120
A3	393	501	613	754	832	842	860	867	860	878	255	651	1,194	1,959	2,701	3,282	3,909	4,503	5,027	5,703
A4	339	383	492	572	592	637	678	701	705	702	221	498	959	1,487	1,922	2,482	3,082	3,643	4,120	4,559
A5	313	330	394	455	467	479	503	526	533	538	204	429	768	1,181	1,519	1,866	2,286	2,735	3,116	3,493
A6	287	297	318	339	360	380	400	425	440	457	186	386	620	881	1,169	1,481	1,822	2,207	2,572	2,972
B1	332	427	477	539	1,166	1,916	2,027	1,912	1,798	1,708	216	555	930	1,400	3,788	7,462	9,210	9,929	10,507	11,092
B2	447	769	752	799	965	1,103	1,124	1,109	1,103	1,204	291	1,000	1,467	2,076	3,133	4,297	5,109	5,761	6,447	7,815
B3	450	579	722	1,034	1,221	1,185	1,142	1,108	1,076	1,089	292	752	1,406	2,685	3,962	4,616	5,189	5,755	6,284	7,067
B4	453	604	976	1,100	1,061	1,038	1,028	1,001	967	942	295	785	1,903	2,858	3,444	4,046	4,670	5,201	5,650	6,114
B5	384	466	857	1,162	1,088	1,025	1,039	1,059	1,032	1,008	250	606	1,669	3,018	3,530	3,991	4,722	5,500	6,033	6,546
B6	295	315	372	437	456	467	508	569	577	583	192	410	726	1,135	1,481	1,821	2,309	2,956	3,376	3,788
C1	479	710	748	780	968	1,226	1,292	1,307	1,306	1,343	311	923	1,458	2,027	3,146	4,777	5,873	6,790	7,631	8,725
C2	520	1,056	1,004	969	993	996	978	973	998	1,204	338	1,372	1,957	2,517	3,224	3,883	4,444	5,054	5,834	7,819
C3	439	557	640	878	1,120	1,092	1,050	1,024	995	1,000	285	723	1,247	2,280	3,635	4,254	4,771	5,318	5,813	6,491
C4	552	840	991	1,010	951	905	880	850	819	797	359	1,091	1,930	2,624	3,089	3,526	4,001	4,416	4,788	5,178
C5	421	667	1,361	1,453	1,356	1,285	1,332	1,328	1,279	1,240	274	866	2,651	3,773	4,402	5,008	6,054	6,899	7,472	8,052
C6	324	387	521	583	646	666	769	861	874	888	211	504	1,016	1,517	2,100	2,598	3,498	4,475	5,106	5,770
D1	457	709	749	786	955	1,127	1,154	1,153	1,148	1,204	297	922	1,460	2,043	3,102	4,392	5,248	5,990	6,712	7,815
D2	450	705	773	870	934	904	876	864	861	934	292	916	1,506	2,259	3,032	3,523	3,981	4,489	5,029	6,063
D3	384	503	644	753	795	783	764	755	743	756	250	654	1,256	1,958	2,582	3,050	3,472	3,923	4,344	4,908
D4	378	525	750	849	856	872	913	904	882	866	245	682	1,462	2,207	2,781	3,398	4,147	4,693	5,153	5,623
D5	396	532	891	925	967	1,049	1,271	1,284	1,254	1,226	257	692	1,735	2,403	3,141	4,087	5,772	6,665	7,322	7,960
D6	352	436	605	671	766	823	926	951	938	931	229	567	1,179	1,744	2,489	3,209	4,211	4,941	5,486	6,046

Table S4 (A) La

Area	La [ppm]											Resource amount of La [REO-t/km^2]										
	0-1 m	1-2 m	2-3 m	3-4 m	4-5 m	5-6 m	6-7 m	7-8 m	8-9 m	9-10 m	average	0-1 m	1-2 m	2-3 m	3-4 m	4-5 m	5-6 m	6-7 m	7-8 m	8-9 m	9-10 m	sum
A1	66	96	178	183	288	408	322	225	172	183	212	42	61	112	116	183	258	204	142	109	116	1,342
A2	49	55	63	74	78	89	162	146	110	119	95	31	35	40	47	49	57	103	93	69	76	599
A3	58	90	123	176	167	131	143	133	116	152	129	36	57	78	111	106	83	91	84	73	97	816
A4	51	63	106	122	99	130	141	130	110	103	105	32	40	67	77	63	82	89	82	70	65	668
A5	47	51	76	94	76	79	95	102	85	85	79	30	32	48	59	48	50	60	64	54	54	499
A6	42	45	52	58	64	68	74	85	79	85	65	26	28	33	37	40	43	47	54	50	54	412
B1	49	76	83	103	559	849	410	157	123	125	253	31	48	52	65	354	537	260	99	78	79	1,605
B2	64	161	101	135	238	261	181	140	147	310	174	40	102	64	85	151	165	115	89	93	196	1,100
B3	64	102	146	294	286	142	124	123	113	174	157	40	64	93	186	181	90	79	78	72	110	994
B4	66	110	257	219	132	137	144	121	101	106	139	42	70	163	138	84	87	91	77	64	67	883
B5	55	78	239	306	110	98	160	178	118	116	146	35	49	151	194	70	62	102	113	75	73	924
B6	42	48	69	90	75	72	105	145	90	88	82	27	30	44	57	47	46	67	92	57	56	522
C1	70	139	119	125	254	373	249	207	191	254	198	44	88	75	79	161	236	158	131	121	161	1,254
C2	72	234	125	120	153	140	119	129	169	456	172	46	148	79	76	97	89	75	82	107	289	1,088
C3	62	96	116	237	307	137	114	124	110	157	146	39	61	73	150	194	87	72	78	70	99	925
C4	78	165	191	158	105	100	109	95	84	89	118	50	105	121	100	67	63	69	60	53	57	744
C5	59	131	404	247	132	126	228	191	126	128	177	37	83	256	157	83	80	145	121	80	81	1,122
C6	45	63	112	107	126	104	201	221	136	143	126	29	40	71	68	80	66	127	140	86	91	797
D1	66	141	118	129	241	293	193	167	163	258	177	42	89	75	82	152	186	123	106	104	163	1,121
D2	64	140	131	171	174	109	102	116	123	243	137	40	89	83	108	110	69	64	73	78	154	869
D3	56	90	136	163	146	110	99	110	102	141	115	35	57	86	103	92	69	63	70	65	90	730
D4	55	98	176	172	132	141	169	125	106	109	128	35	62	112	109	84	89	107	79	67	69	813
D5	56	95	236	145	160	208	376	200	144	140	176	35	60	149	92	101	132	238	127	91	89	1,114
D6	49	72	134	120	163	157	226	164	120	125	133	31	46	85	76	103	99	143	104	76	79	842

Table S4 (B) Ce

Area	Ce [ppm]											Resource amount of Ce [REO-t/km^2]										
	0-1 m	1-2 m	2-3 m	3-4 m	4-5 m	5-6 m	6-7 m	7-8 m	8-9 m	9-10 m	average	0-1 m	1-2 m	2-3 m	3-4 m	4-5 m	5-6 m	6-7 m	7-8 m	8-9 m	9-10 m	sum
A1	104	119	148	169	210	250	216	190	173	179	176	69	79	98	112	140	166	143	126	115	119	1,165
A2	102	108	107	110	113	111	149	184	172	171	133	68	71	71	73	75	74	99	122	114	114	880
A3	100	116	129	151	153	150	159	165	160	168	145	66	77	86	100	101	99	105	109	106	111	962
A4	101	106	120	126	116	138	149	148	137	131	127	67	70	80	84	77	92	99	98	91	87	845
A5	99	100	110	115	110	108	115	115	107	109	109	65	66	73	76	73	72	77	76	71	72	722
A6	94	95	100	100	99	99	102	104	105	107	101	62	63	66	66	66	66	68	69	70	71	667
B1	94	102	106	119	300	404	256	183	169	173	191	62	67	70	79	199	268	170	121	112	115	1,264
B2	98	142	131	144	193	207	180	171	178	234	168	65	94	87	96	128	137	120	113	118	155	1,114
B3	97	118	138	197	210	172	162	158	161	177	159	64	78	92	130	139	114	108	105	107	117	1,054
B4	103	120	176	178	146	153	153	144	138	136	145	68	80	117	118	97	101	101	95	92	90	960
B5	101	114	173	205	149	142	162	158	141	140	148	67	76	114	136	99	94	107	105	93	93	985
B6	96	100	109	115	110	108	123	135	121	119	114	64	66	72	76	73	71	82	89	81	79	754
C1	104	137	145	156	212	254	224	206	197	212	185	69	91	96	104	141	168	149	136	131	141	1,225
C2	98	171	148	148	169	174	161	155	178	279	168	65	113	98	98	112	116	107	103	118	185	1,116
C3	97	117	130	173	215	167	154	150	150	163	152	65	78	86	114	143	111	102	100	100	108	1,006
C4	109	137	161	157	138	137	137	131	129	127	136	72	91	107	104	92	91	91	87	85	84	904
C5	102	134	254	217	184	179	212	181	161	161	179	68	89	169	144	122	118	140	120	107	107	1,184
C6	106	113	137	145	159	149	178	181	155	151	147	70	75	91	96	106	99	118	120	103	100	977
D1	101	135	141	149	200	222	193	177	176	205	170	67	90	93	99	132	147	128	118	117	136	1,126
D2	98	132	137	154	165	149	140	138	146	187	144	65	87	91	102	109	99	93	91	97	124	958
D3	97	110	132	145	146	137	127	125	124	137	128	64	73	87	96	97	91	84	83	83	91	849
D4	97	112	150	156	148	157	162	148	141	141	141	64	75	100	104	98	104	107	98	94	94	937
D5	97	120	189	179	189	205	254	190	181	181	178	64	79	125	119	125	136	169	126	120	120	1,184
D6	109	117	150	163	187	186	203	176	160	156	161	72	78	99	108	124	124	135	117	106	103	1,066

Table S4 (C) Pr

Area	Pr [ppm]											Resource amount of Pr [REO-t/km^2]										
	0-1 m	1-2 m	2-3 m	3-4 m	4-5 m	5-6 m	6-7 m	7-8 m	8-9 m	9-10 m	average	0-1 m	1-2 m	2-3 m	3-4 m	4-5 m	5-6 m	6-7 m	7-8 m	8-9 m	9-10 m	sum
A1	17	26	50	53	84	122	89	61	47	52	60	11	17	33	34	55	80	58	40	31	34	392
A2	12	14	16	19	20	23	43	42	30	33	25	8	9	11	12	13	15	28	27	20	22	165
A3	16	25	36	51	51	38	41	39	34	47	38	10	16	23	33	33	25	27	25	22	30	245
A4	13	17	30	34	27	36	39	36	30	28	29	9	11	19	22	18	24	26	23	19	18	189
A5	13	14	22	27	21	22	27	28	24	24	22	8	9	14	18	14	14	18	19	15	16	145
A6	12	13	15	17	19	20	22	25	23	26	19	8	8	10	11	12	13	14	16	15	17	125
B1	14	22	24	31	169	272	120	50	39	39	78	9	14	16	20	110	178	78	33	26	26	510
B2	18	47	31	41	74	83	54	44	47	103	54	12	30	20	27	48	54	35	29	30	67	354
B3	19	30	44	88	91	45	39	38	35	56	48	12	19	29	57	60	29	25	25	23	36	316
B4	18	32	76	64	39	41	42	35	29	31	41	12	21	50	42	25	27	27	23	19	20	265
B5	15	23	73	93	34	30	50	52	35	35	44	10	15	48	61	22	20	32	34	23	23	287
B6	12	14	21	27	23	22	33	44	28	27	25	8	9	13	18	15	14	21	28	18	18	163
C1	19	39	34	37	76	116	73	62	56	77	59	12	26	22	24	50	75	48	40	37	50	384
C2	22	69	39	38	49	45	38	42	55	155	55	14	45	26	25	32	30	25	28	36	101	360
C3	18	28	35	72	98	43	35	38	34	48	45	12	18	23	47	64	28	23	25	22	32	293
C4	22	49	57	46	31	29	32	27	24	26	34	15	32	37	30	20	19	21	18	16	17	224
C5	17	40	126	77	42	41	74	58	39	39	55	11	26	82	50	27	26	48	38	25	26	359
C6	13	18	34	32	38	33	62	68	43	45	39	8	12	22	21	25	22	40	44	28	29	252
D1	19	41	35	39	74	92	58	51	49	81	54	12	26	23	25	48	60	38	33	32	53	350
D2	19	41	40	51	54	34	31	35	38	78	42	12	27	26	33	35	22	20	23	25	51	274
D3	16	26	40	47	43	32	29	31	30	40	33	10	17	26	31	28	21	19	20	19	26	218
D4	15	28	53	49	39	43	55	37	31	32	38	10	19	34	32	25	28	36	24	20	21	250
D5	16	28	72	45	51	69	129	64	45	43	56	11	18	47	29	33	45	84	42	29	28	367
D6	14	21	40	36	49	50	71	50	37	38	41	9	14	26	24	32	33	46	33	24	25	265

Table S4 (D) Nd

Area	Nd [ppm]											Resource amount of Nd [REO-t/km^2]										
	0-1 m	1-2 m	2-3 m	3-4 m	4-5 m	5-6 m	6-7 m	7-8 m	8-9 m	9-10 m	average	0-1 m	1-2 m	2-3 m	3-4 m	4-5 m	5-6 m	6-7 m	7-8 m	8-9 m	9-10 m	sum
A1	71	111	217	223	352	529	393	262	200	215	257	45	70	137	140	222	333	248	165	126	135	1,620
A2	49	57	66	78	84	101	196	186	136	149	110	31	36	42	49	53	63	123	117	86	94	694
A3	64	105	149	215	214	165	179	169	145	193	160	40	66	94	136	135	104	113	106	91	121	1,006
A4	55	71	126	146	119	160	172	157	131	123	126	34	45	79	92	75	101	108	99	82	77	793
A5	51	57	91	114	89	93	113	119	99	100	93	32	36	57	72	56	58	71	75	62	63	584
A6	46	49	59	67	75	82	89	103	93	105	77	29	31	37	42	47	51	56	65	59	66	483
B1	51	84	93	121	680	1,145	509	198	157	151	319	32	53	59	76	429	721	320	124	99	95	2,009
B2	75	196	126	168	302	347	231	186	191	394	222	47	123	79	106	190	218	145	117	121	248	1,396
B3	75	123	183	370	383	188	162	161	147	226	202	47	77	115	233	241	118	102	102	92	142	1,269
B4	74	133	321	273	164	174	177	146	122	129	171	47	83	202	172	104	109	112	92	77	82	1,079
B5	63	94	312	401	142	126	211	219	145	145	186	40	59	197	252	89	79	133	138	92	91	1,171
B6	47	55	84	112	93	91	136	182	111	111	102	30	34	53	71	58	57	86	115	70	70	645
C1	78	165	143	153	315	491	315	263	237	310	247	49	104	90	96	198	309	198	166	150	195	1,556
C2	90	292	163	155	199	186	156	175	221	578	222	56	184	103	98	126	117	98	110	139	364	1,396
C3	73	116	142	297	408	174	144	154	137	193	184	46	73	90	187	257	110	91	97	87	122	1,158
C4	93	205	239	194	128	120	130	112	98	105	142	58	129	150	122	80	75	82	70	62	66	896
C5	69	162	528	326	172	167	308	242	158	161	229	43	102	333	205	108	105	194	152	100	102	1,444
C6	50	73	137	134	159	137	260	286	179	188	160	32	46	86	84	100	86	164	180	113	119	1,009
D1	75	169	144	159	301	384	243	212	204	315	221	47	107	91	100	190	242	153	134	129	199	1,391
D2	75	171	162	211	223	135	127	144	152	299	170	47	108	102	133	140	85	80	91	96	188	1,070
D3	62	106	164	194	175	128	116	125	118	160	135	39	67	103	122	110	81	73	79	74	101	849
D4	62	117	220	206	157	173	227	153	126	129	157	39	74	139	130	99	109	143	97	79	81	988
D5	64	113	293	181	203	272	531	262	181	174	227	40	71	184	114	128	171	334	165	114	110	1,430
D6	54	84	163	149	203	204	291	207	150	154	166	34	53	103	94	128	128	183	130	94	97	1,044

Table S4 (E) Sm

Area	Sm [ppm]											Resource amount of Sm [REO-t/km^2]										
	0-1 m	1-2 m	2-3 m	3-4 m	4-5 m	5-6 m	6-7 m	7-8 m	8-9 m	9-10 m	average	0-1 m	1-2 m	2-3 m	3-4 m	4-5 m	5-6 m	6-7 m	7-8 m	8-9 m	9-10 m	sum
A1	16	26	52	55	82	118	91	62	48	51	60	10	16	33	34	52	74	57	39	30	32	377
A2	10	12	14	17	18	21	45	43	31	34	24	6	7	9	10	11	13	28	27	19	21	152
A3	14	24	35	50	49	38	41	39	33	45	37	9	15	22	31	31	24	26	24	21	28	230
A4	12	16	28	33	26	36	39	35	29	27	28	7	10	18	21	17	22	24	22	18	17	175
A5	11	12	20	26	20	21	26	27	23	23	21	7	8	13	16	12	13	16	17	14	15	131
A6	10	11	13	15	17	19	21	24	23	25	18	6	7	8	10	11	12	13	15	14	16	111
B1	12	21	24	31	162	253	123	51	40	39	76	8	13	15	20	101	158	77	32	25	24	473
B2	17	47	30	40	71	79	55	45	47	98	53	11	29	19	25	45	49	35	28	30	61	332
B3	18	29	44	87	89	45	38	38	35	54	48	11	18	27	55	56	28	24	24	22	34	299
B4	17	31	76	65	38	40	41	33	28	29	40	11	19	48	40	24	25	26	21	17	18	250
B5	14	22	73	95	33	30	50	51	34	34	43	9	14	46	59	21	19	31	32	21	21	272
B6	10	12	19	26	21	21	32	43	26	26	24	7	8	12	16	13	13	20	27	16	16	149
C1	18	40	35	38	75	113	75	63	56	74	59	11	25	22	24	47	71	47	40	35	47	368
C2	21	70	39	38	48	44	38	43	55	145	54	13	44	25	24	30	28	24	27	35	91	339
C3	17	28	34	70	94	42	34	37	33	46	43	11	17	21	44	59	26	22	23	20	29	272
C4	22	49	56	46	30	28	30	26	23	24	33	14	31	35	29	19	17	19	16	14	15	209
C5	16	39	124	77	42	41	74	57	37	38	54	10	24	77	48	26	25	47	36	23	24	341
C6	11	17	32	32	38	33	63	68	42	44	38	7	11	20	20	24	21	40	43	26	27	238
D1	18	41	35	39	71	89	59	51	49	77	53	11	26	22	24	45	55	37	32	31	48	330
D2	18	41	39	50	52	32	30	34	37	73	41	11	26	24	31	33	20	19	21	23	46	254
D3	14	25	39	46	41	30	27	29	27	37	32	9	16	25	29	26	19	17	18	17	23	198
D4	14	28	52	49	37	41	52	36	30	30	37	9	17	32	31	23	26	33	22	19	19	231
D5	15	27	72	45	50	68	125	63	44	43	55	10	17	45	28	32	42	78	39	28	27	345
D6	12	20	40	36	49	50	71	49	35	36	40	8	13	25	23	31	31	44	31	22	23	250

Table S4 (F) Eu

Area	Eu [ppm]											Resource amount of Eu [REO-t/km^2]										
	0-1 m	1-2 m	2-3 m	3-4 m	4-5 m	5-6 m	6-7 m	7-8 m	8-9 m	9-10 m	average	0-1 m	1-2 m	2-3 m	3-4 m	4-5 m	5-6 m	6-7 m	7-8 m	8-9 m	9-10 m	sum
A1	3.9	6.2	12.2	12.7	19.7	28.4	21.6	14.7	11.2	12.2	14.3	2.4	3.9	7.6	7.9	12.3	17.7	13.5	9.2	7.0	7.6	89.2
A2	2.5	3.0	3.6	4.3	4.5	5.5	10.6	10.0	7.4	8.2	5.9	1.5	1.8	2.3	2.7	2.8	3.4	6.6	6.2	4.6	5.1	37.1
A3	3.5	5.9	8.5	12.3	12.1	9.2	9.8	9.2	8.0	10.9	9.0	2.2	3.7	5.3	7.7	7.6	5.8	6.1	5.8	5.0	6.8	56.0
A4	2.8	3.8	7.0	8.1	6.5	8.6	9.2	8.3	6.8	6.4	6.8	1.7	2.4	4.4	5.1	4.1	5.4	5.8	5.2	4.3	4.0	42.2
A5	2.5	2.9	4.9	6.3	4.9	5.1	6.3	6.8	5.7	5.7	5.1	1.6	1.8	3.1	3.9	3.0	3.2	4.0	4.2	3.6	3.6	32.0
A6	2.3	2.5	3.1	3.7	4.1	4.6	5.1	6.0	5.6	6.3	4.3	1.4	1.6	2.0	2.3	2.6	2.9	3.2	3.7	3.5	3.9	27.1
B1	2.9	5.1	5.8	7.6	40.1	62.7	30.5	12.3	9.5	9.4	18.6	1.8	3.2	3.6	4.8	25.1	39.2	19.0	7.7	6.0	5.9	116.2
B2	4.3	11.4	7.4	9.9	17.6	19.4	13.5	11.0	11.6	24.2	13.0	2.7	7.1	4.6	6.2	11.0	12.1	8.4	6.9	7.2	15.1	81.4
B3	4.4	7.2	10.7	21.5	21.9	10.9	9.3	9.3	8.5	13.3	11.7	2.7	4.5	6.7	13.4	13.7	6.8	5.8	5.8	5.3	8.3	73.0
B4	4.2	7.7	18.8	15.9	9.3	9.7	9.9	8.0	6.6	7.0	9.7	2.6	4.8	11.8	9.9	5.8	6.1	6.2	5.0	4.1	4.4	60.6
B5	3.3	5.3	18.1	23.4	8.1	7.2	12.3	12.4	8.2	8.1	10.6	2.1	3.3	11.3	14.6	5.1	4.5	7.7	7.8	5.1	5.1	66.5
B6	2.4	2.9	4.7	6.4	5.2	5.2	7.9	10.6	6.4	6.4	5.8	1.5	1.8	2.9	4.0	3.3	3.2	4.9	6.6	4.0	4.0	36.3
C1	4.4	9.6	8.4	9.0	18.2	27.5	18.1	15.1	13.7	18.1	14.2	2.8	6.0	5.2	5.6	11.4	17.2	11.3	9.4	8.6	11.3	88.8
C2	5.2	17.1	9.6	9.1	11.7	10.7	9.2	10.5	13.7	35.9	13.3	3.2	10.7	6.0	5.7	7.3	6.7	5.8	6.5	8.6	22.5	82.9
C3	4.2	6.8	8.3	17.2	23.1	10.2	8.3	8.9	7.9	11.3	10.6	2.6	4.2	5.2	10.8	14.5	6.4	5.2	5.6	4.9	7.0	66.4
C4	5.4	12.1	13.8	11.1	7.1	6.6	7.2	6.2	5.4	5.7	8.1	3.4	7.6	8.6	6.9	4.4	4.1	4.5	3.8	3.4	3.6	50.4
C5	3.8	9.5	30.6	19.0	10.2	10.0	18.4	14.0	9.0	9.1	13.4	2.4	6.0	19.2	11.9	6.4	6.2	11.5	8.7	5.6	5.7	83.5
C6	2.6	4.1	8.0	7.8	9.3	8.1	15.7	16.7	10.2	10.7	9.3	1.6	2.6	5.0	4.9	5.8	5.0	9.8	10.4	6.4	6.7	58.2
D1	4.3	9.9	8.5	9.3	17.4	21.6	14.1	12.3	11.9	18.7	12.8	2.7	6.2	5.3	5.8	10.9	13.5	8.8	7.7	7.5	11.7	80.1
D2	4.3	10.0	9.5	12.2	12.7	7.8	7.2	8.3	8.9	17.9	9.9	2.7	6.3	6.0	7.7	7.9	4.9	4.5	5.2	5.6	11.2	61.8
D3	3.5	6.2	9.7	11.2	9.8	7.2	6.5	6.9	6.5	9.0	7.6	2.2	3.9	6.0	7.0	6.2	4.5	4.0	4.3	4.0	5.6	47.7
D4	3.4	6.8	12.8	11.9	9.0	9.9	12.7	8.6	7.1	7.3	9.0	2.1	4.3	8.0	7.5	5.6	6.2	7.9	5.4	4.4	4.5	56.0
D5	3.7	6.8	17.7	11.0	12.4	16.8	31.2	15.4	10.7	10.3	13.6	2.3	4.2	11.1	6.9	7.8	10.5	19.5	9.6	6.7	6.5	85.0
D6	2.9	4.9	9.8	8.9	12.1	12.1	17.4	12.0	8.5	8.7	9.7	1.8	3.1	6.1	5.6	7.6	7.6	10.9	7.5	5.3	5.4	60.8

Table S4 (G) Gd

Area	Gd [ppm]											Resource amount of Gd [REO-t/km^2]										
	0-1 m	1-2 m	2-3 m	3-4 m	4-5 m	5-6 m	6-7 m	7-8 m	8-9 m	9-10 m	average	0-1 m	1-2 m	2-3 m	3-4 m	4-5 m	5-6 m	6-7 m	7-8 m	8-9 m	9-10 m	sum
A1	17.4	28.2	56.0	57.7	90.5	130.0	99.8	67.4	51.5	55.4	65.4	10.8	17.6	34.8	35.9	56.3	80.9	62.1	42.0	32.0	34.5	407
A2	10.3	12.6	15.4	18.8	19.9	24.5	48.1	43.9	31.5	35.0	26.0	6.4	7.8	9.6	11.7	12.4	15.2	29.9	27.4	19.6	21.8	162
A3	15.5	26.6	38.6	55.8	54.5	41.2	44.2	41.2	35.2	48.0	40.1	9.6	16.6	24.1	34.7	33.9	25.6	27.5	25.7	21.9	29.9	249
A4	11.9	16.6	31.5	36.7	29.6	39.2	42.0	38.0	31.1	28.9	30.6	7.4	10.3	19.6	22.9	18.4	24.4	26.2	23.6	19.4	18.0	190
A5	10.8	12.7	22.0	28.3	21.9	22.8	28.7	30.9	26.0	25.9	23.0	6.7	7.9	13.7	17.6	13.6	14.2	17.9	19.2	16.2	16.1	143
A6	9.8	10.9	13.7	16.2	18.5	20.6	23.1	26.9	25.4	28.6	19.4	6.1	6.8	8.5	10.1	11.5	12.8	14.4	16.7	15.8	17.8	121
B1	12.6	23.2	26.5	34.9	183.9	285.7	138.1	54.0	41.8	41.5	84.2	7.8	14.4	16.5	21.7	114.5	177.8	86.0	33.6	26.0	25.9	524
B2	19.1	52.2	33.1	44.7	79.6	87.5	60.5	48.5	50.7	105.3	58.1	11.9	32.5	20.6	27.8	49.5	54.5	37.6	30.2	31.5	65.6	362
B3	19.6	32.8	48.8	97.8	98.8	47.9	40.7	40.9	37.1	58.2	52.3	12.2	20.4	30.4	60.9	61.5	29.8	25.3	25.5	23.1	36.2	325
B4	18.7	34.9	86.2	71.5	41.6	43.2	44.7	36.4	29.7	31.5	43.8	11.7	21.7	53.7	44.5	25.9	26.9	27.8	22.7	18.5	19.6	273
B5	15.0	23.9	82.5	105.8	36.0	31.9	54.7	56.5	37.1	36.2	48.0	9.3	14.9	51.4	65.9	22.4	19.8	34.0	35.2	23.1	22.5	299
B6	10.3	12.4	20.7	28.5	23.3	23.2	35.4	47.6	28.9	28.8	25.9	6.4	7.7	12.9	17.8	14.5	14.5	22.0	29.6	18.0	17.9	161
C1	19.9	44.1	37.9	40.6	83.0	125.1	82.2	68.5	61.4	80.8	64.3	12.4	27.4	23.6	25.3	51.7	77.9	51.2	42.6	38.2	50.3	400
C2	23.5	78.5	42.9	40.9	52.5	47.8	40.6	46.6	60.1	156.4	59.0	14.6	48.9	26.7	25.5	32.6	29.8	25.3	29.0	37.4	97.4	367
C3	18.8	31.2	38.0	78.7	105.0	44.8	36.4	39.2	34.6	49.7	47.7	11.7	19.4	23.7	49.0	65.3	27.9	22.7	24.4	21.6	30.9	297
C4	24.5	55.5	62.9	50.2	31.8	29.5	32.4	27.6	24.0	25.6	36.4	15.3	34.6	39.2	31.2	19.8	18.4	20.2	17.2	14.9	15.9	227
C5	17.4	43.5	139.9	85.5	44.9	43.8	81.5	62.5	40.0	40.4	59.9	10.8	27.1	87.1	53.2	28.0	27.3	50.7	38.9	24.9	25.2	373
C6	11.4	18.3	36.0	35.2	41.8	35.6	69.4	74.0	45.2	47.7	41.5	7.1	11.4	22.4	21.9	26.0	22.2	43.2	46.1	28.2	29.7	258
D1	19.2	45.6	38.6	42.3	79.2	97.9	63.7	55.3	53.0	82.9	57.8	11.9	28.4	24.1	26.3	49.3	61.0	39.7	34.4	33.0	51.6	360
D2	19.3	46.0	43.3	55.7	57.3	34.4	31.8	36.7	39.1	78.7	44.2	12.0	28.6	26.9	34.7	35.7	21.4	19.8	22.8	24.3	49.0	275
D3	15.4	28.3	44.2	50.9	44.6	32.1	28.8	31.0	28.7	40.2	34.4	9.6	17.6	27.5	31.7	27.8	20.0	17.9	19.3	17.9	25.0	214
D4	15.1	30.8	58.5	54.3	40.6	44.6	56.6	38.5	31.4	32.2	40.3	9.4	19.2	36.4	33.8	25.3	27.8	35.2	24.0	19.6	20.0	251
D5	16.5	30.9	80.5	48.6	54.8	73.7	137.6	68.6	47.5	45.5	60.4	10.3	19.2	50.1	30.2	34.1	45.9	85.7	42.7	29.5	28.3	376
D6	12.9	22.1	44.5	39.9	54.2	53.4	77.2	53.4	37.7	38.8	43.4	8.0	13.8	27.7	24.8	33.7	33.2	48.1	33.2	23.5	24.2	270

Table S4 (H) Tb

Area	Tb [ppm]											Resource amount of Tb [REO-t/km^2]										
	0-1 m	1-2 m	2-3 m	3-4 m	4-5 m	5-6 m	6-7 m	7-8 m	8-9 m	9-10 m	average	0-1 m	1-2 m	2-3 m	3-4 m	4-5 m	5-6 m	6-7 m	7-8 m	8-9 m	9-10 m	sum
A1	2.7	4.3	8.6	8.7	13.5	19.4	14.9	10.0	7.5	8.1	9.8	1.7	2.7	5.4	5.5	8.6	12.3	9.5	6.3	4.8	5.2	62.1
A2	1.5	1.9	2.3	2.8	3.0	3.7	7.4	6.7	4.9	5.4	4.0	1.0	1.2	1.5	1.8	1.9	2.4	4.7	4.3	3.1	3.4	25.2
A3	2.3	4.0	5.8	8.4	8.2	6.1	6.6	6.2	5.3	7.2	6.0	1.5	2.5	3.7	5.3	5.2	3.9	4.2	3.9	3.4	4.6	38.2
A4	1.8	2.5	4.7	5.5	4.5	5.8	6.2	5.7	4.7	4.3	4.6	1.1	1.6	3.0	3.5	2.8	3.7	4.0	3.6	3.0	2.8	29.1
A5	1.6	1.9	3.3	4.3	3.3	3.5	4.3	4.7	4.0	3.9	3.5	1.0	1.2	2.1	2.7	2.1	2.2	2.7	3.0	2.5	2.5	22.1
A6	1.5	1.7	2.1	2.4	2.8	3.1	3.5	4.1	3.9	4.3	2.9	0.9	1.1	1.3	1.6	1.8	2.0	2.2	2.6	2.5	2.8	18.7
B1	1.9	3.5	4.0	5.2	27.4	42.4	20.4	8.0	6.2	6.2	12.5	1.2	2.2	2.5	3.3	17.4	26.9	12.9	5.1	4.0	3.9	79.5
B2	2.9	7.8	4.9	6.7	11.9	13.0	9.0	7.2	7.6	15.8	8.7	1.8	4.9	3.1	4.2	7.5	8.3	5.7	4.6	4.8	10.0	55.1
B3	3.0	4.9	7.3	14.6	14.7	7.1	6.1	6.1	5.6	8.7	7.8	1.9	3.1	4.6	9.3	9.4	4.5	3.9	3.9	3.5	5.5	49.7
B4	2.8	5.2	12.8	10.7	6.2	6.4	6.7	5.5	4.5	4.7	6.6	1.8	3.3	8.1	6.8	4.0	4.1	4.2	3.5	2.8	3.0	41.6
B5	2.3	3.6	12.3	15.7	5.4	4.8	8.2	8.5	5.6	5.4	7.2	1.4	2.3	7.8	10.0	3.4	3.0	5.2	5.4	3.6	3.5	45.6
B6	1.6	1.9	3.1	4.2	3.5	3.5	5.3	7.1	4.4	4.3	3.9	1.0	1.2	2.0	2.7	2.2	2.2	3.4	4.5	2.8	2.8	24.7
C1	3.0	6.5	5.6	6.0	12.3	18.5	12.1	10.0	9.1	11.9	9.5	1.9	4.1	3.5	3.8	7.8	11.7	7.7	6.3	5.8	7.6	60.3
C2	3.5	11.7	6.4	6.1	7.8	7.1	6.0	6.9	8.9	23.4	8.8	2.2	7.4	4.1	3.9	4.9	4.5	3.8	4.4	5.7	14.9	55.8
C3	2.9	4.7	5.7	11.8	15.7	6.7	5.4	5.8	5.2	7.4	7.1	1.8	3.0	3.6	7.5	10.0	4.3	3.5	3.7	3.3	4.7	45.3
C4	3.7	8.3	9.4	7.5	4.7	4.4	4.9	4.1	3.6	3.8	5.4	2.3	5.3	6.0	4.8	3.0	2.8	3.1	2.6	2.3	2.4	34.6
C5	2.6	6.5	20.8	12.7	6.7	6.5	12.1	9.3	6.0	6.0	8.9	1.7	4.1	13.2	8.1	4.3	4.2	7.7	5.9	3.8	3.8	56.8
C6	1.7	2.8	5.4	5.3	6.2	5.3	10.3	11.0	6.8	7.2	6.2	1.1	1.8	3.4	3.3	4.0	3.4	6.6	7.0	4.3	4.5	39.3
D1	2.9	6.8	5.7	6.3	11.8	14.5	9.4	8.1	7.8	12.3	8.5	1.8	4.3	3.6	4.0	7.5	9.2	5.9	5.1	5.0	7.8	54.3
D2	2.9	6.9	6.5	8.3	8.6	5.1	4.7	5.4	5.8	11.7	6.6	1.9	4.4	4.1	5.3	5.4	3.3	3.0	3.5	3.7	7.5	41.9
D3	2.3	4.3	6.6	7.6	6.7	4.8	4.3	4.6	4.2	5.9	5.1	1.5	2.7	4.2	4.9	4.2	3.0	2.7	2.9	2.7	3.8	32.7
D4	2.3	4.6	8.8	8.1	6.1	6.7	8.4	5.7	4.7	4.8	6.0	1.4	3.0	5.6	5.2	3.9	4.2	5.3	3.7	3.0	3.0	38.2
D5	2.5	4.7	12.0	7.3	8.2	11.0	20.4	10.2	7.1	6.8	9.0	1.6	3.0	7.6	4.6	5.2	7.0	12.9	6.5	4.5	4.3	57.2
D6	2.0	3.3	6.7	6.0	8.1	7.9	11.5	7.9	5.6	5.8	6.5	1.2	2.1	4.2	3.8	5.2	5.0	7.3	5.0	3.6	3.7	41.2

Table S4 (I) Dy

Area	Dy [ppm]											Resource amount of Dy [REO-t/km^2]										
	0-1 m	1-2 m	2-3 m	3-4 m	4-5 m	5-6 m	6-7 m	7-8 m	8-9 m	9-10 m	average	0-1 m	1-2 m	2-3 m	3-4 m	4-5 m	5-6 m	6-7 m	7-8 m	8-9 m	9-10 m	sum
A1	16.8	27.1	54.2	54.6	84.8	121.3	93.1	62.5	47.2	50.5	61.2	10.4	16.8	33.6	33.8	52.6	75.2	57.7	38.7	29.3	31.3	379
A2	9.3	11.4	14.4	17.7	18.9	23.8	46.0	40.7	29.3	32.1	24.4	5.8	7.1	8.9	11.0	11.7	14.7	28.5	25.2	18.2	19.9	151
A3	14.6	25.1	36.3	52.4	50.9	38.1	41.2	37.9	32.6	44.0	37.3	9.1	15.6	22.5	32.5	31.5	23.6	25.5	23.5	20.2	27.3	231
A4	11.2	15.7	29.8	34.5	27.9	36.5	39.1	35.4	29.4	26.7	28.6	7.0	9.7	18.5	21.4	17.3	22.6	24.2	21.9	18.2	16.5	177
A5	10.1	11.9	20.8	26.5	20.7	21.7	27.2	29.6	25.0	24.4	21.8	6.2	7.4	12.9	16.4	12.8	13.5	16.9	18.3	15.5	15.1	135
A6	9.2	10.3	13.0	15.5	17.8	19.9	22.2	25.8	24.5	27.2	18.5	5.7	6.4	8.0	9.6	11.0	12.3	13.8	16.0	15.2	16.9	115
B1	11.9	22.3	25.3	32.8	172.9	267.4	126.1	48.9	38.0	38.2	78.4	7.4	13.8	15.7	20.4	107.2	165.7	78.1	30.3	23.6	23.7	486
B2	18.1	49.2	30.8	41.5	74.1	81.4	55.6	44.1	46.3	96.6	53.8	11.2	30.5	19.1	25.8	45.9	50.5	34.4	27.4	28.7	59.9	333
B3	18.8	31.2	45.8	91.8	91.6	43.8	37.4	37.3	34.1	53.3	48.5	11.7	19.4	28.4	56.9	56.8	27.1	23.2	23.1	21.1	33.0	301
B4	18.0	33.1	80.8	66.6	38.7	39.5	41.4	34.1	27.8	29.1	40.9	11.1	20.5	50.1	41.3	24.0	24.5	25.7	21.1	17.3	18.0	253
B5	14.2	22.5	76.9	97.4	33.3	29.5	50.9	53.5	35.1	33.5	44.7	8.8	13.9	47.7	60.4	20.6	18.3	31.5	33.1	21.8	20.8	277
B6	9.6	11.8	19.6	26.9	22.3	22.3	33.3	44.8	27.5	27.3	24.5	6.0	7.3	12.1	16.7	13.8	13.8	20.7	27.8	17.1	16.9	152
C1	18.8	41.0	34.6	36.9	76.8	115.1	74.5	61.3	55.5	73.7	58.8	11.7	25.4	21.4	22.8	47.6	71.3	46.2	38.0	34.4	45.7	365
C2	22.4	74.0	39.7	37.7	48.3	43.9	37.1	42.3	54.7	143.5	54.4	13.9	45.9	24.6	23.4	30.0	27.2	23.0	26.2	33.9	88.9	337
C3	18.0	29.6	35.5	73.7	97.2	40.8	33.2	35.6	31.4	45.2	44.0	11.2	18.4	22.0	45.7	60.2	25.3	20.6	22.1	19.5	28.0	273
C4	23.4	52.2	58.9	46.8	29.3	27.1	30.1	25.6	22.2	23.7	33.9	14.5	32.3	36.5	29.0	18.2	16.8	18.6	15.8	13.8	14.7	210
C5	16.6	41.0	130.8	79.1	41.4	40.2	74.5	57.8	37.0	37.5	55.6	10.3	25.4	81.1	49.0	25.7	24.9	46.2	35.8	22.9	23.2	345
C6	10.7	17.4	34.1	33.2	39.1	33.1	63.7	68.3	42.1	44.9	38.7	6.6	10.8	21.1	20.6	24.2	20.5	39.5	42.3	26.1	27.8	240
D1	18.2	42.7	35.4	38.7	73.3	90.1	57.6	49.6	47.9	75.7	52.9	11.3	26.5	22.0	24.0	45.4	55.8	35.7	30.7	29.7	46.9	328
D2	18.4	43.5	40.4	52.0	53.0	31.3	29.1	33.4	35.5	71.9	40.8	11.4	27.0	25.0	32.3	32.8	19.4	18.0	20.7	22.0	44.6	253
D3	14.6	27.0	41.4	47.9	41.4	29.4	26.3	28.3	25.9	36.4	31.9	9.1	16.7	25.6	29.7	25.6	18.3	16.3	17.5	16.1	22.5	197
D4	14.4	29.3	54.9	50.9	37.8	41.1	51.6	35.4	28.8	29.4	37.4	8.9	18.2	34.1	31.6	23.4	25.5	32.0	22.0	17.9	18.2	232
D5	15.8	29.2	74.9	44.7	50.2	67.0	124.3	62.5	43.4	41.5	55.3	9.8	18.1	46.4	27.7	31.1	41.5	77.0	38.7	26.9	25.8	343
D6	12.2	21.1	41.9	37.3	50.4	48.8	70.5	48.9	34.8	36.0	40.2	7.6	13.1	26.0	23.1	31.2	30.2	43.7	30.3	21.6	22.3	249

Table S4 (J) Ho

Area	Ho [ppm]											Resource amount of Ho [REO-t/km^2]										
	0-1 m	1-2 m	2-3 m	3-4 m	4-5 m	5-6 m	6-7 m	7-8 m	8-9 m	9-10 m	average	0-1 m	1-2 m	2-3 m	3-4 m	4-5 m	5-6 m	6-7 m	7-8 m	8-9 m	9-10 m	sum
A1	3.4	5.5	10.8	10.8	17.1	24.3	18.7	12.6	9.5	10.1	12.3	2.1	3.4	6.7	6.7	10.6	15.0	11.6	7.8	5.9	6.3	76.0
A2	1.9	2.3	2.9	3.7	3.9	4.9	9.2	8.0	5.8	6.4	4.9	1.2	1.4	1.8	2.3	2.4	3.0	5.7	5.0	3.6	3.9	30.3
A3	3.0	5.1	7.3	10.6	10.3	7.5	8.1	7.5	6.5	8.7	7.4	1.8	3.1	4.5	6.6	6.4	4.7	5.0	4.6	4.0	5.4	46.1
A4	2.2	3.1	6.0	7.0	5.7	7.2	7.6	7.1	5.9	5.3	5.7	1.4	1.9	3.7	4.3	3.5	4.4	4.7	4.4	3.6	3.3	35.3
A5	2.0	2.4	4.2	5.4	4.3	4.4	5.5	6.1	5.1	4.9	4.4	1.2	1.5	2.6	3.4	2.6	2.7	3.4	3.8	3.2	3.1	27.5
A6	1.8	2.1	2.6	3.2	3.7	4.1	4.6	5.3	5.0	5.6	3.8	1.1	1.3	1.6	2.0	2.3	2.5	2.8	3.3	3.1	3.5	23.5
B1	2.4	4.5	5.2	6.6	35.1	53.8	25.1	9.6	7.5	7.6	15.7	1.5	2.8	3.2	4.1	21.7	33.3	15.5	5.9	4.6	4.7	97.4
B2	3.7	10.0	6.2	8.4	15.0	16.3	11.1	8.7	9.1	19.1	10.7	2.3	6.2	3.8	5.2	9.3	10.1	6.8	5.4	5.6	11.8	66.5
B3	3.9	6.4	9.2	18.6	18.5	8.6	7.4	7.4	6.8	10.5	9.7	2.4	3.9	5.7	11.5	11.5	5.3	4.6	4.6	4.2	6.5	60.2
B4	3.7	6.7	16.3	13.4	7.7	7.8	8.2	6.8	5.6	5.8	8.2	2.3	4.1	10.1	8.3	4.8	4.8	5.1	4.2	3.4	3.6	50.6
B5	2.9	4.6	15.6	19.8	6.7	5.9	10.3	11.0	7.2	6.7	9.1	1.8	2.8	9.7	12.2	4.1	3.7	6.4	6.8	4.4	4.1	56.0
B6	1.9	2.4	3.9	5.4	4.5	4.5	6.8	9.1	5.6	5.6	5.0	1.2	1.5	2.4	3.4	2.8	2.8	4.2	5.6	3.5	3.4	30.7
C1	3.8	8.3	6.9	7.3	15.5	22.9	14.7	12.1	11.0	14.6	11.7	2.4	5.1	4.3	4.5	9.6	14.1	9.1	7.5	6.8	9.0	72.4
C2	4.6	15.0	7.9	7.5	9.7	8.7	7.4	8.4	10.8	28.3	10.8	2.9	9.3	4.9	4.7	6.0	5.4	4.6	5.2	6.7	17.5	67.0
C3	3.7	6.1	7.2	15.0	19.7	8.1	6.6	7.1	6.3	9.0	8.9	2.3	3.8	4.5	9.3	12.2	5.0	4.1	4.4	3.9	5.6	54.9
C4	4.8	10.5	11.9	9.4	5.8	5.4	6.0	5.1	4.4	4.7	6.8	2.9	6.5	7.3	5.8	3.6	3.3	3.7	3.2	2.7	2.9	42.1
C5	3.4	8.3	26.4	15.9	8.2	8.0	14.8	11.6	7.4	7.5	11.1	2.1	5.1	16.3	9.8	5.1	4.9	9.1	7.2	4.6	4.6	68.9
C6	2.2	3.5	6.9	6.6	7.8	6.6	12.6	13.6	8.5	9.1	7.7	1.3	2.2	4.3	4.1	4.8	4.1	7.8	8.4	5.2	5.6	47.8
D1	3.7	8.7	7.1	7.8	14.8	17.9	11.4	9.8	9.5	15.0	10.6	2.3	5.4	4.4	4.8	9.1	11.1	7.0	6.0	5.9	9.3	65.3
D2	3.8	8.9	8.1	10.5	10.7	6.3	5.8	6.7	7.0	14.2	8.2	2.3	5.5	5.0	6.5	6.6	3.9	3.6	4.1	4.4	8.8	50.7
D3	3.0	5.5	8.4	9.7	8.4	5.9	5.2	5.7	5.2	7.3	6.4	1.8	3.4	5.2	6.0	5.2	3.7	3.2	3.5	3.2	4.5	39.8
D4	2.9	6.0	11.1	10.3	7.6	8.2	10.3	7.1	5.7	5.8	7.5	1.8	3.7	6.9	6.4	4.7	5.1	6.4	4.4	3.5	3.6	46.4
D5	3.2	6.0	15.1	8.9	10.0	13.3	24.6	12.5	8.6	8.3	11.1	2.0	3.7	9.4	5.5	6.2	8.2	15.2	7.7	5.3	5.1	68.4
D6	2.5	4.3	8.5	7.5	10.1	9.6	13.9	9.7	6.9	7.2	8.0	1.5	2.7	5.2	4.6	6.2	5.9	8.6	6.0	4.3	4.4	49.5

Table S4 (K) Er

Area	Er [ppm]											Resource amount of Er [REO-t/km^2]										
	0-1 m	1-2 m	2-3 m	3-4 m	4-5 m	5-6 m	6-7 m	7-8 m	8-9 m	9-10 m	average	0-1 m	1-2 m	2-3 m	3-4 m	4-5 m	5-6 m	6-7 m	7-8 m	8-9 m	9-10 m	sum
A1	10.0	15.8	30.6	30.6	48.8	69.1	53.2	36.1	27.2	28.9	35.0	6.2	9.8	18.9	18.9	30.1	42.7	32.8	22.3	16.8	17.8	216
A2	5.5	6.8	8.7	10.8	11.4	14.1	26.0	22.4	16.2	17.9	14.0	3.4	4.2	5.4	6.7	7.1	8.7	16.1	13.8	10.0	11.0	86
A3	8.6	14.7	21.0	30.3	29.3	21.3	22.8	21.0	18.2	24.3	21.2	5.3	9.1	13.0	18.7	18.1	13.2	14.1	13.0	11.2	15.0	131
A4	6.5	9.2	17.4	20.2	16.4	20.5	21.7	20.1	16.9	14.9	16.4	4.0	5.7	10.8	12.4	10.2	12.7	13.4	12.4	10.4	9.2	101
A5	5.9	7.0	12.2	15.5	12.3	12.8	16.0	17.6	14.9	14.1	12.8	3.6	4.3	7.5	9.6	7.6	7.9	9.9	10.9	9.2	8.7	79
A6	5.4	6.1	7.7	9.2	10.7	11.9	13.3	15.2	14.6	16.0	11.0	3.3	3.7	4.8	5.7	6.6	7.4	8.2	9.4	9.0	9.9	68
B1	7.0	13.2	14.9	19.0	100.3	152.6	70.3	27.1	21.1	21.4	44.7	4.3	8.1	9.2	11.7	62.0	94.2	43.4	16.7	13.0	13.2	276
B2	10.7	28.5	17.6	23.7	42.5	46.2	31.0	24.4	25.5	53.1	30.3	6.6	17.6	10.9	14.7	26.2	28.5	19.1	15.1	15.7	32.8	187
B3	11.2	18.3	26.3	52.6	52.2	24.2	20.7	20.7	19.0	29.4	27.5	6.9	11.3	16.2	32.5	32.2	14.9	12.8	12.8	11.7	18.2	170
B4	10.6	19.2	46.3	38.0	22.0	21.9	23.3	19.5	15.9	16.2	23.3	6.6	11.9	28.6	23.4	13.6	13.5	14.4	12.1	9.8	10.0	144
B5	8.4	13.1	44.0	55.4	18.9	16.8	29.0	31.4	20.6	18.7	25.6	5.2	8.1	27.2	34.2	11.7	10.4	17.9	19.4	12.7	11.6	158
B6	5.6	6.9	11.5	15.7	13.1	13.1	19.3	25.7	16.2	15.9	14.3	3.5	4.3	7.1	9.7	8.1	8.1	11.9	15.9	10.0	9.8	88
C1	11.1	23.7	19.7	20.8	43.9	64.6	41.4	34.1	31.0	41.2	33.2	6.8	14.7	12.1	12.9	27.1	39.9	25.6	21.1	19.2	25.5	205
C2	13.3	42.8	22.4	21.2	27.3	24.6	20.6	23.5	30.0	78.6	30.4	8.2	26.4	13.9	13.1	16.9	15.2	12.7	14.5	18.5	48.5	188
C3	10.8	17.4	20.5	42.4	55.5	22.7	18.5	19.9	17.5	25.1	25.0	6.6	10.8	12.6	26.2	34.3	14.0	11.4	12.3	10.8	15.5	154
C4	13.8	30.1	33.7	26.5	16.5	15.0	17.0	14.5	12.6	13.3	19.3	8.5	18.6	20.8	16.4	10.2	9.3	10.5	9.0	7.8	8.2	119
C5	9.8	23.7	74.6	44.7	23.1	22.3	41.2	32.8	21.1	21.1	31.4	6.0	14.6	46.1	27.6	14.3	13.8	25.5	20.2	13.0	13.1	194
C6	6.3	10.3	19.8	19.1	22.2	18.6	35.3	38.2	24.1	25.9	22.0	3.9	6.3	12.2	11.8	13.7	11.5	21.8	23.6	14.9	16.0	136
D1	10.8	24.8	20.3	22.1	41.9	50.6	32.0	27.5	26.6	42.0	29.8	6.6	15.3	12.5	13.6	25.9	31.3	19.8	17.0	16.4	25.9	184
D2	11.0	25.4	23.1	29.8	30.1	17.5	16.2	18.7	19.6	39.6	23.1	6.8	15.7	14.3	18.4	18.6	10.8	10.0	11.5	12.1	24.5	143
D3	8.7	15.9	23.9	27.7	23.7	16.6	14.8	16.1	14.5	20.3	18.2	5.3	9.8	14.8	17.1	14.7	10.3	9.1	9.9	9.0	12.5	112
D4	8.4	17.1	31.7	29.4	21.6	23.1	28.7	20.1	16.2	16.4	21.3	5.2	10.6	19.6	18.1	13.3	14.2	17.7	12.4	10.0	10.1	131
D5	9.4	17.2	42.8	25.2	27.9	36.8	68.2	34.9	24.3	23.1	31.0	5.8	10.6	26.4	15.5	17.2	22.7	42.1	21.6	15.0	14.3	191
D6	7.2	12.5	24.1	21.3	28.5	26.7	38.8	27.2	19.5	20.3	22.6	4.4	7.7	14.9	13.1	17.6	16.5	24.0	16.8	12.1	12.6	140

Table S4 (L) Tm

Area	Tm [ppm]											Resource amount of Tm [REO-t/km^2]										
	0-1 m	1-2 m	2-3 m	3-4 m	4-5 m	5-6 m	6-7 m	7-8 m	8-9 m	9-10 m	average	0-1 m	1-2 m	2-3 m	3-4 m	4-5 m	5-6 m	6-7 m	7-8 m	8-9 m	9-10 m	sum
A1	1.42	2.21	4.27	4.26	6.67	9.44	7.34	5.02	3.72	3.94	4.83	0.88	1.36	2.64	2.63	4.11	5.82	4.53	3.09	2.30	2.43	29.8
A2	0.77	0.95	1.22	1.50	1.59	1.96	3.62	3.11	2.24	2.46	1.94	0.48	0.59	0.75	0.93	0.98	1.21	2.23	1.92	1.38	1.52	12.0
A3	1.22	2.03	2.88	4.12	3.98	2.89	3.11	2.87	2.48	3.28	2.89	0.75	1.25	1.78	2.54	2.45	1.78	1.92	1.77	1.53	2.02	17.8
A4	0.93	1.30	2.41	2.77	2.27	2.79	2.93	2.76	2.32	2.03	2.25	0.57	0.80	1.49	1.71	1.40	1.72	1.81	1.70	1.43	1.25	13.9
A5	0.84	0.99	1.71	2.15	1.73	1.79	2.22	2.45	2.08	1.96	1.79	0.52	0.61	1.05	1.33	1.07	1.11	1.37	1.51	1.28	1.21	11.0
A6	0.77	0.87	1.10	1.30	1.51	1.68	1.87	2.12	2.04	2.22	1.55	0.47	0.54	0.68	0.80	0.93	1.04	1.15	1.31	1.26	1.37	9.5
B1	1.00	1.85	2.09	2.62	13.53	20.52	9.44	3.65	2.84	2.89	6.04	0.62	1.14	1.29	1.61	8.35	12.66	5.82	2.25	1.75	1.78	37.3
B2	1.50	3.88	2.41	3.22	5.75	6.23	4.19	3.30	3.42	7.06	4.10	0.92	2.39	1.49	1.99	3.54	3.84	2.59	2.04	2.11	4.35	25.3
B3	1.57	2.52	3.58	7.13	7.05	3.27	2.81	2.81	2.57	3.95	3.73	0.97	1.56	2.21	4.40	4.35	2.02	1.73	1.73	1.59	2.44	23.0
B4	1.49	2.64	6.25	5.15	3.01	2.94	3.16	2.68	2.18	2.21	3.17	0.92	1.63	3.85	3.18	1.85	1.82	1.95	1.65	1.35	1.36	19.6
B5	1.19	1.82	5.96	7.46	2.59	2.30	3.94	4.34	2.86	2.54	3.50	0.73	1.12	3.68	4.60	1.60	1.42	2.43	2.68	1.76	1.57	21.6
B6	0.80	0.98	1.59	2.14	1.80	1.82	2.64	3.52	2.24	2.19	1.97	0.49	0.61	0.98	1.32	1.11	1.12	1.63	2.17	1.38	1.35	12.2
C1	1.56	3.27	2.70	2.84	5.94	8.73	5.61	4.62	4.15	5.50	4.49	0.96	2.02	1.67	1.75	3.66	5.38	3.46	2.85	2.56	3.39	27.7
C2	1.85	5.78	3.04	2.87	3.70	3.32	2.78	3.17	4.00	10.39	4.09	1.14	3.56	1.87	1.77	2.28	2.05	1.72	1.96	2.47	6.41	25.2
C3	1.51	2.41	2.80	5.75	7.50	3.09	2.51	2.70	2.38	3.38	3.40	0.93	1.49	1.73	3.55	4.62	1.90	1.55	1.66	1.47	2.08	21.0
C4	1.91	4.10	4.57	3.61	2.25	2.05	2.33	1.99	1.73	1.83	2.64	1.18	2.53	2.82	2.23	1.39	1.27	1.43	1.22	1.07	1.13	16.3
C5	1.37	3.24	10.02	6.01	3.12	3.01	5.52	4.46	2.87	2.87	4.25	0.85	2.00	6.18	3.70	1.92	1.86	3.41	2.75	1.77	1.77	26.2
C6	0.90	1.44	2.71	2.60	3.00	2.51	4.74	5.14	3.27	3.54	2.99	0.55	0.89	1.67	1.60	1.85	1.55	2.92	3.17	2.02	2.18	18.4
D1	1.51	3.40	2.77	3.00	5.67	6.83	4.32	3.72	3.56	5.59	4.04	0.93	2.10	1.71	1.85	3.49	4.22	2.67	2.30	2.20	3.45	24.9
D2	1.53	3.46	3.15	4.05	4.09	2.39	2.21	2.54	2.66	5.29	3.14	0.95	2.13	1.94	2.50	2.52	1.47	1.37	1.57	1.64	3.26	19.4
D3	1.22	2.20	3.27	3.78	3.25	2.29	2.03	2.21	1.99	2.75	2.50	0.75	1.36	2.01	2.33	2.00	1.41	1.25	1.36	1.23	1.70	15.4
D4	1.19	2.37	4.31	4.00	2.95	3.13	3.87	2.75	2.22	2.25	2.90	0.73	1.46	2.66	2.46	1.82	1.93	2.39	1.70	1.37	1.39	17.9
D5	1.31	2.38	5.78	3.40	3.76	4.92	9.10	4.73	3.30	3.15	4.18	0.81	1.47	3.56	2.10	2.32	3.03	5.61	2.92	2.03	1.94	25.8
D6	1.02	1.75	3.29	2.91	3.87	3.58	5.21	3.69	2.67	2.78	3.08	0.63	1.08	2.03	1.79	2.39	2.21	3.21	2.27	1.64	1.71	19.0

Table S4 (M) Yb

Area	Yb [ppm]											Resource amount of Yb [REO-t/km^2]										
	0-1 m	1-2 m	2-3 m	3-4 m	4-5 m	5-6 m	6-7 m	7-8 m	8-9 m	9-10 m	average	0-1 m	1-2 m	2-3 m	3-4 m	4-5 m	5-6 m	6-7 m	7-8 m	8-9 m	9-10 m	sum
A1	9.2	14.1	26.9	26.5	41.3	58.4	45.8	31.7	23.4	24.7	30.2	5.68	8.67	16.52	16.31	25.41	35.91	28.16	19.50	14.36	15.19	185.7
A2	5.1	6.2	7.8	9.7	10.3	12.7	23.0	19.4	14.0	15.3	12.3	3.13	3.80	4.79	5.98	6.30	7.81	14.16	11.92	8.58	9.42	75.9
A3	7.9	12.9	18.0	25.7	24.7	17.9	19.3	17.8	15.4	20.1	18.0	4.84	7.92	11.06	15.78	15.18	11.03	11.87	10.96	9.48	12.38	110.5
A4	6.0	8.3	15.2	17.4	14.4	17.3	18.1	17.3	14.7	12.7	14.1	3.71	5.12	9.32	10.70	8.84	10.64	11.14	10.67	9.02	7.78	86.9
A5	5.5	6.4	10.8	13.5	11.0	11.4	14.1	15.6	13.3	12.3	11.4	3.37	3.95	6.66	8.29	6.76	7.00	8.64	9.58	8.16	7.57	70.0
A6	5.0	5.7	7.2	8.5	9.8	10.9	12.1	13.6	13.1	14.1	10.0	3.09	3.50	4.40	5.21	6.00	6.67	7.42	8.36	8.03	8.66	61.3
B1	6.5	11.9	13.4	16.4	83.4	126.2	57.6	22.4	17.5	17.9	37.3	4.02	7.33	8.22	10.11	51.28	77.57	35.43	13.75	10.78	11.01	229.5
B2	9.7	24.3	15.1	20.0	35.4	38.4	25.9	20.3	20.9	43.0	25.3	5.96	14.95	9.27	12.29	21.77	23.63	15.91	12.49	12.86	26.41	155.5
B3	10.1	15.9	22.3	43.9	43.2	20.1	17.3	17.3	15.8	24.1	23.0	6.23	9.80	13.70	26.99	26.58	12.34	10.62	10.62	9.74	14.83	141.5
B4	9.6	16.7	38.5	31.7	18.7	18.0	19.5	16.8	13.7	13.7	19.7	5.92	10.24	23.70	19.51	11.50	11.09	11.99	10.32	8.44	8.43	121.2
B5	7.7	11.5	36.5	45.2	16.0	14.3	24.2	27.1	18.0	15.6	21.6	4.71	7.09	22.44	27.82	9.85	8.82	14.88	16.66	11.05	9.60	132.9
B6	5.3	6.4	10.2	13.7	11.7	11.8	16.8	22.1	14.2	13.8	12.6	3.25	3.96	6.29	8.42	7.18	7.24	10.33	13.58	8.75	8.52	77.5
C1	10.1	20.7	16.9	17.6	36.5	53.7	34.6	28.5	25.4	33.7	27.8	6.22	12.72	10.37	10.80	22.45	33.03	21.29	17.50	15.63	20.74	170.8
C2	11.9	36.0	18.8	17.7	22.7	20.4	17.2	19.5	24.4	63.0	25.2	7.34	22.15	11.58	10.87	13.98	12.57	10.56	11.97	14.99	38.76	154.8
C3	9.7	15.3	17.5	35.5	45.9	19.0	15.5	16.6	14.6	20.7	21.0	5.97	9.38	10.77	21.81	28.25	11.67	9.51	10.20	9.01	12.70	129.3
C4	12.2	25.6	28.3	22.4	14.0	12.7	14.5	12.4	10.8	11.4	16.4	7.52	15.73	17.40	13.75	8.64	7.82	8.89	7.62	6.64	7.01	101.0
C5	8.9	20.2	61.4	36.6	19.2	18.5	33.6	27.5	17.9	17.7	26.1	5.45	12.44	37.74	22.53	11.81	11.37	20.64	16.90	10.99	10.87	160.7
C6	5.9	9.3	17.1	16.5	18.8	15.7	29.2	31.7	20.4	22.0	18.7	3.61	5.71	10.53	10.12	11.55	9.68	17.94	19.47	12.53	13.55	114.7
D1	9.8	21.5	17.3	18.6	34.8	42.0	26.6	22.9	21.8	34.2	24.9	6.01	13.20	10.61	11.42	21.41	25.85	16.35	14.06	13.40	21.02	153.3
D2	9.9	21.7	19.6	25.1	25.2	14.8	13.7	15.7	16.3	32.2	19.4	6.08	13.36	12.06	15.40	15.49	9.08	8.43	9.65	10.03	19.81	119.4
D3	7.9	14.0	20.4	23.5	20.1	14.2	12.6	13.7	12.4	17.0	15.6	4.85	8.60	12.54	14.44	12.37	8.72	7.76	8.45	7.60	10.43	95.8
D4	7.7	15.0	26.8	24.8	18.4	19.3	23.8	17.2	13.8	14.0	18.1	4.71	9.21	16.48	15.28	11.29	11.88	14.63	10.56	8.51	8.63	111.2
D5	8.5	15.1	35.6	20.9	23.0	29.8	55.1	29.1	20.4	19.4	25.7	5.25	9.26	21.91	12.87	14.13	18.35	33.85	17.87	12.52	11.95	158.0
D6	6.7	11.2	20.5	18.1	23.9	21.8	31.8	22.7	16.6	17.2	19.1	4.09	6.90	12.62	11.13	14.70	13.43	19.56	13.98	10.19	10.61	117.2

Table S4 (N) Lu

Area	Lu [ppm]											Resource amount of Lu [REO-t/km^2]										
	0-1 m	1-2 m	2-3 m	3-4 m	4-5 m	5-6 m	6-7 m	7-8 m	8-9 m	9-10 m	average	0-1 m	1-2 m	2-3 m	3-4 m	4-5 m	5-6 m	6-7 m	7-8 m	8-9 m	9-10 m	sum
A1	1.41	2.11	3.85	3.79	6.04	8.51	6.61	4.59	3.42	3.61	4.39	0.87	1.30	2.36	2.33	3.71	5.23	4.06	2.82	2.10	2.22	27.0
A2	0.80	0.98	1.22	1.50	1.58	1.95	3.40	2.80	2.03	2.22	1.85	0.49	0.60	0.75	0.92	0.97	1.20	2.09	1.72	1.25	1.36	11.3
A3	1.22	1.96	2.72	3.87	3.72	2.67	2.84	2.62	2.28	2.97	2.69	0.75	1.20	1.67	2.38	2.28	1.64	1.74	1.61	1.40	1.82	16.5
A4	0.93	1.27	2.31	2.64	2.19	2.58	2.67	2.60	2.21	1.88	2.13	0.57	0.78	1.42	1.62	1.35	1.58	1.64	1.59	1.36	1.16	13.1
A5	0.84	0.99	1.66	2.06	1.70	1.75	2.15	2.39	2.04	1.87	1.74	0.52	0.61	1.02	1.26	1.04	1.08	1.32	1.47	1.25	1.15	10.7
A6	0.77	0.87	1.10	1.30	1.51	1.68	1.87	2.09	2.01	2.15	1.54	0.47	0.54	0.67	0.80	0.93	1.03	1.15	1.28	1.24	1.32	9.4
B1	1.00	1.82	2.03	2.47	12.44	18.68	8.32	3.29	2.59	2.64	5.53	0.61	1.12	1.25	1.52	7.64	11.47	5.11	2.02	1.59	1.62	33.9
B2	1.48	3.64	2.25	2.99	5.28	5.69	3.77	2.95	3.06	6.35	3.75	0.91	2.23	1.38	1.83	3.24	3.49	2.32	1.81	1.88	3.90	23.0
B3	1.56	2.43	3.36	6.60	6.47	2.98	2.56	2.55	2.35	3.57	3.44	0.96	1.49	2.06	4.05	3.97	1.83	1.57	1.57	1.44	2.19	21.1
B4	1.49	2.53	5.77	4.76	2.80	2.65	2.88	2.50	2.05	2.02	2.95	0.91	1.55	3.54	2.92	1.72	1.63	1.77	1.54	1.26	1.24	18.1
B5	1.18	1.76	5.51	6.79	2.42	2.16	3.62	4.09	2.70	2.30	3.25	0.73	1.08	3.38	4.17	1.49	1.33	2.22	2.51	1.66	1.41	20.0
B6	0.81	0.99	1.56	2.07	1.79	1.81	2.56	3.33	2.15	2.08	1.92	0.50	0.61	0.96	1.27	1.10	1.11	1.57	2.04	1.32	1.28	11.8
C1	1.53	3.05	2.46	2.57	5.39	7.81	4.98	4.08	3.70	4.92	4.05	0.94	1.87	1.51	1.58	3.31	4.80	3.06	2.50	2.27	3.02	24.9
C2	1.82	5.36	2.78	2.61	3.37	3.00	2.51	2.82	3.55	9.34	3.71	1.12	3.29	1.71	1.60	2.07	1.84	1.54	1.73	2.18	5.73	22.8
C3	1.50	2.34	2.65	5.34	6.87	2.82	2.30	2.45	2.17	3.05	3.15	0.92	1.43	1.63	3.28	4.22	1.73	1.41	1.51	1.33	1.87	19.3
C4	1.88	3.86	4.22	3.33	2.08	1.87	2.13	1.83	1.59	1.68	2.45	1.16	2.37	2.59	2.04	1.27	1.15	1.31	1.12	0.98	1.03	15.0
C5	1.36	3.06	9.17	5.46	2.85	2.74	4.95	4.09	2.65	2.60	3.89	0.84	1.88	5.63	3.35	1.75	1.68	3.04	2.51	1.63	1.60	23.9
C6	0.90	1.42	2.59	2.48	2.81	2.35	4.32	4.69	3.03	3.28	2.79	0.55	0.87	1.59	1.52	1.72	1.44	2.65	2.88	1.86	2.01	17.1
D1	1.49	3.19	2.56	2.75	5.16	6.14	3.84	3.30	3.17	5.01	3.66	0.91	1.96	1.57	1.69	3.17	3.77	2.36	2.03	1.95	3.08	22.5
D2	1.52	3.28	2.94	3.74	3.75	2.18	2.03	2.30	2.39	4.76	2.89	0.93	2.01	1.81	2.30	2.30	1.34	1.24	1.41	1.47	2.92	17.7
D3	1.22	2.14	3.08	3.51	2.99	2.09	1.86	2.03	1.82	2.49	2.32	0.75	1.32	1.89	2.16	1.84	1.28	1.14	1.24	1.12	1.53	14.3
D4	1.18	2.29	4.04	3.71	2.72	2.84	3.51	2.55	2.04	2.07	2.69	0.73	1.40	2.48	2.28	1.67	1.74	2.16	1.56	1.25	1.27	16.5
D5	1.32	2.31	5.36	3.11	3.40	4.40	8.11	4.31	3.02	2.90	3.82	0.81	1.42	3.29	1.91	2.09	2.70	4.98	2.65	1.86	1.78	23.5
D6	1.02	1.72	3.10	2.72	3.56	3.20	4.68	3.36	2.46	2.56	2.84	0.63	1.05	1.90	1.67	2.19	1.96	2.87	2.06	1.51	1.57	17.4

Table S4 (O) Y

Area	Y [ppm]											Resource amount of Y [REO-t/km^2]										
	0-1 m	1-2 m	2-3 m	3-4 m	4-5 m	5-6 m	6-7 m	7-8 m	8-9 m	9-10 m	average	0-1 m	1-2 m	2-3 m	3-4 m	4-5 m	5-6 m	6-7 m	7-8 m	8-9 m	9-10 m	sum
A1	103	167	327	333	546	766	588	404	309	331	388	71	115	224	228	375	525	404	277	212	227	2,658
A2	56	70	91	115	123	155	288	246	177	194	152	38	48	62	79	85	106	197	169	122	133	1,039
A3	84	152	222	331	311	226	245	224	193	266	225	57	104	152	227	214	155	168	154	132	183	1,546
A4	63	92	183	216	173	221	236	221	183	162	175	43	63	126	148	119	152	162	152	126	111	1,200
A5	54	65	120	157	121	128	163	182	151	144	128	37	44	82	108	83	88	112	125	104	99	881
A6	47	53	68	84	100	113	129	152	143	159	105	32	37	47	58	68	78	89	104	98	109	720
B1	65	129	149	190	1,137	1,710	789	280	216	223	489	45	88	102	131	780	1,173	541	192	148	153	3,352
B2	104	309	180	249	462	503	333	249	267	598	325	71	212	124	171	317	345	229	170	183	410	2,231
B3	105	185	275	580	555	248	212	210	191	315	288	72	127	189	398	380	170	145	144	131	216	1,972
B4	104	199	503	416	232	230	245	205	165	171	247	72	137	345	285	159	158	168	141	113	117	1,695
B5	80	130	470	601	191	169	297	331	210	191	267	55	89	323	412	131	116	204	227	144	131	1,831
B6	48	61	108	153	124	123	193	274	161	157	140	33	42	74	105	85	84	132	188	110	108	961
C1	115	261	212	223	491	724	462	375	343	472	368	79	179	146	153	337	496	317	257	235	324	2,523
C2	129	471	232	217	284	254	210	231	315	895	324	89	323	159	149	195	174	144	158	216	614	2,220
C3	100	174	211	458	589	232	187	201	175	263	259	69	119	145	314	404	159	128	138	120	180	1,776
C4	137	320	361	286	171	155	176	149	128	138	202	94	219	248	196	117	106	121	102	88	94	1,386
C5	93	247	810	478	237	226	431	348	218	221	331	64	169	556	328	162	155	295	239	149	152	2,270
C6	56	98	203	193	227	183	377	420	253	276	228	38	67	139	132	156	125	259	288	173	189	1,567
D1	107	268	215	233	460	559	349	293	285	473	324	73	184	148	160	316	384	240	201	196	325	2,225
D2	104	268	242	321	316	177	163	186	201	437	241	71	184	166	220	217	121	112	128	138	300	1,656
D3	81	160	254	301	250	170	149	163	147	212	189	56	110	174	207	171	116	102	112	100	145	1,294
D4	79	174	337	317	224	237	291	204	164	168	220	54	119	231	218	153	163	200	140	112	115	1,506
D5	86	171	457	261	289	381	706	357	247	240	320	59	117	314	179	198	261	484	245	170	165	2,191
D6	66	122	253	220	301	274	412	289	202	215	235	45	84	174	151	206	188	282	198	139	147	1,614

Table S5 (A)

Core No.	KR13-02_PC05																									KR13-02_PC04						
depth [mbsf]	1.5	2.47	2.47	2.97	2.97	2.97	2.97	2.97	2.97	2.97	2.97	2.97	2.97	2.97	2.97	2.97	2.97	2.97	3.47	3.47	3.97	5.965	6.965	6.965	7.955	7.99	7.99	8.39	8.39	8.39	8.39	11.96
SiO_2 [%]	0.29	0.10	0.00	0.05	0.05	0.08	0.08	0.04	0.05	0.02	0.08	0.08	0.03	0.06	0.05	0.04	0.10	0.05	0.07	0.31	0.12	0.14	0.08	0.16	0.20	0.10	0.13	0.02	0.04	0.09	0.06	0.31
TiO_2	0.05	0.06	0.06	0.04	0.03	0.04	0.04	0.04	0.03	0.03	0.07	0.04	0.03	0.03	0.03	0.03	0.04	0.03	0.03	0.05	0.04	0.10	0.05	0.04	0.06	0.08	0.05	0.05	0.04	0.03	0.05	0.05
Al_2O_3	0.03	0.00	0.00	0.01	0.01	0.00	0.00	0.00	0.00	0.00	0.00	0.00	0.00	0.00	0.01	0.00	0.00	0.00	0.00	0.09	0.04	0.05	0.04	0.06	0.05	0.02	0.02	0.00	0.01	0.04	0.03	0.10
FeO	0.19	0.12	0.06	0.09	0.15	0.04	0.11	0.09	0.08	0.11	0.08	0.09	0.06	0.06	0.06	0.13	0.09	0.14	0.16	0.17	0.23	0.21	0.09	0.16	0.13	0.19	0.21	0.09	0.07	0.07	0.14	0.12
MnO	0.45	0.02	0.00	0.03	0.02	0.06	0.00	0.03	0.01	0.00	0.02	0.01	0.00	0.01	0.00	0.00	0.02	0.00	0.09	0.14	0.01	0.10	0.13	0.05	0.03	0.04	0.02	0.00	0.05	0.01	0.00	0.11
MgO	0.49	0.26	0.29	0.56	0.36	0.42	0.44	0.60	0.50	0.16	0.48	0.54	0.39	0.36	0.44	0.44	0.49	0.42	0.39	0.39	0.52	0.75	0.50	0.44	0.20	0.25	0.21	0.36	0.51	0.51	0.48	0.47
CaO	37.9	47.6	47.4	41.4	46.2	41.9	45.0	41.3	45.3	40.2	44.1	48.9	39.6	39.9	40.0	44.0	43.3	42.7	33.0	44.3	43.5	39.3	41.7	43.7	51.3	45.9	40.5	44.4	43.8	41.4	45.4	43.2
Na_2O	1.42	1.36	1.28	1.78	1.39	1.41	1.39	1.79	1.36	0.62	1.60	1.14	1.15	1.27	1.38	1.47	1.45	1.24	1.57	1.71	1.34	1.45	1.34	1.73	1.25	0.91	0.91	1.23	1.13	1.11	1.29	1.01
K_2O	0.00	0.04	0.02	0.04	0.04	0.04	0.02	0.05	0.04	0.03	0.04	0.07	0.03	0.02	0.04	0.04	0.00	0.05	0.04	0.10	0.04	0.05	0.04	0.04	0.04	0.08	0.05	0.05	0.01	0.06	0.03	0.12
P_2O_5	29.0	38.4	37.9	28.6	32.8	30.0	31.2	28.6	31.2	24.3	30.4	32.2	26.9	27.7	27.4	30.0	30.3	28.6	22.7	33.5	30.5	28.2	29.9	31.0	39.3	32.8	27.6	33.1	30.3	30.7	32.6	31.2
Total (Major elements)	69.8	87.9	87.0	72.6	81.0	74.0	78.3	72.5	78.6	65.4	76.8	83.0	68.2	69.3	69.4	76.2	75.7	73.2	58.1	80.7	76.2	70.2	73.8	77.4	92.5	80.2	69.5	79.2	75.8	74.0	80.0	76.3
SrO	0.12	0.19	0.19	0.14	0.14	0.10	0.14	0.14	0.17	0.11	0.19	0.19	0.16	0.08	0.13	0.13	0.10	0.11	0.10	0.17	0.07	0.14	0.12	0.14	0.23	0.16	0.13	0.16	0.19	0.12	0.11	0.19
F	2.24	3.43	3.41	2.23	3.10	2.18	2.23	2.55	3.03	0.76	2.74	3.32	2.68	2.28	2.25	3.18	3.44	2.57	2.39	2.33	2.53	3.03	2.99	2.69	3.32	3.22	2.01	3.47	3.49	3.21	3.47	2.54
BaO	0.00	0.05	0.00																0.00	0.00	0.00	0.00	0.00	0.03	0.00	0.02	0.00	0.01	0.00	0.00	0.05	0.00
Cl	0.78	0.38	0.36	0.90	0.54	0.50	0.54	0.71	0.57	0.61	0.65	0.46	0.56	0.51	0.55	0.46	0.41	0.50	0.96	0.51	0.65	0.68	0.49	0.60	0.15	0.24	0.24	0.19	0.25	0.38	0.64	0.13
SO_3	0.00	0.88	1.09	2.40	2.09	1.73	1.93	3.36	2.41	1.34	3.03	2.05	1.84	1.75	2.20	2.32	1.72	2.36	1.95	2.31	2.58	1.88	2.06	2.28	0.44	1.60	2.05	1.54	2.08	1.51	1.71	2.37
Sc [ppm]	367	42.8	88.6	239	273	308	294	297	219	223	268	316	204	285	232	197	268	252	210	186	276	188	249	212	7.01	256	317	301	174	237	174	181
V	2.87	3.52	4.12	3.48	1.96	3.97	4.34	3.81	4.78	3.84	13.4	6.44	4.18	4.09	3.39	4.52	3.64	6.21	5.32	18.6	6.99	15.5	3.20	3.72	1.47	12.6	11.3	6.49	5.62	6.54	6.27	18.1
Cr	8.27	0.00	4.73	0.00	0.00	0.00	0.00	0.00	6.23	0.00	22.3	23.5	5.84	18.3	0.00	6.23	0.00	14.1	2.27	0.00	2.99	13.5	1.64	0.00	0.00	6.88	3.94	5.08	3.41	0.01	6.79	0.00
Co	1.85	3.47	0.61	0.00	0.00	0.00	1.33	1.92	0.00	1.18	25.1	0.46	0.48	0.00	1.45	0.71	0.25	0.00	1.82	30.1	2.40	18.6	0.16	1.57	1.18	14.6	7.97	3.41	3.10	6.28	2.97	14.8
Ni	50.2	9.87	2.25	0.00	0.00	0.00	0.00	0.00	0.00	0.00	0.00	0.00	0.00	0.00	0.00	0.00	0.00	0.00	5.42	46.4	7.13	28.9	4.82	0.00	21.3	41.1	21.0	8.59	0.79	24.9	6.89	46.0
Cu	53.6	46.9	32.7	37.6	33.5	23.4	22.9	45.4	32.2	27.8	68.1	28.9	24.8	25.8	22.3	22.1	13.7	29.1	24.0	64.6	31.5	51.8	15.1	16.3	9.09	66.6	50.1	43.0	36.1	42.8	23.3	99.5
Zn	66.1	217	119	81.2	122	91.3	272	103	82.1	81.1	81.2	65.2	56.5	43.2	45.6	59.9	103	38.4	115	129	151	68.4	75.1	69.7	43.0	185	114	62.5	99.6	76.9	194	155
Ga	123	13.8	39.9	129	58.1	63.6	75.4	95.8	79.2	117	120	62.9	94.6	139	105	118	62.0	85.0	167	105	220	176	171	85.2	9.19	152	153	138	133	178	225	81.9
Rb	0.94	1.22	0.36	1.18	1.25	0.46	0.78	1.65	0.90	0.97	10.4	0.00	1.38	1.03	0.88	1.18	0.00	0.36	2.39	5.25	3.15	10.8	0.91	0.47	1.12	5.97	3.90	2.35	1.48	4.36	1.95	8.24
Sr	1081	1288	1404	1614	1440	1107	1240	1458	1321	1364	1671	1472	1347	1316	1452	1548	981	1319	1155	1724	1519	1531	1146	1088	1700	2206	1399	1248	1555	1143	1175	2008
Y	2523	534	1411	6453	3507	3253	3758	8205	4490	4207	5278	4750	5209	5748	5078	6225	3273	4334	4843	2483	6371	4998	4625	2434	134	5491	6627	5609	3697	4964	5694	2756
Zr	179	20.6	21.1	139	114	121	112	127	76.2	68.2	95.0	183	76.2	78.2	82.7	86.3	86.0	77.4	89.0	110	117	61.7	89.5	87.9	2.02	109	117	101	53.6	88.1	56.9	83.1
Nb	0.00	0.04	0.04	0.01	0.00	0.03	0.13	0.02	0.04	0.15	0.74	0.34	0.00	0.04	0.18	0.04	0.09	0.01	0.31	0.56	0.40	1.01	0.07	0.00	0.09	1.10	0.55	0.35	0.11	0.52	0.11	0.39
Ba	15.2	17.4	33.5	16.8	19.9	16.3	17.2	15.7	13.8	18.3	79.2	28.7	15.6	13.8	20.4	20.9	16.1	17.4	15.2	58.3	20.0	35.8	16.3	21.4	9.03	55.7	21.8	17.5	23.4	33.4	21.7	143
La	1262	200	714	3481	1512	1627	2072	2575	2325	2338	3342	2404	2439	2961	3038	3073	1569	2022	2974	1281	3912	2860	2344	1294	75.2	3451	3575	2343	2656	2977	3617	1697
Ce	1031	19.5	163	571	626	535	796	680	823	1398	3600	399	1041	1817	726	988	736	895	717	480	944	1195	978	539	27.8	1391	1345	840	1042	1578	2212	872
Pr	324	56.0	173	932	343	442	512	759	588	608	816	590	722	826	900	1050	490	538	808	355	1063	704	658	307	17.7	848	949	626	574	706	940	373
Nd	1394	222	682	4287	1678	1897	2027	3449	2211	2547	3627	2254	2923	4080	3911	4209	2149	2614	3376	1468	4441	3167	3030	1391	70.8	3681	4121	2925	2686	3173	4315	1705
Sm	314	55.0	164	1033	415	486	539	822	626	592	905	501	819	905	977	961	494	557	902	332	1187	747	660	306	14.3	845	1020	724	607	792	937	364
Eu	88.9	15.3	41.0	289	108	106	128	231	159	156	242	155	206	228	252	263	140	151	213	84.6	280	189	174	84.3	3.52	219	242	187	152	200	234	93.4
Gd	382	68.1	176	1125	449	510	606	975	634	603	806	589	760	947	954	983	513	648	945	410	1243	798	713	335	15.1	870	1029	778	600	858	967	406
Tb	62.4	10.2	29.2	178	70.1	72.7	89.8	153	95.7	119	136	92.6	122	153	149	169	79.6	99.3	155	57.9	204	123	116	53.4	2.87	133	163	118	93.6	144	153	65.3
Dy	382	64.0	196	1013	469	533	603	862	617	662	818	640	753	974	787	1011	525	668	762	359	1002	736	730	360	17.8	801	969	759	559	827	930	434
Ho	86.9	15.7	45.3	216	106	117	121	203	144	149	211	149	177	204	165	213	116	137	164	79.9	216	157	152	75.2	3.84	180	217	166	115	175	194	92.9
Er	258	48.9	120	541	335	323	366	488	365	424	482	359	421	579	408	430	281	444	424	223	558	401	397	206	10.9	514	567	433	295	479	527	261
Tm	36.6	7.04	19.2	67.2	43.7	47.0	56.3	68.7	49.4	57.5	66.2	50.5	55.9	71.7	63.4	59.3	46.4	50.2	57.2	31.7	75.2	53.2	55.2	33.4	1.69	71.2	81.3	63.8	40.3	65.4	69.3	34.7
Yb	291	53.7	141	430	285	327	373	521	384	406	447	391	403	477	336	360	283	384	335	213	441	372	369	249	10.5	483	492	388	291	455	454	235
Lu	46.7	8.05	21.7	67.6	51.3	54.6	50.4	60.6	53.4	52.7	59.1	50.5	50.9	66.3	45.9	45.9	42.8	49.9	50.7	34.2	66.7	51.5	50.5	31.6	1.91	72.1	71.6	59.5	40.1	64.1	63.3	36.3
Hf	1.10	0.09	0.21	1.20	0.43	1.35	1.14	0.58	0.61	0.52	0.65	1.42	0.30	0.46	0.22	0.71	0.43	0.47	0.64	1.22	0.85	0.62	0.78	0.50	0.00	0.77	0.65	0.69	0.29	1.04	0.41	0.41
Ta	0.00	0.00	0.02	0.05	0.00	0.00	0.04	0.13	0.00	0.00	0.02	0.00	0.00	0.05	0.12	0.00	0.04	0.00	0.00	0.00	0.00	0.02	0.00	0.00	0.01	0.04	0.02	0.02	0.00	0.10	0.00	0.02
Pb	0.84	1.89	1.35	2.63	1.08	1.00	1.07	2.36	1.33	0.83	7.27	2.17	1.04	2.24	1.60	1.69	0.75	1.01	1.43	18.3	1.88	7.39	0.55	0.85	0.68	6.84	5.20	1.32	1.36	5.98	3.02	4.69
Th	60.0	1.39	6.30	128	35.7	37.9	37.5	97.3	48.3	92.1	98.3	19.2	136	177	159	115	64.4	48.4	189	48.5	248	122	84.3	12.7	0.77	118	156	85.7	97.5	122	232	36.5
U	18.7	8.81	14.6	24.8	13.3	18.7	18.5	20.7	16.9	14.5	23.7	28.4	19.0	18.1	20.2	18.9	15.1	17.9	21.0	35.9	27.6	9.40	18.8	19.3	1.25	19.5	21.2	20.1	14.3	21.5	19.9	18.5
ΣREY	8482	1377	4094	20685	10000	10331	12097	20053	13565	14321	20835	13374	16103	20036	17790	20041	10736	13590	16725	7893	22003	16552	15052	7700	408	19051	21470	16019	13448	17458	21308	9426
Total (Major and Minor elements)[%]	73.88	92.87	92.44	80.46	87.97	79.58	84.39	81.36	86.11	69.72	85.56	90.40	75.05	76.07	76.42	84.37	82.54	80.20	65.25	86.90	84.43	77.72	80.99	83.85	96.63	87.61	76.34	86.26	83.25	81.10	88.27	82.86

Table S5 (B)

Core No.	KR13-02_PC05								
depth [mbsf]	1.5	2.47	2.47	2.97	2.97	2.97	2.97	2.97	2.97
SiO_2 [%]	57.7	55.4	59.2	59.3	59.5	58.9	58.8	59.5	57.8
TiO_2	0.04	0.02	0.04	0.07	0.05	0.05	0.03	0.07	0.03
Al_2O_3	19.8	18.8	20.8	20.2	20.5	19.7	20.2	20.5	19.5
FeO	0.62	0.34	0.69	0.58	0.66	0.64	0.82	0.82	0.76
MnO	0.00	0.03	0.04	0.04	0.01	0.02	0.00	0.04	0.11
MgO	0.58	0.21	0.33	0.35	0.48	0.36	0.42	0.47	0.64
CaO	0.17	0.22	0.18	0.16	0.10	0.16	0.20	0.16	0.28
Na_2O	6.20	7.68	8.08	8.12	7.05	7.22	7.69	7.39	7.20
K_2O	5.01	4.68	3.83	4.39	4.23	4.52	3.97	4.59	4.98
P_2O_5	0.04	0.12	0.11	0.30	0.04	0.11	0.03	0.16	0.04
Total (Major elements)	90.0	87.4	93.1	93.2	92.5	91.5	92.1	93.4	91.3
Sc [ppm]	3.61	0.00	3.12	3.32	5.09	2.48	2.34	1.07	2.31
V	9.09	3.11	3.16	9.01	12.8	8.47	8.83	9.29	3.07
Cr	1.59	0.00	0.00	12.8	12.5	19.1	4.42	0.00	1.34
Co	0.00	1.43	1.79	3.12	2.01	3.04	2.07	1.64	3.76
Ni	0.00	0.00	0.00	9.49	0.00	0.00	0.00	0.00	0.00
Cu	33.0	18.5	27.6	63.8	26.0	37.3	25.2	32.1	44.8
Zn	13.6	12.7	16.8	21.8	12.6	21.9	13.5	6.08	17.0
Ga	2.86	2.29	2.46	3.50	1.73	2.71	2.51	5.47	4.73
Rb	32.4	36.2	20.7	24.9	37.1	31.5	30.0	26.1	18.7
Sr	16.5	11.4	32.5	25.3	13.5	18.5	31.8	8.91	10.1
Y	4.44	10.5	7.66	80.0	3.26	14.7	2.69	2.79	4.39
Zr	16.5	11.4	18.3	20.2	19.7	15.7	19.8	78.4	15.0
Nb	1.35	1.13	1.21	2.15	1.32	0.75	1.19	27.3	1.90
Ba	91.1	139	108	91.5	131	94.3	109	77.0	77.0
La	2.54	7.57	8.57	24.0	2.25	8.73	2.90	6.80	2.18
Ce	3.74	2.68	8.14	11.7	3.70	4.83	3.67	20.4	10.7
Pr	0.69	1.68	1.47	6.60	0.52	2.29	1.30	0.48	0.51
Nd	2.46	6.29	12.2	33.3	2.14	8.10	2.48	3.08	2.03
Sm	0.56	2.37	0.96	6.00	0.46	0.91	0.79	0.69	0.87
Eu	0.16	0.37	0.60	2.01	0.32	1.37	0.22	0.24	0.08
Gd	0.54	2.51	1.01	5.39	0.57	1.93	0.83	0.75	0.00
Tb	0.12	0.33	0.12	1.71	0.05	0.24	0.06	0.09	0.06
Dy	0.74	1.60	1.33	7.89	0.66	1.68	0.50	1.36	0.45
Ho	0.15	0.43	0.28	1.56	0.12	0.52	0.32	0.13	0.19
Er	0.46	1.27	0.87	4.08	0.42	0.75	0.26	0.67	0.28
Tm	0.09	0.08	0.08	0.58	0.05	0.13	0.06	0.04	0.04
Yb	0.55	0.60	0.75	4.80	0.37	0.82	0.24	0.41	0.23
Lu	0.08	0.16	0.14	0.54	0.14	0.26	0.05	0.06	0.01
Hf	0.59	0.67	0.57	0.26	0.41	0.57	0.33	1.50	0.44
Ta	0.15	0.00	0.06	0.12	0.20	0.00	0.07	0.79	0.14
Pb	1.02	2.59	1.97	3.80	2.60	5.58	2.59	2.45	3.14
Th	0.46	0.11	0.81	0.82	0.33	0.51	0.56	0.50	0.14
U	0.10	0.03	0.06	0.42	0.17	0.20	0.18	0.12	0.08
ΣREY	17.3	38.4	44.1	190	15.0	47.3	16.4	38.0	22.0

Table S6 (A) Normally REY-rich mud

Grain size [μm]	< 20	20 - 37	37 - 75	75 - 125	> 125	> 20 (in total)	Bulk	Bulk (calculated)
Weight distribution [%]	79.11	6.58	7.48	3.54	3.29	20.89	-	
Major Element [%]								
Na_2O	2.05	4.34	3.92	3.28	3.01	3.80	5.04	2.42
MgO	3.04	0.99	1.16	1.64	2.03	1.32	2.37	2.68
Al_2O_3	16.5	16.4	15.2	14.3	13.2	15.1	14.9	16.2
P_2O_5	0.59	1.24	2.17	4.39	1.49	2.14	0.99	0.91
K_2O	3.26	5.37	4.86	4.15	3.70	4.72	3.53	3.57
CaO	1.77	2.37	4.34	6.59	3.37	3.95	2.17	2.23
TiO_2	0.79	0.23	0.25	0.37	0.48	0.30	0.57	0.69
MnO	2.16	0.55	0.57	0.88	7.91	1.77	1.85	2.08
Fe_2O_3	9.43	2.73	3.07	4.96	6.63	3.84	7.15	8.26
Rare Earth Element [ppm]								
La	76.3	134	229	361	129	206	107	103
Ce	130	96.2	154	226	188	153	111	135
Pr	24.0	44.5	76.5	116	42.1	67.6	35.7	33.1
Nd	96.7	183	314	477	173	278	146	135
Sm	23.4	44.8	76.9	117	42.3	68.1	35.6	32.7
Eu	5.76	11.2	19.0	29.0	10.5	16.9	8.79	8.08
Gd	26.2	51.2	87.4	132	48.1	77.4	40.2	36.9
Tb	3.95	7.68	13.2	19.9	7.27	11.7	6.05	5.56
Dy	24.3	47.8	82.1	124	45.6	72.6	37.1	34.3
Ho	4.99	9.81	16.7	25.3	9.38	14.8	7.55	7.05
Er	14.2	27.8	47.5	71.5	26.7	42.1	21.6	20.0
Tm	1.95	3.81	6.54	9.81	3.75	5.79	2.93	2.75
Yb	12.2	23.8	41.0	61.7	24.1	36.4	18.3	17.3
Lu	1.87	3.64	6.24	9.43	3.78	5.57	2.77	2.64
Y	143	282	503	764	268	440	214	205
ΣREY	589	971	1673	2543	1021	1496	795	778

Table S6 (B) Highly REY-rich mud

Grain size [μm]	< 20	20 - 37	37 - 75	75 - 125	> 125	> 20 (in total)	Bulk	Bulk (calculated)
Weight distribution [%]	64.15	3.52	12.38	10.74	9.20	35.85	-	
Major Element [%]								
Na_2O	1.98	3.66	2.72	2.48	3.51	2.94	5.13	2.33
MgO	4.11	1.73	2.08	2.21	3.38	2.42	2.97	3.50
Al_2O_3	22.4	17.9	14.5	13.8	18.3	15.6	16.6	20.0
P_2O_5	1.30	12.6	17.9	9.91	3.80	11.4	5.40	4.91
K_2O	3.42	4.22	3.19	3.19	4.22	3.56	3.43	3.47
CaO	4.10	20.1	28.0	14.7	7.08	17.9	8.86	9.04
TiO_2	1.02	0.48	0.53	0.57	0.76	0.60	0.77	0.87
MnO	2.71	0.72	0.67	1.12	19.2	5.56	2.89	3.73
Fe_2O_3	12.8	5.35	5.96	6.23	10.4	7.12	9.08	10.8
Rare Earth Element [ppm]								
La	232	1363	1885	904	317	1137	572	557
Ce	177	590	814	411	349	552	293	311
Pr	75.2	493	675	327	101	406	177	194
Nd	310	2046	2821	1354	417	1688	738	804
Sm	75.4	461	637	305	103	383	180	186
Eu	18.6	115	159	76.2	25.4	95.4	44.9	46.1
Gd	85.9	529	735	349	118	441	206	213
Tb	12.7	79.2	109	52.0	17.5	65.7	30.8	31.7
Dy	78.4	493	722	324	110	423	190	202
Ho	15.9	101	140	66.1	22.4	83.8	38.8	40.3
Er	44.4	284	392	185	63.6	235	109	113
Tm	5.91	38.0	52.8	24.9	8.63	31.7	14.6	15.1
Yb	35.7	233	324	154	53.8	195	89.1	92.7
Lu	5.34	34.9	48.6	23.0	8.16	29.2	13.4	13.9
Y	478	2955	4090	1967	691	2470	1254	1192
ΣREY	1651	9814	13605	6523	2404	8235	3950	4011

Table S6 (C) Extremely REY-rich mud

Grain size [μm]	< 20	20 - 37	37 - 75	75 - 125	> 125	> 20 (in total)	Bulk	Bulk (calculated)
Weight distribution [%]	55.35	6.71	26.41	10.04	1.49	44.65	-	
Major Element [%]								
Na_2O	1.22	1.91	1.62	1.93	1.84	1.74	3.56	1.45
MgO	2.79	0.83	0.97	0.96	1.91	0.98	2.20	1.98
Al_2O_3	14.7	7.57	6.92	7.64	7.71	7.20	12.1	11.3
P_2O_5	3.85	16.4	17.5	16.0	9.28	16.7	10.4	9.59
K_2O	3.62	2.27	1.79	2.12	2.11	1.95	2.79	2.87
CaO	5.96	22.9	23.9	22.0	13.4	23.0	16.1	13.6
TiO_2	0.67	0.47	0.45	0.33	0.46	0.43	0.49	0.56
MnO	1.92	0.39	0.35	0.90	15.0	0.97	1.50	1.49
Fe_2O_3	7.19	2.73	2.96	2.77	5.20	2.96	5.74	5.30
Rare Earth Element [ppm]								
La	408	1581	1674	1514	739	1593	1014	937
Ce	195	633	644	563	311	613	463	382
Pr	114	474	512	462	220	485	362	280
Nd	487	2057	2211	1995	943	2097	1486	1206
Sm	114	452	486	442	221	462	335	270
Eu	28.7	113	122	111	55.4	116	83.4	67.7
Gd	132	522	560	508	254	532	381	311
Tb	19.5	77.4	83.3	75.3	37.7	79.1	56.4	46.1
Dy	121	479	515	465	235	489	352	286
Ho	24.9	98.4	106	95.5	47.9	100	72.4	58.6
Er	69.6	277	295	267	135	281	203	164
Tm	9.28	36.9	39.6	35.6	18.1	37.6	27.2	21.9
Yb	56.1	224	241	218	113	229	165	133
Lu	8.34	33.5	36.1	32.8	16.9	34.3	24.8	20.0
Y	769	3207	3339	3121	1552	3210	2200	1859
ΣREY	2558	10265	10865	9907	4897	10360	7226	6041

Table S7

Type of Slurry	Normally REY-rich mud Bulk	OF	UF	Bulk (calc.)	Highly REY-rich mud Bulk	OF	UF	Bulk (calc.)	Extremely REY-rich mud Bulk	OF	UF	Bulk (calc.)
Slurry density [g/cm^3]	1.102	1.071	1.197	-	1.101	1.073	1.171	-	1.104	1.060	1.298	-
Flow amount [L]	-	2.74	0.58	-	-	2.93	0.51	-	-	2.85	0.60	-
Particle recovery [%]	-	57.5	42.5	-	-	66.9	33.1	-	-	40.2	59.8	-
Concentration factor of ΣREY	-	-	1.94	-	-	-	2.61	-	-	-	1.85	-
REY recovery rate [%]	-	29.3	70.7	-	-	25.0	75.0	-	-	6.99	93.0	-
Major Element [%]												
Na	6.91	8.31	4.83	6.83	6.68	7.41	4.70	6.51	6.02	8.79	2.99	5.32
Mg	1.72	2.20	1.43	1.87	1.89	2.09	1.30	1.83	1.72	2.36	1.15	1.64
Al	6.90	6.88	7.80	7.27	6.76	7.22	6.43	6.95	5.22	6.09	4.27	5.00
P	0.44	0.22	1.03	0.56	1.57	0.64	4.99	2.08	3.55	0.59	6.64	4.21
K	2.33	2.34	3.23	2.72	2.46	2.45	2.26	2.38	2.11	2.38	1.74	2.00
Ca	1.72	1.39	2.80	1.99	3.76	1.80	9.13	4.23	8.39	2.12	16.1	10.5
Ti	0.38	0.51	0.36	0.44	0.33	0.33	0.29	0.32	0.31	0.36	0.25	0.30
Mn	1.07	1.04	1.26	1.14	1.44	1.57	1.04	1.40	1.54	1.20	2.05	1.71
Fe	4.76	5.38	3.98	4.79	3.99	4.26	2.90	3.81	3.43	4.10	2.66	3.23
Rare Earth Elements [ppm]												
La	111	64.7	216	129	362	156	950	419	738	148	1377	882
Ce	108	91.8	144	114	214	129	467	241	340	104	598	399
Pr	29.6	16.1	60.9	35.1	104	43.2	272	119	212	41.3	390	250
Nd	128	71.2	268	155	447	186	1225	530	947	181	1725	1104
Sm	30.0	15.6	59.7	34.4	104	42.9	279	121	216	40.9	398	254
Eu	14.3	3.83	15.5	8.79	26.1	10.6	69.8	30.2	56.6	10.6	105	67.1
Gd	14.5	18.0	71.6	40.8	116	47.2	310	134	256	48.1	461	295
Tb	5.07	2.64	10.6	6.01	17.4	7.07	46.3	20.0	38.0	7.13	72.1	46.0
Dy	31.7	16.3	65.5	37.2	108	43.9	290	125	239	44.8	432	277
Ho	6.23	3.28	13.6	7.68	21.3	8.66	57.8	25.0	48.7	9.17	89.0	56.9
Er	18.3	9.75	39.5	22.4	60.0	24.3	161	69.7	139	25.7	248	159
Tm	2.58	1.32	5.26	2.99	8.07	3.20	21.8	9.36	18.5	3.40	35.3	22.5
Yb	16.4	8.28	33.3	18.9	49.2	20.1	134	57.8	114	21.0	218	139
Lu	2.37	1.23	4.95	2.81	6.95	2.81	18.7	8.07	16.8	3.02	32.1	20.4
Y	204	105	393	228	672	272	1729	755	1424	307	2721	1750
ΣREE	722	429	1401	842	2315	997	6031	2664	4802	994	8902	5722
Trace Elements [ppm]												
Sc	25.9	22.6	36.2	28.4	50.4	30.3	112	57.5	89.3	29.8	158	106
V	130	145	104	127	104	111	74.3	98.8	89.1	113	76.4	91.0
Cr	35.9	35.7	27.8	32.3	30.3	28.0	29.6	28.5	23.5	31.9	26.2	28.5
Co	161	166	175	170	185	205	124	178	213	171	269	229
Ni	295	173	570	342	361	330	443	368	549	315	844	631
Cu	92.3	328	377	349	268	277	229	261	330	277	396	348
Zn	234	227	224	226	143	150	152	150	166	182	198	191
As	26.2	25.5	27.3	26.3	40.8	28.4	71.5	42.7	42.4	19.6	68.4	48.8
Rb	65.2	68.9	76.0	71.9	77.9	82.7	62.3	76.0	54.6	67.7	38.2	50.1
Sr	217	233	253	242	344	249	634	377	559	246	860	613
Zr	117	146	119	134	126	130	123	128	127	123	125	124
Nb	9.49	11.7	8.61	10.4	10.0	10.2	8.46	9.64	9.28	10.5	8.31	9.20
Mo	79.2	89.9	66.6	80.0	68.6	75.5	41.7	64.3	74.9	79.1	72.4	75.1
Cs	4.48	5.47	3.57	4.66	5.69	6.27	3.54	5.37	3.26	4.75	1.64	2.89
Ba	211	233	229	231	282	298	216	271	241	256	214	231
Hf	2.92	3.52	2.87	3.24	3.00	3.07	2.84	3.00	3.15	3.25	2.93	3.06
Ta	0.38	0.60	0.44	0.53	0.50	0.50	0.47	0.49	0.48	0.58	0.42	0.48
Pb	37.9	44.1	34.1	39.9	40.9	43.7	28.1	38.5	38.5	40.6	36.9	38.4
Th	9.00	6.78	14.2	9.91	19.1	10.1	46.7	22.2	29.1	8.55	49.7	33.1
U	2.10	1.85	2.80	2.25	3.70	2.23	8.08	4.17	6.80	2.21	11.6	7.85
